# Genome evolution of a nonparasitic secondary heterotroph, the diatom *Nitzschia putrida*

**DOI:** 10.1126/sciadv.abi5075

**Published:** 2022-04-29

**Authors:** Ryoma Kamikawa, Takako Mochizuki, Mika Sakamoto, Yasuhiro Tanizawa, Takuro Nakayama, Ryo Onuma, Ugo Cenci, Daniel Moog, Samuel Speak, Krisztina Sarkozi, Andrew Toseland, Cock van Oosterhout, Kaori Oyama, Misako Kato, Keitaro Kume, Motoki Kayama, Tomonori Azuma, Ken-ichiro Ishii, Hideaki Miyashita, Bernard Henrissat, Vincent Lombard, Joe Win, Sophien Kamoun, Yuichiro Kashiyama, Shigeki Mayama, Shin-ya Miyagishima, Goro Tanifuji, Thomas Mock, Yasukazu Nakamura

**Affiliations:** 1 Graduate School of Agriculture, Kyoto University, Kyoto 606-8502, Japan.; 2 Department of Informatics, National Institute of Genetics, Research Organization of Information and Systems, Shizuoka 411-8540, Japan.; 3 Graduate School of Life Sciences, Tohoku University, Sendai 980-8578, Japan.; 4 Department of Gene Function and Phenomics, National Institute of Genetics, Shizuoka 411-8540, Japan.; 5 Université de Lille, CNRS, UMR 8576 – UGSF – Unité de Glycobiologie Structurale et Fonctionnelle, F-59000 Lille, France.; 6 Laboratory for Cell Biology, Philipps University Marburg, Karl-von-Frisch-Str. 8.; 7 SYNMIKRO Research Center, Hans-Meerwein-Str. 6, 35032, Marburg, Germany.; 8 School of Environmental Sciences, University of East Anglia, Norwich Research Park, Norwich, UK.; 9 Graduate School of Humanities and Sciences, Ochanomizu University, Tokyo, Japan.; 10 Department of Clinical Medicine, Faculty of Medicine, University of Tsukuba, Ibaraki 305-8572, Japan.; 11 Graduate School of Human and Environmental Studies, Kyoto University, Kyoto 606-8501, Japan.; 12 Architecture et Fonction des Macromolécules Biologiques (AFMB), CNRS, Université Aix-Marseille, 163 Avenue de Luminy, 13288 Marseille, France.; 13 INRA, USC 1408 AFMB, 13288 Marseille, France.; 14 Department of Biological Sciences, King Abdulaziz University, Jeddah 21589, Saudi Arabia.; 15 The Sainsbury Laboratory, University of East Anglia, Norwich Research Park, Norwich, UK.; 16 Graduate School of Engineering, Fukui University of Technology, Fukui, Japan.; 17 Advanced Support Center for Science Teachers, Tokyo Gakugei University, Koganei, Tokyo, Japan.; 18 Department of Zoology, National Museum of Nature and Science, Tsukuba 305-0005, Japan.

## Abstract

Secondary loss of photosynthesis is observed across almost all plastid-bearing branches of the eukaryotic tree of life. However, genome-based insights into the transition from a phototroph into a secondary heterotroph have so far only been revealed for parasitic species. Free-living organisms can yield unique insights into the evolutionary consequence of the loss of photosynthesis, as the parasitic lifestyle requires specific adaptations to host environments. Here, we report on the diploid genome of the free-living diatom *Nitzschia putrida* (35 Mbp), a nonphotosynthetic osmotroph whose photosynthetic relatives contribute ca. 40% of net oceanic primary production. Comparative analyses with photosynthetic diatoms and heterotrophic algae with parasitic lifestyle revealed that a combination of gene loss, the accumulation of genes involved in organic carbon degradation, a unique secretome, and the rapid divergence of conserved gene families involved in cell wall and extracellular metabolism appear to have facilitated the lifestyle of a free-living secondary heterotroph.

## Teaser

Genomic adaptation to loss of photosynthesis and an osmotrophic lifestyle requires unique evolutionary processes.

## INTRODUCTION

The loss of photosynthesis in photoautotrophs is successful if compensated by a competitive advantage arising from the availability of an extracellular energy source. Hence, many secondary heterotrophs evolve as parasites (*1*–*3*), relying on sufficient resources provided by their hosts. Well-studied examples are the Apicomplexa [e.g., (*4*)], which have lost photosynthesis secondarily. However, the loss of photosynthesis can also lead to free-living secondary heterotrophs, which are as common as parasites (*2*, *5*–*8*). Despite their significance, our knowledge about the evolution of free-living secondary heterotrophs is very limited, and we therefore lack insights into evolutionary processes required for them to thrive without photosynthesis and independently of a resource-providing host. Given that a parasitic lifestyle accelerates the rate of evolution (cf. Red Queen hypothesis) (*9*) and of loss of conserved orthologous genes [e.g., (*10*)], the genome analysis of a nonparasitic secondary heterotroph can provide insights uncompromised by parasite-specific adaptations. Hence, the diatom *Nitzschia putrida*, isolated from mangrove estuaries, is the ideal model to test these hypotheses because it is an example of a free-living secondary heterotroph (*5*, *11*) within the diverse group of largely photoautotrophic diatoms (*12*, *13*). As several genomes of the latter have recently become available including close phylogenetic relatives (*14*–*16*), a genome-based comparative metabolic reconstruction of *N. putrida* promises to reveal fresh insights into what is required to thrive as a free-living secondary heterotroph. Thus, here, we have analyzed the draft genome sequence of *N. putrida*, which provides insight into evolutionary processes underpinning lifestyle shifts from photoautotrophy to free-living heterotrophy in the context of a coastal surface ocean ecosystem.

## RESULTS

### Genome assembly

**Fig. 1.**
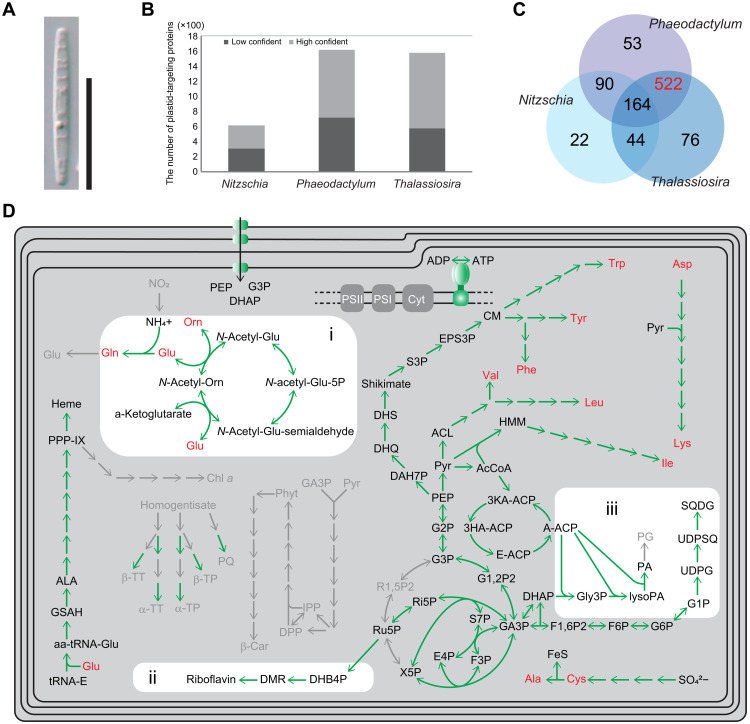
The heterotrophic diatom *N. putrida* and its plastid proteome. (**A**) The frustule view of *N. putrida*. Bar, 10 μm. (**B**) Estimated plastid proteome size in three diatoms. Light and dark gray bars show low and high confident plastid-targeted proteins identified by ASAFind (*22*), respectively. Data of two photosynthetic diatoms *P. tricornutum* and *T. pseudonana* are derived from the previous study (*22*). (**C**) Unique and shared plastid-targeted orthogroups. Highlighted in red is the orthogroup exclusively shared by the two photosynthetic diatoms. (**D**) Predicted metabolic map of the nonphotosynthetic plastid. Representative pathways found in photosynthetic diatom species are shown. Green and light gray arrows show the presence and absence of the responsible protein sequences for the reactions in the genome, respectively. Amino acids are highlighted in red. Abbreviations are described in the Supplementary Materials.

K-mer–based GenomeScope analysis (*17*) with 150–base pair (bp)–long Illumina short reads suggested the genome of *N. putrida* (Fig. 1A) to be diploid (fig. S1A). To provide a high-quality genome with long-range contiguity, PacBio sequencing (RSII platform) was performed, resulting in ≥40-fold coverage. Because of the confirmed diploid nature of the *N. putrida* genome, we have applied the Falcon assembler and Falcon_unzip version 0.5 (*18*) to provide a first draft genome of this species. On the basis of this assembly, we estimated a genome size of 35 Mbp, including 87 scaffolds with an N50 of 860.9 kbp. The longest scaffold was 3.8 Mbp. The heterozygous regions of the genome (alternate contigs) estimated by the Falcon assembler resulted in 12 Mbp, with an N50 of 121 kbp (table S1). The Falcon assembly was error-corrected and polished by approximately 150-fold coverage of Illumina short reads, which were subsequently used for generating the final assembly with Pilon 1.2.2 (*19*) including manual curation.

According to the k-mer–assessed diploid nature of the *N. putrida* genome, the read coverage of the homozygous regions is approximately twofold higher than the read coverage for the heterozygous regions, suggesting the presence of diverged alleles as previously identified in the genome of the photoautotroph diatom *Fragilariopsis cylindrus* (fig. S1, A and B). Thus, most of the diverged allelic variants can be found in the heterozygous regions characterized by the presence of alternate contigs, whereas the regions with no corresponding alternate contigs are homozygous (fig. S1B). On the basis of the analysis with Braker2 version 2.0.3 (*20*), the *Nitzschia* genome comprises 15,003 and 5767 inferred protein-coding loci on the primary and alternate contigs, respectively (table S1). Almost 40% of loci in the genome of *N. putrida* appear to be characterized by diverged alleles. A BUSCOv3 analysis (*21*) revealed the genome to be complete at a level of 90.1% based on the haploid set of genes.

### The loss of photosynthesis

The haploid set of genes was used to reconstruct the nuclear-encoded plastid proteome of *N. putrida* and therefore to reveal the extent of gene loss including key genes of photosynthesis. A comparative analysis of the *N. putrida* plastome (*22*) with its photosynthetic counterparts revealed that more than 50% of nuclear encoded plastid proteins have been lost (Fig. 1B). More than 500 orthogroups (OrthoFinder) (*23*) of nuclear-encoded plastid proteins, which are usually shared between photosynthetic diatoms (*22*), are missing in the predicted plastid proteome of *N. putrida* (Fig. 1C). The missing part of the plastid proteome included genes encoding for proteins of light-harvesting antenna including fucoxanthin-chlorophyll *a*/*c* protein (*fcp*), photosystem II and I (e.g., *psbA*, *psbC*, *psbO*, *psaA*, *psaB*, and *psaD*), the cytochrome *b6*/*f* complex (e.g., *petA*), and carbon fixation (e.g., *rbcS* and *rbcL*) in addition to genes of the Calvin cycle [e.g., phosphoribulokinase (*prk*)]. Furthermore, a substantial number of key genes were missing for the biosynthesis of chlorophyll, carotenoids, and plastoquinones (fig. S1C).

Despite the loss of some of these key photosynthesis genes, there is still a substantial number of genes left encoding common plastid metabolic pathways as known from photosynthetic diatoms, including the generation of adenosine 5′-triphosphate (ATP) by adenosine triphosphatase (ATPase) subunits, which are encoded both in the nuclear and plastid genomes (*24*). Almost all genes encoding for plastid enzymes to synthesize essential amino acids are still encoded in the nuclear genome of *N. putrida*. Furthermore, all genes of the heme pathway have been found, and *N. putrida* appears to be able to synthesize riboflavin. The presence of plastid-targeted transporters (*25*) enables the transport of phosphoenolpyruvate, 3-phosphoglycerate, and dihydroxyacetone-phosphate across the plastid membranes. In addition, our genome-based reconstruction of plastid metabolism identified the biosynthesis pathway for lipids and the ornithine cycle in *N. putrida* (Fig. 1D and fig. S2). The latter has been reported neither in previous transcriptome-based studies with this species (*26*) nor in any other secondary heterotroph ([.g., (*6*, *7*)]. As *N. putrida* has an osmotrophic lifestyle, it relies on dissolved organic matter and nutrients. Thus, the biosynthesis of a variety of metabolic compounds supports the osmotrophic lifestyle, lacking abilities to prey or to parasitize on other organisms.

### Communication between organelles and light-dependent gene expression

**Fig. 2.**
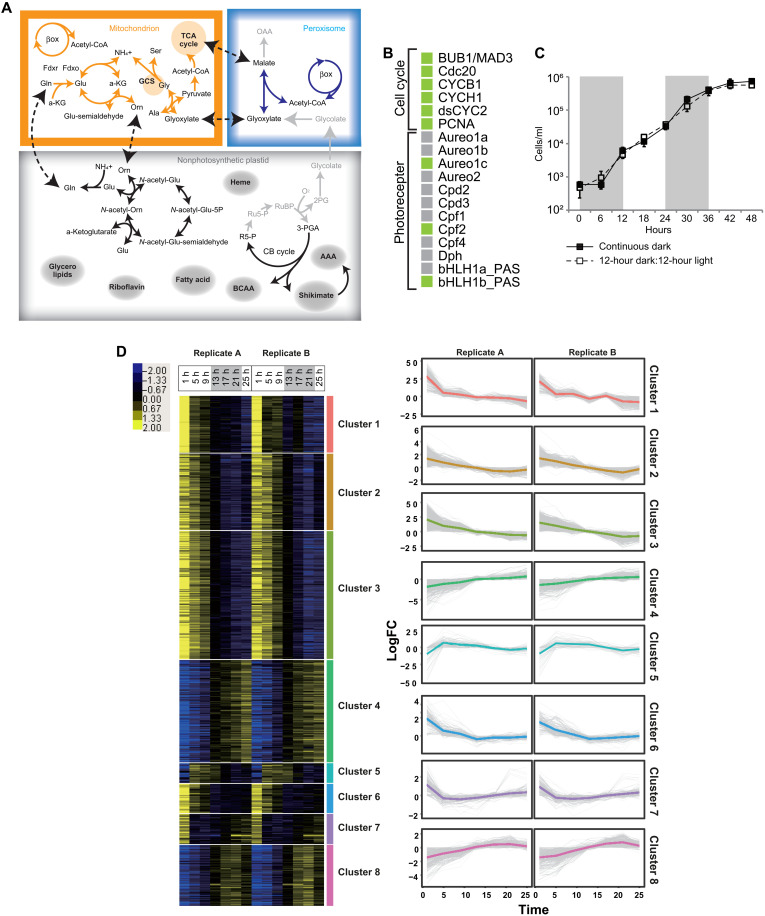
Loss of genes for the plastid-peroxisome metabolic flow and photoreceptors. (**A**) Metabolic interactions between a mitochondrion and a nonphotosynthetic plastid and between a mitochondrion and a peroxisome. Black, orange, and blue arrows show presence of responsible protein sequences for the reactions in a plastid, a mitochondrion, and a peroxisome, respectively, while light gray arrows show absence of responsible protein sequences. Dashed arrows show possible interorganellar metabolic flows. Abbreviations are described in the Supplementary Materials. (**B**) Photoreceptor and cell-cycle genes in the *N. putrida* genome. The other genes are shown in fig. S5. Light green and light gray boxes show the presence and absence of corresponding genes, respectively. (**C**) Growth of the heterotrophic diatom under the different light conditions. Closed boxes show growth in the continuous dark condition, while open boxes show growth in the light-dark condition. Shaded in gray are the dark periods in the light-dark cultivation conditions. (**D**) Left: Heatmap showing the reproducible expression patterns of genes (Pearson’s correlation coefficient < 0.9). *k*-means clustering was calculated for each gene based on reads per kilobase of transcript per million mapped reads (RPKM) + 1 values, which were transformed to log_2_ and centered by median values. Yellow and blue indicate up-regulation and down-regulation of the gene, respectively. Right: The line graphs showing expression pattern of genes in each cluster. The colored line indicates the average value of the expression patterns. LogFC, log fold change.

The lack of CO_2_ fixation in plastids of *N. putrida*—which reduces the amount of amino acids, lipids, and other metabolites to be synthesized—appears to be partially compensated for by the remodeling of metabolic interactions with mitochondria and peroxisomes and by retaining active recycling of nitrogen (Figs. 1 and 2 and figs. S3 and S4). It appears that the nonphotosynthetic plastid of *N. putrida* still exchanges glutamine and ornithine, both of which are important intermediates of the ornithine cycle. All genes for the ornithine-urea cycle have been retained in the *N. putrida* genome. The ornithine-urea cycle is indispensable for nitrogen recycling in photosynthetic diatoms (*27*, *28*), and even after the loss of photosynthesis, nitrogen recycling appears to be essential in *N. putrida* (Fig. 2A) due to its osmotrophic lifestyle. Usually, the ornithine-urea cycle is tightly linked with tricarboxylic acid cycle and/or photorespiration in photosynthetic diatoms (*27*, *28*). However, *N. putrida* is not likely to perform photorespiration (Fig. 2A). The metabolic exchange with the peroxisome through glycolate likely has ceased as phosphoglycolate phosphatase and peroxisomal glycolate oxidase are missing. Thus, photorespiration is unlikely to take place in nonphotosynthetic plastids of *N. putrid* due to the lack of ribulose 1,5-bisphosphate carboxylase/oxygenase and other key enzymes of the Calvin cycle (Fig. 1). Nevertheless, peroxisomes still appear to play a role in *N. putrida* for the production of malate or glyoxylate, which feed into respiratory pathways of the mitochondria to support ATP and NADPH (reduced form of nicotinamide adenine dinucleotide phosphate) production (Fig. 2A).

Light in photosynthetic organisms not only plays a substantial role for photosynthesis generating ATP and NADPH but also regulates cell division, diel cycles, and different signaling processes unlike in many heterotrophic organisms (*29*–*31*). As a consequence, we identified the remaining photoreceptors and cell-cycle regulators such as cyclins and cyclin-dependent kinases (*32*). Although they were still encoded and expressed in the genome of *N. putrida* (Fig. 2B and fig. S5, A and B), we were unable to identify a diel cycle in cell division (Fig. 2C). This suggests that these cel-cycle regulators potentially have neo/subfunctionalized and therefore have a different regulatory role in *N. putrida* unrelated to the diel cycle. The loss of the transcription factor *bHLH-1a* (RITMO1), which has been identified as a master regulator of diel periodicity (*33*), corroborates our finding that *N. putrida* has lost the ability to perform diel cycles. In addition, most of the other photoreceptors known from photosynthetic diatoms have also been lost (Fig. 2B) such as the blue light sensing aureochromes 1a/b, both of which are transcription factors responsible for photoacclimation (*34*). Despite the lack of light-dependent cell-cycle regulation, a few remaining photoreceptors were identified including bHLH1b_PAS, aureochrome 1c, and cryptochrome-DASH/CPF2 (Fig. 2B) (*29*, *35*). Basic ZIP [basic leucine zipper proteins (bZIP)] transcription factors having potentially light-sensitive Per-Arnt-Sim (PAS) domains (bZIP-PAS) (*36*) were also identified in the *N. putrida* genome such as homologs to bZIP6 and bZIP7 of *Phaeodactylum tricornutum* (*37*). The latter homolog has been duplicated and diversified in *N. putrida* (fig. S5C). The presence of bZIP-PAS protein in a heterotrophic eukaryote is not unprecedented as some oomycetes, nonphotosynthetic parasites, have been reported to also encode them in their genomes [e.g., (*38*)]. Although their role in regulating gene expression remains to be investigated in *N. putrida*, light still appears to influence the expression of some genes in this heterotrophic species. Comparative transcriptome analyses every 4 hours during a shift from a light phase to darkness (Fig. 2D) revealed eight clusters characterized by different expression patterns. Furthermore, there was no cluster explicitly representing the light-dependent gene expression patterns as seen in photosynthetic algae [e.g., (*29*, *39*)]. However, one of the clusters contained genes only expressed in the mid-light phase: cluster 7 containing 90 genes (0.6% total). Forty four of them were genes with known functional domains based on a KOG (EuKaryotic Orthologous Groups) analysis, and 21 of them were encoding proteins for substrate import and carbon metabolism (fig. S5D). However, the photoreceptor homologs above—bHLH1b_PAS, aureochrome 1c, and cryptochrome-DASH/CPF2—were not part of this cluster, and there was no explicit trend in their gene expression patterns with respect to changes between light and dark conditions.

### The genetic toolkit for the evolution of nonparasitic secondary heterotrophy

Despite the loss of many nuclear genes and their families, the genome size of *N. putrida* is not significantly different to photosynthetic relatives such as *F. cylindrus* and *P. tricornutum* and the more distantly related diatom *Thalassiosira pseudonana* (table S1). This is distinct from evolutionary trends observed in parasitic eukaryotes that have lost photosynthesis as they have smaller genomes encoding smaller gene families compared to their photosynthetic relatives (fig. S6). By comparing KOGs of paralogous proteins, there was no significant difference in the number of unique KOG IDs between these four diatom species (fig. S7, A and B). However, when we compared the number of paralogous proteins assigned to each KOG ID, there were several KOG categories for which *N. putrida* had a higher number of paralogous proteins compared to the other diatom species: nucleotide transport (F), transcription (K), signal transduction (T), intracellular trafficking, secretion, vesicular transport (U), and cytoskeleton (Z) (fig. S7C). Even after normalization by total gene numbers, nucleotide transport (F), signal transduction (T), and cytoskeleton (Z) genes were more abundant in the *N. putrida* genome (fig. S8A). This observation was corroborated by *N. putrida*–specific enrichment of Pfam domains such as adenylate/guanylate cyclase and cyclic nucleotide esterase, leucine-rich repeat (LRR), and glycosyl/galactosyl transferase domains (fig. S8B).

A microbial heterotroph acquires nutrients either by phagotrophy, the preferred nutrition of many parasites, or by osmotrophy. The latter requires uptake of dissolved organic compounds by osmosis as realized by bacteria and fungi, for instance (*40*, *41*). As *N. putrida* grows well under axenic conditions (*5*, *42*), it is likely an osmotroph, dependent on the uptake of dissolved organic compounds across the silicified cell wall and the plasma membrane. As realized by osmotrophic fungi, *N. putrida* may even be able to degrade higher–molecular weight compounds extracellularly to be subsequently taken up as individual molecules by specific transporters or even osmosis (*40*, *41*). Thus, it is likely that cell wall, membrane, and secreted proteins were diversified in *N. putrida* compared to photosynthetic diatoms to facilitate osmotrophy.

**Fig. 3.**
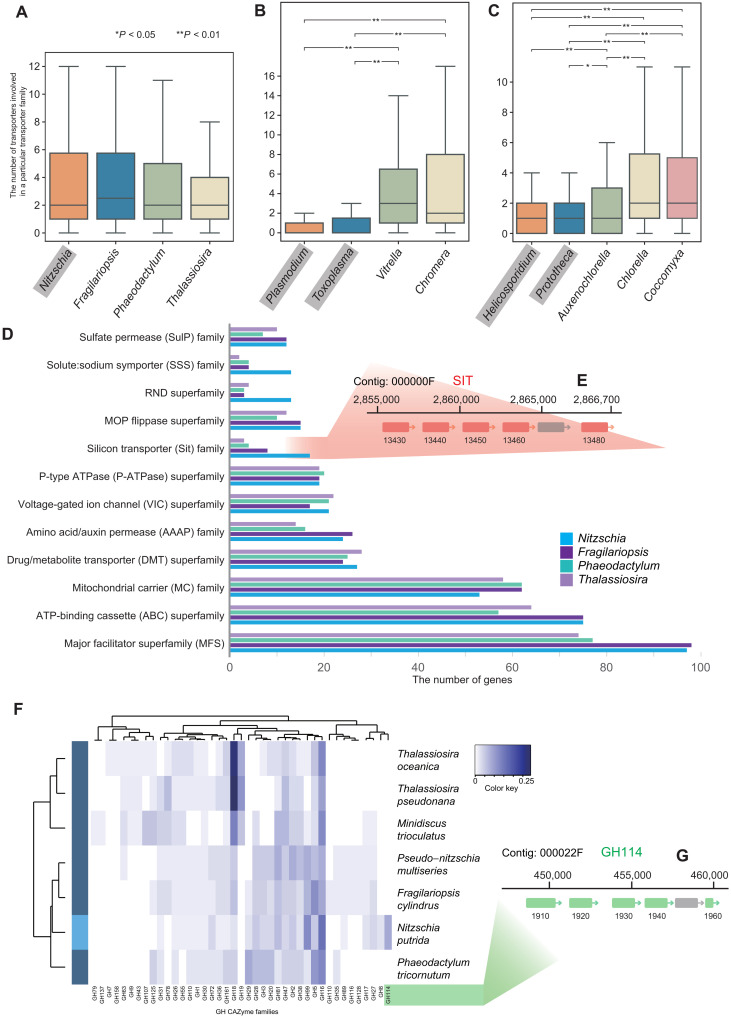
Diversity of transporters and carbohydrate active enzymes in *N. putrida*. (**A**) Distribution of the number of transporters in each transporter family of diatoms. Differences in the distributions among species were tested by the Wilcoxon signed-rank test corrected with the Benjamini-Hochberg procedure (*P* < 0.05), but there is no significant difference. Outliers were omitted in the boxplot. Nonphotosynthetic species are highlighted in gray. (**B**) Distribution of the number of transporters in each transporter family of Alveolata. Details are described in (A). (**C**) Distribution of the number of transporters in each transporter family of green algae (Trebouxiophyceae). Details are described in (A). (**D**) The gene number of transporters in the 12 most abundant transporter families of *N. putrida*. (**E**) Silicon transporter (SIT) genes tandemly located in the contig 000000F. SIT genes are highlighted in light red with the gene IDs. (**F**) Glycoside hydrolase (GH) families from the Carbohydrate Active enZyme (CAZy) database focused on diatoms. The diagram shows a heatmap of CAZyme prevalence in each taxon (number of a particular CAZyme family divided by the total number of CAZyme genes in the organism); the white to blue color scheme indicates low to high prevalence, respectively. Dendrograms (left and top) show respectively the relative taxa proximity with respect co-occurrence of CAZyme families and the co-occurrence of CAZyme families with one another within genomes. (**G**) GH114 genes tandemly located in the contig 000022F. GH114 genes are highlighted in light green with the gene IDs.

To address this hypothesis, we analyzed the enrichment of paralog proteins and differences in nutrient transporters involved in the uptake of dissolved organic compounds such as solute carriers. A comparison to photosynthetic diatoms and parasitic nonphotosynthetic algal species [*Prototheca* and *Helicosporidium* (green algae), and the apicomplexans *Plasmodium* and *Toxoplasma*] has revealed that *N. putrida* has a unique composition of genes encoding transporters, which is therefore different to photosynthetic algae and parasitic nonphotosynthetic algal species (Fig. 3, A to C). For instance, the number of genes encoding silicon transporters (SITs), solute symporters, and the resistance-nodulation–cell division superfamily was more than twice as abundant in *N. putrida* compared to photosynthetic diatom species (Fig. 3D and fig. S9A). However, in contrast to the difference between *N. putrida* and photosynthetic diatom species, there is no enrichment of particular transporters in parasitic algal species when compared to their photosynthetic relatives (fig. S9, B and C).

Expansion of those gene families may, at least partly, have been achieved by recent tandem duplications (Fig. 3E). To gain insight into when the expansion had occurred, we performed a coalescence analysis, which revealed that SITs in *N. putrida* began to expand around 3.3 million years (Ma) ago [1.2 to 6.6, 95% confidence interval (CI)], while divergence from another nonphotosynthetic diatom *N. alba* is estimated to have occurred around 6.67 Ma ago (2.5 to 11.5, 95% CI; fig. S10). The split between *F. cylindrus* and *P. multiseries*, which was used to date the tree, was estimated at 9.7 Ma ago (7.6 to 11.6, 95% CI).

Thus, the recent expansion of SITs suggests neo/subfunctionalization of the gene family in response to the change in lifestyle. The divergence rate of SIT genes was much larger than that of control genes (e.g., myosin), indicating that SIT diversification might have contributed to the adaptation of the heterotrophic lifestyle. In support of this hypothesis, we detected several sites under positive selection in different members of the SIT family (table S2), which implies that the evolution of those genes may have been driven by diversifying selection.

The solute sodium symporters are estimated to have diverged around 7.5 Ma ago (3.8 to 11.1, 95% CI), markedly earlier than the SIT gene family. Although the divergence rate is also larger than that of control genes (fig. S10), we did not find evidence of diversifying selection in this gene family. The differences between these two families of transporters suggest that their expansion might have occurred in a stepwise manner and driven by different evolutionary forces.

Furthermore, although the overall carbohydrate-active enzyme (CAZyme) family composition of *Nitzschia* was not different from that of photosynthetic diatoms (fig. S11), families encoding β-glycoside hydrolase (GH8), laminarinase (GH16_3), pectinase (GH28), β-glucanase (GH72), α-mannan hydrolyzing enzymes (GH99), and β-1,2-glucan hydrolytic enzymes (GH114) were enriched in *N. putrida* compared to photosynthetic species (Fig. 3F). Expansion of these families might, at least partly, have been achieved by recent tandem duplications (Fig. 3G), suggesting an important role of these genes for the heterotrophic lifestyle of *N. putrida*. Notably, more than one-third of proteins assigned to the above six CAZyme families are predicted to be secreted in *N. putrida* (see below). The CAZyme compositions suggest that *N. putrida* might be able to degrade extracellular polysaccharides such as ß-1,3 glucans (e.g., lichenin, paramylon, callose, and laminarin), starches, β-1,2-glucans, pectin, and α-mannan. As *N. putrida* has been isolated from disintegrating mangrove leaves in a paddle (*5*, *42*), this species might play a role in degrading dead leaves and therefore facilitating carbon recycling in mangroves. To gain first insight into how transcription of CAZyme genes is regulated by different carbon sources, we performed comparative transcriptome analyses with starved *N. putrida* cells in comparison to cells growing on glucose and starch. However, we found that only a limited number of genes encoding CAZymes were differentially expressed (table S3). About half of these genes were up-regulated in response specifically to starch as a carbon source, while only one CAZyme gene was up-regulated in response to glucose (table S3). This observation suggests that most of the CAZymes in *N. putrida* are not for the utilization of glucose and only very few for starch utilization. Arguably, providing a very limited set of organic substrates does not reflect the complexity of organic carbon provided by disintegrating leaves in a mangrove ecosystem. Hence, this might be the main reason for the limited transcriptome response observed in our experiments.

### The predicted secretome of the nonparasitic, free-living secondary heterotroph *N. putrida*

**Fig. 4.**
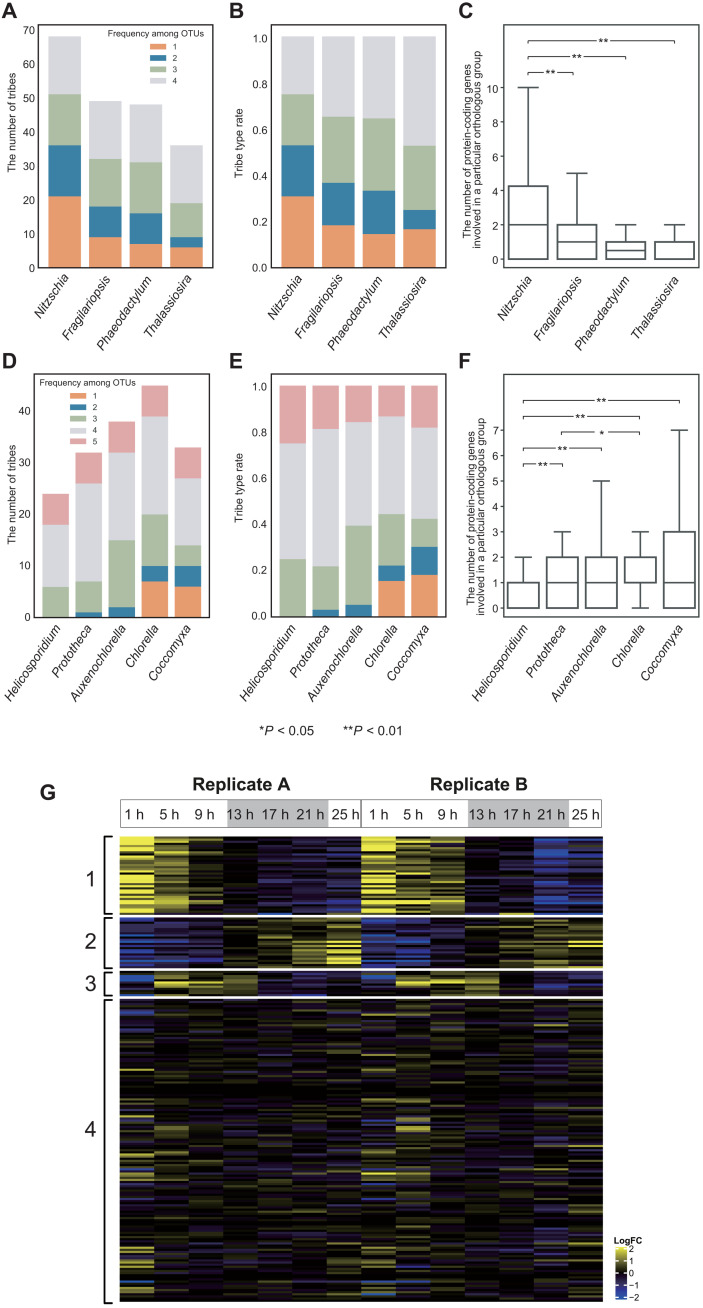
Secretome of nonphotosynthetic algae. (**A**) The number of secretome tribes of diatoms, including at least four sequences, clustered by TribeMCL (*45*). Different colors represent tribe categories as follows: 1, species specific tribes; 2 to 4, tribes shared by two to four species, respectively. OTUs, operational taxonomic unit. (**B**) Proportion of each tribe category in diatoms. Details are described in (A). (**C**) Distribution of the number of protein sequences in each secretome tribe in diatoms. Outliers were omitted in the boxplot. The Wilcoxon signed-rank test corrected with the Benjamini-Hochberg procedure was used for tests of statistical significance. (**D**) The number of secretome tribes in green algae (trebouxiophytes), including at least four sequences, clustered by TribeMCL (*45*). Different colors represent tribe categories as follows: 1, species specific tribes; 2 to 5, tribes shared by two to five species, respectively. (**E**) Proportion of each tribe category in green algae. Details are described in (D). (**F**) Distribution of the number of protein sequences in each secretome tribe in green algae. Details are described in (C). (**G**) Expression of the 10 largest tribes in *N. putrida* during the 25 hours of cultivation. Genes in the tribes could be divided into four clusters. Details are described in Fig. 2D.

Given that the secretome plays an important role for substrate degradation and subsequent uptake of low–molecular weight compounds in osmotrophs (*40*), we conducted a comparative analysis to predict secreted proteins of *N. putrida* in silico by identifying proteins with N-terminal signal peptides and a lack of transmembrane domains. The resulting proteins were clustered using TribeMCL (*43*), and plastid- and lysosome-localized proteins were subsequently removed using ASAFind according to their characteristic targeting motifs (*22*) and Pfam domains. The number of putatively secreted proteins is 978, 998, 596, and 718 in *N. putrida*, *F. cylindrus*, *P. tricornutum*, and *T. pseudonana*, respectively, which corresponds to between 5 and 7% of the total number of genes in their genomes (fig. S12A). Nevertheless, there were significant differences when we compared the diversity of proteins between these four diatom species (Fig. 4, A and B); *N. putrida*, on average, had a significantly higher number of proteins per tribe than any of the other diatom species (two-sided Wilcoxon signed-rank test; *P* < 0.01; Fig. 4C). In particular, proteins involved in heterotrophy such as organic matter degradation/modification including CAZymes and peptidases were more abundant in *N. putrida* than in the photosynthetic diatom genomes (188 in *N. putrida*, 142 in *F. cylindrus*, 118 in *P. tricornutum*, and 101 in *T. pseudonana*; fig. S12A). This is in contrast to parasitic green algae because their predicted secretomes are smaller than those of their photosynthetic relatives and show no explicit enrichment of secretome proteins per tribe (Fig. 4, D to F).

The most common secreted proteins in *N. putrida* are LRR-containing proteins (fig. S12B), many of which contain additional domains such as tegument and glycoprotein domains, suggesting an increased functional diversity (fig. S13). Only very few LRR-containing proteins were identified in the predicted secretomes of the photosynthetic diatoms, indicating that signal peptide–dependent secretion of abundant and diverse LRR-containing proteins may be an essential requirement in this secondary heterotroph, such as for environmental signaling (*44*). In addition to LRR-containing proteins, the top 10 most enriched proteins in *N. putrida* were von Willebrand factor type D (VWFD) proteins involved in adhesion or clotting, two types of endopeptidases, trypsin and leishmanolysin (cell-surface peptidase of the human parasite *Leishmania*), intradiol ring-cleavage dioxygenase protein for degradation of aromatic compounds, methyltransferase, and four proteins with unknown function (fig. S12B). LRR-containing proteins and VWFDs might play important roles in *N. putrida* for attaching to disintegrating mangrove leaves (*5*, *42*, *45*). Endopeptidases and aromatic compound degradation may facilitate the utilization of their complex carbon compounds.

Furthermore, transcriptional dynamics of the predicted secretome over a diel cycle (Fig. 2) revealed the presence of four different clusters. Genes in cluster 1 were transcribed at the beginning of the first light phase and genes in cluster 2 at the end of the dark phase and into the second light phase (Fig. 4G). Genes of cluster 3 were most strongly expressed in the middle and end of the first light phase, whereas genes in cluster 4 were relatively weakly expressed throughout day and night. These results suggest that stimuli including light and/or nutrients play a role in the regulation of these genes, which might either be a relict from the photosynthetic ancestor or a response to diel cycles of organic substances in the aquatic system occupied by *N. putrida*. There is only weak evidence of lateral transfer of secretome genes in *N. putrida* (figs. S14 and S15) with five genes of potential lateral origin (figs. S14 and S15). Thus, the origin of most secretome proteins in *N. putrida* likely was derived vertically from homologs of a photosynthetic ancestor.

## DISCUSSION

*N. putrida* experienced a series of genetic adaptations toward a heterotrophic lifestyle. This diatom species took a step backward in one of the major evolutionary transitions, from photoautotrophs to heterotrophs, potentially relaxing selection on some of the now redundant gene networks and their functions. As expected, more than 50% of nuclear encoded plastid proteins have been lost in the *N. putrida* plastid proteome in comparison to its photosynthetic counterparts (*22*). However, the total number of genes (~15,000) fell within the range of photosynthetic microalgae, and we found no evidence of pseudogene formation, genome streamlining [e.g., (*46*)], gene family contraction (cf. birth-and-death hypothesis) (*47*), or reductive genome evolution (Black Queen hypothesis) (*48*). The relatively large genome size is not unexpected given that *N. putrida* is a free-living osmotroph. This free-living lifestyle in a complex and highly variable coastal marine environment likely is the reason why a substantial number of genes including some photoreceptors, cell cycle regulators, and common plastid metabolic pathways usually present in photosynthetic diatoms have remained. Although some of the latter genes were still expressed, *N. putrida* appears to lack a diel growth cycle, which suggests that these cell-cycle regulators have neo/subfunctionalized. However, as a certain number of genes still appear to be regulated by light, osmotrophy potentially benefits from diel fluctuations of resources such as dissolved organic carbon in aquatic environments (*49*–*51*). For photoautotrophs, it is important to regulate the cell cycle in accordance with diel cycles for optimizing photosynthesis and therefore cell proliferation (*29*–*31*, *52*, *53*). Without being reliant on light as its primary energy source, the osmotroph *N. putrida* no longer requires coordinating its cell cycle with diel cycles. Thus, after the loss of photosynthesis, the strict light-dependent regulation of gene expression might have become less important and gene expression therefore may have become predominantly regulated by other stimuli. Many photoreceptors are missing, but duplication of genes for bZIP transcription factors with PAS domains and genes for signal transduction and cellular regulatory roles such as adenyl/guanyl cyclase and cyclic nucleotide esterase domains was enriched in the *N. putrida* genome. Furthermore, the peroxisome-plastid interaction is no longer required after the loss of photosynthesis, giving rise to loss of carbon fixation in the context of glycolate recycling. In contrast, the ornithine-urea cycle likely remains to be functional to facilitate nitrogen recycling.

Gene family expansions and neo/subfunctionalizations appear to have played a prominent role in the adaptation to its different lifestyle given that many proteins predicted to be secreted have diversified in *N. putrida*, possibly to facilitate osmotrophy. Together, the marked change of lifestyle associated with the “devolution” did not result in reductive genome evolution as known from nonphotosynthetic plastid-bearing parasites.

## METHODS

### Cultivation, DNA and RNA extraction, and sequencing

*N. putrida* NIES-4239 was cultivated in the Daigo’s IMK medium (Wako) including 1% Luria-Bertani medium based on the artificial seawater made with MARINE ART SF-1 (Osaka Yakken Co.) at 20°C under the 12-hour light and 12-hour dark conditions: 50 μmol photons/m^2^ per second with plant cultivation light-emitting diode light (BC-BML3, Biomedical Science). DNA was extracted with the Extrap Soil DNA Kit Plus version 2 (Nippon Steel). Total DNA was subjected to library construction with TruSeq DNA PCR-Free (350; Illumina) and to 151-bp paired-end sequencing by HiSeqX, resulting in 660 million paired-end reads, and to PacBio RSII, with SMRT cell 8Pac v3 and DNA Polymerase Binding Kit P6 v2, in Macrogen, resulting in 1.3-Gb subreads. Total RNA was extracted with TRIzol (Sigma-Aldrich) according to the manufacturer’s instruction and was subjected to library construction with TruSeq RNA Sample Prep Kit v2 (Illumina) and 101-bp paired-end sequencing by HiSeq 2500, resulting in 107.5 million paired-end reads.

### Genome assembly and construction of gene models

PacBio reads were assembled into contigs using Falcon (version 0.7.0) (*18*) with a length cutoff of 7000 bp for seed reads and an estimated genome size of 33 Mbp. Genome size estimation was performed on the GenomeScope web server (http://qb.cshl.edu/genomescope/) based on the k-mer frequency distribution of Illumina reads calculated by JellyFish version 2.2.6 with a k-mer size of 21. The resultant primary and associate contigs were then subjected to Falcon_unzip (version 0.5.0) (*18*), generating partially haplotype-phased contigs (primary contigs) and fully phased contigs (haplotigs). The assembly was polished using PacBio reads and Quiver program, followed by single-nucleotide polymorphism (SNP) and short insertion-deletion (indel) error correction using Pilon (version 1.2.2) with Illumina reads mapped by the Burrows-Wheeler Aligner (version 0.7.15) (*19*). Indel errors in the vicinity of hetero-SNPs were further fixed manually, as they were difficult to be automatically corrected. Contigs derived from plastid and mitochondrial genomes were identified using BLASTN and separated from contigs derived from the nuclear genome.

RNA sequencing reads were trimmed under the parameters of ILLUMINACLIP:TruSeq3-PE.fa:2:30:10, LEADING:20 TRAILING:20, SLIDINGWINDOW:4:15, and MINLEN:75 using Trimmomatic (version 0.36) (*54*). The trimmed reads aligned to the assembled contigs using HISAT2 (version 2.0.4) (*55*). They were provided to BRAKER2 gene annotation pipeline (version 2.0.3) (*20*) as training data to be used for ab initio prediction of protein-coding genes. In addition, PASA (version 2.3.3) (*56*) was used to generate transcript-based gene models by integrating de novo transcriptome assembly and genome-guided assembly using Trinity (version 2.5.0) (*57*). The genome-guided assembly used the mapping result from HISAT2 with --dta option. TransDecoder (version 5.0.2) (*58*) was used to extract protein coding regions from PASA result with the alignment files from BlastP (version 2.7.1) (*59*) against UniRef90 with -evalue 1e-5 option and hmmscan (http://hmmer.org/, version 3.1b2) against Pfam (*60*) database.

The gene models that overlapped with the results from BRAKER were removed using BlastP with evalue 1e-5 option, and the remaining gene models were merged with the BRAKER gene models to generate the final gene annotation. Transposable elements in the NIES-4239 genome were searched by RepeatMasker (version 4.9.0) using Dfam3.1 and RepBase-20170127 as reference repeat libraries (*61*). The predicted gene set was available in Dryad (https://doi.org/10.5061/dryad.j3tx95xft).

The integrity of gene annotation was assessed by BUSCO (version 3.0.2) (*21*) and the Eukaryota odb9 (version 2) dataset. The manipulation of SAM/BAM file was used by SAMtools (version 1.9). The sequence files of gene region from gff file were used by GffRead (version 0.9.11) (*62*).

Organellar genome annotation was performed by comparison with previously sequenced organellar genomes of nonphotosynthetic diatoms (*24*). Gene sets and their arrangements of the plastid and mitochondrial genomes sequenced in this study were found to be identical to previously sequenced nonphotosynthetic diatoms (*24*). Assembled genomes were deposited to DNA Data Bank of Japan (http://getentry.ddbj.nig.ac.jp/) under the accession numbers BLYE01000001 to BLYE01000234 for the nuclear genome, LC600866 for the mitochondrial genome, and LC600867 for the plastid genome.

### Functional annotation

The predicted protein coding genes were annotated using InterProScan, and RPS-BLAST search was performed against KEGG orthology database (*63*, *64*). KO identifiers for Kyoto Encyclopedia of Genes and Genomes (KEGG) metabolic pathways were assigned using KEGG Automatic Annotation Server (*65*). Transporter proteins were annotated with TransportTP (*66*) followed by manual curation. Reference proteome datasets for three photosynthetic diatom species were obtained from the JGI Genome Portal: *P.* *tricornutum* CCAP 1055/1 v2.0 (Phatr2_bd_unmapped_GeneModels_FilteredModels1_aa.fasta and Phatr2_chromosomes_geneModels_FilteredModels2_aa.fasta, 10,402 protein sequences in total), *T. pseudonana* CCMP 1335 (Thaps3_bd_unmapped_GeneModels_FilteredModels1_aa.fasta and Thaps3_chromosomes_geneModels_FilteredModels2_aa.fasta, 11,776 sequences), and *F. cylindrus* CCMP 1102 (Fracy1_GeneModels_FilteredModels3_aa.fasta, 21,066 sequences). KEGG and KOG annotation was performed with them in the same manner as NIES-4239. Other details for annotation of CAZyme, cyclins, cyclin-dependent kinases, bZIP transcription factors, photoreceptor proteins, mitochondrial proteins, plastid proteins, and secretome proteins are described in the Supplementary Materials. Evolutionary analyses, comparative transcriptome analyses under the 12-hour light and 12-hour dark condition, those in different carbon sources, and biochemical experiments for lipids, fatty acids, and quinones are also described in the Supplementary Materials. Transcriptome data obtained in this study were deposited to DNA Data Bank of Japan (https://ddbj.nig.ac.jp/resource/bioproject/PRJDB11016 and https://ddbj.nig.ac.jp/resource/bioproject/PRJDB12553).

## Supplementary Materials

### This PDF file includes:

Supplementary Text Figs. S1 to S15 Tables S1 to S3 References

## Notes

View/request a protocol for this paper from *Bio-protocol*.

## References

[1] J. M. Freesec, C. E. Lane, Parasitism finds many solutions to the same problems in red algae (Florideophyceae, Rhodophyta). Mol. Biochem. Parasitol. 214, 105–111 (2017). 28427949 10.1016/j.molbiopara.2017.04.006

[2] L. Hadariová, M. Vesteg, V. Hampl, J. Krajčovič, Reductive evolution of chloroplasts in non-photosynthetic plants, algae and protists. Curr. Genet. 64, 365–387 (2018). 29026976 10.1007/s00294-017-0761-0

[3] J. Janouškovec, G. G. Paskerova, T. S. Miroliubova, K. V. Mikhailov, T. Birley, V. V. Aleoshin, T. G. Simdyanov, Apicomplexan-like parasites are polyphyletic and widely but selectively dependent on cryptic plastid organelles. eLife 8, e49662 (2019). 31418692 10.7554/eLife.49662 PMC6733595

[4] J. C. Kissinger, B. P. Brunk, J. Crabtree, M. J. Fraunholz, B. Gajria, A. J. Milgram, D. S. Pearson, J. Schug, A. Bahl, S. J. Diskin, H. Ginsburg, G. R. Grant, D. Gupta, P. Labo, L. Li, M. D. Mailman, S. K. McWeeney, P. Whetzel, C. J. Stoeckert Jr., D. S. Roos, The *Plasmodium* genome database. Nature 419, 490–492 (2002). 12368860 10.1038/419490a

[5] R. Kamikawa, N. Yubuki, M. Yoshida, M. Taira, N. Nakamura, K. Ishida, B. S. Leander, H. Miyashita, T. Hashimoto, S. Mayama, Y. Inagaki, Multiple losses of photosynthesis in *Nitzschia* (Bacillariophyceae). Phycol. Res. 63, 19–28 (2015).

[6] R. G. Dorrell, T. Azuma, M. Nomura, G. A. de Kerdrel, L. Paoli, S. Yang, C. Bowler, K. Ishii, H. Miyashita, G. H. Gile, R. Kamikawa, Principles of plastid reductive evolution illuminated by nonphotosynthetic chrysophytes. Proc. Natl. Acad. Sci. U.S.A. 116, 6914–6923 (2019). 30872488 10.1073/pnas.1819976116 PMC6452693

[7] M. Kayama, J. F. Chen, T. Nakada, Y. Nishimura, T. Shikanai, T. Azuma, H. Miyashita, S. Takaichi, Y. Kashiyama, R. Kamikawa, A non-photosynthetic green alga illuminates the reductive evolution of plastid electron transport systems. BMC Biol. 18, 126 (2020). 32938439 10.1186/s12915-020-00853-w PMC7495860

[8] M. Kayama, K. Maciszewski, A. Yabuki, H. Miyashita, A. Karnkowska, R. Kamikawa, Highly reduced plastid genomes of the non-photosynthetic dictyochophyceans *Pteridomonas* spp. (Ochrophyta, SAR) are retained for tRNA-Glu-based organellar heme biosynthesis. Front. Plant Sci. 11, 602455 (2020). 33329672 10.3389/fpls.2020.602455 PMC7728698

[9] L. Van Valen, Molecular evolution as predicted by natural selection. J. Mol. Evol. 3, 89–101 (1974). 4407466 10.1007/BF01796554

[10] G. Sun, Y. Xu, H. Liu, T. Sun, J. Zhang, C. Hettenhausen, G. Shen, J. Qi, Y. Qin, J. Li, L. Wang, W. Chang, Z. Guo, I. T. Baldwin, J. Wu, Large-scale gene losses underlie the genome evolution of parasitic plant *Cuscuta australis*. Nat. Commun. 9, 2683 (2018). 29992948 10.1038/s41467-018-04721-8 PMC6041341

[11] C. W. Li, B. E. Volcani, Four new apochlorotic diatoms. Br. Phycol. J. 22, 375–382 (1987).

[12] C. B. Field, M. J. Behrenfeld, J. T. Randerson, P. Falkowski, Primary production of the biosphere: Integrating terrestrial and oceanic components. Science 281, 237–240 (1998). 9657713 10.1126/science.281.5374.237

[13] D. G. Mann, The species concept in diatoms. Phycologia 38, 437–495 (1999).

[14] E. V. Armbrust, J. A. Berges, C. Bowler, B. R. Green, D. Martinez, N. H. Putnam, S. Zhou, A. E. Allen, K. E. Apt, M. Bechner, M. A. Brzezinski, B. K. Chaal, A. Chiovitti, A. K. Davis, M. S. Demarest, J. C. Detter, T. Glavina, D. Goodstein, M. Z. Hadi, U. Hellsten, M. Hildebrand, B. D. Jenkins, J. Jurka, V. V. Kapitonov, N. Kröger, W. W. Y. Lau, T. W. Lane, F. W. Larimer, J. C. Lippmeier, S. Lucas, M. Medina, A. Montsant, M. Obornik, M. S. Parker, B. Palenik, G. J. Pazour, P. M. Richardson, T. A. Rynearson, M. A. Saito, D. C. Schwartz, K. Thamatrakoln, K. Valentin, A. Vardi, F. P. Wilkerson, D. S. Rokhsar, The genome of the diatom *Thalassiosira pseudonana*: Ecology, evolution, and metabolism. Science 306, 79–86 (2004). 15459382 10.1126/science.1101156

[15] C. Bowler, A. E. Allen, J. H. Badger, J. Grimwood, K. Jabbari, A. Kuo, U. Maheswari, C. Martens, F. Maumus, R. P. Otillar, E. Rayko, A. Salamov, K. Vandepoele, B. Beszteri, A. Gruber, M. Heijde, M. Katinka, T. Mock, K. Valentin, F. Verret, J. A. Berges, C. Brownlee, J.-P. Cadoret, A. Chiovitti, C. J. Choi, S. Coesel, A. De Martino, J. C. Detter, C. Durkin, A. Falciatore, J. Fournet, M. Haruta, M. J. J. Huysman, B. D. Jenkins, K. Jiroutova, R. E. Jorgensen, Y. Joubert, A. Kaplan, N. Kröger, P. G. Kroth, J. La Roche, E. Lindquist, M. Lommer, V. Martin-Jézéquel, P. J. Lopez, S. Lucas, M. Mangogna, K. McGinnis, L. K. Medlin, A. Montsant, M.-P. O.-L. Secq, C. Napoli, M. Obornik, M. S. Parker, J.-L. Petit, B. M. Porcel, N. Poulsen, M. Robison, L. Rychlewski, T. A. Rynearson, J. Schmutz, H. Shapiro, M. Siaut, M. Stanley, M. R. Sussman, A. R. Taylor, A. Vardi, P. von Dassow, W. Vyverman, A. Willis, L. S. Wyrwicz, D. S. Rokhsar, J. Weissenbach, E. V. Armbrust, B. R. Green, Y. Van de Peer, I. V. Grigoriev, The *Phaeodactylum* genome reveals the evolutionary history of diatom genomes. Nature 456, 239–244 (2008). 18923393 10.1038/nature07410

[16] T. Mock, R. P. Otillar, J. Strauss, M. McMullan, P. Paajanen, J. Schmutz, A. Salamov, R. Sanges, A. Toseland, B. J. Ward, A. E. Allen, C. L. Dupont, S. Frickenhaus, F. Maumus, A. Veluchamy, T. Wu, K. W. Barry, A. Falciatore, M. I. Ferrante, A. E. Fortunato, G. Glöckner, A. Gruber, R. Hipkin, M. G. Janech, P. G. Kroth, F. Leese, E. A. Lindquist, B. R. Lyon, J. Martin, C. Mayer, M. Parker, H. Quesneville, J. A. Raymond, C. Uhlig, R. E. Valas, K. U. Valentin, A. Z. Worden, E. V. Armbrust, M. D. Clark, C. Bowler, B. R. Green, V. Moulton, C. van Oosterhout, I. V. Grigoriev, Evolutionary genomics of the cold-adapted diatom *Fragilariopsis cylindrus*. Nature 541, 536–540 (2017). 28092920 10.1038/nature20803

[17] G. W. Vurture, F. J. Sedlazeck, M. Nattestad, C. J. Underwood, H. Fang, J. Gurtowski, M. C. Schatz, GenomeScope: Fast reference-free genome profiling from short reads. Bioinformatics 33, 2202–2204 (2017). 28369201 10.1093/bioinformatics/btx153 PMC5870704

[18] C. S. Chin, P. Peluso, F. J. Sedlazeck, M. Nattestad, G. T. Concepcion, A. Clum, C. Dunn, R. O’Malley, R. Figueroa-Balderas, A. Morales-Cruz, G. R. Cramer, M. Delledonne, C. Luo, J. R. Ecker, D. Cantu, D. R. Rank, M. C. Schatz, Phased diploid genome assembly with single-molecule real-time sequencing. Nat. Methods 13, 1050–1054 (2016). 27749838 10.1038/nmeth.4035 PMC5503144

[19] B. J. Walker, T. Abeel, T. Shea, M. Priest, A. Abouelliel, S. Sakthikumar, C. A. Cuomo, Q. Zeng, J. Wortman, S. K. Young, A. M. Earl, Pilon: An integrated tool for comprehensive microbial variant detection and genome assembly improvement. PLOS ONE 9, e112963 (2014). 25409509 10.1371/journal.pone.0112963 PMC4237348

[20] K. J. Hoff, S. Lange, A. Lomsadze, M. Borodovsky, M. Stanke, BRAKER1: Unsupervised RNA-Seq-based genome annotation with GeneMark-ET and AUGUSTUS. Bioinformatics 32, 767–769 (2016). 26559507 10.1093/bioinformatics/btv661 PMC6078167

[21] R. M. Waterhouse, M. Seppey, F. A. Simão, M. Manni, P. Ioannidis, G. Klioutchnikov, E. V. Kriventseva, E. M. Zdobnov, BUSCO applications from quality assessments to gene prediction and phylogenomics. Mol. Biol. Evol. 35, 543–548 (2018). 29220515 10.1093/molbev/msx319 PMC5850278

[22] A. Gruber, G. Rocap, P. G. Kroth, E. V. Armbrust, E. V. T. Mock, Plastid proteome prediction for diatoms and other algae with secondary plastids of the red lineage. Plant J. 81, 519–528 (2015). 25438865 10.1111/tpj.12734 PMC4329603

[23] D. M. Emms, S. Kelly, OrthoFinder: Solving fundamental biases in whole genome comparisons dramatically improves orthogroup inference accuracy. Genome Biol. 16, 157 (2015). 26243257 10.1186/s13059-015-0721-2 PMC4531804

[24] R. Kamikawa, G. Tanifuji, S. A. Ishikawa, K. Ishii, Y. Matsuno, N. T. Onodera, K. Ishida, T. Hashimoto, H. Miyashita, S. Mayama, Y. Inagaki, Proposal of a twin arginine translocator system-mediated constraint against loss of ATP synthase genes from nonphotosynthetic plastid genomes. Mol. Biol. Evol. 32, 2598–2604 (2015). 26048548 10.1093/molbev/msv134

[25] D. Moog, A. Nozawa, Y. Tozawa, R. Kamikawa, Substrate specificity of plastid phosphate transporters in a non-photosynthetic diatom and its implication in evolution of red alga-derived complex plastids. Sci. Rep. 10, 1167 (2020). 31980711 10.1038/s41598-020-58082-8 PMC6981301

[26] R. Kamikawa, D. Moog, S. Zauner, G. Tanifuji, K. Ishida, H. Miyashita, H. Mayama, T. Hashimoto, U. G. Maier, J. M. Archibald, Y. Inagaki, A non-photosynthetic diatom reveals early steps of reductive evolution in plastids. Mol. Biol. Evol. 34, 2355–2366 (2017). 28549159 10.1093/molbev/msx172

[27] A. E. Allen, C. L. Dupont, M. Oborník, A. Horák, A. Nunes-Nesi, J. P. McCrow, H. Zheng, D. A. Johnson, H. Hu, A. R. Fernie, C. Bowler, Evolution and metabolic significance of the urea cycle in photosynthetic diatoms. Nature 473, 203–207 (2011). 21562560 10.1038/nature10074

[28] S. R. Smith, C. L. Dupont, J. K. McCarthy, J. T. Broddrick, M. Oborník, A. Horák, Z. Füssy, J. Cihlář, S. Kleessen, H. Zheng, J. P. McCrow, K. K. Hixson, W. L. Araújo, A. Nunes-Nesi, A. Fernie, Z. Nikoloski, B. O. Palsson, A. E. Allen, Evolution and regulation of nitrogen flux through compartmentalized metabolic networks in a marine diatom. Nat. Commun. 10, 4552 (2019). 31591397 10.1038/s41467-019-12407-y PMC6779911

[29] J. Ashworth, S. Coesel, A. Lee, E. V. Armbrust, M. V. Orellana, N. S. Baliga, Genome-wide diel growth state transitions in the diatom *Thalassiosira pseudonana*. Proc. Natl. Acad. Sci. U.S.A. 110, 7518–7523 (2013). 23596211 10.1073/pnas.1300962110 PMC3645528

[30] M. S. Chauton, P. Winge, T. Brembu, O. Vadstein, A. M. Bones, Gene regulation of carbon fixation, storage, and utilization in the diatom *Phaeodactylum tricornutum* acclimated to light/dark cycles. Plant Physiol. 161, 1034–1048 (2013). 23209127 10.1104/pp.112.206177 PMC3561001

[31] S. R. Smith, J. T. F. Gillard, A. B. Kustka, J. P. McCrow, J. H. Badger, H. Zheng, A. M. New, C. L. Dupont, T. Obata, A. R. Fernie, A. E. Allen, Correction: Transcriptional orchestration of the global cellular response of a model pennate diatom to diel light cycling under iron limitation. PLOS Genet. 13, e1006688 (2017). 28355217 10.1371/journal.pgen.1006688 PMC5371279

[32] M. J. J. Huysman, C. Martens, K. Vandepoele, J. Gillard, E. Rayko, M. Heijde, C. Bowler, D. Inzé, Y. Van de Peer, L. De Veylder, W. Vyverman, Genome-wide analysis of the diatom cell cycle unveils a novel type of cyclins involved in environmental signaling. Genome Biol. 11, R17 (2010). 20146805 10.1186/gb-2010-11-2-r17 PMC2872877

[33] R. Annunziata, A. Ritter, A. Emidio Fortunato, A. Manzotti, S. Cheminant-Navarro, N. Agier, M. J. J. Huysman, P. Winge, A. M. Bones, F. Bouget, M. Cosentino Lagomarsino, J. Bouly, A. Falciatore, bHLH-PAS protein RITMO1 regulates diel biological rhythms in the marine *diatom Phaeodactylum tricornutum*. Proc. Natl. Acad. Sci. U.S.A. 116, 13137–13142 (2019). 31171659 10.1073/pnas.1819660116 PMC6600994

[34] P. G. Kroth, C. Wilhelm, T. Kottke, An update on aureochromes: Phylogeny–mechanism–function. J. Plant Physiol. 217, 20–26 (2017). 28797596 10.1016/j.jplph.2017.06.010

[35] S. Coesel, M. Mangogna, T. Ishikawa, M. Heijde, A. Rogato, G. Finazzi, T. Todo, C. Bowler, A. Falciatore, Diatom PtCPF1 is a new cryptochrome/photolyase family member with DNA repair and transcription regulation activity. EMBO Rep. 10, 655–661 (2009). 19424294 10.1038/embor.2009.59 PMC2711838

[36] A. E. Fortunato, M. Jaubert, G. Enomoto, J. Bouly, R. Raniello, M. Thaler, M. Malviya, J. S. Bernardes, F. Rappaport, B. Gentili, M. J. J. Huysman, A. Carbone, C. Bowler, M. Ribera d’Alcalà, M. Ikeuchi, A. Falciatore, Diatom phytochromes reveal the existence of far-red-light-based sensing in the ocean. Plant Cell 28, 616–628 (2016). 26941092 10.1105/tpc.15.00928 PMC4826011

[37] E. Rayko, F. Maumus, U. Maheswari, K. Jabbari, C. Bowler, Transcription factor families inferred from genome sequences of photosynthetic stramenopiles. New Phytol. 188, 52–66 (2010). 20646219 10.1111/j.1469-8137.2010.03371.x

[38] G. Kong, Y. Chen, Y. Deng, D. Feng, L. Jiang, L. Wan, M. Li, Z. Jiang, P. Xi, The basic leucine zipper transcription factor PlBZP32 associated with the oxidative stress response is critical for pathogenicity of the lychee downy blight oomycete *Peronophythora litchi*. mSphere 5, e00261-20 (2020). 32493721 10.1128/mSphere.00261-20 PMC7273347

[39] T. Fujiwara, S. Hirooka, O. Ohbayashi, R. Onuma, S. Miyagishima, Relationship between cell cycle and diel transcriptomic changes in metabolism in a unicellular red alga. Plant Physiol. 183, 1484–1501 (2020). 32518202 10.1104/pp.20.00469 PMC7401142

[40] T. A. Richards, N. J. Talbot, Horizontal gene transfer in osmotrophs: Playing with public goods. Nat. Rev. Microbiol. 11, 720–727 (2013). 24018383 10.1038/nrmicro3108

[41] T. A. Richards, N. J. Talbot, Osmotrophy. Curr. Biol. 28, R1179–R1180 (2018). 30352181 10.1016/j.cub.2018.07.069

[42] K. Ishii, R. Kamikawa, Growth characterization of non-photosynthetic diatoms, *Nitzschia* spp., inhabiting estuarine mangrove forests of Ishigaki Island, Japan. Plankton Benthos Res. 12, 164–170 (2017).

[43] A. J. Enright, S. Van Dongen, C. A. Ouzounis, An efficient algorithm for large-scale detection of protein families. Nucleic Acids Res. 30, 1575–1584 (2002). 11917018 10.1093/nar/30.7.1575 PMC101833

[44] Y. Belkhadir, L. Yang, J. Hetzel, J. L. Dangl, J. Chory, The growth–defense pivot: Crisis management in plants mediated by LRR-RK surface receptors. Trends Biochem. Sci. 39, 447–456 (2014). 25089011 10.1016/j.tibs.2014.06.006 PMC4177940

[45] U. Karsten, A. S. Mostaert, R. J. King, M. Kamiya, Y. Hara, Osmoprotectors in some species of Japanese mangrove macroalgae. Phycol. Res. 44, 109–112 (1996).

[46] Y. I. Wolf, E. V. Koonin, Genome reduction as the dominant mode of evolution. Bioessays 35, 829–837 (2013). 23801028 10.1002/bies.201300037 PMC3840695

[47] M. Nei, S. Kumar S, *Molecular Evolution and Phylogenetics* (Oxford Univ. Press, New York, 2000), pp. 17–203

[48] J. J. Morris, R. E. Lenski, E. R. Zinser, The Black Queen hypothesis: Evolution of dependencies through adaptive gene loss. MBio 3, e00036-12 (2012). 22448042 10.1128/mBio.00036-12 PMC3315703

[49] E. A. Ottesen, C. R. Young, S. M. Gifford, J. M. Eppley, R. Marin III, S. C. Schuster, C. A. Scholin, E. F. DeLong, Multispecies diel transcriptional oscillations in open ocean heterotrophic bacterial assemblages. Science 345, 207–212 (2014). 25013074 10.1126/science.1252476

[50] F. O. Aylward, J. M. Eppley, J. M. Smith, F. P. Chavez, C. A. Scholin, E. F. DeLong, Microbial community transcriptional networks are conserved in three domains at ocean basin scales. Proc. Natl. Acad. Sci. U.S.A. 112, 5443–5448 (2015). 25775583 10.1073/pnas.1502883112 PMC4418921

[51] K. R. Frischkorn, S. T. Haley, S. T. Dyhrman, Coordinated gene expression between *Trichodesmium* and its microbiome over day–night cycles in the North Pacific Subtropical Gyre. ISME J. 12, 997–1007 (2018). 29382945 10.1038/s41396-017-0041-5 PMC5864210

[52] E. A. Ottesen, C. R. Young, J. M. Eppley, J. P. Ryan, F. P. Chavez, C. A. Scholin, E. F. DeLong, Pattern and synchrony of gene expression among sympatric marine microbial populations. Proc. Natl. Acad. Sci. U.S.A. 110, E488–E497 (2013). 23345438 10.1073/pnas.1222099110 PMC3568374

[53] M. D. Hernández Limón, G. M. M. Hennon, M. J. Harke, K. R. Frischkorn, S. T. Haley, S. T. Dyhrman, Transcriptional patterns of *Emiliania huxleyi* in the North Pacific Subtropical Gyre reveal the daily rhythms of its metabolic potential. Environ. Microbiol. 22, 381–396 (2020). 31709692 10.1111/1462-2920.14855

[54] A. M. Bolger, M. A. Lohse, B. Usadel, Trimmomatic: A flexible trimmer for Illumina sequence data. Bioinformatics 30, 2114–2120 (2014). 24695404 10.1093/bioinformatics/btu170 PMC4103590

[55] D. Kim, B. Langmead, S. L. Salzberg, HISAT: A fast spliced aligner with low memory requirements. Nat. Methods 12, 357–360 (2015). 25751142 10.1038/nmeth.3317 PMC4655817

[56] B. J. Haas, A. L. Delcher, S. M. Mount, J. R. Wortman, R. K. Smith Jr., L. I. Hannick, R. Maiti, C. M. Ronning, D. B. Rusch, C. D. Town, S. L. Salzberg, O. White, Improving the *Arabidopsis* genome annotation using maximal transcript alignment assemblies. Nucleic Acids Res. 31, 5654–5666 (2003). 14500829 10.1093/nar/gkg770 PMC206470

[57] M. G. Grabherr, B. J. Haas, M. Yassour, J. Z. Levin, D. A. Thompson, I. Amit, X. Adiconis, L. Fan, R. Raychowdhury, Q. Zeng, Z. Chen, E. Mauceli, N. Hacohen, A. Gnirke, N. Rhind, F. di Palma, B. W. Birren, C. Nusbaum, K. Lindblad-Toh, N. Friedman, A. Regev, Full-length transcriptome assembly from RNA-Seq data without a reference genome. Nat. Biotechnol. 29, 644–652 (2011). 21572440 10.1038/nbt.1883 PMC3571712

[58] B. J. Haas, A. Papanicolaou, M. Yassour, M. Grabherr, P. D. Blood, J. Bowden, M. Brian Couger, D. Eccles, B. Li, M. Lieber, M. D. MacManes, M. Ott, J. Orvis, N. Pochet, F. Strozzi, N. Weeks, R. Westerman, T. William, C. N. Dewey, R. Henschel, R. D. LeDuc, N. Friedman, A. Regev, De novo transcript sequence reconstruction from RNA-seq using the Trinity platform for reference generation and analysis. Nat. Protocol 8, 1494–1512 (2013). 10.1038/nprot.2013.084 PMC3875132 23845962

[59] C. Camacho, G. Coulouris, V. Avagyan, N. Ma, J. Papadopoulos, K. Bealer, T. L. Madden, BLAST^+^: Architecture and applications. BMC Bioinformatics 10, 421 (2009). 20003500 10.1186/1471-2105-10-421 PMC2803857

[60] S. El-Gebali, J. Mistry, A. Bateman, S. R. Eddy, A. Luciani, S. C. Potter, M. Qureshi, L. J. Richardson, G. A. Salazar, A. Smart, E. L. L. Sonnhammer, L. Hirsh, L. Paladin, D. Piovesan, S. C. E. Tosatto, R. D. Finn, The Pfam protein families database in 2019. Nucleic Acids Res. 47, D427–D432 (2019). 30357350 10.1093/nar/gky995 PMC6324024

[61] M. Tarailo-Graovac, N. Chen, Using RepeatMasker to identify repetitive elements in genomic sequences. Curr. Protoc. Bioinform. 25, 4.10.1–4.10.14 (2009). 10.1002/0471250953.bi0410s25 19274634

[62] G. Pertea, M. Pertea, GFF utilities: GffRead and GffCompare. F1000Research 9, 304 (2020). 10.12688/f1000research.23297.1 PMC7222033 32489650

[63] P. Jones, D. Binns, H.-Y. Chang, M. Fraser, W. Li, C. McAnulla, H. McWilliam, J. Maslen, A. Mitchell, G. Nuka, S. Pesseat, A. F. Quinn, A. Sangrador-Vegas, M. Scheremetjew, S.-Y. Yong, R. Lopez, S. Hunter, InterProScan 5: Genome-scale protein function classification. Bioinformatics 30, 1236–1240 (2014). 24451626 10.1093/bioinformatics/btu031 PMC3998142

[64] A. Marchler-Bauer, S. H. Bryant, CD-Search: Protein domain annotations on the fly. Nucleic Acids Res. 32, W327–W331 (2004). 15215404 10.1093/nar/gkh454 PMC441592

[65] Y. Moriya, M. Itoh, S. Okuda, A. C. Yoshizawa, M. Kanehisa, KAAS: An automatic genome annotation and pathway reconstruction server. Nucleic Acids Res. 35, W182–W185 (2007). 17526522 10.1093/nar/gkm321 PMC1933193

[66] H. Li, V. A. Benedito, M. K. Udvardi, P. X. Zhao, TransportTP: A two-phase classification approach for membrane transporter prediction and characterization. BMC Bioinformatics 10, 418 (2009). 20003433 10.1186/1471-2105-10-418 PMC3087344

[67] V. Lombard, H. Golaconda Ramulu, E. Drula, P. M. Coutinho, B. Henrissat, The carbohydrate-active enzymes database (CAZy) in 2013. Nucleic Acids Res. 42, D490–D495 (2014). 24270786 10.1093/nar/gkt1178 PMC3965031

[68] S. F. Altschul, T. L. Madden, A. A. Schäffer, J. Zhang, Z. Zhang, W. Miller, D. J. Lipman, Gapped BLAST and PSI-BLAST: A new generation of protein database search programs. Nucleic Acids Res. 25, 3389–3402 (1997). 9254694 10.1093/nar/25.17.3389 PMC146917

[69] J. Mistry, R. D. Finn, S. R. Eddy, A. Bateman, M. Punta, Challenges in homology search: HMMER3 and convergent evolution of coiled-coil regions. Nucleic Acids Res. 41, e121 (2013). 23598997 10.1093/nar/gkt263 PMC3695513

[70] B. A. Curtis, G. Tanifuji, F. Burki, A. Gruber, M. Irimia, S. Maruyama, M. C. Arias, S. G. Ball, G. H. Gile, Y. Hirakawa, J. F. Hopkins, A. Kuo, S. A. Rensing, J. Schmutz, A. Symeonidi, M. Elias, R. J. M. Eveleigh, E. K. Herman, M. J. Klute, T. Nakayama, M. Oborník, A. Reyes-Prieto, E. V. Armbrust, S. J. Aves, R. G. Beiko, P. Coutinho, J. B. Dacks, D. G. Durnford, N. M. Fast, B. R. Green, C. J. Grisdale, F. Hempel, B. Henrissat, M. P. Höppner, K.-I. Ishida, E. Kim, L. Kořený, P. G. Kroth, Y. Liu, S.-B. Malik, U. G. Maier, D. McRose, T. Mock, J. A. D. Neilson, N. T. Onodera, A. M. Poole, E. J. Pritham, T. A. Richards, G. Rocap, S. W. Roy, C. Sarai, S. Schaack, S. Shirato, C. H. Slamovits, D. F. Spencer, S. Suzuki, A. Z. Worden, S. Zauner, K. Barry, C. Bell, A. K. Bharti, J. A. Crow, J. Grimwood, R. Kramer, E. Lindquist, S. Lucas, A. Salamov, G. I. McFadden, C. E. Lane, P. J. Keeling, M. W. Gray, I. V. Grigoriev, J. M. Archibald, Algal genomes reveal evolutionary mosaicism and the fate of nucleomorphs. Nature 492, 59–65 (2012). 23201678 10.1038/nature11681

[71] U. Cenci, S. J. Sibbald, B. A. Curtis, R. Kamikawa, L. Eme, D. Moog, B. Henrissat, E. Maréchal, M. Chabi, C. Djemiel, A. J. Roger, E. Kim, J. M. Archibald, Nuclear genome sequence of the plastid-lacking cryptomonad *Goniomonas avonlea* provides insights into the evolution of secondary plastids. BMC Biol. 16, 137 (2018). 30482201 10.1186/s12915-018-0593-5 PMC6260743

[72] J. R. Bray, J. T. Curtis, An ordination of the upland forest communities of Southern Wisconsin. Ecol. Monogr. 27, 325–349 (1957).

[73] J. H. Ward Jr., Hierarchical grouping to optimize an objective function. J. Am. Stat. Assoc. 58, 236–244 (1963).

[74] J. Oksanen, Multivariate Analysis of Ecological Communities in R: Vegan tutorial (2015); https://www.mooreecology.com/uploads/2/4/2/1/24213970/vegantutor.pdf.

[75] K. Katoh, D. M. Standley, MAFFT multiple sequence alignment software version 7: Improvements in performance and usability. Mol. Biol. Evol. 30, 772–780 (2013). 23329690 10.1093/molbev/mst010 PMC3603318

[76] T. A. Hall, BioEdit: A user-friendly biological sequence alignment editor and analysis program for windows 95/98/NT. Nucleic Acids Symp. Ser. 41, 95–98 (1999).

[77] L. T. Nguyen, H. A. Schmidt, A. Von Haeseler, B. Q. Minh, IQ-TREE: A fast and effective stochastic algorithm for estimating maximum-likelihood phylogenies. Mol. Biol. Evol. 32, 268–274 (2015). 25371430 10.1093/molbev/msu300 PMC4271533

[78] A. Davis, R. Abbriano, S. R. Smith, M. Hildebrand, Clarification of photorespiratory processes and the role of malic enzyme in diatoms. Protist 168, 134–153 (2017). 28104538 10.1016/j.protis.2016.10.005

[79] N. H. Gonzalez, G. Felsner, F. D. Schramm, A. Klingl, U. G. Maier, K. Bolte, A single peroxisomal targeting signal mediates matrix protein import in diatoms. PLOS ONE 6, e25316 (2011). 21966495 10.1371/journal.pone.0025316 PMC3178647

[80] A. K. Mix, U. Cenci, T. Heimerl, P. Marter, M. L. Wirkner, D. Moog, Identification and localization of peroxisomal biogenesis proteins indicates the presence of peroxisomes in the cryptophyte *Guillardia theta* and other “Chromalveolates”. Genome Biol. Evol. 10, 2834–2852 (2018). 30247558 10.1093/gbe/evy214 PMC6203080

[81] K. Sidiropoulos, G. Viteri, C. Sevilla, S. Jupe, M. Webber, M. Orlic-Milacic, B. Jassal, B. May, V. Shamovsky, C. Duenas, K. Rothfels, L. Matthews, H. Song, L. Stein, R. Haw, P. D’Eustachio, P. Ping, H. Hermjakob, A. Fabregat, Reactome enhanced pathway visualization. Bioinformatics 33, 3461–3467 (2017). 29077811 10.1093/bioinformatics/btx441 PMC5860170

[82] A. Fabregat, F. Korninger, G. Viteri, K. Sidiropoulos, P. Marin-Garcia, P. Ping, G. Wu, L. Stein, P. D’Eustachio, H. Hermjakob, Reactome graph database: Efficient access to complex pathway data. PLOS Comput. Biol. 14, e1005968 (2018). 29377902 10.1371/journal.pcbi.1005968 PMC5805351

[83] A. Fabregat, K. Sidiropoulos, G. Viteri, P. Marin-Garcia, P. Ping, L. Stein, P. D’Eustachio, H. Hermjakob, Reactome diagram viewer: Data structures and strategies to boost performance. Bioinformatics 34, 1208–1214 (2018). 29186351 10.1093/bioinformatics/btx752 PMC6030826

[84] B. Jassal, L. Matthews, G. Viteri, C. Gong, P. Lorente, A. Fabregat, K. Sidiropoulos, J. Cook, M. Gillespie, R. Haw, F. Loney, B. May, M. Milacic, K. Rothfels, C. Sevilla, V. Shamovsky, S. Shorser, T. Varusai, J. Weiser, G. Wu, L. Stein, H. Hermjakob, P. D’Eustachio, The reactome pathway knowledgebase. Nucleic Acids Res. 48, D498–D503 (2020). 31691815 10.1093/nar/gkz1031 PMC7145712

[85] UniProt Consortium, UniProt: A worldwide hub of protein knowledge. Nucleic Acids Res. 47, D506–D515 (2019). 30395287 10.1093/nar/gky1049 PMC6323992

[86] S. F. Altschul, W. Gish, W. Miller, E. W. Myers, D. J. Lipman, Basic local alignment search tool. J. Mol. Biol. 215, 403–410 (1990). 2231712 10.1016/S0022-2836(05)80360-2

[87] T. Aramaki, R. Blanc-Mathieu, H. Endo, K. Ohkubo, M. Kanehisa, S. Goto, H. Ogata, KofamKOALA: KEGG Ortholog assignment based on profile HMM and adaptive score threshold. Bioinformatics 36, 2251–2252 (2020). 31742321 10.1093/bioinformatics/btz859 PMC7141845

[88] Y. Fukasawa, J. Tsuji, S. C. Fu, K. Tomii, P. Horton, K. Imai, MitoFates: Improved prediction of mitochondrial targeting sequences and their cleavage sites. Mol. Cell. Proteomics 14, 1113–1126 (2015). 25670805 10.1074/mcp.M114.043083 PMC4390256

[89] K. Kume, T. Amagasa, T. Hashimoto, H. Kitagawa, NommPred: Prediction of mitochondrial and mitochondrion-related organelle proteins of nonmodel organisms. Evol. Bioinform. Online 14, 1176934318819835 (2018). 30626996 10.1177/1176934318819835 PMC6305954

[90] T. N. Petersen, S. Brunak, G. von Heijne, H. Nielsen, SignalP 4.0: Discriminating signal peptides from transmembrane regions. Nat. Methods 8, 785–786 (2011). 21959131 10.1038/nmeth.1701

[91] M. Kanehisa, S. Goto, Y. Sato, M. Furumichi, M. Tanabe, KEGG for integration and interpretation of large-scale molecular data sets. Nucleic Acids Res. 40, D109–D114 (2012). 22080510 10.1093/nar/gkr988 PMC3245020

[92] E. L. Sonnhammer, G. von Heijne, A. Krogh, A hidden Markov model for predicting transmembrane helices in protein sequences. Proc. Int. Conf. Intell. Syst. Mol. Biol. 6, 175–182 (1998). 9783223

[93] A. Krogh, B. Larsson, G. von Heijne, E. L. Sonnhammer, Predicting transmembrane protein topology with a hidden markov model: Application to complete genomes. J. Mol. Biol. 305, 567–580 (2001). 11152613 10.1006/jmbi.2000.4315

[94] C. G. Bruckner, C. Rehm, H. P. Grossart, P. G. Kroth, Growth and release of extracellular organic compounds by benthic diatoms depend on interactions with bacteria. Environ. Microbiol. 13, 1052–1063 (2011). 21244599 10.1111/j.1462-2920.2010.02411.x

[95] M. T. Buhmann, B. Schulze, A. Förderer, D. Schleheck, P. G. Kroth, Bacteria may induce the scretion of mucin-like proteins by the diatom *Phaeodactylum tricornutum*. J. Phycol. 52, 463–474 (2016). 26993172 10.1111/jpy.12409

[96] M. Lachnit, M. T. Buhmann, J. Klemm, N. Kröger, N. Poulsen, Identification of proteins in the adhesive trails of the diatom *Amphora coffeaeformis*. Philos. Trans. R. Soc. B 374, 20190196 (2019). 10.1098/rstb.2019.0196 PMC6745473 31495312

[97] G. Dell’Aquila, S. Zauner, T. Heimerl, J. Kahnt, V. Samel-Gondesen, S. Runge, F. Hempel, U. G. Maier, Mobilization and cellular distribution of phosphate in the diatom *Phaeodactylum tricornutum*. Front. Plant Sci. 11, 579 (2020). 32582227 10.3389/fpls.2020.00579 PMC7283521

[98] E. G. Bligh, W. J. Dyer, A rapid method of total lipid extraction and purification. Can. J. Biochem. Physiol. 37, 911–917 (1959). 13671378 10.1139/o59-099

[99] E. Mitani, F. Nakayama, I. Matsuwaki, I. Ichi, A. Kawabata, M. Kawachi, M. Kato, Fatty acid composition profiles of 235 strains of three microalgal divisions within the NIES microbial culture collection. Microb. Resour. Syst. 33, 19–29 (2017).

[100] R. S. Wright, A reagent for the non-destructive location of steroids and some other lipophilic materials on silica gel thin-layer chromatograms. J. Chromatogr. 59, 220–221 (1971). 4329284 10.1016/s0021-9673(01)80033-9

[101] N. Sato, Lipids in *Cryptomonas* CR-1. I. Occurrence of betaine lipids. Plant Cell. Physiol. 32, 819–825 (1991).

[102] C. F. Allen, P. Good, [48] Acyl lipids in photosynthetic systems. Methods Enzymol. 23, 523–547 (1971).

[103] L. K. Johnson, H. Alexander, C. T. Brown, Re-assembly, quality evaluation, and annotation of 678 microbial eukaryotic reference transcriptomes. GigaScience 8, giy158 (2019). 30544207 10.1093/gigascience/giy158 PMC6481552

[104] A. Criscuolo, S. Gribaldo, BMGE (Block Mapping and Gathering with Entropy): A new software for selection of phylogenetic informative regions from multiple sequence alignments. BMC Evol. Biol. 10, 210 (2010). 20626897 10.1186/1471-2148-10-210 PMC3017758

[105] B. Langmead, S. L. Salzberg, Fast gapped-read alignment with Bowtie 2. Nat. Methods 9, 357–359 (2012). 22388286 10.1038/nmeth.1923 PMC3322381

[106] H. Li, B. Handsaker, A. Wysoker, T. Fennell, J. Ruan, N. Homer, G. Marth, G. Abecasis, R. Durbin; 1000 Genome Project Data Processing Subgroup, The sequence alignment/map format and SAMtools. Bioinformatics 25, 2078–2079 (2009). 19505943 10.1093/bioinformatics/btp352 PMC2723002

[107] A. R. Quinlan, I. M. Hall, BEDTools: A flexible suite of utilities for comparing genomic features. Bioinformatics 26, 841–842 (2010). 20110278 10.1093/bioinformatics/btq033 PMC2832824

[108] R. Ihaka, R. Gentleman, R: A language for data analysis and graphics. J. Comp. Graph. Stat. 5, 299–314 (1996).

[109] M. J. L. de Hoon, S. Imoto, J. Nolan, S. Miyano, Open source clustering software. Bioinformatics 20, 1453–1454 (2004). 14871861 10.1093/bioinformatics/bth078

[110] A. J. Saldanha, Java Treeview - Extensible visualization of microarray data. Bioinformatics 20, 3246–3248 (2004). 15180930 10.1093/bioinformatics/bth349

[111] A. Löytynoja, Phylogeny-aware alignment with PRANK. Methods Mol. Biol. 1079, 155–170 (2014). 24170401 10.1007/978-1-62703-646-7_10

[112] S. Capella-Gutiérrez, J. M. Silla-Martínez, T. Gabaldón, trimAl: A tool for automated alignment trimming in large-scale phylogenetic analyses. Bioinformatics 25, 1972–1973 (2009). 19505945 10.1093/bioinformatics/btp348 PMC2712344

[113] G. Talavera, J. Castresana, Improvement of phylogenies after removing divergent and ambiguously aligned blocks from protein sequence alignments. Syst. Biol. 56, 564–577 (2007). 17654362 10.1080/10635150701472164

[114] R. Bouckaert, T. G. Vaughan, J. Barido-Sottani, S. Duchêne, M. Fourment, A. Gavryushkina, J. Heled, G. Jones, D. Kühnert, N. De Maio, M. Matschiner, BEAST 2.5: An advanced software platform for Bayesian evolutionary analysis. PLOS Comput. Biol. 15, e1006650 (2019). 30958812 10.1371/journal.pcbi.1006650 PMC6472827

[115] S. Höhna, M. R. May, B. R. Moore, TESS: An R package for efficiently simulating phylogenetic trees and performing Bayesian inference of lineage diversification rates. Bioinformatics 32, 789–791 (2016). 26543171 10.1093/bioinformatics/btv651

[116] Z. Yang, W. S. W. Wong, R. Nielsen, Bayes empirical bayes inference of amino acid sites under positive selection. Mol. Biol. Evol. 22, 1107–1118 (2005). 15689528 10.1093/molbev/msi097

[117] Z. Yang, PAML 4: Phylogenetic analysis by maximum likelihood. Mol. Biol. Evol. 24, 1586–1591 (2007). 17483113 10.1093/molbev/msm088

[118] J.-F. Pombert, N. A. Blouin, C. Lane, D. Boucias, P. J. Keeling, A lack of parasitic reduction in the obligate parasitic green alga *Helicosporidium*. PLOS Genet. 10, e1004355 (2014). 24809511 10.1371/journal.pgen.1004355 PMC4014436

[119] S. Suzuki, R. Endoh, R. Manabe, M. Ohkuma, Y. Hirakawa, Multiple losses of photosynthesis and convergent reductive genome evolution in the colourless green algae *Prototheca*. Sci. Rep. 8, 940 (2018). 29343788 10.1038/s41598-017-18378-8 PMC5772498

[120] C. A. Durkin, J. A. Koester, S. J. Bender, E. V. Armbrust, The evolution of silicon transporters in diatoms. J. Phycol. 52, 716–731 (2016). 27335204 10.1111/jpy.12441 PMC5129515

