# Supplementary material for: Genome evolution of a nonparasitic secondary heterotroph, the diatom Nitzschia putrida
Source: Sci Adv. 2022 Apr 29;8(17):eabi5075. doi: 10.1126/sciadv.abi5075 (PMC9054022; doi:10.1126/sciadv.abi5075)
Supplement: Supplementary file 1 — Supplementary Text Figs. S1 to S15 Tables S1 to S3 References [file sciadv.abi5075_sm.pdf]

Science Advances
AAAS

Supplementary Materials for

Genome evolution of a nonparasitic secondary heterotroph, the diatom *Nitzschia putrida*

Ryoma Kamikawa*, Takako Mochizuki, Mika Sakamoto, Yasuhiro Tanizawa, Takuro Nakayama, Ryo Onuma, Ugo Cenci, Daniel Moog, Samuel Speak, Krisztina Sarkozi, Andrew Toseland, Cock van Oosterhout, Kaori Oyama, Misako Kato, Keitaro Kume, Motoki Kayama, Tomonori Azuma, Ken-ichiro Ishii, Hideaki Miyashita, Bernard Henrissat, Vincent Lombard, Joe Win, Sophien Kamoun, Yuichiro Kashiyama, Shigeki Mayama, Shin-ya Miyagishima, Goro Tanifuji, Thomas Mock*, Yasukazu Nakamura

*Corresponding author. Email: kamikawa.ryoma.7v@kyoto-u.ac.jp (R.K.); t.mock@uea.ac.uk (T.M.)

Published 29 April 2022, *Sci. Adv.* **8**, eabi5075 (2022)
DOI: 10.1126/sciadv.abi5075

This PDF file includes:

Supplementary Text
Figs. S1 to S15
Tables S1 to S3
References

Supplementary Text

CAZyme annotation

We performed a manual annotation of CAZymes (*67*) using a combination of BLAST (*68*) and HMM searches (*69*), similar to that done previously (*70,71*). To assess the similarity between the CAZyme family profiles of the two species, we generated heat maps derived from an average linkage hierarchical clustering based on Bray-Curtis dissimilarity matrix distances and Ward's method (*72-73*). The heat maps were computed with Rstudio software (https://www.rstudio.com/) using vegan in the R package (https://github.com/vegandevs/vegan) (*74*) with vegdist and hclust commands.

Annotation of Cyclins, Cyclin-dependent kinases, bZIP transcription factors, and photoreceptor proteins

Cyclins, cyclin-dependent kinases, transcription factors were retrieved from the results of Pfam annotation. In addition, those proteins were surveyed by homology-based search with homologues of *P. tricornutum* and *T. pseudonana* (*32,37*) as queries. We also specifically surveyed photoreceptor proteins with diel cycle-based expression, according to the list of the previous study (*33*). Detected sequences were again confirmed as proteins of our interest by reciprocal blastP search against non-redundant database of GenBank. For Cyclins, CDKs, and bZIP transcription factors in *N. putrida*, *P. tricornutum*, and *T. pseudonana* were subjected to MAFFT (version 7.394; *75*), followed by removal of ambiguously aligned sites by Bioedit (*76*). The resultant datasets were subjected to the maximum likelihood analysis with IQ-TREE (*77*) under the LG+Γ+F model with 100 non-parametric bootstrap analyses.

Annotation of peroxisomal proteins

To identify potential peroxisomal proteins, the protein models of the diatom were screened via KEGG annotation and BLAST analyses. First, annotation of all the protein sequences was performed via GhostKOALA (KEGG Automatic Annotation Server; https://www.kegg.jp/ghostkoala/). Peroxisomal protein candidates were subsequently identified using the "KEGG Mapper – Reconstruct Pathway" tool (https://www.genome.jp/kegg/tool/map_pathway.html). In addition, all the *Nitzschia* proteins

were screened for the presence of a peroxisomal targeting signal of type 1 (PTS1, a C-terminal tri-peptide) using a local command line script identifying those entries, which contain the amino acids [SAC][KRHS][LM] within the last three positions of a protein sequence. Detected proteins were then functionally annotated with WebMGA (http://weizhong-lab.ucsd.edu/webMGA/server/kog/) as well as BlastKOALA (https://www.kegg.jp/blastkoala/) and their sequences were further investigated for the presence of additional targeting signals using SignalP 3.0 (http://www.cbs.dtu.dk/services/SignalP-3.0/) and 4.1 (http://www.cbs.dtu.dk/services/SignalP-4.1/), TargetP 1.1 (http://www.cbs.dtu.dk/services/TargetP-1.1/index.php), PredSL (http://aias.biol.uoa.gr/PredSL/), Predotar (https://urgi.versailles.inra.fr/predotar/) and TMHMM 2.0 (http://www.cbs.dtu.dk/services/TMHMM/). To identify factors for peroxisomal biogenesis/maintenance (peroxins) as well as for photorespiration and the glyoxylate cycle, manual BLAST analyses against the *Nitzschia* proteins were conducted using protein queries from the diatoms *Phaeodactylum tricornutum* and *Thalassiosira pseudonana,* the cryptophyte *Guillardia theta* (*78-80*) as well as yeast (peroxin identification) and an e-value cut-off of e-4. For verification, identified candidates were analyzed via reciprocal BlastP against the NCBI nr database (https://blast.ncbi.nlm.nih.gov) and using NCBI Conserved Domain Search (https://www.ncbi.nlm.nih.gov/Structure/cdd/wrpsb.cgi) for identification of conserved domains within the protein sequences. Missing proteins present in peroxisomes of many organisms were especially surveyed in the transcriptome data.

Annotation of mitochondrial proteins

To identify mitochondrial proteins, we retrieved mitochondrial reference pathways and proteins of *Homo sapiens*: from a stand-alone reactome server (*81-84*) on our systems, Uniprot (*85*) ids of proteins which are annotated that its subcellar locations are ‘mitochondrial matrix’, ‘mitochondrial inner membrane’, ‘mitochondrial outer membrane’ or ‘mitochondrial intermembrane space’ were extracted using the cypher query language. To search and annotate for mitochondrial proteins, we performed PSI-BLAST (*59,68,86*) search with proteins which have above uniprot ids as queries for the assemblies of genome and transcriptome. As validation of those annotations, we performed three analyses: 1) psi-blast search with above blast-hit sequences for swissprot (*85*), 2) KEGG orthology (KO) number assignment by kofamscan (*87*),

3) mitochondrial import signal analysis by Mitofates (*88*) and Nommpred (*89*). We manually refined our annotations based on these results. Missing proteins present in mitochondria of many organisms and/or in each metabolic pathway were especially surveyed in the transcriptome data (see above).

Annotation of plastid proteins

To identify plastid proteins, protein sequences were subjected to SignalP4.0 (*90*) followed by ASAFind (*22*). Functions and related metabolisms for proteins predicted to be localized in the plastid were estimated by their KEGG ID (see above) and KEGG mapper (*91*). Missing proteins in each metablic pathway were especially surveyed in the transcriptome data. Plastid proteins in the photosynthetic diatoms *Phaeodactylum tricornutum* and *Thalassiosira pseudonana* have been already published (*22*). We retrieved them and clustered with the plastid proteins of *N. putrida* as orthogroups by Orthofinder with default settings (*23*).

Secretome analysis

For the four diatom proteome datasets, we detected protein sequences with N-terminal signal peptides and no internal transmembrane domain by evaluation with signalP4.0 (*90*) and HMTMM (*92,93*), respectively. Those sequences highly likely comprise secretome and plastid sequences. The sequences were clustered by TribeMCL (*45*) into Tribes, with homology under e-30 criterion. If a tribe comprises sequences predicted to be localized in plastids with the "low confidence" category or non-plastidal by ASAFind (*22*), more than or equal to those predicted as plastid-localized with the "high confidence" category, we removed the tribe. Functional categorization was performed with domain annotation by Pfam, and we also removed tribes if the included sequences were predicted to have a domain for apparent organellar proteins such as plastid protein translocons, plastid heme biosynthesis, components of photosystems, organellar transporters, and lysosomal proteins by KO definition and/or Pfam. To evaluate whether the above procedure to detect secreted proteins is appropriate for diatoms, we made a benchmark dataset of diatoms including 21 secreted proteins experimentally confirmed (*94-97*) and 62 of their homologues, 182 plastid proteins, 29 mitochondrial proteins, and 29 proteins localized in other compartments such as cytosol and nucleus. From the benchmark set, our method identified 77 secreted proteins as secretome proteins, indicating 92.8% recovery rate. But non-secreted

proteins were not identified as secretome proteins in our benchmark set, suggesting that our secretome dataset for whole protein data in the four diatoms less likely comprise high proportion of non-secreted proteins. In addition, the benchmark dataset included 17 secreted protein sequences, experimentally verified, of *Phaeodactyum*, and of them, 13 protein sequences were retrieved by our genome-wide secretome analysis. The four proteins not identified as secreted proteins were appeared to lack signal peptides detectable either by SignalP4.0 and SignalP3.0, indicating those sequences might be secreted by signal peptide-independent ways.

Difference in the distributions of the number of protein sequences in each secretome tribe among species was tested by the Wilcoxon signed rank test. *p*-values for three pairs of comparisons that include *N. putrida* as a counterpart were adjusted by the Benjamini–Hochberg procedure for multiple-testing correction. The Wilcoxon signed rank test and the Benjamini–Hochberg procedure were conducted by using 'wilcoxon' function implemented in SciPy library (version 1.4.1) and 'multipletests' function implemented in Statsmodels library (version 0.11.1) for Python, respectively.

For Leucin-Rich Repeat (LRR) proteins, additional domains were also searched by NCBI Conserved Domain Search. All the possible secreted LRR proteins in *N. putrida*, *P. tricornutum*, and *T. pseudonana* were subjected to MAFFT (version 7.394; *75*) and ambiguously aligned sites were removed by Bioedit (*76*). The resultant dataset was subjected to IQ-TREE (*77*) under the LG+Γ+F model with 100 non-parametric bootstrap analyses.

For secretomes of parasitic green algae, we detected protein sequences with N-terminal signal peptides and no internal transmembrane domain by evaluation with signalP4.0 (*90*) and HMTMM (*92,93*), respectively. Those sequences highly likely comprise secretome proteins. Functional categorization was performed with domain annotation by Pfam. Clustering and comparison among parasitic species and their photosynthetic relatives were performed as described above for diatoms.

Analysis of lipids, fatty acids, and quinones/quinols

Crude lipids were extracted from the cells by the method of Bligh and Dyer (*98*). The lipid fraction was evaporated, and then the residue was heated at 90°C for 2 h with 2 mL of 5% (w/w) HCI-methanol to obtain fatty acid methyl esters. The methanol solution was extracted with 2 mL of n-hexane twice. The layer of n-hexane was concentrated to a minimum volume for use in gas-

chromatographic analysis. Gas chromatography was performed, according to (*99*), with a fused-silica capillary column (0.25 mm internal diameter x 50 m; ChrompackCp-Sil 88, Agilent Technologies Inc., USA) with the oven temperature was increased from 150 to 210˚C at 5°C min^{-1}. The fatty acid composition was calculated by a Chromatopac C-R8A data processor (Shimadzu Corp., Kyoto, Japan). Each fatty acid was identified by comparing the retention time with those of known standards. Thin layer chromatograph (silica gel 60) of the crude lipids developed by hexane/diethyl ether/acetate (80:20:1 v/v/v). Lipids were visualized under UV light at 365 nm after spraying the plate with 0.01% (w/v) primulin in 80% (v/v) acetone (*100*). Two-dimensional thin layer chromatograph of the polar lipids was performed according to Sato (*101*) with the following solvent systems: first dimension,acetone/benzene/methanol/water (8:3:2:1 v/v/v/v); second dimension, chloroform/methanol/28% ammonium (13:7:1 v/v/v). Each spot of polar lipids was detected as described above. Each lipid was identified by spraying the plate with a reagent specific to each lipid class (*102*). Each spot on a plate was scraped off, and then subjected to gas-chromatographic analysis and fatty acid identification as described above.

Quinone/quinol extraction and detection were performed as described in (*7*). In this analysis, we treated the quinone/quinol extract with ferric chloride (final concentration, 1.2 mM) before the analysis for oxidation of total quinones and quinols, to convert them to quinone forms for higher detection sensitivity according to (*7*). We could identify only ubiquinone but no plastoquinone from the cells of *N. putrida* NIES-4239 cultivated as described above for genome sequencing, supporting lack of the complete pathway of plastoquinol synthesis (Fig. 1).

Horizontal gene transfers

Total *N. putrida* protein sequences were subjected to Orthofinder with default settings (*23*) together with whole protein sets of the photosynthetic diatom genomes *P. tricornutum*, *F. cylindrus*, and *T. pseudonana*, resulting in 4,920 proteins unique to *N. putrida*. The 4,920 proteins were subjected to similarity search using BLASTP (BLAST+ 2.6.0) against GenBank non-redundant protein sequence database and protein sequence database provided by the Marine Microbial Eukaryote Transcriptome Sequencing Project (https://github.com/dib-lab/dib-MMETSP) (*103*) with an e-value threshold of 1.0E-15. BLASTP hits from two databases were combined and sorted by their bit-scores. Top ten hits with higher bit-scores for each query sequence were then inspected their taxonomic composition with two criteria: 1) if those hits do

not include any diatom sequences, namely, sequences from diatom species or dinoflagellate species bearing diatom-derived plastids, 2) if the ten hits do not include the diatom sequences except sequences from diatoms belonging to genus *Nitzschia*. Query sequences, whose top ten BLASTP hits match the two criteria, were considered as initial candidates for 1) genes derived from *N. putrida* specific horizontal gene transfer (HGT) event and 2) *Nitzchia*-lineage-specific HGT, respectively. Each of candidates with HGT origin was aligned together with all of the hit sequences in aforementioned BLASTP analyses by MAFFT (version 7.394) (*75*). After ambiguously aligned positions were removed by BMGE (version 1.12) (*104*), each single-gene alignment was subjected to a maximum-likelihood (ML) phylogenetic analysis by IQ-TREE (*77*) under LG+Γ+F model. The statistical supports for the bipartitions in ML trees were assessed by a nonparametric ML bootstrap analysis (100 replicates) under the same substitution model used for the ML trees. All topologies of ML trees for both of *N. putrida*-specific HGT and *Nitzchia*-lineage-specific HGT candidates were manually assessed.

Transcriptome analyses

The cells of *N. putrida* NIES-4239 cultivated under the 12 hours light and 12 hours dark conditions under the same condition as described above were then cultivated under the dark conditions for 48 hours. The cells possibly acclimated to the dark condition were transferred to the fresh liquid medium and incubated under the 12 hours light and 12 hours dark condition for 25 hours in which the last one hour was of the second light term. RNA was extracted every 4 hours at the one hour, 5 hours, and 9 hours after initiation of the first light condition, one hour, 5 hours, and 9 hours after initiation of the dark condition, and one hour after initiation of the second light term. The cultivation and RNA extraction were performed twice, samples which were called Replicates A and B. The extracted total RNAs were subjected to 151 bp paired-end sequencing by NextSeq 500 according to the manufacturer’s instruction in Bioengineering Lab, Japan, resulting in 9.8-13 million paired-end reads for each sample. Adapter trimming and quality filtering were performed with fastX toolkit (http://hannonlab.cshl.edu/fastx_toolkit/). In quality filtering, reads with quality scores > 20 for at least 75% of their length were retained, resulting in 7.3-11.1 million paired-end reads. To obtain gene expression scores, one side of the paired-end reads was mapped to the reference by Bowtie2 ver. 2.3.4.1 (*105*). SAMtools ver. 1.8 (*106*), BEDtools ver. 2.19.1 (*107*), and R ver. 3.5.3 (*108*) were used to calculate the reads per

kilobase of exon per million mapped reads (RPKM). To extract the reproducible change in transcriptome of Replicate A and B, the genes with low value of Pearson's correlation coefficient between two biological replicates (≦ 0.9) were omitted, resulting in 1,971 genes. To investigate expression patterns of 1,971 genes, the values of RPKM + 1 of each gene were transformed to log2 and centered by median values, and *k*-means clustering (*k* = 8) was performed by using Cluster 3.0 (*109*) based on Spearman correlation and complete linkage. The clustered expression patterns were visualized by Java TreeView (*110*) and R. Raw reads used for the transcriptome analyses were deposited in DNA Data Bank of Japan under the accession number PRJDB11016.

To investigate transcriptional regulation of CAZyme genes in different carbon sources, we performed comparative transcriptome analyses. *N. putrida* cells were cultivated as described above. Cells were then incubated in the carbon source-depleted medium, the IMK medium for three days. Those cells were transferred to the following three different media: the IMK medium, the glucose-rich medium that is the IMK medium including 0.2% glucose (w/v), and the starch-rich medium that is the IMK medium including 0.2% starch (w/v). After incubation for 3 days in the media, cells were subjected to RNA extraction as described above. Those procedures were performed in duplicate. RNA was subjected to to 101 bp paired-end sequencing by DNBSEQ G 400 RS High throughput Sequencing Kit (MGI Tech Co Ltd) according to the manufacturer's instructions in Bioengineering Lab, Japan, resulting in 13-17 million paired-end reads for each sample. Obtained reads were subjected to adapter trimming and quality filtering as described above, resulting in 13.3 – 16.7 million paired-end reads. The left reads were then used for mapping onto the gene model with Bowtie2, and differentially expressed genes were detected with Rsem and EdgeR, all of which are equipped in Trinity package (*57*,*58*).

Divergence time estimation

All sequences of interest in diatoms were first clustered using cd-hit/4.6.8, removing sequences of 100% alignment and clustering at low identity. The largest clusters for each gene family was then aligned using PRANK 170427 (*111*), poorly aligned sequences were removed with TrimAl 1.2 (*112*) and put into gene blocks to remove gapped columns with Gblocks v0.91b (*113*). Aligned sequences containing all species with available data (N = 1 to 4) were then analysed using phylogenetic analyses. Divergence estimates were obtained using Bayesian Markov Chain Monte Carlo (MCMC) analyse is implemented in Beast 2.5.0 (*114*). The analysis was carried out

using a relaxed molecular clock approach with an uncorrected log-normal distribution model of rate of variation, the HKY substitution model, four gamma categories and a Yule model of speciation, for each gene family 5 runs were carried out with 20 million MCMC generations sampled every 1,000th generation. The results from all runs were combined and summarised using LOGcombiner v1.10.4, these were checked for convergence using Tracer v1.7.1 (http://tree.bio.ed.ac.uk/software/tracer/). A maximum clade credibility tree was generated using Tree Annotator v1.10.4 and was graphically visualised using FigTree v 1.4.3 software (http://tree.bio.ed.ac.uk/software/figtree/). The Phylogenetic Maximum Clade Credibility (MCC) trees were used to determine the rate of "speciation" (or more accurately, the expansion) within a given gene family. The R package TESS (*115*) was used for this analysis with the mass extinction turned off, measuring only for the speciation rate (expansion rate), using the phylogenetic trees produced in the divergence time estimations.

Likelihood ratio tests (2ΔL) were performed by PAML 4 (*116*) between 3 Codeml site model pairs (M0/M3; M1a/M2a and M7/M8), with the first model representing a site model without positive selection and the second a model that allows a proportion of sites to be under positive selection. In all 3 cases the model with positive selection was a significantly better fit to the data. For the M2a and M8 models, several sites were predicted to be positively selected by Bayes Empirical Bayes analysis (*117*). The sites 457 and 460 were identified by both models with high confidence (> 95%) and 457 by M8.

Abbreviations in figure legends

Fig. 1.
Glu: Glutamate, Gln: Glutamine, Orn: Ornithine, NH^{4+}: ammonium, NO_2: nitrite, N-Acetyl-Glu: N-Acetylglutamate, N-Acetyl-Glu-5P: N-Acetylglutamyl-5phosphate, N-Acetyl-Glu-semialdehyde: N-Acetylglutamyl-semialdehyde, a-ketoglutarate: alpha-ketoglutarate, PPP-IX: protoporphyrin-IX, ALA: 5-aminolevulinic acid, GSAH: Glutamate-1-semialdehyde, aa-tRNA-Glu: amino acyl-tRNA-glutamate, tRNA-E: glutamyl tRNA, Chl *a*: chlorophyll *a*, α-TT: alpha-Tocotrierol, β-TT: beta-tocotrierol, α-TP: alpha-tocopherol, β-TP: beta-tocopherol, PQ: plastoquinone/plastoquinol, GA3P: glyceroaldehyde-3phosphate, Pyr: Pyruvate, IPP: Isopentenylpyrophosphate, DPP: Dimethylallylpyrophosphate, Phy: Phytoene, β-Car: beta-carotene, DHB4P: 3,4-Dihydroxy 2 butanone 4phosphate, DMR: 6,7-Dimethyl-8- ribityl umazine, Asp: Aspartate, Lys: Lysine, Ile: Isoleucine, Leu: Leucine, Val: Valine, MM: Methylmalate, ACL: Acetolactate, AcCoA: Acetyl-CoA, ACP: acyl-carrier protein, 3KA-ACP: 3ketoacyl-ACP, A-ACP: acyl-ACP, E-ACP: enoyl-ACP, 3HA-ACP: 3hydroxyacyl-ACP, PEP: phosphoenolpyruvate, G2P: 2 phosphoglycerate, G3P: 3 phosphoglycerate, DAH7P: 3-deoxy-7-phosphoheptulonate, DHQ: 3-dehydroquinic acid, DHS: 3-dehydroshikimate, S3P: shikimate

3phosphate, EPS3P: 5-enolpyruvylshikimate -3-phosphate, CM: Chorismate, Trp: Tryptophan, Tyr: Tyrosine, Phe: Phenylalanine, G1,2P2: Glycerol 1,2 bisphosphate, R1,5P2: Ribulose 1,5 bisphosphate, Ru5P: Ribulose 5phosphate, Ri5P: Ribose 5phosphate, S7P: sedoheptulose 7-phosphate, E4P: Erythrose 4phosphate, F6P: Fructose 6phosphate, X5P: Xylulose 5phosphate, F1,6P2: Fructose 1,6 bisphosphate, Gly3P: Glycerate 3phosphate, LysoPA: Lysophosphatidic acid, PA: Phosphatidic acid, PG: Phosphatidyl glycerol, G6P: Glucose 6phosphate, G1P: Glucose 1phosphate, UDPG: UDP-glucose, UDPSQ: UDP-sulfoquinovose, SQDG: Sulfoquinovosyl diacylglycerol, SO_4^{2-}: sulfate, Cys: Cysteine, FeS: Iron-sulfur cluster, Ala: Alanine, Cyt: cytochrome *b6*/*f* complex, PSI: photosystem I, PSII: Photosystem II.

Fig. 2
NH^{4+}: ammonium, CO_2: carbon dioxide, HCO_3^-: bicarbonate, Arg: Arginine, Orn: Ornithine, Asp: Aspartate, βox: fatty acid β oxidation, Gln: Glutamine, Glu: Glutamate, a-KG: alpha-ketoglutarate, Ser: Serine, Gly: Glycine, GCS: Glycine cleavage system, Ala: Alanine, TCA: Tricarboxylic acid cycle, OAA: oxaloacetate, BCAA: Branched chain amino acid synthesis, AAA: aromatic amino acid synthesis. Other abbreviations are described in Fig. 1.

A

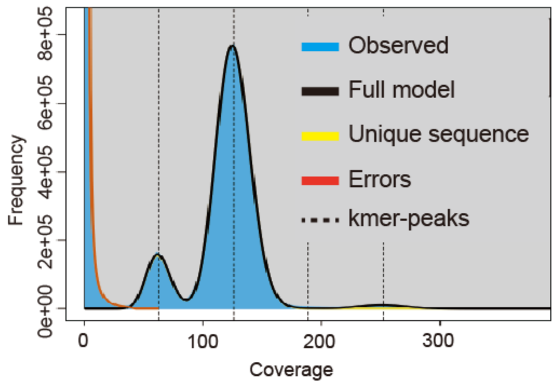

property	max
Heterozygosity	0.32881%
Genome Haploid Length	33,216,246 bp
Genome Repeat Length	3,592,209 bp
Genome Unique Length	29,624,037 bp
Model Fit	97.8751%
Read Error Rate	0.584794%

property	min
Heterozygosity	0.324931%
Genome Haploid Length	33,205,190 bp
Genome Repeat Length	3,591,013 bp
Genome Unique Length	29,614,176 bp
Model Fit	96.3579%
Read Error Rate	0.584794%

B

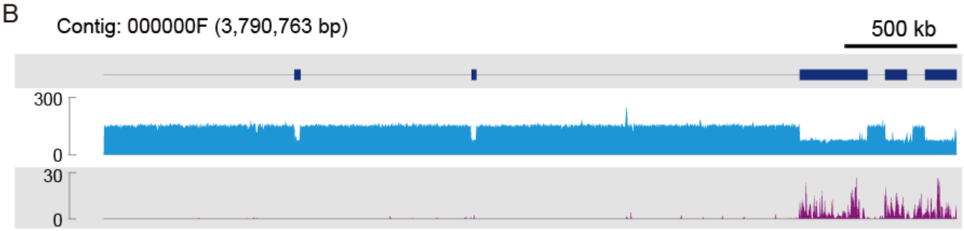

C

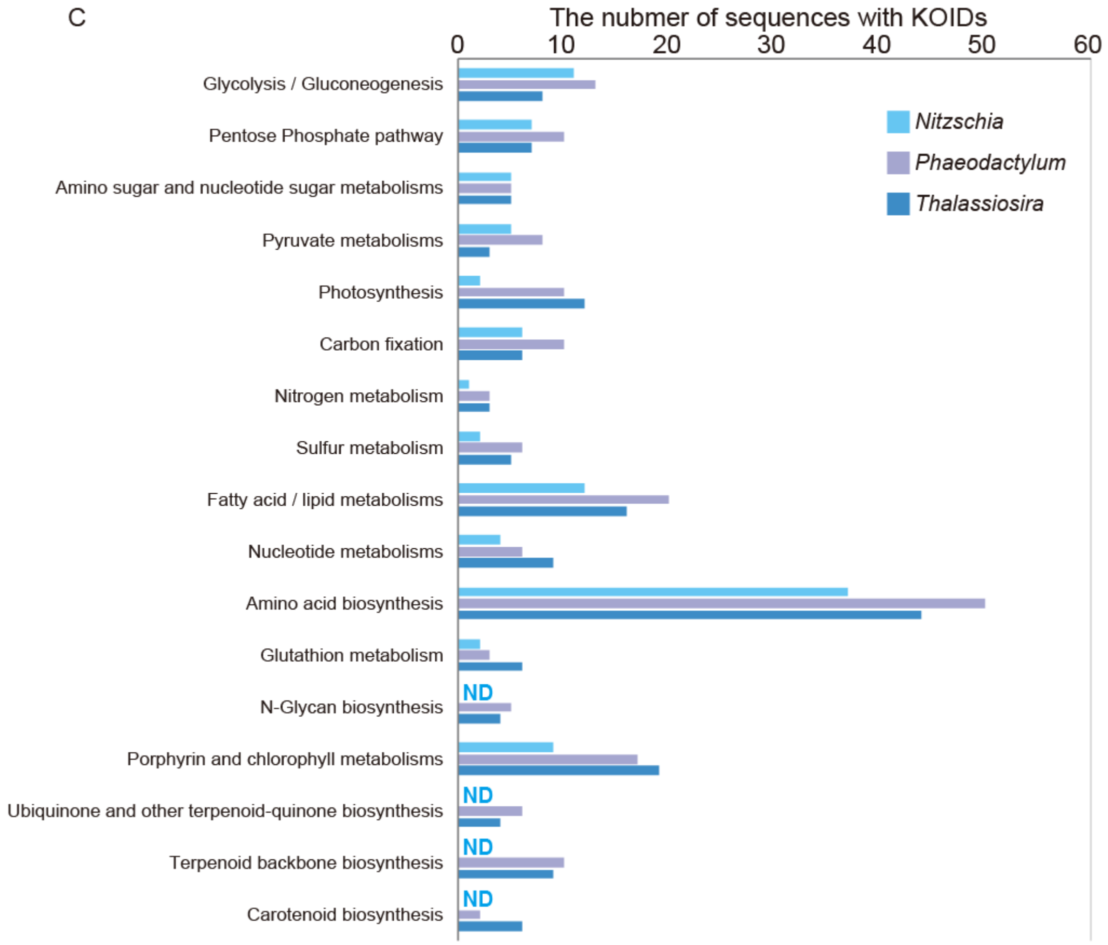

Fig. S1.

Genomic features showing diploid genome structure with a portion of heterozygosity and the encoded plastid proteome. A. GenomeScope analysis. **B.** Read mapping of Illumina short reads onto the primary contig 000000F. Dark blue boxes show primary contig regions corresponding to haplotigs. Light blue shows the read depth, while purple shows the number of variants, such as SNPs and Indels, per 1,000 bp. **C.** Comparison of KO ID numbers among plastids of the three diatoms. Each bar indicates numbers of unique KO IDs in each functional category. ND: not detected.

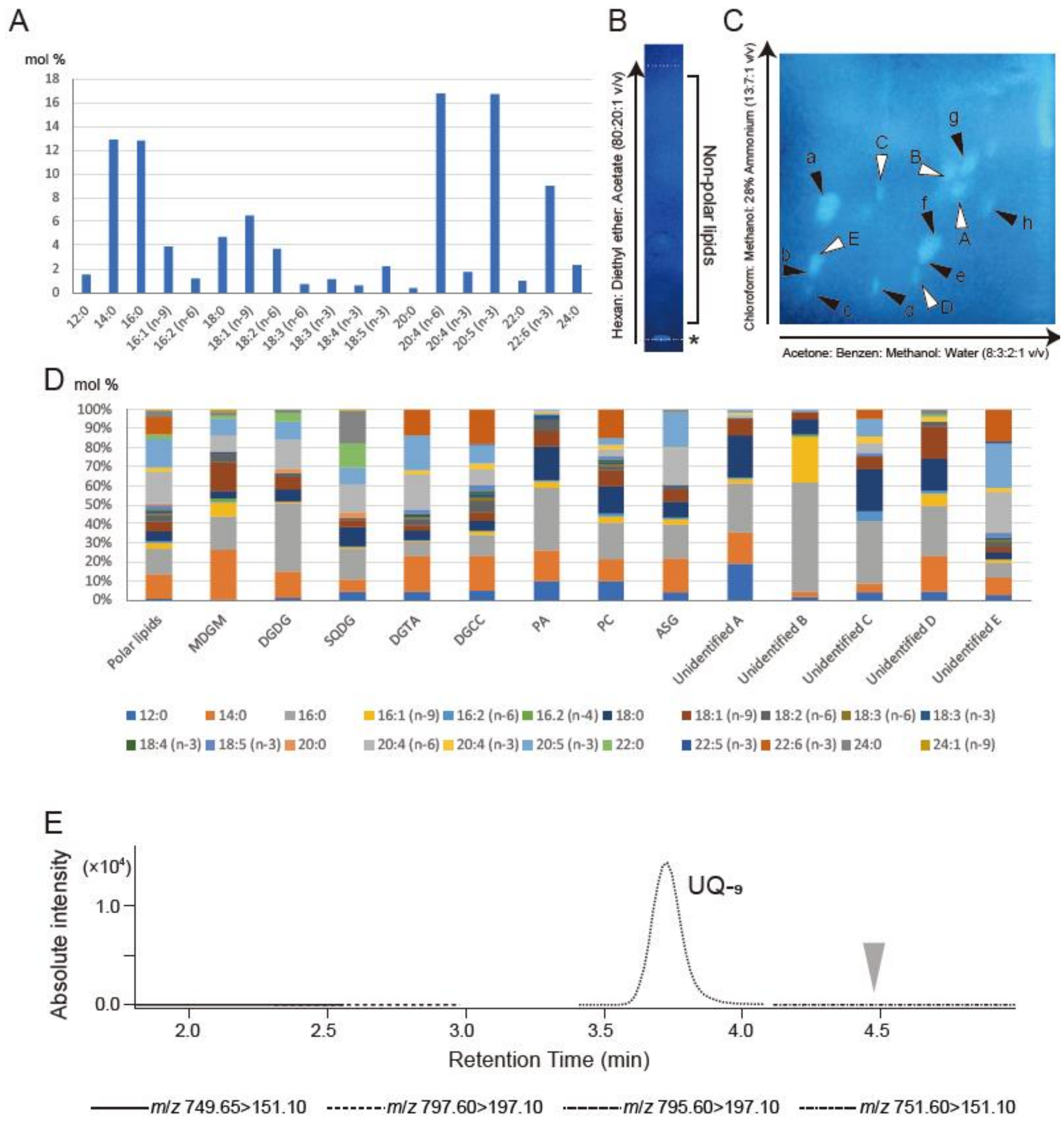

Fig. S2.

Fatty acids, lipids, and quinones in *Nitzschia putrida* NIES-4239. A. Fatty acid composition of the crude lipid extract. Crude lipids were methylated after extraction and subjected to gas chromatography. **B.** Thin layer chromatograph (silica gel 60) of the crude lipids developed by hexane/diethyl ether/acetate (80:20:1 v/v/v). Lipids were visualized under UV light at 365 nm after spraying the plate with 0.01% (w/v) primulin in 80% (v/v) acetone. Although lipids of 19 mg dry weight cells were applied, explicit spots of neutral lipids were not appeared. Polar lipids were indicated by an asterisk. **C.** Two-dimensional thin layer chromatograph of the polar lipids.

Solvent systems: first dimension,acetone/benzene/methanol/water (8:3:2:1 v/v/v/v); second dimension, chloroform/methanol/28% ammonium (13:7:1 v/v/v). Each spot of polar lipids was detected as described above. Each spot of lipids was detected as polar lipids by primuline under UV. Each lipid was identified by the retardation factors of standard materials and the color reaction. a: Diacylglycerylhydroxymethyltrimethyl- β -alanine, b: Diacylglyceryl carboxyhydroxymethylcholine, c: Phosphatidylcholine, d: Phosphatidic acid, e: Digalactosyl diacylglycerol, f: Sulfoquivonosyl diacylglycerol, g: Monogalactosyl diacylglycerol, h: Acylated steryl glycoside, Open arrowheads A - E: unidentified lipids A – E. We could not detect phosphatidyl glycerol (PG), supporting absence of PG biosynthesis. D. Fatty acid compositions in each spot appeared in C. E. LC-MS/MS chromatograms (multiple reaction monitoring mode) of the acetone extract after oxidative treatment with ferric chloride. The grey arrow indicates the retention time for plastoquinone peak in this system (*7*).

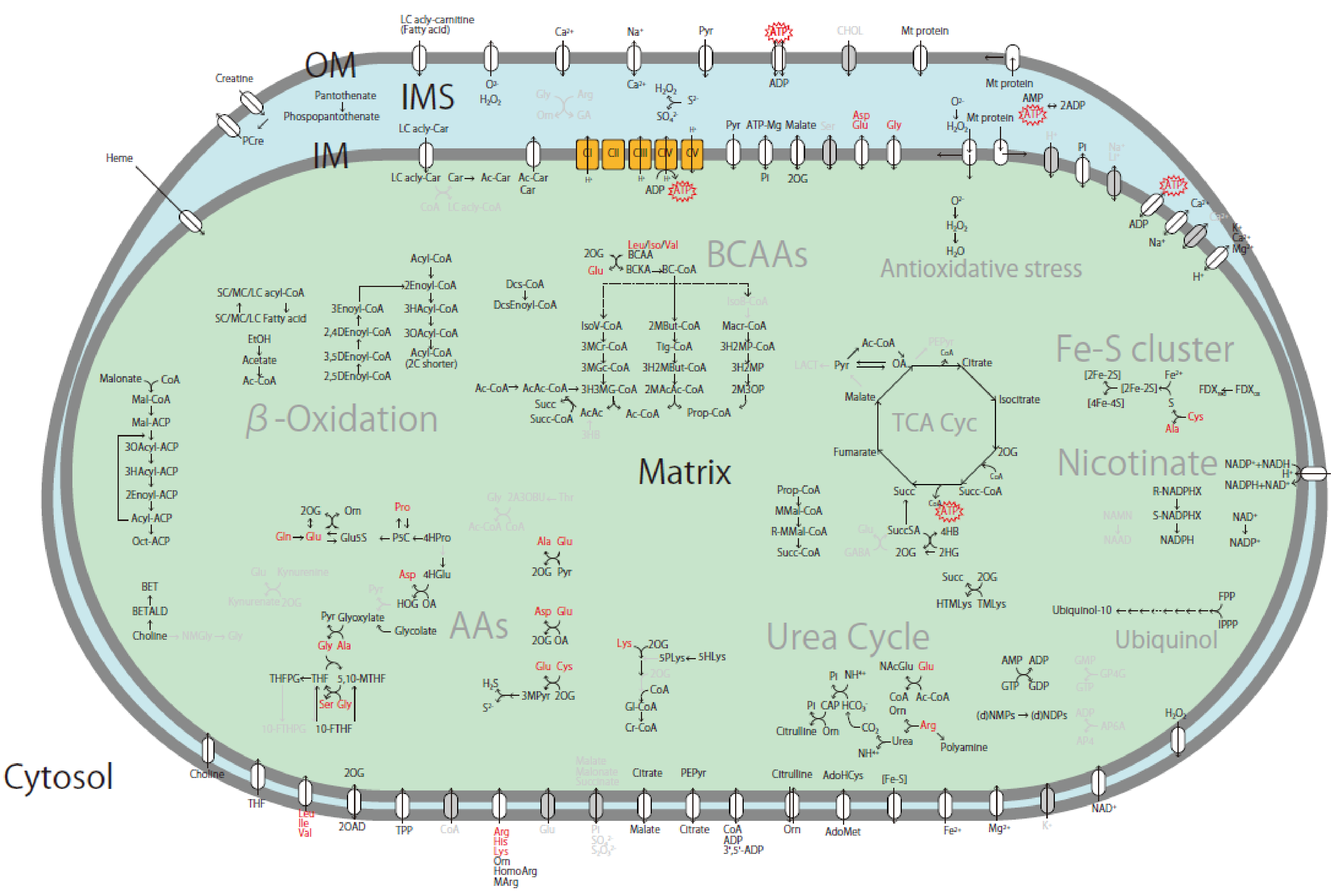

OM
IMS
IM
Cytosol
Matrix
β-Oxidation
BCAAs
Antioxidative stress
Fe-S cluster
TCA Cyc
Nicotinate
AAs
Urea Cycle
Ubiquinol

Fig. S3.

Mitochondrial metabolisms predicted from the genome data of *Nitzschia putrida.*

Overview of the metabolic pathway of the mitochondria based on in silico analyses. OM, IMS, and IM: outer membrane, intermembrane space, and inner membrane, respectively. Arrows indicates metabolic reactions. Essential amino acids and ATP are highlighted in red. Ovals indicates membrane translocators. Undetectable reactions and their involving substrates are shown in gray. Abbreviations: 10-FTHF: 10-Formyltetrahydrofolate, 10-FTHPG: 10-formyltetrahydrofolate polyglutamate, 2:4DEnoyl-CoA: 2:4-dienoyl-CoA, 2:5DEnoyl-CoA: 2:5-dienoyl-CoA, 2A3OBU: 2-Amino-3-oxobutanoic acid, 2Enoyl-ACP: 2-enoyl-ACP, 2Enoyl-CoA: 2-enoyl-CoA, 2HG: 2-Hydroxyglutarate, 2M3OP: 2-Methyl-3-oxopropanoate, 2MAcAc-CoA: 2-Methylacetoacetyl-CoA, 2MBut-CoA: 2-Methylbutanoyl-CoA, 2OAD: 2-oxoadipate, 2OG: 2-oxoglutarate, 3:5DEnoyl-CoA: 3:5-dienoyl-CoA, 3Enoyl-CoA: 3-enoyl-CoA, 3H2MBut-CoA: 3-Hydroxy-2-methylbutanoyl-CoA, 3H2MP: 3-Hydroxy-2-methylpropanoate, 3H2MP-CoA: 3-hydroxy-2-methylpropanoyl-CoA, 3H3MGl-CoA: 3-Hydroxy-3-methylglutaryl-CoA, 3HAcyl-ACP: 3-hydroxyacyl-ACP, 3HAcyl-CoA: 3-hydroxyacyl-CoA, 3HB: 3-Hydroxybutanate, 3MCr-CoA: 3-methylcrotonoyl-CoA, 3MGc-CoA: 3-Methylglutaconyl-CoA, 3MPyr: 3-Mercaptopyruvate, 3OAcyl-ACP: 3-oxoacyl-ACP, 3OAcyl-CoA: 3-oxoacyl-CoA, 4HB: 4-Hydroxybutanoate, 4HGlu: 4-Hydroxy-L-glutamate, 4HPro: 4-Hydroxyproline, 5:10-MTHF: 5:10-methylenetetrahydrofolate, 5HLys: 5-Hydroxy-L-lysine, 5PLys: 5-Phosphooxy-L-lysine, Ac-Car: Acetylcarnitine, Ac-CoA: Acetyl-CoA, AcAc: Acetoacetate, AcAc-CoA: Acetoacetyl-CoA, ACP: Acyl-carrier protein, AdoHCys: S-Adenosyl-L-homocysteine, AdoMet: S-Adenosyl-L-methionine, AP4: Adenosine tetraphosphate, AP6A: Diadenosine hexaphosphate, BC-CoA: Branched-chain acyl-CoA, BCAA: Branched-chain amino acid, BCKA: Branched-chain ketoacid (2-oxoacid), CAP: Carbamoyl phosphate, Car: L-Carnitine, CHOL: Cholesterol, CI - CV: Complex I - V, CoA: Coenzyme A, Cr-CoA: Crotonoyl-CoA, Dcs-CoA: Docosanoyl-CoA, DcsEnoyl-CoA: Docos-2-enoyl-CoA, FDX: Ferredoxin, FPP: Farnesyl diphosphate, GA: Guanidinoacetate, Gl-CoA: Glutaryl-CoA, Glu5S: Glutamate 5-semialdehyde, GP4G: Diguanosine tetraphosphate, HOG: 4-Hydroxy-2-oxoglutarate, HomoArg: Homoarginine, HTMLys: Hydroxy-trimethyllysine, IPPP: Isopentenyl diphosphate, IsoB-CoA: Isobutyryl-CoA, IsoV-CoA: Isovaleryl-CoA, LACT: Lactate, LC acly-Car: Long-chain acyl-Carnitine, LC acyl-CoA: Long-chain acyl-CoA, Macr-CoA: Methacrylyl-CoA, Mal-ACP: Malonyl-ACP, Mal-CoA: Malonyl-CoA, MArg: Methylarginine, MC acyl-CoA: Medium-chain acyl-CoA, MMal-CoA: Methylmalonyl-CoA, NAAD: Nicotinamide adenine dinucleotide, NAcGlu: N-Acetyl-L-glutamate, NAD: Nicotinamide adenine dinucleotide, NAMN: Nicotinamide mononucleotide, NMGly: N-Methacryloylglycinamide, OA: Oxaloacetate, Oct-ACP: Octanoyl-ACP, Orn: Ornithine, P5C: Pyrroline-5-carboxylate, PCre: Phosphocreatine, PEPyr: Phosphoenolpyruvate, Pi: Phosphate, Prop-CoA: Propanoyl-CoA, Pyr: Pyruvate, R-MMal-CoA: R-Methylmalonyl-CoA, R-NADPHX: R-NADPH-hydrate, S-NADPHX: S-NADPH-hydrate, SC acyl-CoA: Short-chain acyl-CoA, Succ: Succinate, Succ-CoA: Succinyl-CoA, SuccSA: Succinate semialdehyde, THF: Tetrahydrofolate, THFPG: Tetrahydrofolate polyglutamate, Tig-CoA: Tiglyl-CoA, TMLys: Trimethyllysine, TPP: Thiamine diphosphate.

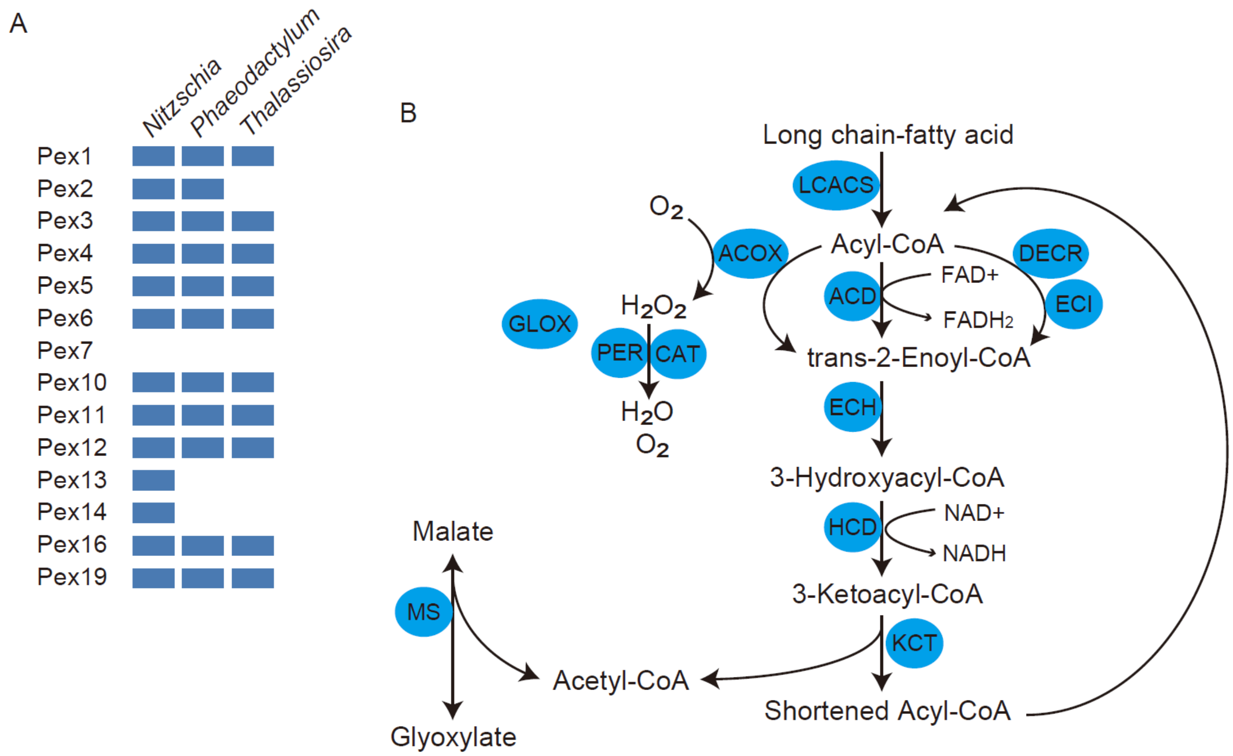

Fig. S4.

Peroxisomal metabolisms and peroxin compositions predicted from the genome data of *Nitzschia putrida.* **A.** Distribution of Pex factors. Genes identified in the diatom genomes are shown by blue boxes. *Nitzschia*: *N. putrida*, *Phaeodactylum*: *P. tricornutum*, *Thalassiosira*: *T. pseudonana*. **B.** Predicted peroxisomal metabolism in *N. putrida*. Arrows show metabolic reactions while grey circles show responsible enzymes detected in the genome. ACD: Acyl-CoA dehydrogenase, ACOX: acyl-CoA oxidase, CAT: catalase, DECR: peroxisomal 2,4-dienoyl-CoA reductase, ECH: 3-alpha,7-alpha,12-alpha-trihydroxy-5-beta-cholest-24-enoyl-CoA hydratase, ECI: peroxisomal 3,2-trans-enoyl-CoA isomerase, GLOX: glutathione-dependent disulfide-bond oxidoreductase, HCD: 3-hydroxyacyl-CoA dehydrogenase, KCT: beta-ketoacyl-coa thiolase, LCACS: long-chain acyl-CoA synthetase, MS: malate synthase, PER: peroxiredoxin.

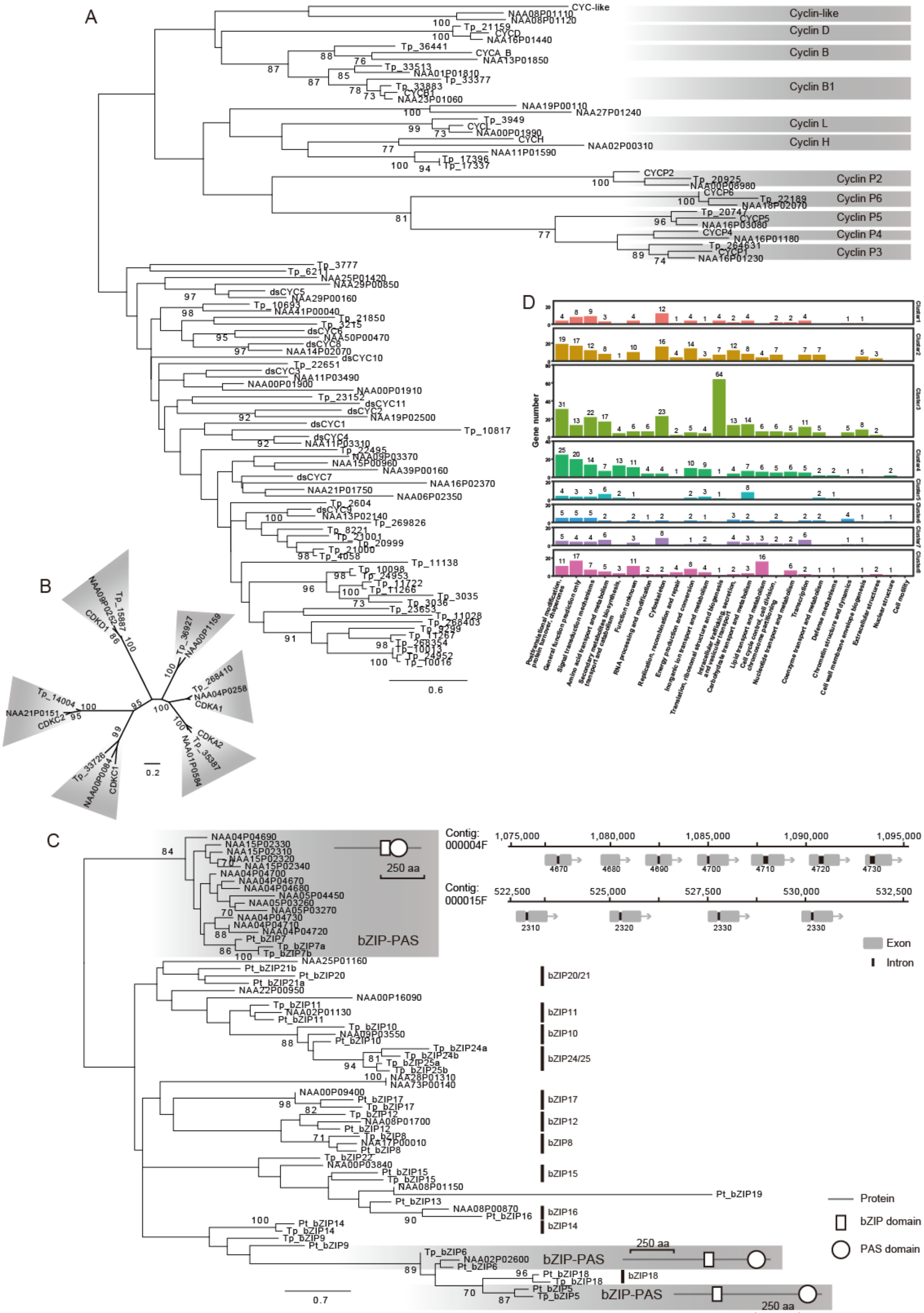

A
CYC-like
NAA08P01110
NAA08P01120
Tp_21159
CYCD
NAA16P01440
Tp_36441
CYCA_B
NAA13P01850
Tp_33513
NAA01P01810
Tp_33377
Tp_33883
CYCB1
NAA23P01060
NAA19P00110
NAA27P01240
Tp_3949
CYCL
NAA00P01990
CYCH
NAA02P00310
NAA11P01590
Tp_17396
Tp_17337
CYCP2
Tp_20925
NAA00P08980
CYCP6
Tp_22189
NAA19P02070
Tp_20747
CYCP5
NAA16P03080
CYCP4
NAA16P01180
Tp_264631
CYCP1
NAA16P01230
Cyclin-like
Cyclin D
Cyclin B
Cyclin B1
Cyclin L
Cyclin H
Cyclin P2
Cyclin P6
Cyclin P5
Cyclin P4
Cyclin P3
Tp_3777
Tp_6211
NAA25P01420
NAA29P00850
dsCYC5
NAA29P00160
Tp_10693
NAA41P00040
Tp_21850
Tp_3215
dsCYC6
NAA50P00470
dsCYC8
NAA14P02070
dsCYC10
Tp_22651
dsCYC3
NAA11P03490
NAA00P01900
NAA00P01910
Tp_23152
dsCYC11
dsCYC2
NAA19P02500
dsCYC1
Tp_10817
dsCYC4
NAA11P03310
Tp_22495
NAA09P03370
NAA15P00960
NAA39P00160
dsCYC7
NAA16P02370
NAA21P01750
NAA06P02350
Tp_2604
dsCYC9
NAA13P02140
Tp_269826
Tp_8221
Tp_21001
Tp_20999
Tp_21000
Tp_4058
Tp_11138
Tp_10098
Tp_24953
Tp_11722
Tp_11266
Tp_3035
Tp_3036
Tp_23653
Tp_11028
Tp_268403
Tp_3299
Tp_11267
Tp_268354
Tp_10013
Tp_24952
Tp_10016
0.6
B
Tp_15887
NAA09P02520
CDKD1
Tp_36927
NAA00P1159
Tp_268410
NAA04P0258
CDKA1
CDKA2
Tp_35387
NAA01P0584
Tp_33726
NAA00P0084
CDKC1
Tp_14004
NAA21P0151
CDKC2
0.2
C
NAA04P04690
NAA15P02330
NAA15P02310
NAA15P02320
NAA15P02340
NAA04P04700
NAA04P04670
NAA04P04680
NAA05P04450
NAA05P03260
NAA05P03270
NAA04P04730
NAA04P04710
NAA04P04720
Pt_bZIP7
Tp_bZIP7a
Tp_bZIP7b
bZIP-PAS
250 aa
Contig: 000004F
1,075,000
1,080,000
1,085,000
1,090,000
1,095,000
4670
4680
4690
4700
4710
4720
4730
Contig: 000015F
522,500
525,000
527,500
530,000
532,500
2310
2320
2330
2330
Exon
Intron
NAA25P01160
Pt_bZIP21b
Pt_bZIP20
Pt_bZIP21a
NAA22P00950
bZIP20/21
NAA00P16090
Tp_bZIP11
NAA02P01130
Pt_bZIP11
bZIP11
Tp_bZIP10
NAA09P03550
Pt_bZIP10
bZIP10
Tp_bZIP24a
Tp_bZIP24b
Tp_bZIP25a
Tp_bZIP25b
NAA28P01310
NAA73P00140
bZIP24/25
NAA00P09400
Pt_bZIP17
Tp_bZIP17
bZIP17
Tp_bZIP12
NAA08P01700
Pt_bZIP12
bZIP12
Tp_bZIP8
NAA17P00010
Pt_bZIP8
bZIP8
Tp_bZIP22
NAA00P03840
Pt_bZIP15
Tp_bZIP15
bZIP15
NAA08P01150
Pt_bZIP19
Pt_bZIP13
NAA08P00870
Pt_bZIP16
bZIP16
bZIP14
Pt_bZIP14
Tp_bZIP14
Tp_bZIP9
Pt_bZIP9
Tp_bZIP6
NAA02P02600
Pt_bZIP6
bZIP-PAS
Pt_bZIP18
Tp_bZIP18
bZIP18
Pt_bZIP5
Tp_bZIP5
bZIP-PAS
250 aa
0.7
Protein
bZIP domain
PAS domain
D
Gene number
Cluster1
Cluster2
Cluster3
Cluster4
Cluster5
Cluster6
Cluster7
Cluster8
Posttranslational modification, protein turnover, chaperones
General function prediction only
Signal transduction mechanisms
Amino acid transport and metabolism
Secondary metabolites biosynthesis, transport and catabolism
Function unknown
RNA processing and modification
Cytoskeleton
Replication, recombination and repair
Energy production and conversion
Inorganic ion transport and metabolism
Translation, ribosomal structure and biogenesis
Intracellular trafficking, secretion, and vesicular transport
Carbohydrate transport and metabolism
Lipid transport and metabolism
Cell cycle control, cell division, chromosome partitioning
Nucleotide transport and metabolism
Transcription
Coenzyme transport and metabolism
Defense mechanisms
Chromatin structure and dynamics
Cell wall/membrane/envelope biogenesis
Extracellular structures
Nuclear structure
Cell motility

Fig. S5.

Phylogenetic diversity of genes for cell cycle regulation and transcription and functional annotation of differentially expressed genes. A. Cyclin gene phylogeny of diatoms. CYC and dsCYC are cyclin and diatom-specific cyclins of *Phaeodactylum tricornutum*, respectively (*32*). Tp: *Thalassiosira pseudonana*. Numbers of *Thalassiosira* homologues are of protein ID in *T. pseudonana* genome v3 in JGI. **B.** Cyclin-dependent kinase phylogeny of diatoms. CDKs are *Phaeodactylum* homologues (32). **C.** bZIP phylogeny in diatoms. Tp_bZIP and Pt_bZIP are of bZIP homologues in *T. pseudonana* and *P. tricornutum* according to (*37*). Domain structures are depicted for bZIP homologues with PAS domain (bZIP5-bZIP7). Positions of genes for duplicated and diversified homologues of bZIP7 with PAS domain in *N. putrida* are also depicted. Many of them are of tandem duplication. **D.** KOG-based functional annotation of differentially expressed genes clustered in Fig. 2D.

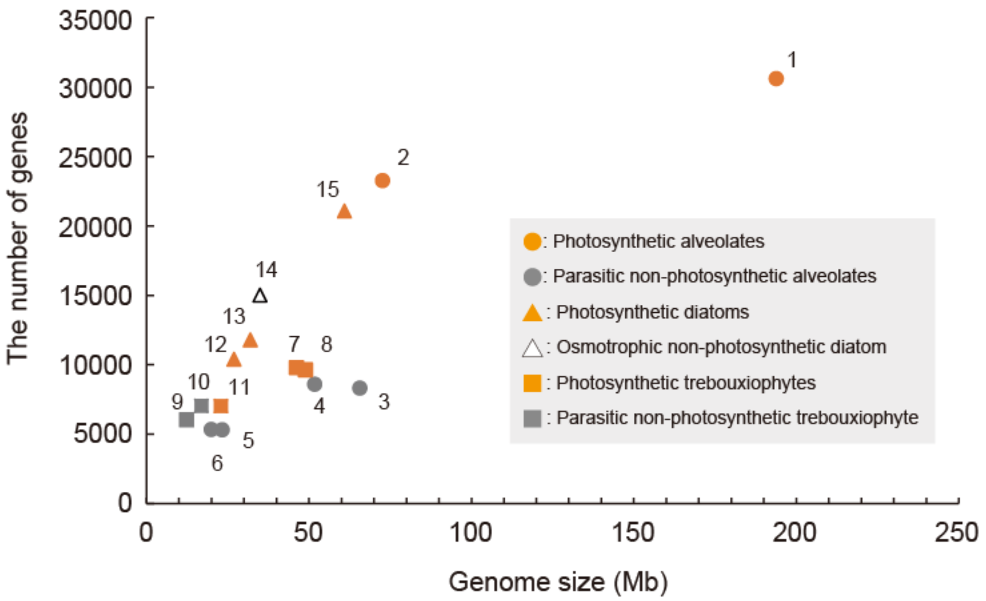

Fig. S6.

The genome sizes and the number of encoded proteins in eukaryotes that have lost photosynthesis secondarily and their close photosynthetic relatives. 1. *Chromera velia*, 2. *Vitrella brassicaformis*, 3. *Toxoplasma gondii* ME49, 4. *Eimeria tenera* Houghton, 5. *Plasmodium falciparum* 3D7, 6. *Hepatocystis* sp. ex *Piliocolobus tephrosceles* 2019, 7. *Chlorella variabilis* NC64A, 8. *Coccomyxa subellipsoidea* C-169, 9. *Helicosporidium* sp. 10. Prototheca stagnora, 11. *Auxenochlorella protothecoides*, 12. *Phaeodactylum tricornutum*, 13. *Thalassiosira pseudonana*, 14. *Nitzschia putrida*, 15. *Fragilariopsis cylindrus*. Data of *C. velia* and *V. brassicaformis* are from CryptoDB (https://cryptodb.org/cryptodb/app/), those of *T. gondii* and *E. tenera* are from ToxoDB (https://toxodb.org/toxo/app/), those of *P. falciparum* and *Hepatocystis* sp. are from PlasmoDB (https://plasmodb.org/plasmo/app/), and those of *C. variabilis*, *C. subellipsoidea*, *A. protothecoides*, *P. tricornutum*, *T. pseudonana*, and *F. cylindrus* are from JGI Genome Portal (https://genome.jgi.doe.gov/portal/). *Helicosporidium* and *Prototheca* genomes are from Pombert et al. (*118*) and Suzuki et al. (*119*), respectively.

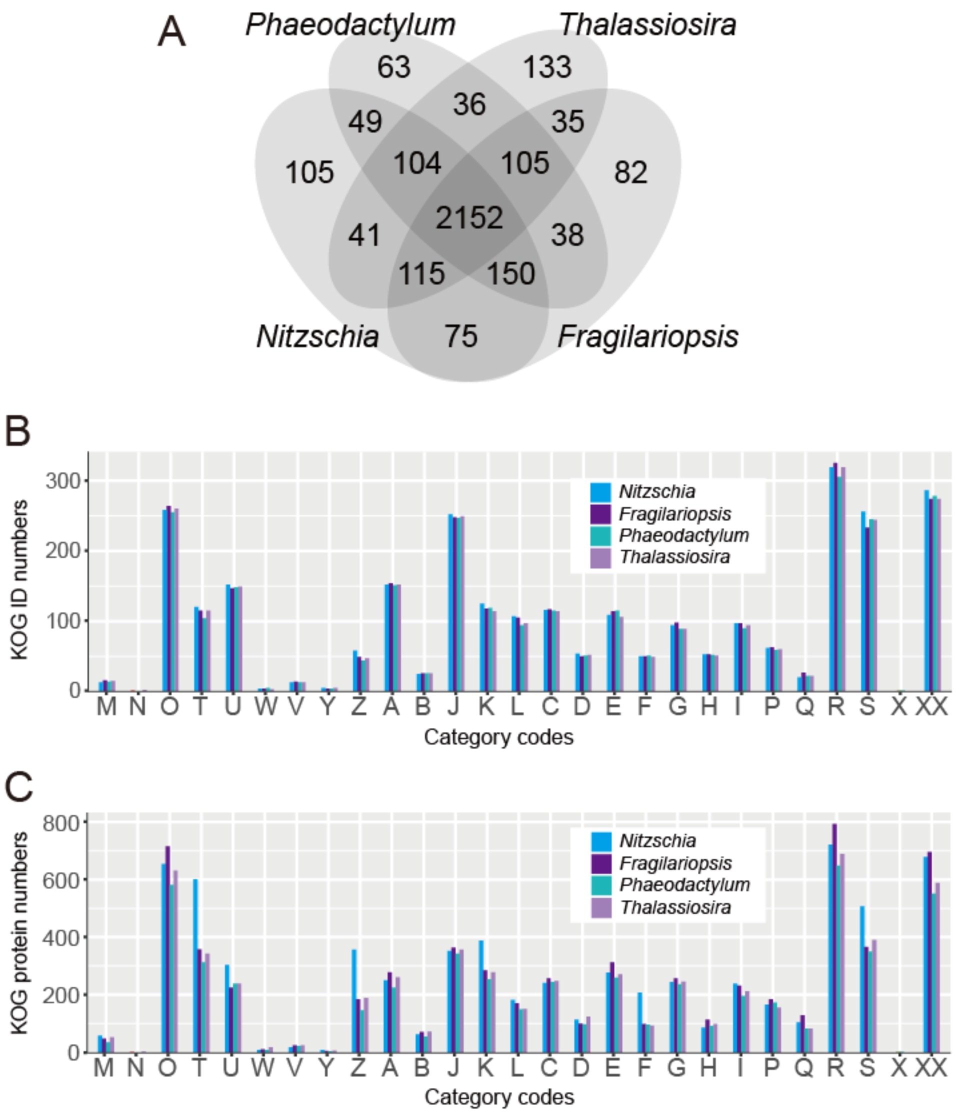

Fig. S7.

KOG in *N. putrida*. A. Venn diagram of KOG IDs shared by four diatoms. Nitzschia: Nitzschia putrida, Phaeodactylum: Phaeodactylum tricornutum, Thalassiosira: Thalassiosira pseudonana, Fragilariopsis: Fragilariopsis cylindrus. **B**. Comparison of the number of KOG ID among diatoms. KOG categories are as follows: A, RNA processing and modification; B, chromatin structure and dynamics; C, energy production and conversion; D, cell cycle control, cell division and chromosome partitioning; E, amino acid transport and metabolism; F, nucleotide transport and metabolism; G, carbohydrate transport and metabolism; H, coenzyme transport and metabolism; I, lipid transport and metabolism; J, translation, ribosomal structure and biogenesis; K, transcription; L, replication, recombination and repair; M, cell wall, membrane or envelope biogenesis; N, cell motility; O, post-translational modification, protein turnover, chaperones; P, inorganic ion transport and metabolism; Q, secondary metabolites biosynthesis, transport and catabolism; R, general function prediction only; S, function unknown; T, signal transduction; U, intracellular trafficking, secretion and vesicular transport; V, defence mechanisms; W,

extracellular structures; Y, nuclear structure; Z, cytoskeleton. **C**. Comparison of the number of genes assigned to each KOG category. Other details are described above.

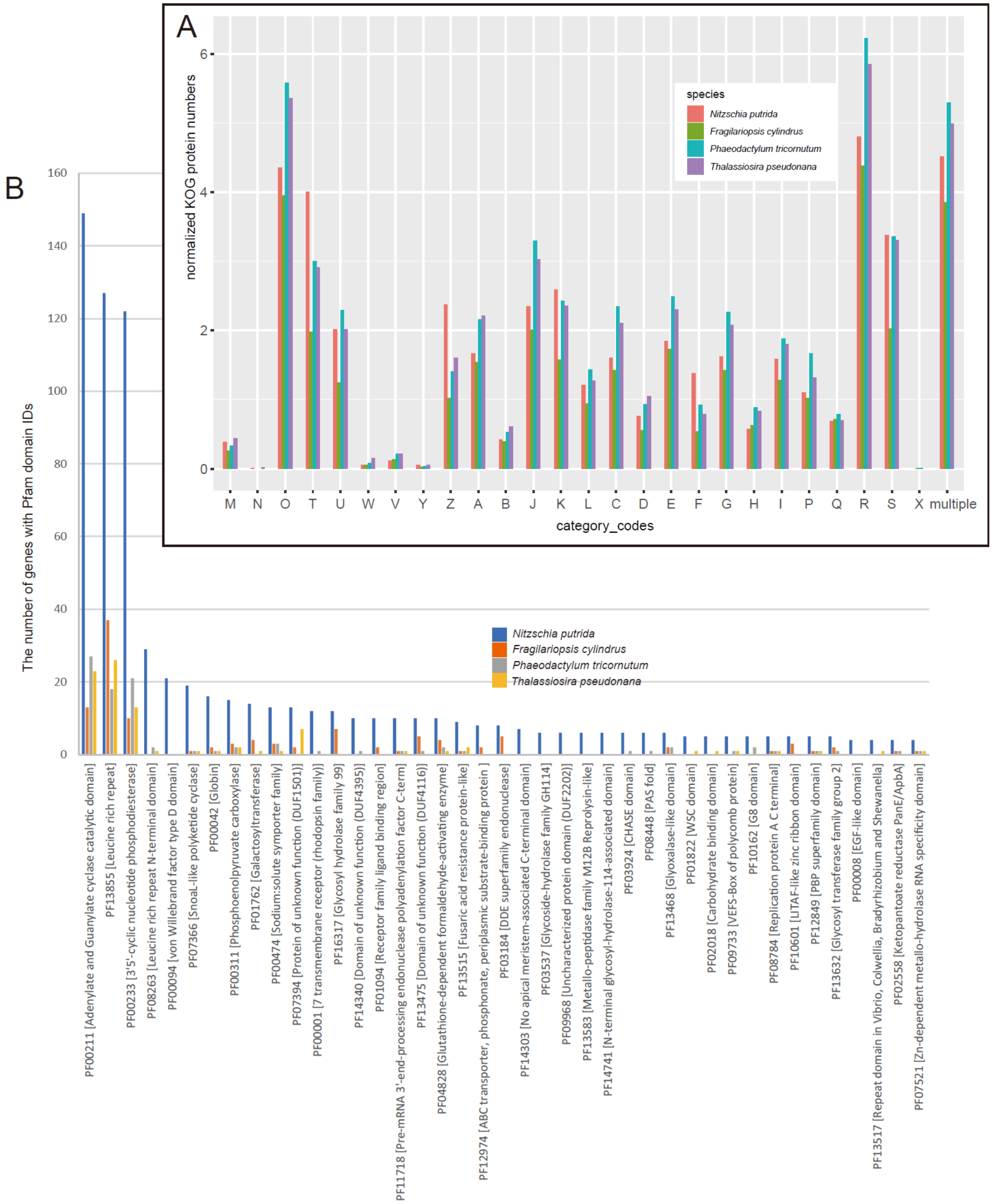

A
normalized KOG protein numbers
0
2
4
6
species
Nitzschia putrida
Fragilariopsis cylindrus
Phaeodactylum tricornutum
Thalassiosira pseudonana
M N O T U W V Y Z A B J K L C D E F G H I P Q R S X multiple
category_codes
B
The number of genes with Pfam domain IDs
0
20
40
60
80
100
120
140
160
Nitzschia putrida
Fragilariopsis cylindrus
Phaeodactylum tricornutum
Thalassiosira pseudonana
PF00211 [Adenylate and Guanylate cyclase catalytic domain]
PF13855 [Leucine rich repeat]
PF00233 [3'5'-cyclic nucleotide phosphodiesterase]
PF08263 [Leucine rich repeat N-terminal domain]
PF00094 [von Willebrand factor type D domain]
PF07366 [SnoaL-like polyketide cyclase]
PF00042 [Globin]
PF00311 [Phosphoenolpyruvate carboxylase]
PF01762 [Galactosyltransferase]
PF00474 [Sodium:solute symporter family]
PF07394 [Protein of unknown function (DUF1501)]
PF00001 [7 transmembrane receptor (rhodopsin family)]
PF16317 [Glycosyl hydrolase family 99]
PF14340 [Domain of unknown function (DUF4395)]
PF01094 [Receptor family ligand binding region]
PF11718 [Pre-mRNA 3'-end-processing endonuclease polyadenylation factor C-term]
PF13475 [Domain of unknown function (DUF4116)]
PF04828 [Glutathione-dependent formaldehyde-activating enzyme]
PF13515 [Fusaric acid resistance protein-like]
PF12974 [ABC transporter, phosphonate, periplasmic substrate-binding protein]
PF03184 [DDE superfamily endonuclease]
PF14303 [No apical meristem-associated C-terminal domain]
PF03537 [Glycoside-hydrolase family GH114]
PF09968 [Uncharacterized protein domain (DUF2202)]
PF13583 [Metallo-peptidase family M12B Reprolysin-like]
PF14741 [N-terminal glycosyl-hydrolase-114-associated domain]
PF03924 [CHASE domain]
PF08448 [PAS fold]
PF13468 [Glyoxalase-like domain]
PF01822 [WSC domain]
PF02018 [Carbohydrate binding domain]
PF09733 [VEFS-Box of polycomb protein]
PF10162 [G8 domain]
PF08784 [Replication protein A C terminal]
PF10601 [LITAF-like zinc ribbon domain]
PF12849 [PBP superfamily domain]
PF13632 [Glycosyl transferase family group 2]
PF00008 [EGF-like domain]
PF13517 [Repeat domain in Vibrio, Colwellia, Bradyrhizobium and Shewanella]
PF02558 [Ketopantoate reductase PanE/ApbA]
PF07521 [Zn-dependent metallo-hydrolase RNA specificity domain]

Fig. S8.

The number of genes to which 41 Pfam domain IDs are assigned in *Nitzschia putrida*. A. Comparison of the number of genes assigned to each KOG category, normalized by the total gene number of each genome. Other details are described Fig. 3F. B. Here chosen are Pfam IDs containing at least 4 *Nitzschia* sequences and of which assigned sequences in *Nitzschia* are as 4 or more times large as the mean number of sequences with the same Pfam IDs in the 3 photosynthetic diatoms.

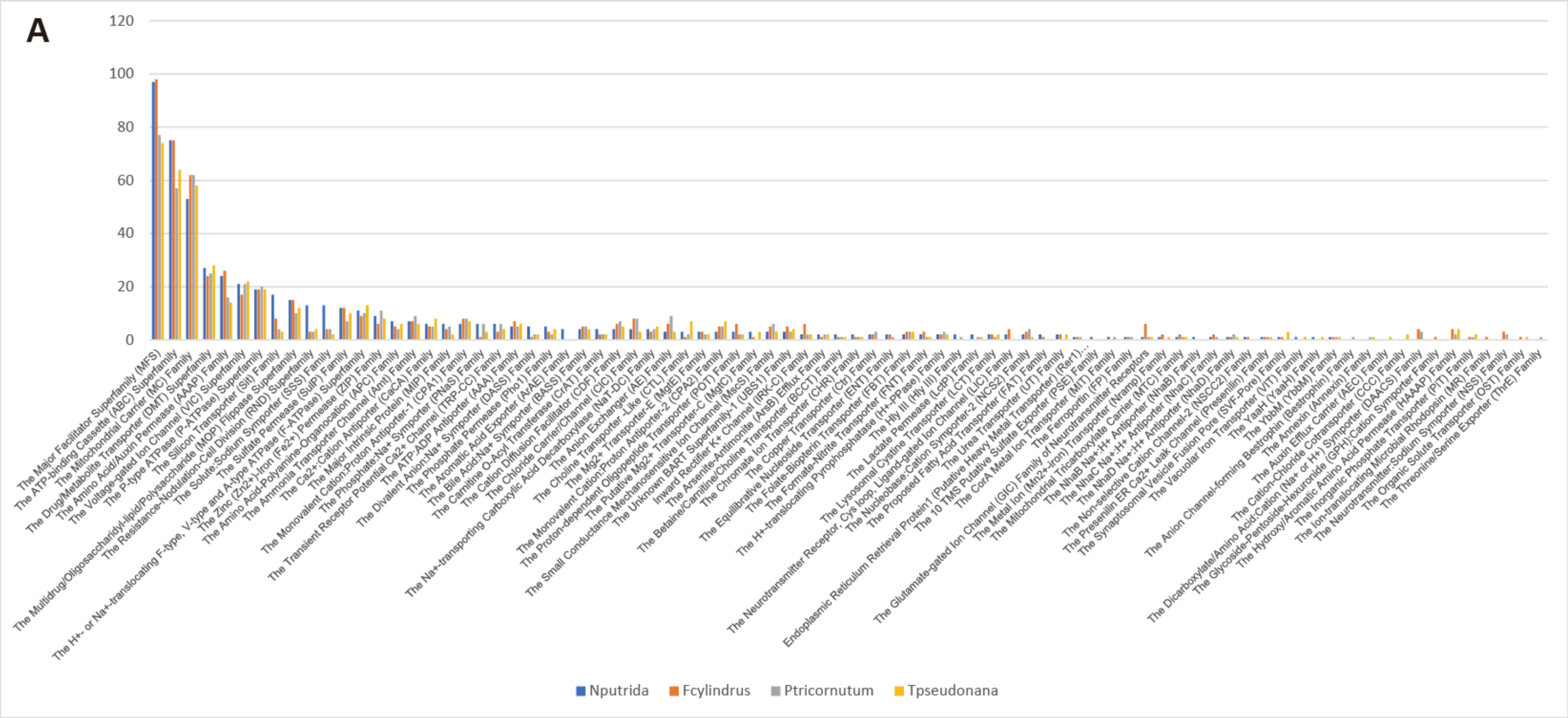
A
120
100
80
60
40
20
0
The Major Facilitator Superfamily (MFS)
The ATP-binding Cassette (ABC) Superfamily
The Mitochondrial Carrier (MC) Family
The Drug/Metabolite Transporter (DMT) Superfamily
The Amino Acid/Auxin Permease (AAAP) Family
The Voltage-gated Ion Channel (VIC) Superfamily
The P-type ATPase (P-ATPase) Superfamily
The Silicon Transporter (Sit) Family
The Multidrug/Oligosaccharidyl-lipid/Polysaccharide (MOP) Flippase Superfamily
The Resistance-Nodulation-Cell Division (RND) Superfamily
The Solute:Sodium Symporter (SSS) Family
The Sulfate Permease (SulP) Family
The H+- or Na+-translocating F-type, V-type and A-type ATPase (F-ATPase) Superfamily
The Zinc (Zn2+)-Iron (Fe2+) Permease (ZIP) Family
The Amino Acid-Polyamine-Organocation (APC) Family
The Ammonia Transporter Channel (Amt) Family
The Ca2+:Cation Antiporter (CaCA) Family
The Major Intrinsic Protein (MIP) Family
The Monovalent Cation:Proton Antiporter-1 (CPA1) Family
The Phosphate:Na+ Symporter (PNaS) Family
The Transient Receptor Potential Ca2+ Channel (TRP-CC) Family
The ATP:ADP Antiporter (AAA) Family
The Divalent Anion:Na+ Symporter (DASS) Family
The Phosphate Permease (Pho1) Family
The Aromatic Acid Exporter (ArAE) Family
The Bile Acid:Na+ Symporter (BASS) Family
The Carnitine O-Acyl Transferase (CrAT) Family
The Cation Diffusion Facilitator (CDF) Family
The Chloride Carrier/Channel (ClC) Family
The Na+-transporting Carboxylic Acid Decarboxylase (NaT-DC) Family
The Anion Exchanger (AE) Family
The Choline Transporter-like (CTL) Family
The Mg2+ Transporter-E (MgtE) Family
The Monovalent Cation:Proton Antiporter-2 (CPA2) Family
The Proton-dependent Oligopeptide Transporter (POT) Family
The Putative Mg2+ Transporter-C (MgtC) Family
The Small Conductance Mechanosensitive Ion Channel (MscS) Family
The Unknown BART Superfamily-1 (UBS1) Family
Inward Rectifier K+ Channel (IRK-C) Family
The Arsenite-Antimonite (ArsB) Efflux Family
The Betaine/Carnitine/Choline Transporter (BCCT) Family
The Chromate Ion Transporter (CHR) Family
The Copper Transporter (Ctr) Family
The Equilibrative Nucleoside Transporter (ENT) Family
The Folate-Biopterin Transporter (FBT) Family
The Formate-Nitrite Transporter (FNT) Family
The H+-translocating Pyrophosphatase (H+-PPase) Family
The Hly III (Hly III) Family
The Lactate Permease (LctP) Family
The Lysosomal Cystine Transporter (LCT) Family
The Neurotransmitter Receptor, Cys loop, Ligand-gated Ion Channel (LIC) Family
The Nucleobase:Cation Symporter-2 (NCS2) Family
The Proposed Fatty Acid Transporter (FAT) Family
The Urea Transporter (UT) Family
Endoplasmic Reticulum Retrieval Protein1 (Putative Heavy Metal Transporter) (Rer1)...
The 10 TMS Putative Sulfate Exporter (PSE) Family
The CorA Metal Ion Transporter (MIT) Family
The Ferroportin (FP) Family
The Glutamate-gated Ion Channel (GIC) Family of Neurotransmitter Receptors
The Metal Ion (Mn2+-iron) Transporter (Nramp) Family
The Mitochondrial Tricarboxylate Carrier (MTC) Family
The NhaB Na+:H+ Antiporter (NhaB) Family
The NhaC Na+:H+ Antiporter (NhaC) Family
The NhaD Na+:H+ Antiporter (NhaD) Family
The Non-selective Cation Channel-2 (NSCC2) Family
The Presenilin ER Ca2+ Leak Channel (Presenilin) Family
The Synaptosomal Vesicle Fusion Pore (SVF-Pore) Family
The Vacuolar Iron Transporter (VIT) Family
The YaaH (YaaH) Family
The YbbM (YbbM) Family
The Anion Channel-forming Bestrophin (Bestrophin) Family
The Annexin (Annexin) Family
The Auxin Efflux Carrier (AEC) Family
The Cation-Chloride Cotransporter (CCC) Family
The Dicarboxylate/Amino Acid:Cation (Na+ or H+) Symporter (DAACS) Family
The Glycoside-Pentoside-Hexuronide (GPH):Cation Symporter Family
The Hydroxy/Aromatic Amino Acid Permease (HAAAP) Family
The Inorganic Phosphate Transporter (PiT) Family
The Ion-translocating Microbial Rhodopsin (MR) Family
The Neurotransmitter:Sodium Symporter (NSS) Family
The Organic Solute Transporter (OST) Family
The Threonine/Serine Exporter (ThrE) Family
Nputrida
Fcylindrus
Ptricornutum
Tpseudonana

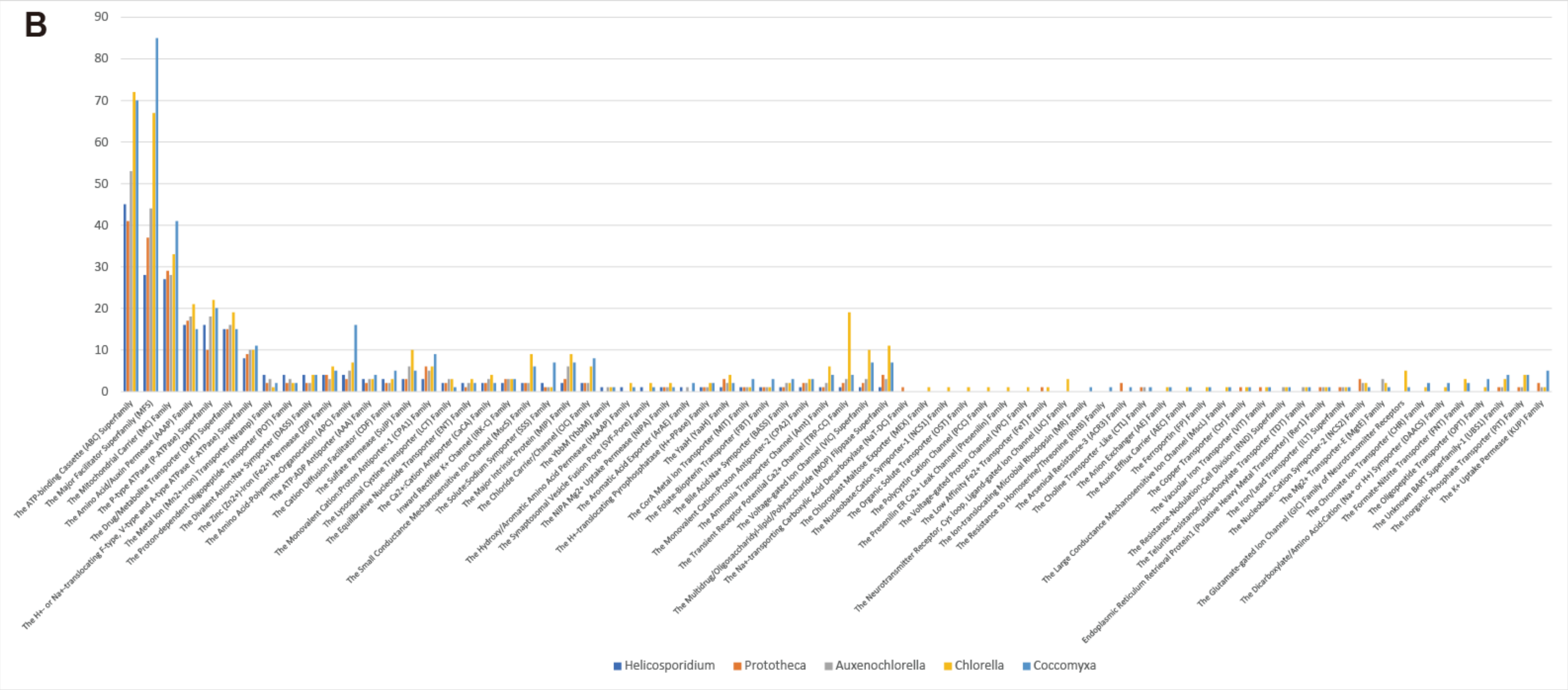

B
0
10
20
30
40
50
60
70
80
90
The ATP-binding Cassette (ABC) Superfamily
The Major Facilitator Superfamily (MFS)
The Mitochondrial Carrier (MC) Family
The Amino Acid/Auxin Permease (AAAP) Family
The P-type ATPase (P-ATPase) Superfamily
The Drug/Metabolite Transporter (DMT) Superfamily
The H+- or Na+-translocating F-type, V-type and A-type ATPase (F-ATPase) Superfamily
The Metal Ion (Mn2+-iron) Transporter (Nramp) Family
The Proton-dependent Oligopeptide Transporter (POT) Family
The Divalent Anion:Na+ Symporter (DASS) Family
The Zinc (Zn2+)-Iron (Fe2+) Permease (ZIP) Family
The Amino Acid-Polyamine-Organocation (APC) Family
The ATP:ADP Antiporter (AAA) Family
The Cation Diffusion Facilitator (CDF) Family
The Sulfate Permease (SulP) Family
The Monovalent Cation:Proton Antiporter-1 (CPA1) Family
The Lysosomal Cystine Transporter (LCT) Family
The Equilibrative Nucleoside Transporter (ENT) Family
The Ca2+:Cation Antiporter (CaCA) Family
Inward Rectifier K+ Channel (IRK-C) Family
The Small Conductance Mechanosensitive Ion Channel (MscS) Family
The Solute:Sodium Symporter (SSS) Family
The Major Intrinsic Protein (MIP) Family
The Chloride Carrier/Channel (ClC) Family
The YbhM (YbhM) Family
The Hydroxy/Aromatic Amino Acid Permease (HAAAP) Family
The Synaptosomal Vesicle Fusion Pore (SVF-Pore) Family
The NIPA Mg2+ Uptake Permease (NIPA) Family
The Aromatic Acid Exporter (ArAE) Family
The H+-translocating Pyrophosphatase (H+-PPase) Family
The YaaH (YaaH) Family
The CorA Metal Ion Transporter (MIT) Family
The Folate-Biopterin Transporter (FBT) Family
The Bile Acid:Na+ Symporter (BASS) Family
The Monovalent Cation:Proton Antiporter-2 (CPA2) Family
The Ammonia Transporter Channel (Amt) Family
The Transient Receptor Potential Ca2+ Channel (TRP-CC) Family
The Voltage-gated Ion Channel (VIC) Superfamily
The Multidrug/Oligosaccharidyl-lipid/Polysaccharide (MOP) Flippase Superfamily
The Na+-transporting Carboxylic Acid Decarboxylase (NaT-DC) Family
The Chloroplast Maltose Exporter (MEX) Family
The Nucleobase:Cation Symporter-1 (NCS1) Family
The Organic Solute Transporter (OST) Family
The Polycystin Cation Channel (PCC) Family
The Presenilin ER Ca2+ Leak Channel (Presenilin) Family
The Voltage-gated Proton Channel (VPC) Family
The Low Affinity Fe2+ Transporter (FeT) Family
The Neurotransmitter Receptor, Cys loop, Ligand-gated Ion Channel (LIC) Family
The Ion-translocating Microbial Rhodopsin (MR) Family
The Resistance to Homoserine/Threonine (RhtB) Family
The Arsenical Resistance-3 (ACR3) Family
The Choline Transporter -Like (CTL) Family
The Anion Exchanger (AE) Family
The Auxin Efflux Carrier (AEC) Family
The Ferroportin (FP) Family
The Large Conductance Mechanosensitive Ion Channel (MscL) Family
The Copper Transporter (Ctr) Family
The Vacuolar Iron Transporter (VIT) Family
The Resistance-Nodulation-Cell Division (RND) Superfamily
The Tellurite-resistance/Dicarboxylate Transporter (TDT) Family
Endoplasmic Reticulum Retrieval Protein1 (Putative Heavy Metal Transporter) (Rer1) Family
The Iron/Lead Transporter (ILT) Superfamily
The Nucleobase:Cation Symporter-2 (NCS2) Family
The Mg2+ Transporter-E (MgtE) Family
The Glutamate-gated Ion Channel (GIC) Family of Neurotransmitter Receptors
The Chromate Ion Transporter (CHR) Family
The Dicarboxylate/Amino Acid:Cation (Na+ or H+) Symporter (DAACS) Family
The Formate-Nitrite Transporter (FNT) Family
The Oligopeptide Transporter (OPT) Family
The Unknown BART Superfamily-1 (UBS1) Family
The Inorganic Phosphate Transporter (PiT) Family
The K+ Uptake Permease (KUP) Family
Helicosporidium
Prototheca
Auxenochlorella
Chlorella
Coccomyxa

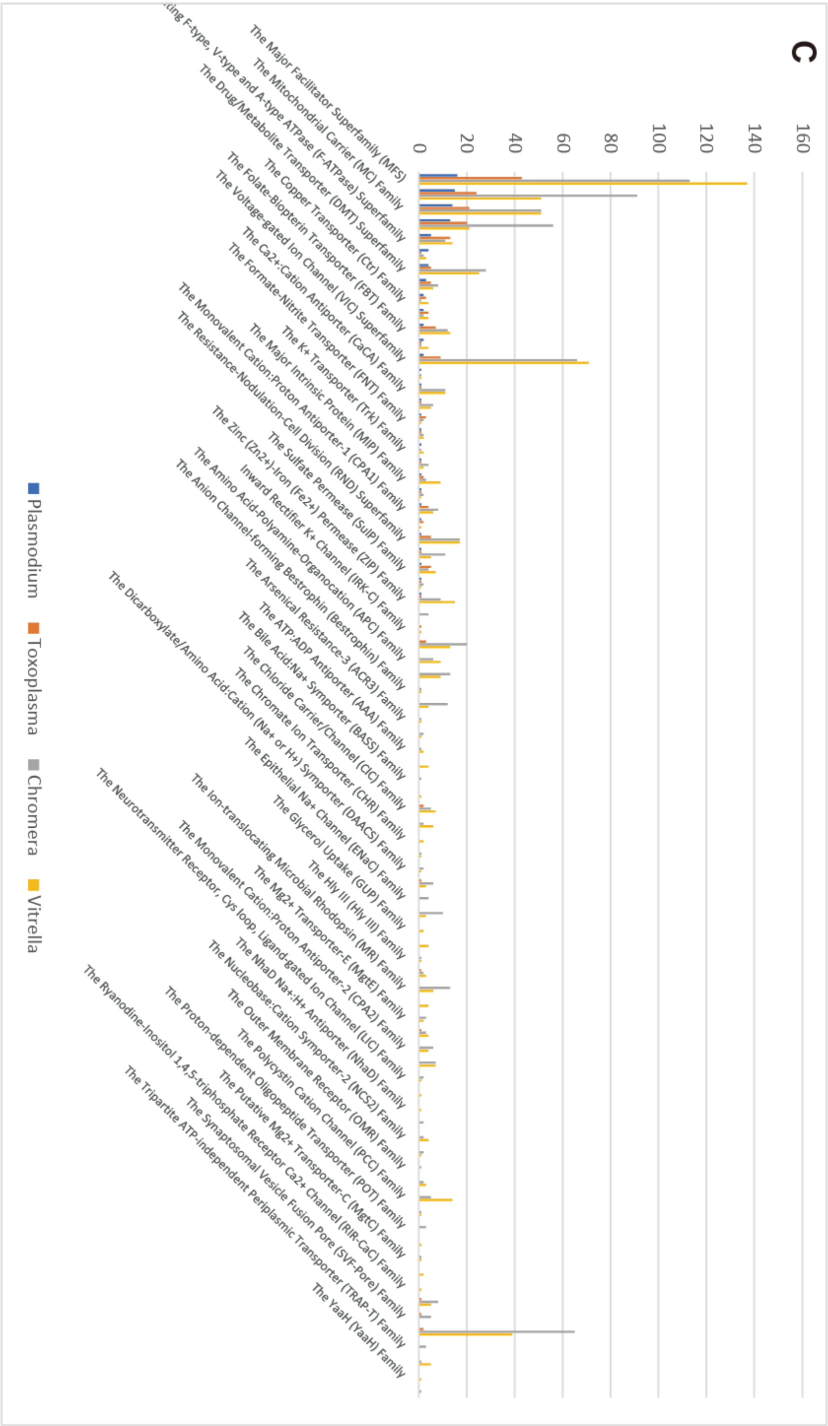

Fig. S9.

Transporter genes in the four diatoms (A), five green algae (B), and four alveolates (C). Transporters were identified by TransportTP (http://bioinfo3.noble.org/transporter/) (*66*).

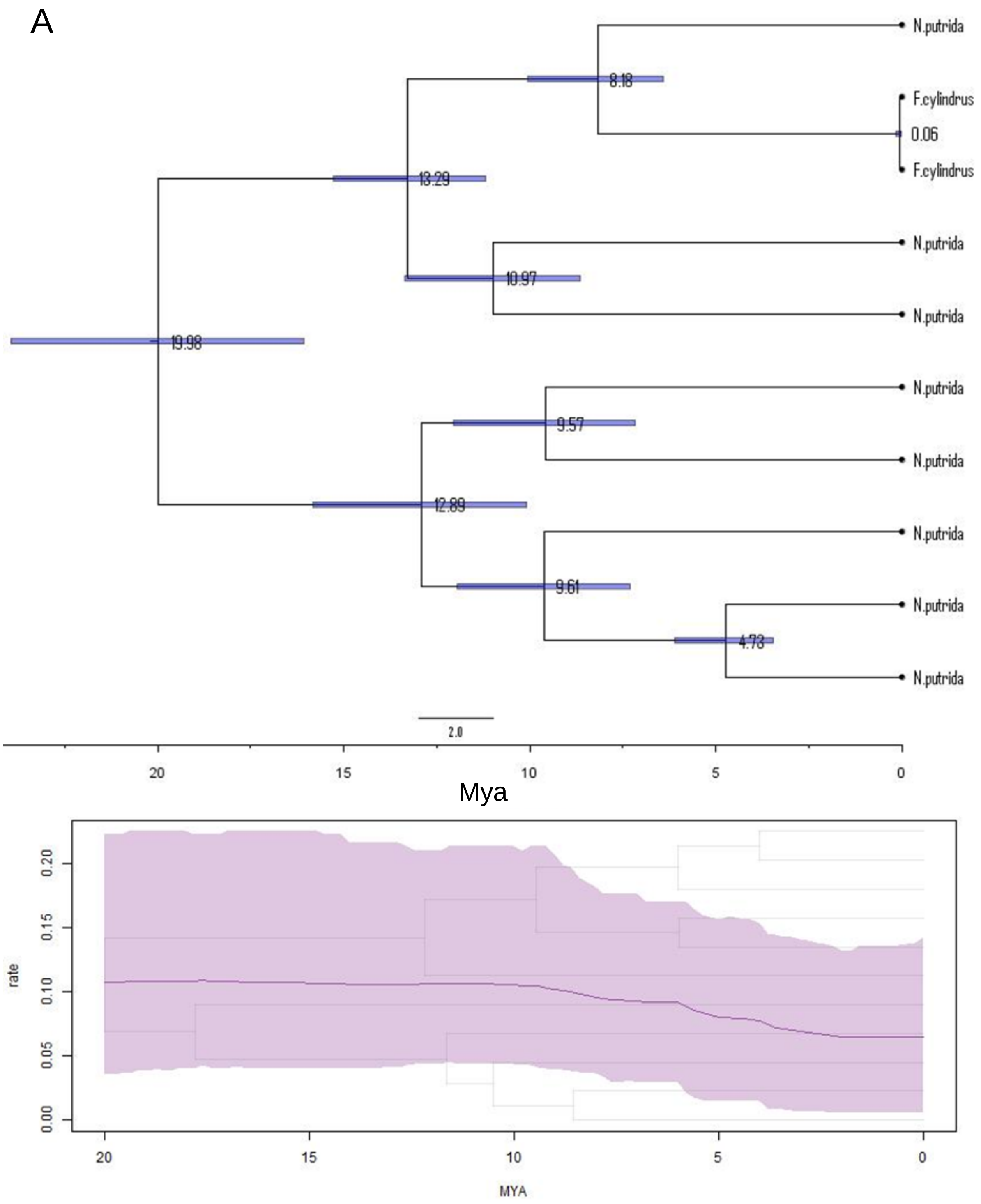
A
N.putrida
8.18
F.cylindrus
0.06
F.cylindrus
13.29
N.putrida
10.97
N.putrida
19.98
N.putrida
9.57
N.putrida
12.89
N.putrida
9.61
N.putrida
4.73
N.putrida
2.0
20 15 10 5 0
Mya
rate
0.00 0.05 0.10 0.15 0.20
20 15 10 5 0
MYA

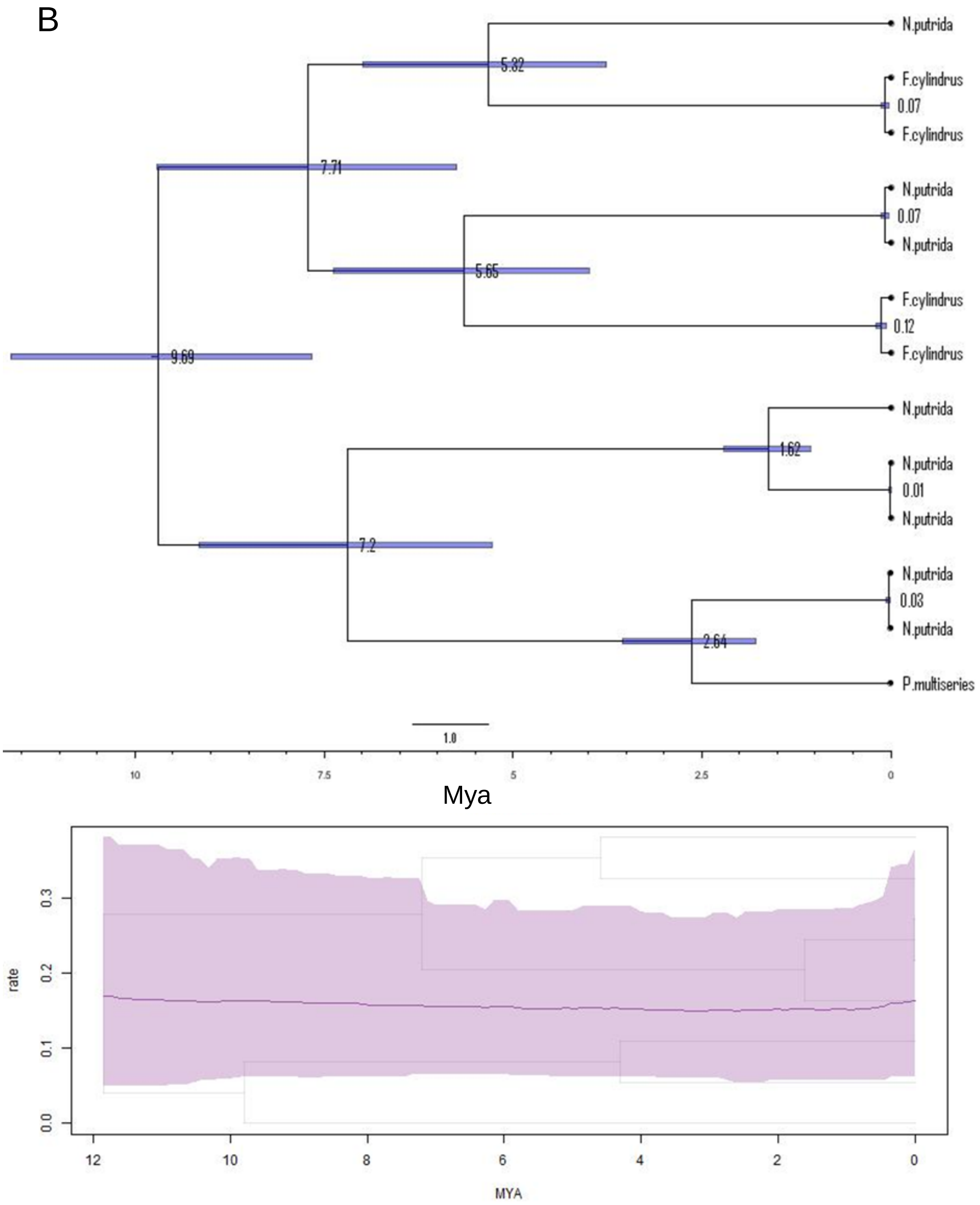
B
N.putrida
5.82
F.cylindrus
0.07
F.cylindrus
7.71
N.putrida
0.07
N.putrida
5.65
F.cylindrus
0.12
F.cylindrus
9.69
N.putrida
1.62
N.putrida
0.01
N.putrida
7.2
N.putrida
0.03
N.putrida
2.64
P.multiseries
1.0
10
7.5
5
2.5
0
Mya
rate
0.0
0.1
0.2
0.3
12
10
8
6
4
2
0
MYA

C

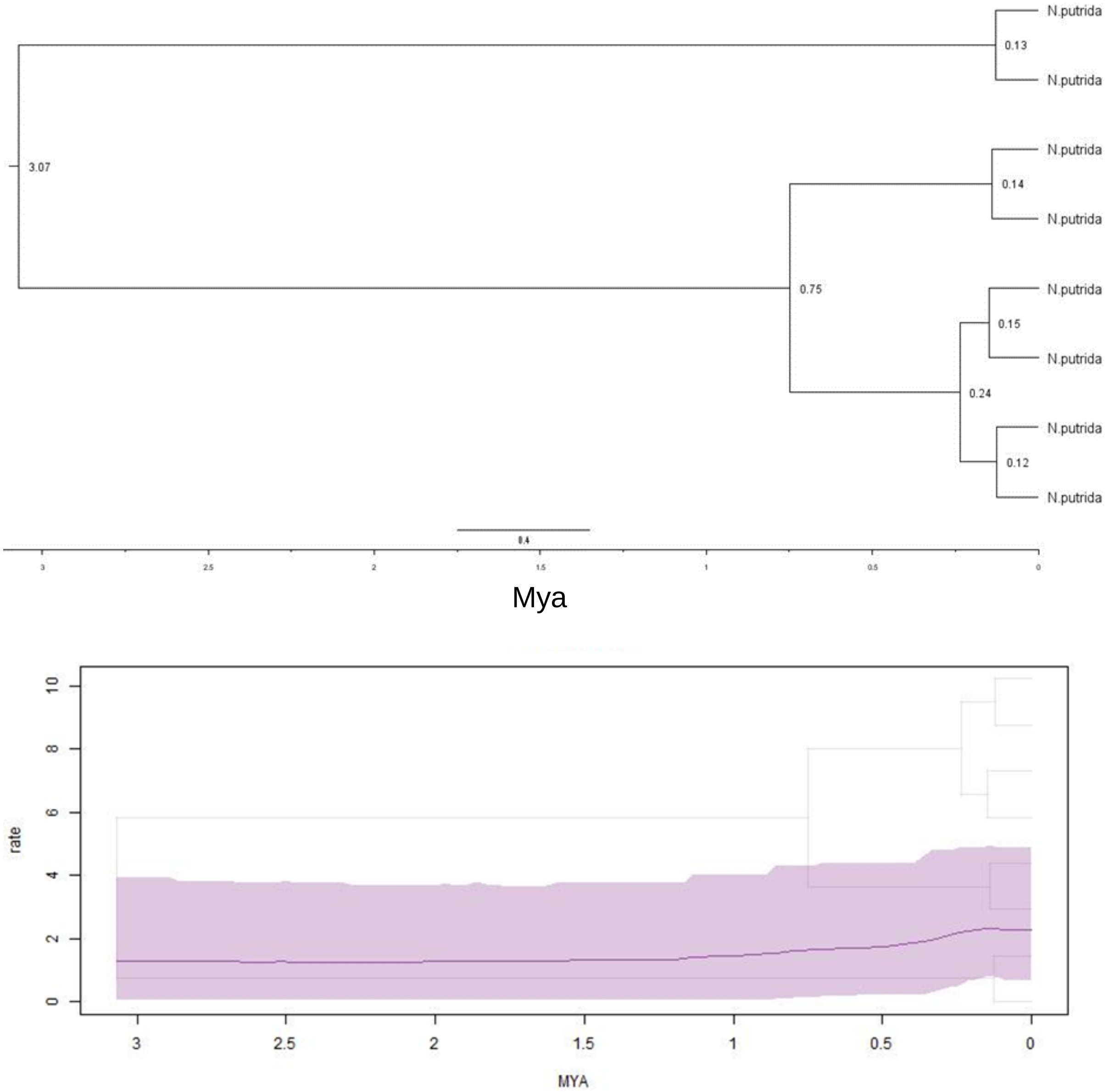

N.putrida
0.13
N.putrida
N.putrida
3.07
0.14
N.putrida
N.putrida
0.75
0.15
N.putrida
0.24
N.putrida
0.12
N.putrida
0.4
3
2.5
2
1.5
1
0.5
0
Mya
rate
10
8
6
4
2
0
3
2.5
2
1.5
1
0.5
0
MYA

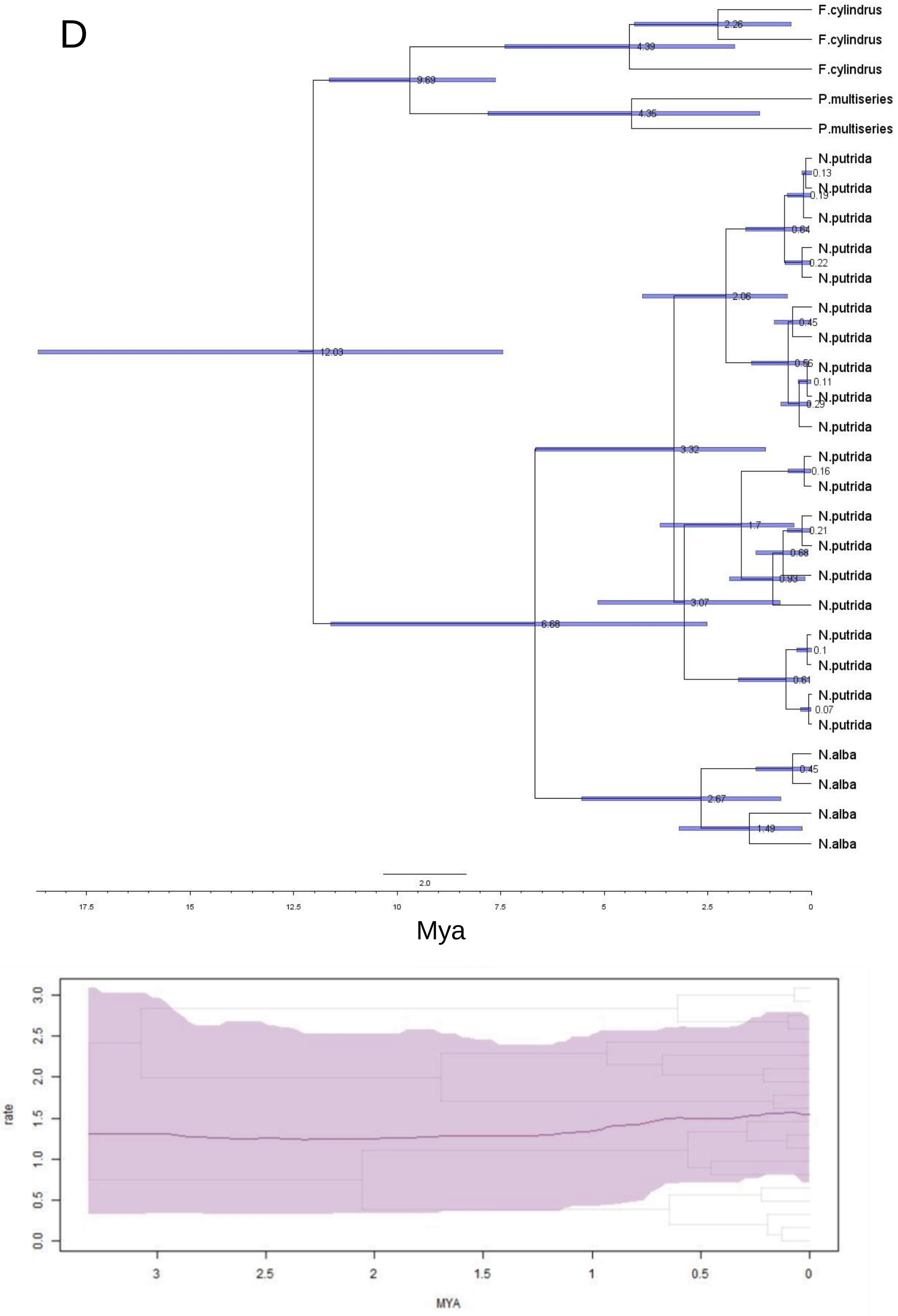

D
F.cylindrus
F.cylindrus
F.cylindrus
P.multiseries
P.multiseries
N.putrida
N.alba
2.26
4.39
9.69
4.35
12.03
0.13
0.19
0.64
0.22
2.06
0.45
0.56
0.11
0.29
3.32
0.16
1.7
0.21
0.68
0.93
3.07
6.68
0.1
0.61
0.07
0.45
2.67
1.49
2.0
17.5
15
12.5
10
7.5
5
2.5
0
Mya
rate
MYA

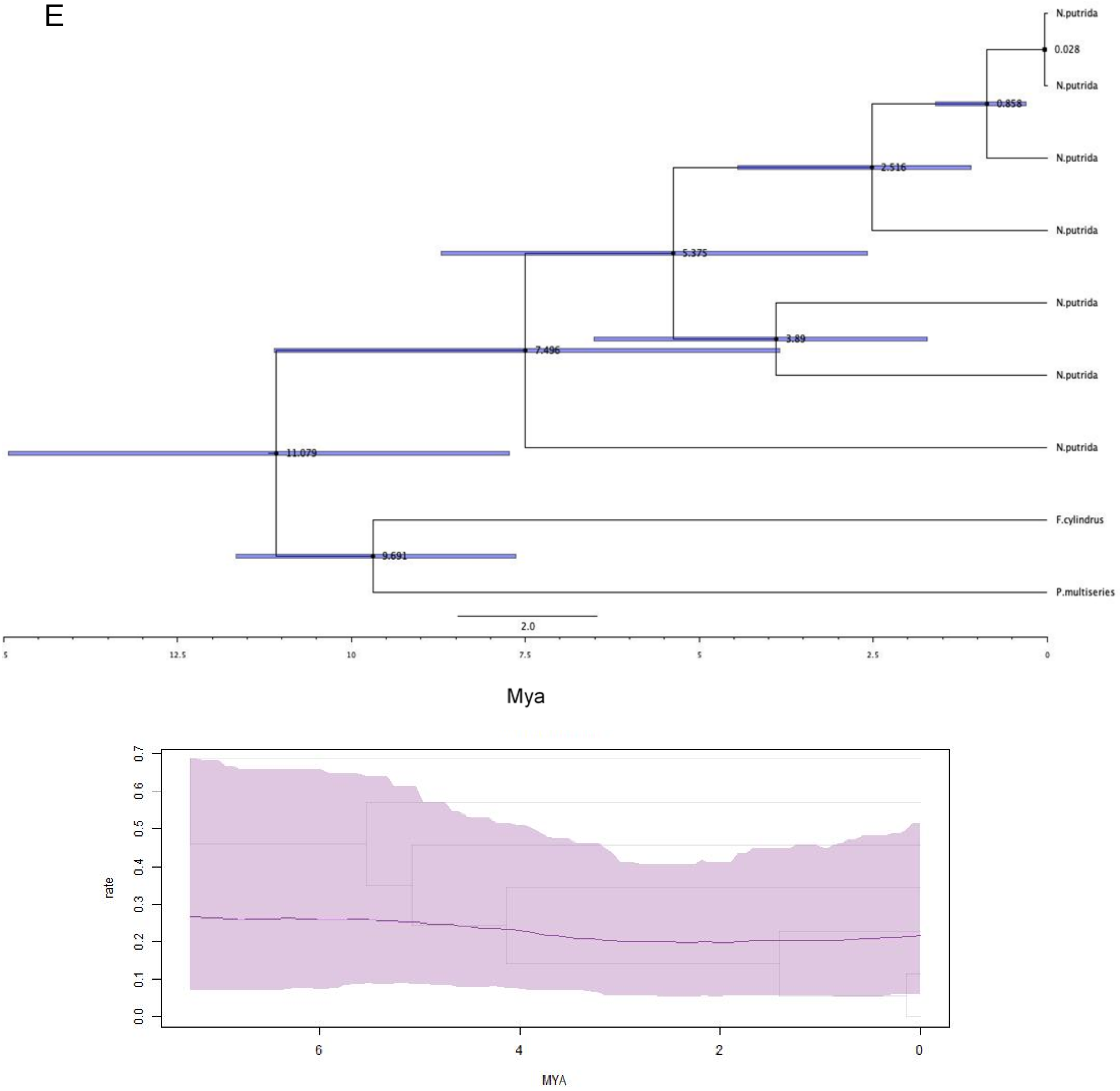
E
N.putrida
0.028
N.putrida
0.858
N.putrida
2.516
N.putrida
5.375
N.putrida
3.89
7.496
N.putrida
N.putrida
11.079
F.cylindrus
9.691
P.multiseries
2.0
5
12.5
10
7.5
5
2.5
0
Mya
rate
0.0
0.1
0.2
0.3
0.4
0.5
0.6
0.7
6
4
2
0
MYA

Fig. S10.

Expansion rates and divergence estimates of transporter gene families. Phylogenetic Maximum Clade Credibility (MCC) tree summarised by TreeAnnotator v2.6.1. Expansion rate was calculated using the Bayesian inference lineage diversification rate analysis tool TESS for R (*115*) using the MCC phylogenetic tree produced by BEAST v2.6.1 (*114*). Divergence estimates were obtained using Bayesian Markov Chain Monte Carlo (MCMC) analyses using corresponding sequences from related species implemented in Beast v2.6.1. (114). Divergence estimates for all nodes are given in Millions of years (Myr) before present. **A.** Myosin (control) for *Fragilariopsis cylindrus* (n=2) and the largest cluster of expanded Myosin gene family of *Nitzschia putrida* NIES-4235 (n=8). There is no evidence for a significant change in the expansion rate in the past 20 million years. **B.** Ammonium transporters (control) for *Pseudo-nitzschia multiseries* (n=1), *Fragilariopsis cylindrus* (n=4) and the largest cluster of expanded NH^+ gene family of *Nitzschia putrida* NIES-4235 (n=8). There is no evidence for a significant change in the expansion rate in the past 12 million years. **C.** Resistance-nodulation-cell division superfamily for the largest cluster of expanded RND gene family of *Nitzschia putrida* NIES-4235 (n=8). There is no evidence for a significant change in the expansion rate in the past 3 million years. **D.** Silicon transporters for *Fragilariopsis cylindrus* (n=3), *Pseudo-nitzschia multiseries* (n=2), *Nitzschia alba* (n=4) and the expanded SIT gene family of *Nitzschia putrida* NIES-4235 (n=20). There is no evidence for a significant change in the expansion rate in the past 3.3 million years. Divergence estimates show that *N. putrida* SIT gene family expansions occurred at around 3.3 mya (1.2 - 6.6, 95% CI), this date is later than speciation estimate between *N. putrida* and *N. alba* 6.7 mya (2.5 - 11.5, 95% CI), suggesting SIT gene family expansions were species-specific adaptions to a heterotrophic lifestyle rather than pre-adaptation. The split between *F. cylindrus* and *P. multiseries* that was used to date the tree is estimated at 9.7 mya (7.6 - 11.6, 95% CI). The mean (5-95% CI) divergence estimates for all nodes given in mya, and a scale bar is shown. *F. cylindrus*, *P. multiseries* and *N. alba* SIT gene sequences taken from published data (120). **E.** Solute:sodium symporters for *Pseudo-nitzschia multiseries* (n=1), *Fragilariopsis cylindrus* (n=1) and the largest cluster of expanded SST gene family of *Nitzschia putrida* NIES-4235 (n=7). There is no evidence for a significant change in the expansion rate in the past 7.5 million years. Divergence estimates show that *N. putrida* SST gene family expansions occurred at around 7.5 mya (3.8 - 11.1, 95% CI). The split between *F. cylindrus* and *P. multiseries* that was used to date the tree is estimated at 9.7 mya (7.6 - 11.6, 95% CI). The mean (5-95% CI) divergence estimates for all nodes given in mya, and a scale bar is shown.

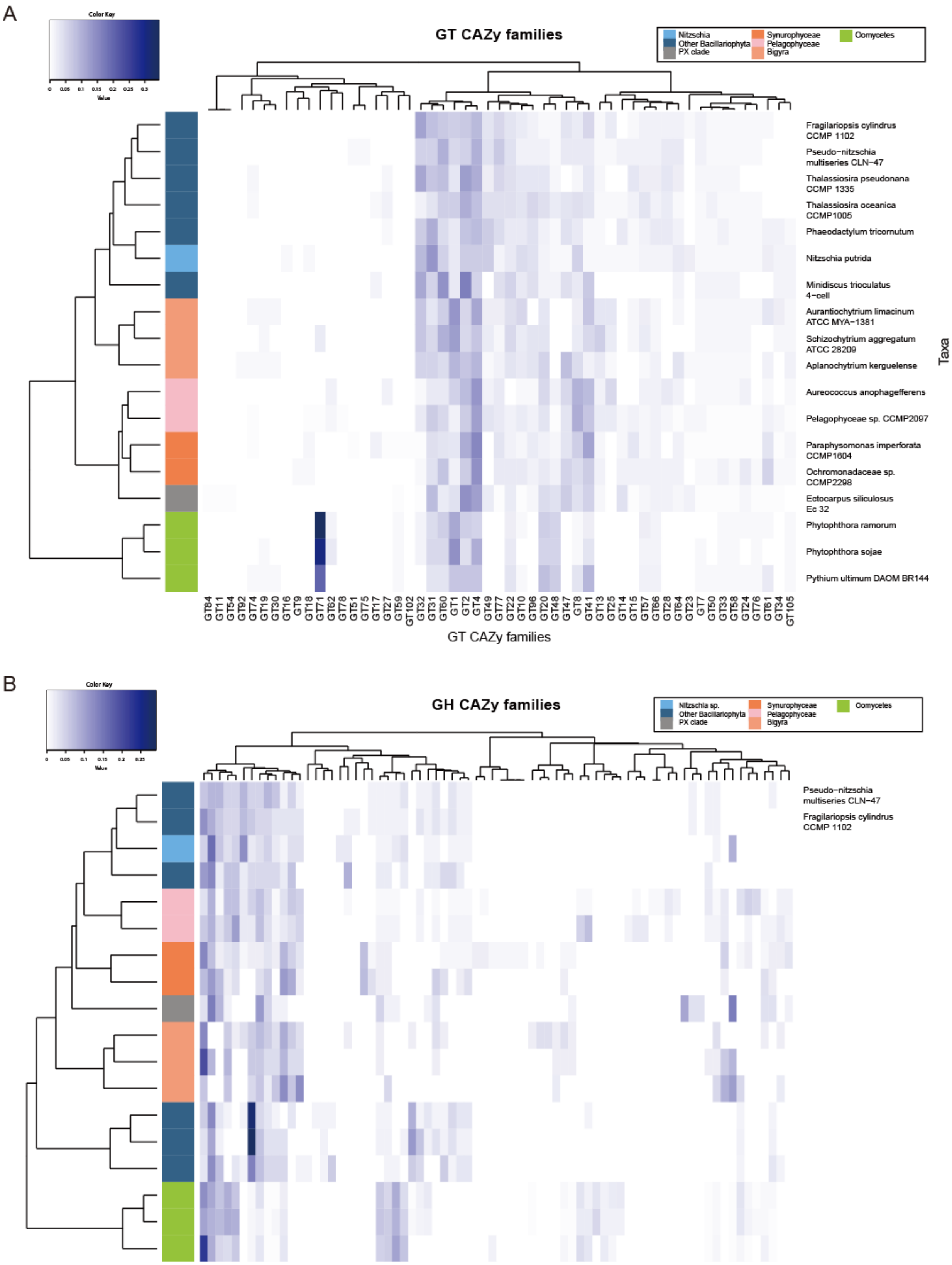
A
Color Key
Value
GT CAZy families
Nitzschia
Other Bacillariophyta
PX clade
Synurophyceae
Pelagophyceae
Bigyra
Oomycetes
Fragilariopsis cylindrus CCMP 1102
Pseudo-nitzschia multiseries CLN-47
Thalassiosira pseudonana CCMP 1335
Thalassiosira oceanica CCMP1005
Phaeodactylum tricornutum
Nitzschia putrida
Minidiscus trioculatus 4-cell
Aurantiochytrium limacinum ATCC MYA-1381
Schizochytrium aggregatum ATCC 28209
Aplanochytrium kerguelense
Aureococcus anophagefferens
Pelagophyceae sp. CCMP2097
Paraphysomonas imperforata CCMP1604
Ochromonadaceae sp. CCMP2298
Ectocarpus siliculosus Ec 32
Phytophthora ramorum
Phytophthora sojae
Pythium ultimum DAOM BR144
Taxa
GT84 GT11 GT54 GT92 GT74 GT19 GT30 GT16 GT9 GT18 GT71 GT62 GT78 GT51 GT75 GT17 GT27 GT59 GT102 GT32 GT31 GT60 GT1 GT2 GT4 GT49 GT77 GT22 GT10 GT96 GT20 GT48 GT47 GT8 GT41 GT13 GT25 GT14 GT15 GT57 GT66 GT28 GT64 GT23 GT7 GT50 GT33 GT58 GT24 GT76 GT61 GT34 GT105
GT CAZy families
B
Color Key
Value
GH CAZy families
Nitzschia sp.
Other Bacillariophyta
PX clade
Synurophyceae
Pelagophyceae
Bigyra
Oomycetes
Pseudo-nitzschia multiseries CLN-47
Fragilariopsis cylindrus CCMP 1102

Fig. S11.

Carbohydrate-active enzyme (CAZyme) in *N. putrida*. A. Glycosyltransferases (GT) families from the CAZy database classification (www.cazy.org) in various Stramenopiles. The diagram shows a heatmap of CAZyme prevalence in each taxon (number of a particular CAZy family divided by the total number of CAZymes in the organism); the white to blue color scheme indicates low to high prevalence, respectively. Dendrograms (left and top of the figure) show respectively the relative proximity of taxa with respect co-occurrence of CAZyme families and the co-occurrence of CAZyme families with one another within genomes (for details see methods described in (*71*)). *Nitzschia* is in light blue while other Bacillariophyta are in dark blue. We observe that all Bacillariophyta group together indicating a close GT repertoire. **B.** Glycoside Hydrolases (GH) families in various Stramenopiles. The diagram shows a heatmap of CAZyme prevalence in each taxon (number of a particular CAZyme family divided by the total number of CAZyme in the organism); the white to blue colour scheme indicates low to high prevalence, respectively. Dendrograms (left and top) show respectively the relative taxa proximity with respect co-occurrence of CAZyme families and the co-occurrence of CAZyme families with one another within genomes (for details see methods described in (*71*)). The same color code as in Figure 3 is used. Bacillariophyta are split into two groups indicating differences in the GH repertoire of *Nitzschia*, *F. cylindrus*, *Pseudo-nitzschia* and *P. tricornutum*, on one hand, and on the other hand *Thalassiosira* spp. and *M. trioculatus.*

A

	Nitzschia	Fragilariopsis	Phaeodactylum	Thalassiosira
total (%)	978 (6.5)	998 (5.5)	596 (5.7)	718 (6.1)
Peptide degradation	87	53	44	46
Carbohydrate metabolisms	101	89	74	52
Extracellular matrix/adhesion/cell surface associated proteins	127	42	19	23
Substrate modification	96	58	52	49
Redox	16	14	7	13
Others including unknown functions	551	742	400	535

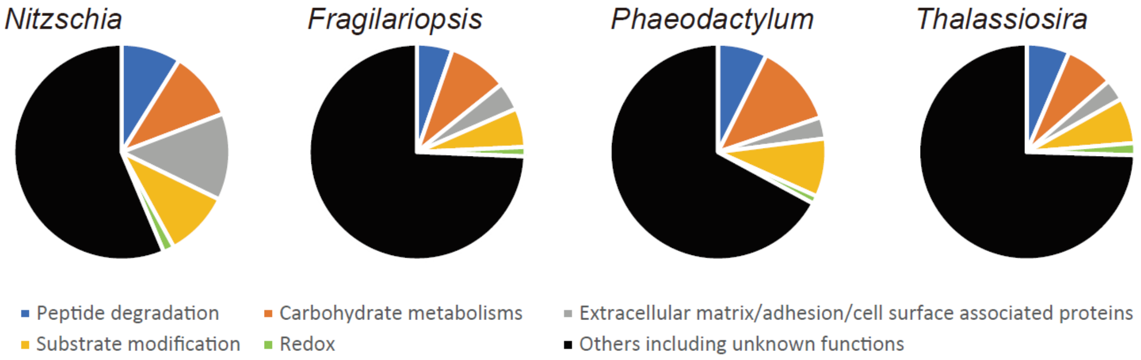

B

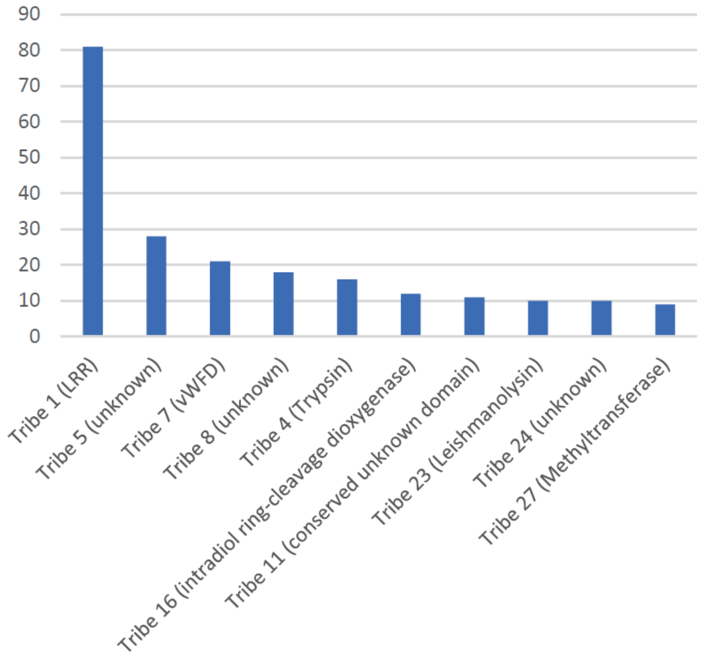

Fig. S12.

Secretomes in diatoms. A. The number of genes for predicted secreted proteins in diatoms and their annotated functions. **B.** Ten most abundant secretome sequences in *N. putrida*. Assigned protein domains or functions are described in parentheses.

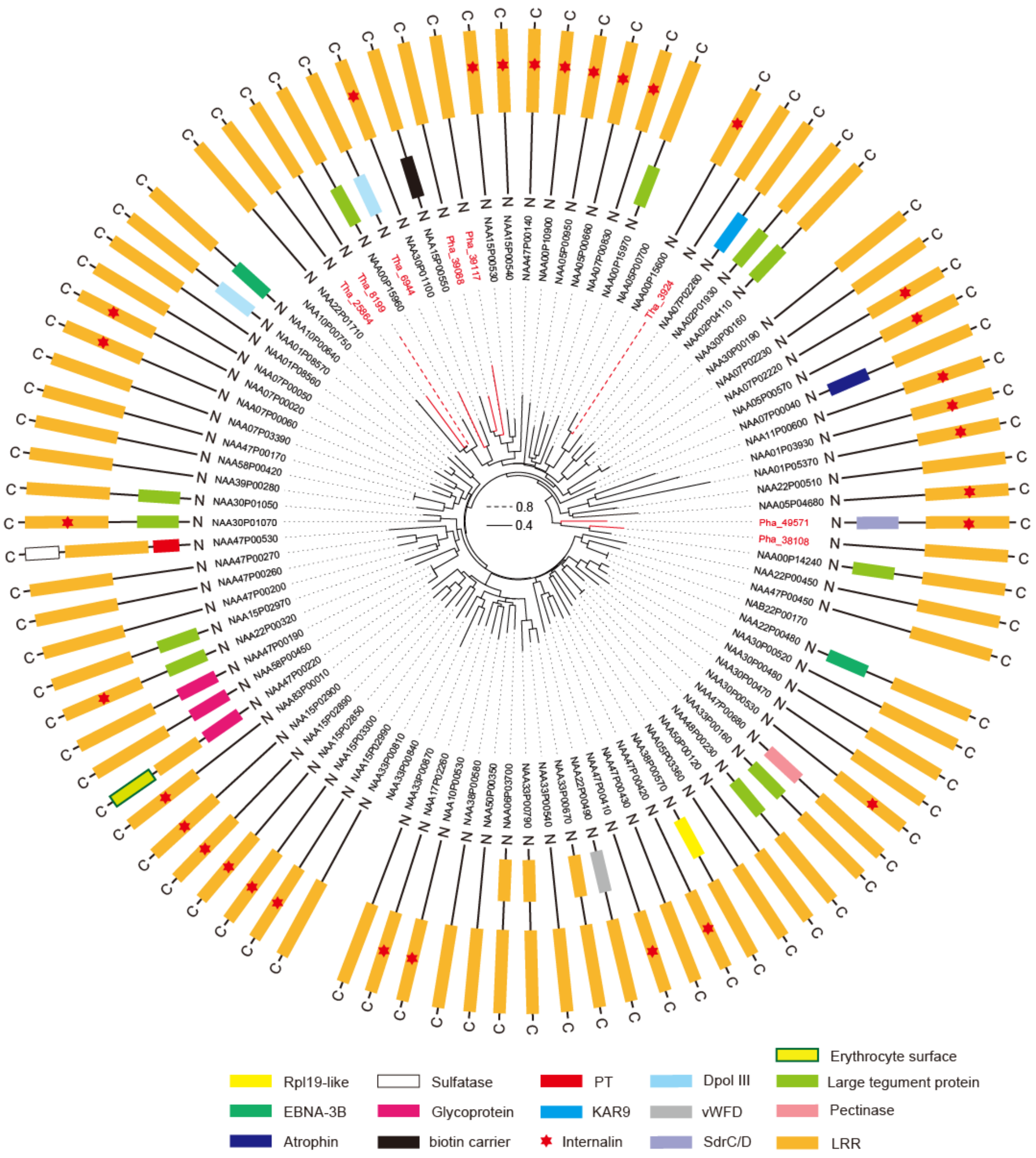

Fig. S13.

Secreted leucine rich repeat domain-containing protein diversity in diatoms. Phylogenetic tree centred in the figure was reconstructed by IQ-TREE. Domains contained in each sequence were predicted by NCBI Conserved Domain Search and depicted next to the gene IDs; in only several sequences no domain was predicted regardless of apparent homologies (e^{-30} in TribeMCL) to LRR proteins.

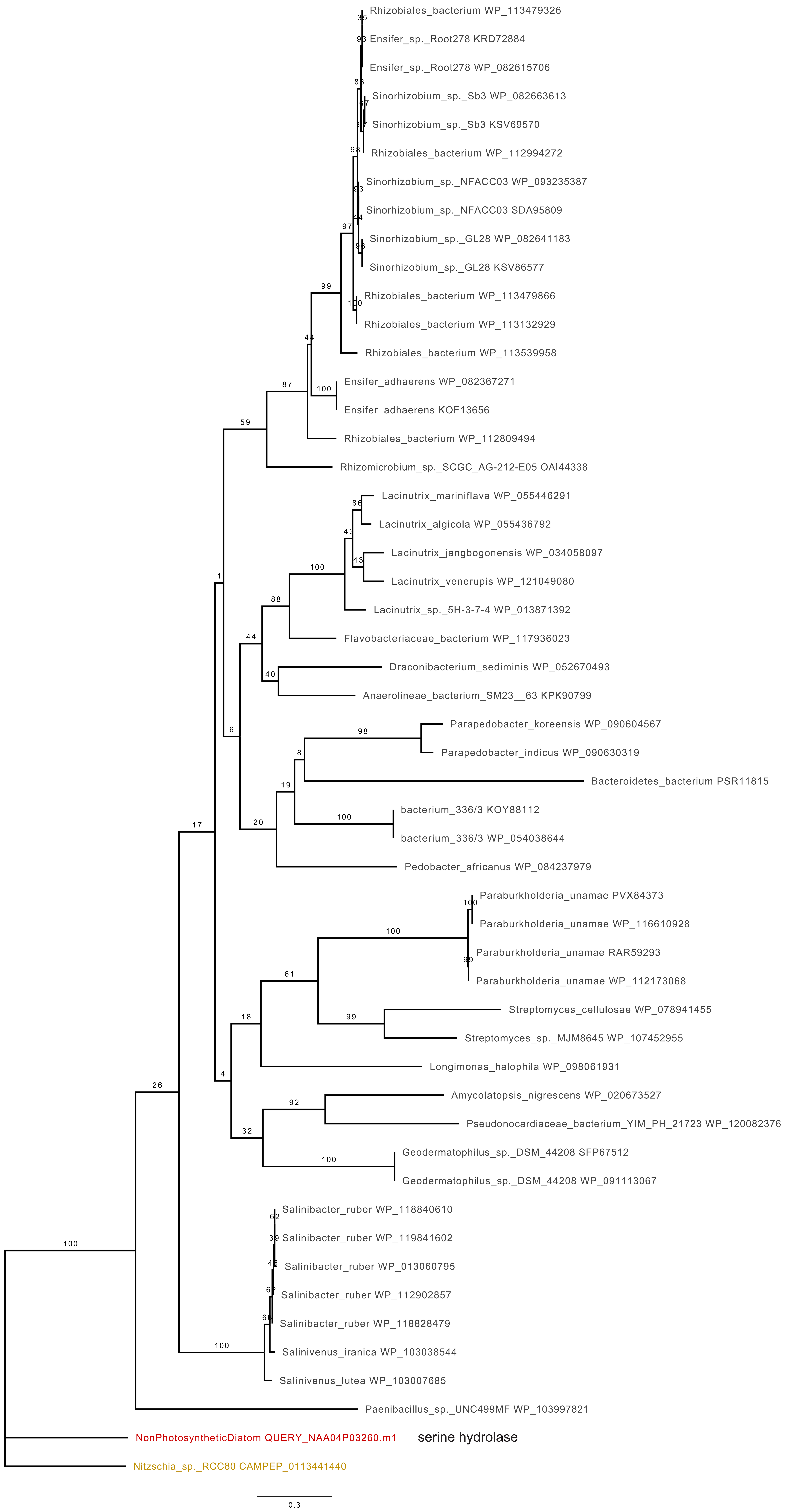
Rhizobiales_bacterium WP_113479326
Ensifer_sp._Root278 KRD72884
Ensifer_sp._Root278 WP_082615706
Sinorhizobium_sp._Sb3 WP_082663613
Sinorhizobium_sp._Sb3 KSV69570
Rhizobiales_bacterium WP_112994272
Sinorhizobium_sp._NFACC03 WP_093235387
Sinorhizobium_sp._NFACC03 SDA95809
Sinorhizobium_sp._GL28 WP_082641183
Sinorhizobium_sp._GL28 KSV86577
Rhizobiales_bacterium WP_113479866
Rhizobiales_bacterium WP_113132929
Rhizobiales_bacterium WP_113539958
Ensifer_adhaerens WP_082367271
Ensifer_adhaerens KOF13656
Rhizobiales_bacterium WP_112809494
Rhizomicrobium_sp._SCGC_AG-212-E05 OAI44338
Lacinutrix_mariniflava WP_055446291
Lacinutrix_algicola WP_055436792
Lacinutrix_jangbogonensis WP_034058097
Lacinutrix_venerupis WP_121049080
Lacinutrix_sp._5H-3-7-4 WP_013871392
Flavobacteriaceae_bacterium WP_117936023
Draconibacterium_sediminis WP_052670493
Anaerolineae_bacterium_SM23__63 KPK90799
Parapedobacter_koreensis WP_090604567
Parapedobacter_indicus WP_090630319
Bacteroidetes_bacterium PSR11815
bacterium_336/3 KOY88112
bacterium_336/3 WP_054038644
Pedobacter_africanus WP_084237979
Paraburkholderia_unamae PVX84373
Paraburkholderia_unamae WP_116610928
Paraburkholderia_unamae RAR59293
Paraburkholderia_unamae WP_112173068
Streptomyces_cellulosae WP_078941455
Streptomyces_sp._MJM8645 WP_107452955
Longimonas_halophila WP_098061931
Amycolatopsis_nigrescens WP_020673527
Pseudonocardiaceae_bacterium_YIM_PH_21723 WP_120082376
Geodermatophilus_sp._DSM_44208 SFP67512
Geodermatophilus_sp._DSM_44208 WP_091113067
Salinibacter_ruber WP_118840610
Salinibacter_ruber WP_119841602
Salinibacter_ruber WP_013060795
Salinibacter_ruber WP_112902857
Salinibacter_ruber WP_118828479
Salinivenus_iranica WP_103038544
Salinivenus_lutea WP_103007685
Paenibacillus_sp._UNC499MF WP_103997821
NonPhotosyntheticDiatom QUERY_NAA04P03260.m1
serine hydrolase
Nitzschia_sp._RCC80 CAMPEP_0113441440
0.3

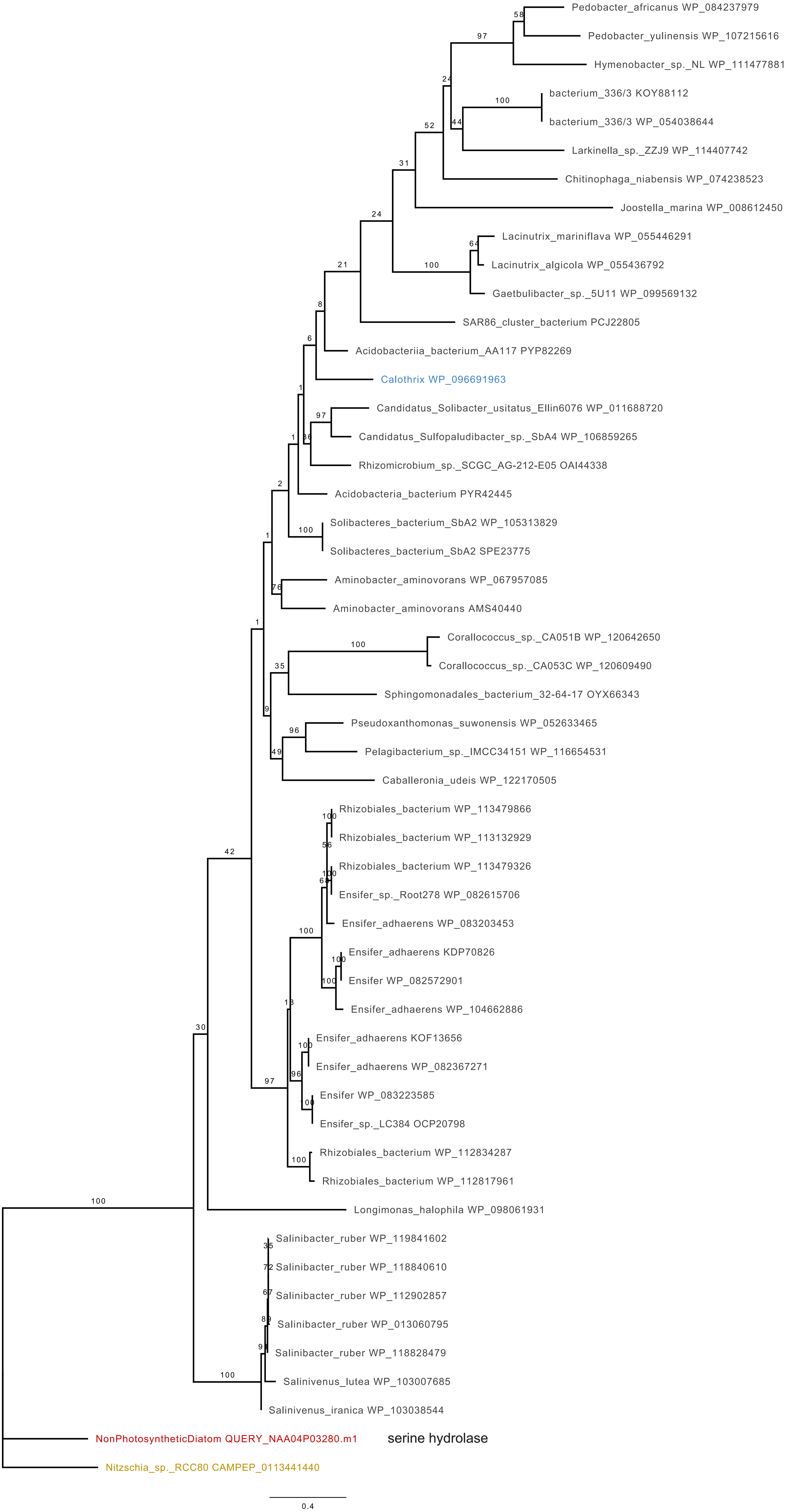
Pedobacter_africanus WP_084237979
Pedobacter_yulinensis WP_107215616
Hymenobacter_sp._NL WP_111477881
bacterium_336/3 KOY88112
bacterium_336/3 WP_054038644
Larkinella_sp._ZZJ9 WP_114407742
Chitinophaga_niabensis WP_074238523
Joostella_marina WP_008612450
Lacinutrix_mariniflava WP_055446291
Lacinutrix_algicola WP_055436792
Gaetbulibacter_sp._5U11 WP_099569132
SAR86_cluster_bacterium PCJ22805
Acidobacteriia_bacterium_AA117 PYP82269
Calothrix WP_096691963
Candidatus_Solibacter_usitatus_Ellin6076 WP_011688720
Candidatus_Sulfopaludibacter_sp._SbA4 WP_106859265
Rhizomicrobium_sp._SCGC_AG-212-E05 OAI44338
Acidobacteria_bacterium PYR42445
Solibacteres_bacterium_SbA2 WP_105313829
Solibacteres_bacterium_SbA2 SPE23775
Aminobacter_aminovorans WP_067957085
Aminobacter_aminovorans AMS40440
Corallococcus_sp._CA051B WP_120642650
Corallococcus_sp._CA053C WP_120609490
Sphingomonadales_bacterium_32-64-17 OYX66343
Pseudoxanthomonas_suwonensis WP_052633465
Pelagibacterium_sp._IMCC34151 WP_116654531
Caballeronia_udeis WP_122170505
Rhizobiales_bacterium WP_113479866
Rhizobiales_bacterium WP_113132929
Rhizobiales_bacterium WP_113479326
Ensifer_sp._Root278 WP_082615706
Ensifer_adhaerens WP_083203453
Ensifer_adhaerens KDP70826
Ensifer WP_082572901
Ensifer_adhaerens WP_104662886
Ensifer_adhaerens KOF13656
Ensifer_adhaerens WP_082367271
Ensifer WP_083223585
Ensifer_sp._LC384 OCP20798
Rhizobiales_bacterium WP_112834287
Rhizobiales_bacterium WP_112817961
Longimonas_halophila WP_098061931
Salinibacter_ruber WP_119841602
Salinibacter_ruber WP_118840610
Salinibacter_ruber WP_112902857
Salinibacter_ruber WP_013060795
Salinibacter_ruber WP_118828479
Salinivenus_lutea WP_103007685
Salinivenus_iranica WP_103038544
NonPhotosyntheticDiatom QUERY_NAA04P03280.m1
serine hydrolase
Nitzschia_sp._RCC80 CAMPEP_0113441440
0.4

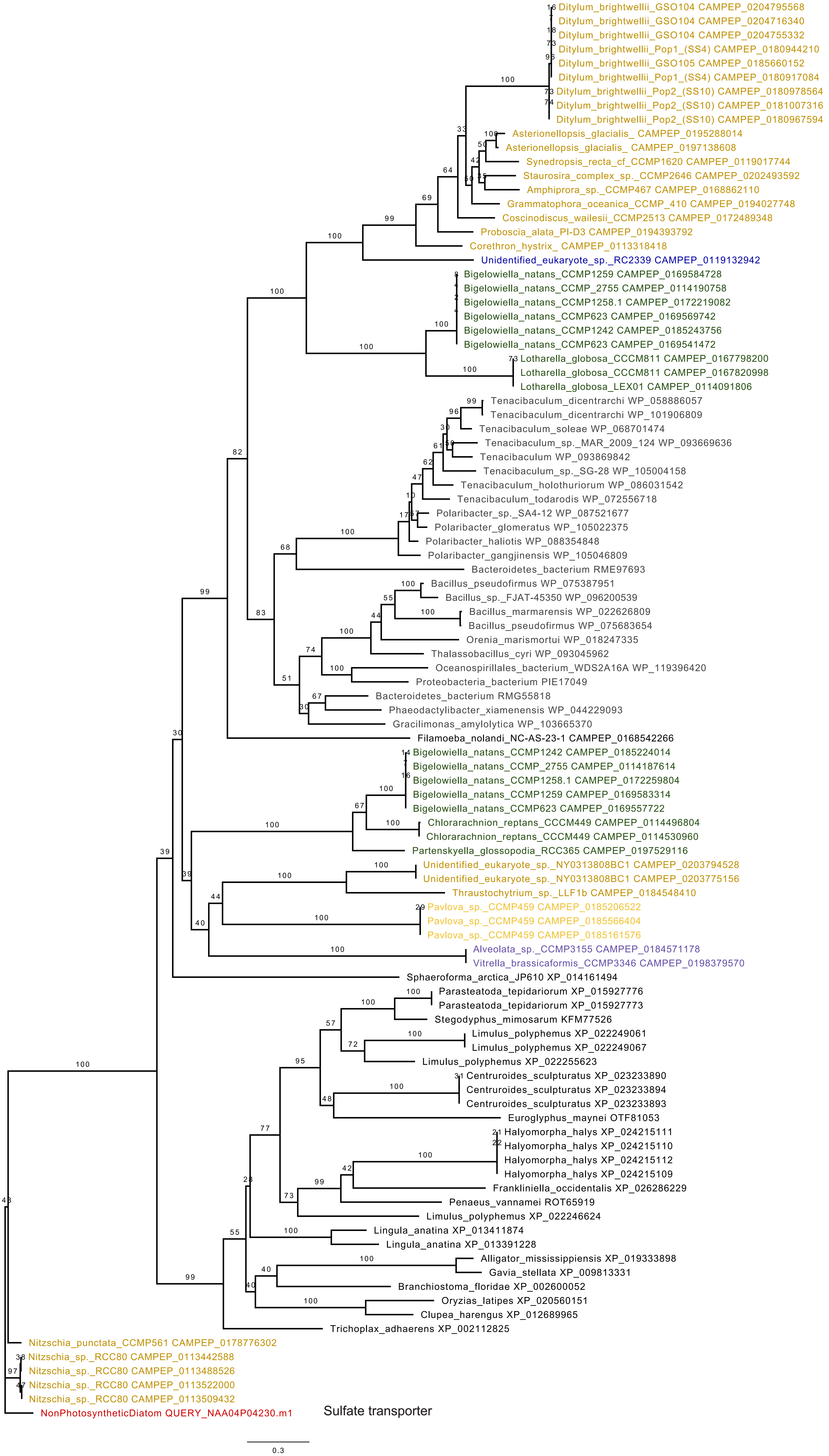
Ditylum_brightwellii_GSO104 CAMPEP_0204795568
Ditylum_brightwellii_GSO104 CAMPEP_0204716340
Ditylum_brightwellii_GSO104 CAMPEP_0204755332
Ditylum_brightwellii_Pop1_(SS4) CAMPEP_0180944210
Ditylum_brightwellii_GSO105 CAMPEP_0185660152
Ditylum_brightwellii_Pop1_(SS4) CAMPEP_0180917084
Ditylum_brightwellii_Pop2_(SS10) CAMPEP_0180978564
Ditylum_brightwellii_Pop2_(SS10) CAMPEP_0181007316
Ditylum_brightwellii_Pop2_(SS10) CAMPEP_0180967594
Asterionellopsis_glacialis_ CAMPEP_0195288014
Asterionellopsis_glacialis_ CAMPEP_0197138608
Synedropsis_recta_cf_CCMP1620 CAMPEP_0119017744
Staurosira_complex_sp._CCMP2646 CAMPEP_0202493592
Amphiprora_sp._CCMP467 CAMPEP_0168862110
Grammatophora_oceanica_CCMP_410 CAMPEP_0194027748
Coscinodiscus_wailesii_CCMP2513 CAMPEP_0172489348
Proboscia_alata_PI-D3 CAMPEP_0194393792
Corethron_hystrix_ CAMPEP_0113318418
Unidentified_eukaryote_sp._RC2339 CAMPEP_0119132942
Bigelowiella_natans_CCMP1259 CAMPEP_0169584728
Bigelowiella_natans_CCMP_2755 CAMPEP_0114190758
Bigelowiella_natans_CCMP1258.1 CAMPEP_0172219082
Bigelowiella_natans_CCMP623 CAMPEP_0169569742
Bigelowiella_natans_CCMP1242 CAMPEP_0185243756
Bigelowiella_natans_CCMP623 CAMPEP_0169541472
Lotharella_globosa_CCCM811 CAMPEP_0167798200
Lotharella_globosa_CCCM811 CAMPEP_0167820998
Lotharella_globosa_LEX01 CAMPEP_0114091806
Tenacibaculum_dicentrarchi WP_058886057
Tenacibaculum_dicentrarchi WP_101906809
Tenacibaculum_soleae WP_068701474
Tenacibaculum_sp._MAR_2009_124 WP_093669636
Tenacibaculum WP_093869842
Tenacibaculum_sp._SG-28 WP_105004158
Tenacibaculum_holothuriorum WP_086031542
Tenacibaculum_todarodis WP_072556718
Polaribacter_sp._SA4-12 WP_087521677
Polaribacter_glomeratus WP_105022375
Polaribacter_haliotis WP_088354848
Polaribacter_gangjinensis WP_105046809
Bacteroidetes_bacterium RME97693
Bacillus_pseudofirmus WP_075387951
Bacillus_sp._FJAT-45350 WP_096200539
Bacillus_marmarensis WP_022626809
Bacillus_pseudofirmus WP_075683654
Orenia_marismortui WP_018247335
Thalassobacillus_cyri WP_093045962
Oceanospirillales_bacterium_WDS2A16A WP_119396420
Proteobacteria_bacterium PIE17049
Bacteroidetes_bacterium RMG55818
Phaeodactylibacter_xiamenensis WP_044229093
Gracilimonas_amylolytica WP_103665370
Filamoeba_nolandi_NC-AS-23-1 CAMPEP_0168542266
Bigelowiella_natans_CCMP1242 CAMPEP_0185224014
Bigelowiella_natans_CCMP_2755 CAMPEP_0114187614
Bigelowiella_natans_CCMP1258.1 CAMPEP_0172259804
Bigelowiella_natans_CCMP1259 CAMPEP_0169583314
Bigelowiella_natans_CCMP623 CAMPEP_0169557722
Chlorarachnion_reptans_CCCM449 CAMPEP_0114496804
Chlorarachnion_reptans_CCCM449 CAMPEP_0114530960
Partenskyella_glossopodia_RCC365 CAMPEP_0197529116
Unidentified_eukaryote_sp._NY0313808BC1 CAMPEP_0203794528
Unidentified_eukaryote_sp._NY0313808BC1 CAMPEP_0203775156
Thraustochytrium_sp._LLF1b CAMPEP_0184548410
Pavlova_sp._CCMP459 CAMPEP_0185206522
Pavlova_sp._CCMP459 CAMPEP_0185566404
Pavlova_sp._CCMP459 CAMPEP_0185161576
Alveolata_sp._CCMP3155 CAMPEP_0184571178
Vitrella_brassicaformis_CCMP3346 CAMPEP_0198379570
Sphaeroforma_arctica_JP610 XP_014161494
Parasteatoda_tepidariorum XP_015927776
Parasteatoda_tepidariorum XP_015927773
Stegodyphus_mimosarum KFM77526
Limulus_polyphemus XP_022249061
Limulus_polyphemus XP_022249067
Limulus_polyphemus XP_022255623
Centruroides_sculpturatus XP_023233890
Centruroides_sculpturatus XP_023233894
Centruroides_sculpturatus XP_023233893
Euroglyphus_maynei OTF81053
Halyomorpha_halys XP_024215111
Halyomorpha_halys XP_024215110
Halyomorpha_halys XP_024215112
Halyomorpha_halys XP_024215109
Frankliniella_occidentalis XP_026286229
Penaeus_vannamei ROT65919
Limulus_polyphemus XP_022246624
Lingula_anatina XP_013411874
Lingula_anatina XP_013391228
Alligator_mississippiensis XP_019333898
Gavia_stellata XP_009813331
Branchiostoma_floridae XP_002600052
Oryzias_latipes XP_020560151
Clupea_harengus XP_012689965
Trichoplax_adhaerens XP_002112825
Nitzschia_punctata_CCMP561 CAMPEP_0178776302
Nitzschia_sp._RCC80 CAMPEP_0113442588
Nitzschia_sp._RCC80 CAMPEP_0113488526
Nitzschia_sp._RCC80 CAMPEP_0113522000
Nitzschia_sp._RCC80 CAMPEP_0113509432
NonPhotosyntheticDiatom QUERY_NAA04P04230.m1
Sulfate transporter
0.3

Fig. S14.

Phylogeny of three laterally transferred genes exclusively shared in the genus *Nitzschia*. Numbers on branches are maximum likelihood bootstrap values. Highlighted in red are sequences of *N. putrida*.

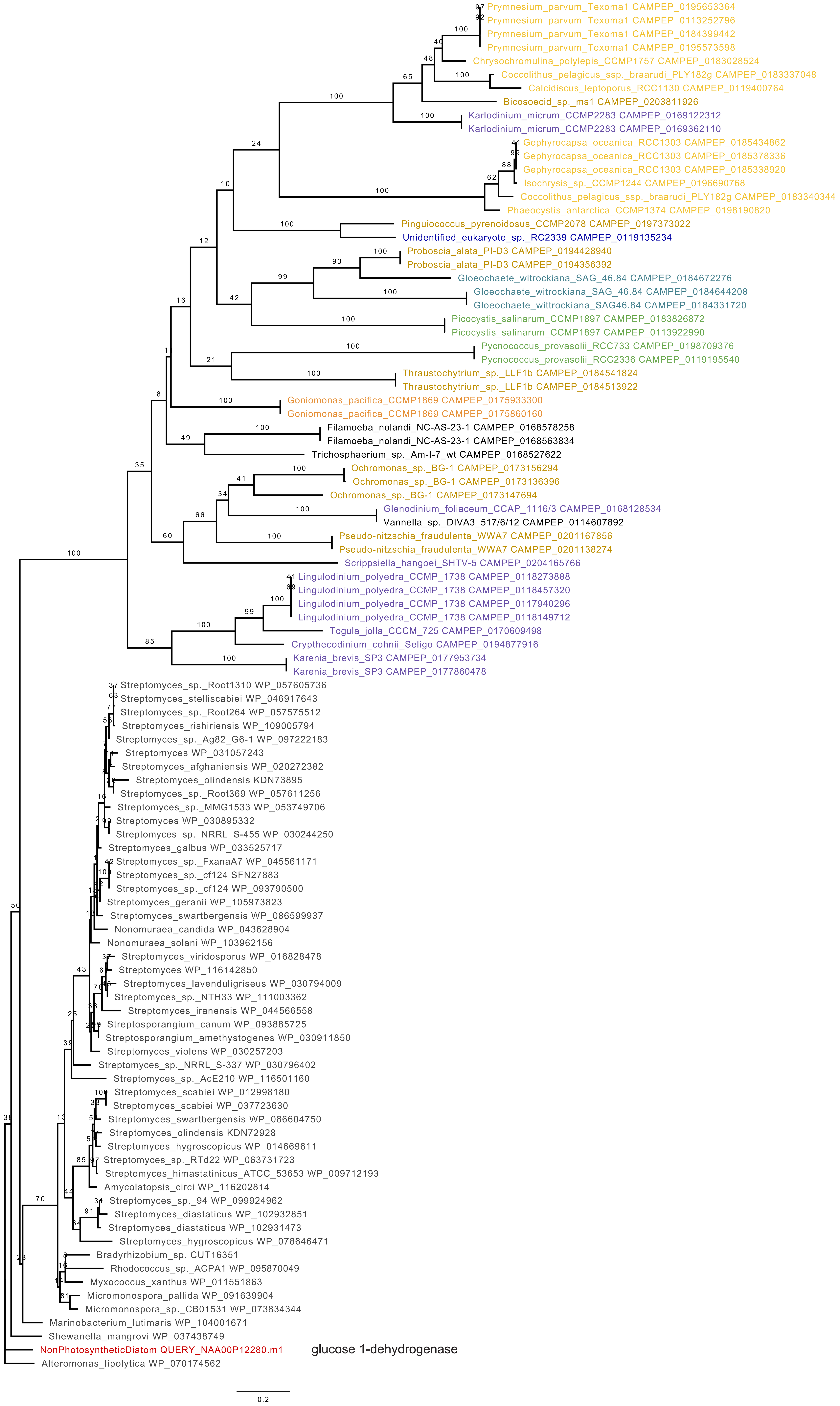
Prymnesium_parvum_Texoma1 CAMPEP_0195653364
Prymnesium_parvum_Texoma1 CAMPEP_0113252796
Prymnesium_parvum_Texoma1 CAMPEP_0184399442
Prymnesium_parvum_Texoma1 CAMPEP_0195573598
Chrysochromulina_polylepis_CCMP1757 CAMPEP_0183028524
Coccolithus_pelagicus_ssp._braarudi_PLY182g CAMPEP_0183337048
Calcidiscus_leptoporus_RCC1130 CAMPEP_0119400764
Bicosoecid_sp._ms1 CAMPEP_0203811926
Karlodinium_micrum_CCMP2283 CAMPEP_0169122312
Karlodinium_micrum_CCMP2283 CAMPEP_0169362110
Gephyrocapsa_oceanica_RCC1303 CAMPEP_0185434862
Gephyrocapsa_oceanica_RCC1303 CAMPEP_0185378336
Gephyrocapsa_oceanica_RCC1303 CAMPEP_0185338920
Isochrysis_sp._CCMP1244 CAMPEP_0196690768
Coccolithus_pelagicus_ssp._braarudi_PLY182g CAMPEP_0183340344
Phaeocystis_antarctica_CCMP1374 CAMPEP_0198190820
Pinguiococcus_pyrenoidosus_CCMP2078 CAMPEP_0197373022
Unidentified_eukaryote_sp._RC2339 CAMPEP_0119135234
Proboscia_alata_PI-D3 CAMPEP_0194428940
Proboscia_alata_PI-D3 CAMPEP_0194356392
Gloeochaete_witrockiana_SAG_46.84 CAMPEP_0184672276
Gloeochaete_witrockiana_SAG_46.84 CAMPEP_0184644208
Gloeochaete_wittrockiana_SAG46.84 CAMPEP_0184331720
Picocystis_salinarum_CCMP1897 CAMPEP_0183826872
Picocystis_salinarum_CCMP1897 CAMPEP_0113922990
Pycnococcus_provasolii_RCC733 CAMPEP_0198709376
Pycnococcus_provasolii_RCC2336 CAMPEP_0119195540
Thraustochytrium_sp._LLF1b CAMPEP_0184541824
Thraustochytrium_sp._LLF1b CAMPEP_0184513922
Goniomonas_pacifica_CCMP1869 CAMPEP_0175933300
Goniomonas_pacifica_CCMP1869 CAMPEP_0175860160
Filamoeba_nolandi_NC-AS-23-1 CAMPEP_0168578258
Filamoeba_nolandi_NC-AS-23-1 CAMPEP_0168563834
Trichosphaerium_sp._Am-I-7_wt CAMPEP_0168527622
Ochromonas_sp._BG-1 CAMPEP_0173156294
Ochromonas_sp._BG-1 CAMPEP_0173136396
Ochromonas_sp._BG-1 CAMPEP_0173147694
Glenodinium_foliaceum_CCAP_1116/3 CAMPEP_0168128534
Vannella_sp._DIVA3_517/6/12 CAMPEP_0114607892
Pseudo-nitzschia_fraudulenta_WWA7 CAMPEP_0201167856
Pseudo-nitzschia_fraudulenta_WWA7 CAMPEP_0201138274
Scrippsiella_hangoei_SHTV-5 CAMPEP_0204165766
Lingulodinium_polyedra_CCMP_1738 CAMPEP_0118273888
Lingulodinium_polyedra_CCMP_1738 CAMPEP_0118457320
Lingulodinium_polyedra_CCMP_1738 CAMPEP_0117940296
Lingulodinium_polyedra_CCMP_1738 CAMPEP_0118149712
Togula_jolla_CCCM_725 CAMPEP_0170609498
Crypthecodinium_cohnii_Seligo CAMPEP_0194877916
Karenia_brevis_SP3 CAMPEP_0177953734
Karenia_brevis_SP3 CAMPEP_0177860478
Streptomyces_sp._Root1310 WP_057605736
Streptomyces_stelliscabiei WP_046917643
Streptomyces_sp._Root264 WP_057575512
Streptomyces_rishiriensis WP_109005794
Streptomyces_sp._Ag82_G6-1 WP_097222183
Streptomyces WP_031057243
Streptomyces_afghaniensis WP_020272382
Streptomyces_olindensis KDN73895
Streptomyces_sp._Root369 WP_057611256
Streptomyces_sp._MMG1533 WP_053749706
Streptomyces WP_030895332
Streptomyces_sp._NRRL_S-455 WP_030244250
Streptomyces_galbus WP_033525717
Streptomyces_sp._FxanaA7 WP_045561171
Streptomyces_sp._cf124 SFN27883
Streptomyces_sp._cf124 WP_093790500
Streptomyces_geranii WP_105973823
Streptomyces_swartbergensis WP_086599937
Nonomuraea_candida WP_043628904
Nonomuraea_solani WP_103962156
Streptomyces_viridosporus WP_016828478
Streptomyces WP_116142850
Streptomyces_lavenduligriseus WP_030794009
Streptomyces_sp._NTH33 WP_111003362
Streptomyces_iranensis WP_044566558
Streptosporangium_canum WP_093885725
Streptosporangium_amethystogenes WP_030911850
Streptomyces_violens WP_030257203
Streptomyces_sp._NRRL_S-337 WP_030796402
Streptomyces_sp._AcE210 WP_116501160
Streptomyces_scabiei WP_012998180
Streptomyces_scabiei WP_037723630
Streptomyces_swartbergensis WP_086604750
Streptomyces_olindensis KDN72928
Streptomyces_hygroscopicus WP_014669611
Streptomyces_sp._RTd22 WP_063731723
Streptomyces_himastatinicus_ATCC_53653 WP_009712193
Amycolatopsis_circi WP_116202814
Streptomyces_sp._94 WP_099924962
Streptomyces_diastaticus WP_102932851
Streptomyces_diastaticus WP_102931473
Streptomyces_hygroscopicus WP_078646471
Bradyrhizobium_sp. CUT16351
Rhodococcus_sp._ACPA1 WP_095870049
Myxococcus_xanthus WP_011551863
Micromonospora_pallida WP_091639904
Micromonospora_sp._CB01531 WP_073834344
Marinobacterium_lutimaris WP_104001671
Shewanella_mangrovi WP_037438749
NonPhotosyntheticDiatom QUERY_NAA00P12280.m1
Alteromonas_lipolytica WP_070174562
glucose 1-dehydrogenase
0.2

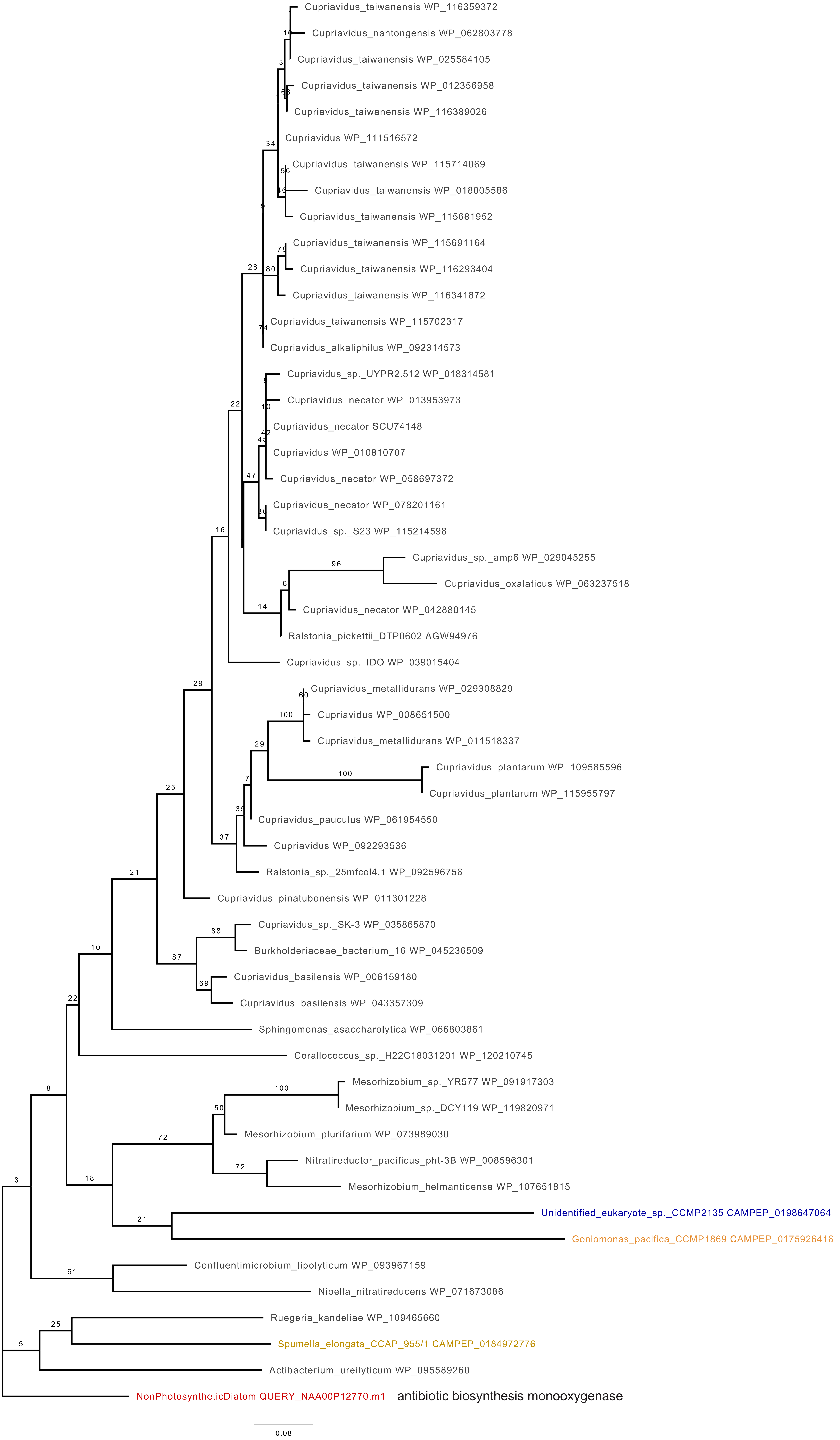

Cupriavidus_taiwanensis WP_116359372
Cupriavidus_nantongensis WP_062803778
Cupriavidus_taiwanensis WP_025584105
Cupriavidus_taiwanensis WP_012356958
Cupriavidus_taiwanensis WP_116389026
Cupriavidus WP_111516572
Cupriavidus_taiwanensis WP_115714069
Cupriavidus_taiwanensis WP_018005586
Cupriavidus_taiwanensis WP_115681952
Cupriavidus_taiwanensis WP_115691164
Cupriavidus_taiwanensis WP_116293404
Cupriavidus_taiwanensis WP_116341872
Cupriavidus_taiwanensis WP_115702317
Cupriavidus_alkaliphilus WP_092314573
Cupriavidus_sp._UYPR2.512 WP_018314581
Cupriavidus_necator WP_013953973
Cupriavidus_necator SCU74148
Cupriavidus WP_010810707
Cupriavidus_necator WP_058697372
Cupriavidus_necator WP_078201161
Cupriavidus_sp._S23 WP_115214598
Cupriavidus_sp._amp6 WP_029045255
Cupriavidus_oxalaticus WP_063237518
Cupriavidus_necator WP_042880145
Ralstonia_pickettii_DTP0602 AGW94976
Cupriavidus_sp._IDO WP_039015404
Cupriavidus_metallidurans WP_029308829
Cupriavidus WP_008651500
Cupriavidus_metallidurans WP_011518337
Cupriavidus_plantarum WP_109585596
Cupriavidus_plantarum WP_115955797
Cupriavidus_pauculus WP_061954550
Cupriavidus WP_092293536
Ralstonia_sp._25mfcol4.1 WP_092596756
Cupriavidus_pinatubonensis WP_011301228
Cupriavidus_sp._SK-3 WP_035865870
Burkholderiaceae_bacterium_16 WP_045236509
Cupriavidus_basilensis WP_006159180
Cupriavidus_basilensis WP_043357309
Sphingomonas_asaccharolytica WP_066803861
Corallococcus_sp._H22C18031201 WP_120210745
Mesorhizobium_sp._YR577 WP_091917303
Mesorhizobium_sp._DCY119 WP_119820971
Mesorhizobium_plurifarium WP_073989030
Nitratireductor_pacificus_pht-3B WP_008596301
Mesorhizobium_helmanticense WP_107651815
Unidentified_eukaryote_sp._CCMP2135 CAMPEP_0198647064
Goniomonas_pacifica_CCMP1869 CAMPEP_0175926416
Confluentimicrobium_lipolyticum WP_093967159
Nioella_nitratireducens WP_071673086
Ruegeria_kandeliae WP_109465660
Spumella_elongata_CCAP_955/1 CAMPEP_0184972776
Actibacterium_ureilyticum WP_095589260
NonPhotosyntheticDiatom QUERY_NAA00P12770.m1
antibiotic biosynthesis monooxygenase
0.08

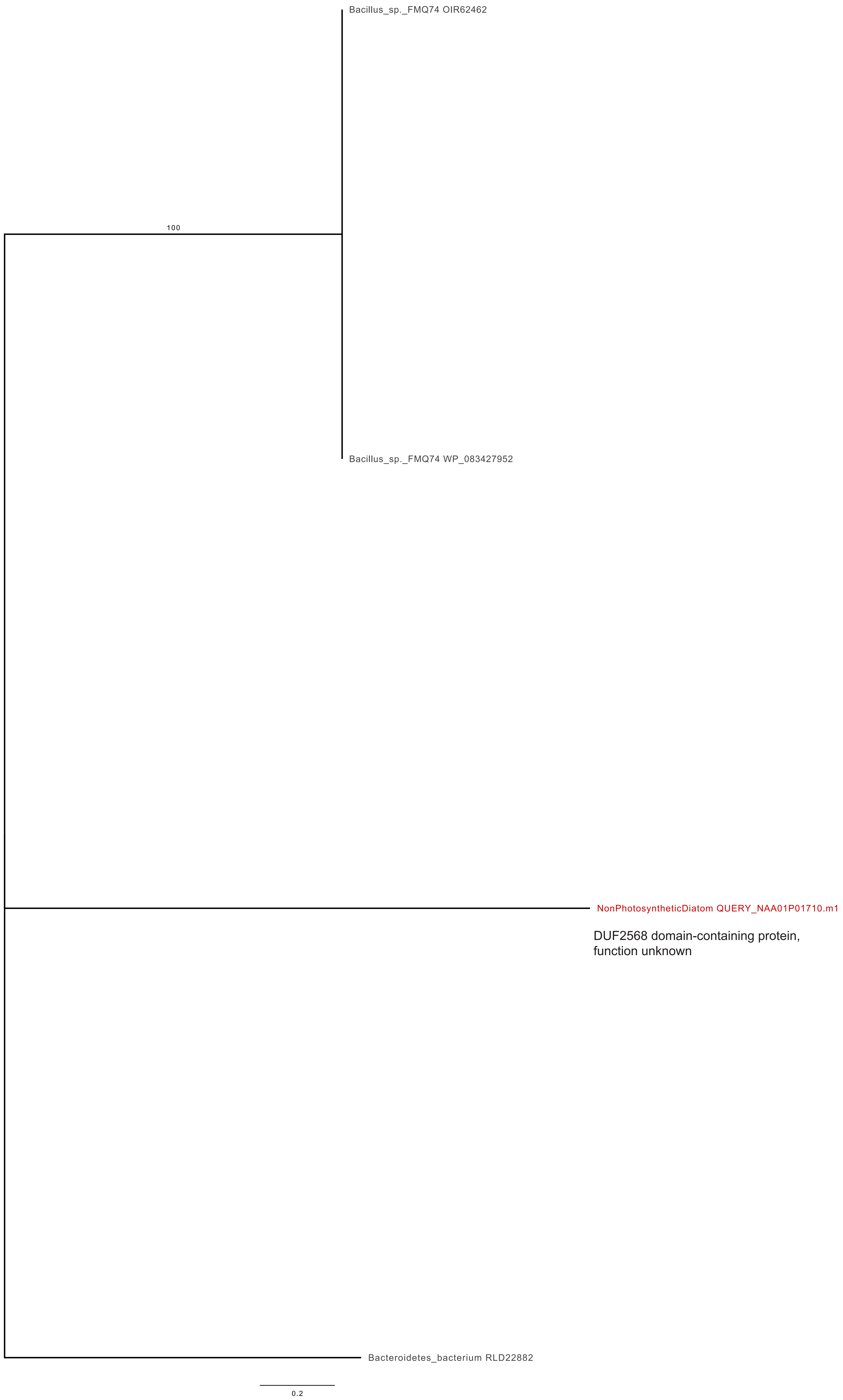

Bacillus_sp._FMQ74 OIR62462
100
Bacillus_sp._FMQ74 WP_083427952
NonPhotosyntheticDiatom QUERY_NAA01P01710.m1
DUF2568 domain-containing protein, function unknown
Bacteroidetes_bacterium RLD22882
0.2

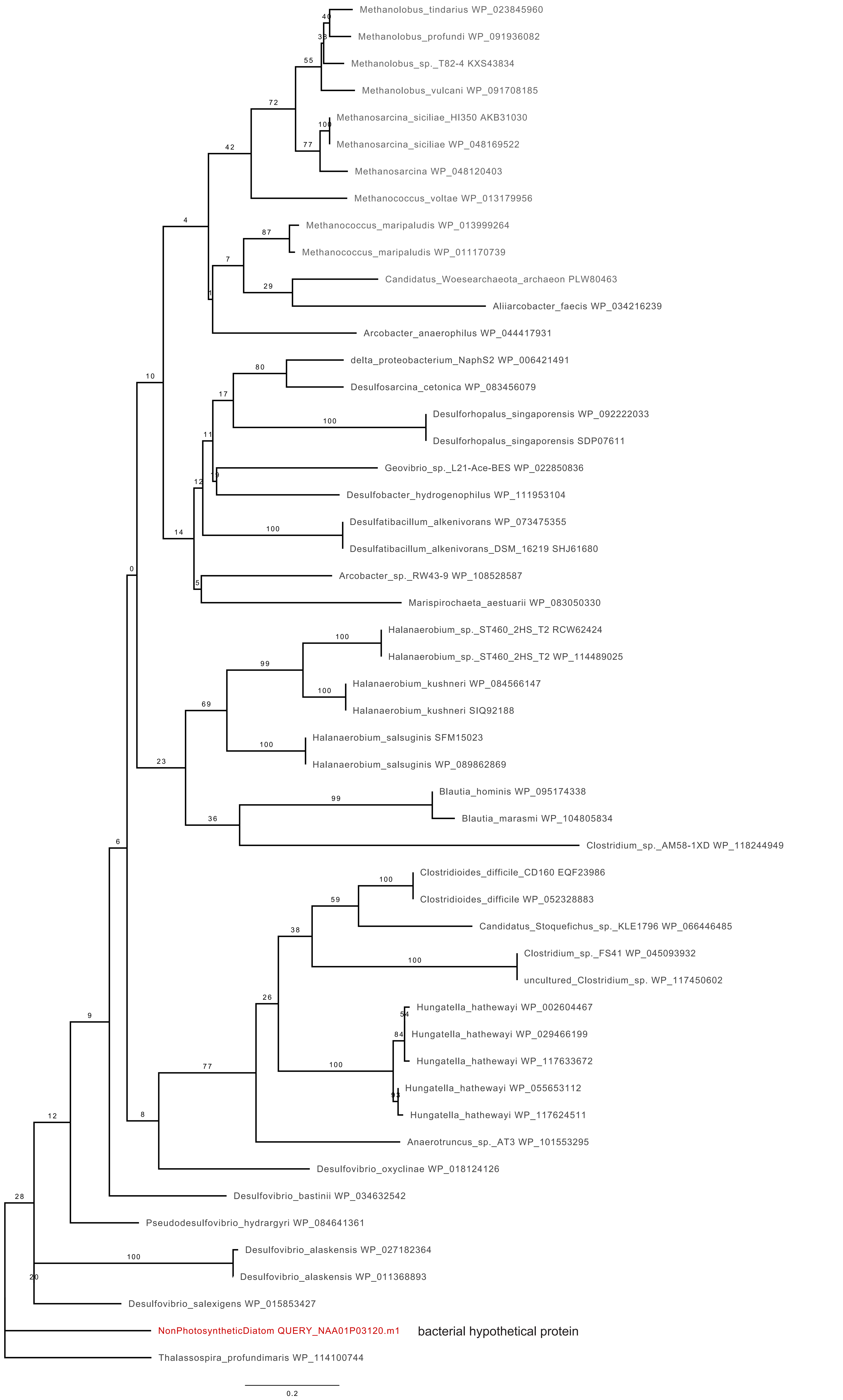
Methanolobus_tindarius WP_023845960
Methanolobus_profundi WP_091936082
Methanolobus_sp._T82-4 KXS43834
Methanolobus_vulcani WP_091708185
Methanosarcina_siciliae_HI350 AKB31030
Methanosarcina_siciliae WP_048169522
Methanosarcina WP_048120403
Methanococcus_voltae WP_013179956
Methanococcus_maripaludis WP_013999264
Methanococcus_maripaludis WP_011170739
Candidatus_Woesearchaeota_archaeon PLW80463
Aliiarcobacter_faecis WP_034216239
Arcobacter_anaerophilus WP_044417931
delta_proteobacterium_NaphS2 WP_006421491
Desulfosarcina_cetonica WP_083456079
Desulforhopalus_singaporensis WP_092222033
Desulforhopalus_singaporensis SDP07611
Geovibrio_sp._L21-Ace-BES WP_022850836
Desulfobacter_hydrogenophilus WP_111953104
Desulfatibacillum_alkenivorans WP_073475355
Desulfatibacillum_alkenivorans_DSM_16219 SHJ61680
Arcobacter_sp._RW43-9 WP_108528587
Marispirochaeta_aestuarii WP_083050330
Halanaerobium_sp._ST460_2HS_T2 RCW62424
Halanaerobium_sp._ST460_2HS_T2 WP_114489025
Halanaerobium_kushneri WP_084566147
Halanaerobium_kushneri SIQ92188
Halanaerobium_salsuginis SFM15023
Halanaerobium_salsuginis WP_089862869
Blautia_hominis WP_095174338
Blautia_marasmi WP_104805834
Clostridium_sp._AM58-1XD WP_118244949
Clostridioides_difficile_CD160 EQF23986
Clostridioides_difficile WP_052328883
Candidatus_Stoquefichus_sp._KLE1796 WP_066446485
Clostridium_sp._FS41 WP_045093932
uncultured_Clostridium_sp. WP_117450602
Hungatella_hathewayi WP_002604467
Hungatella_hathewayi WP_029466199
Hungatella_hathewayi WP_117633672
Hungatella_hathewayi WP_055653112
Hungatella_hathewayi WP_117624511
Anaerotruncus_sp._AT3 WP_101553295
Desulfovibrio_oxyclinae WP_018124126
Desulfovibrio_bastinii WP_034632542
Pseudodesulfovibrio_hydrargyri WP_084641361
Desulfovibrio_alaskensis WP_027182364
Desulfovibrio_alaskensis WP_011368893
Desulfovibrio_salexigens WP_015853427
NonPhotosyntheticDiatom QUERY_NAA01P03120.m1
bacterial hypothetical protein
Thalassospira_profundimaris WP_114100744
0.2

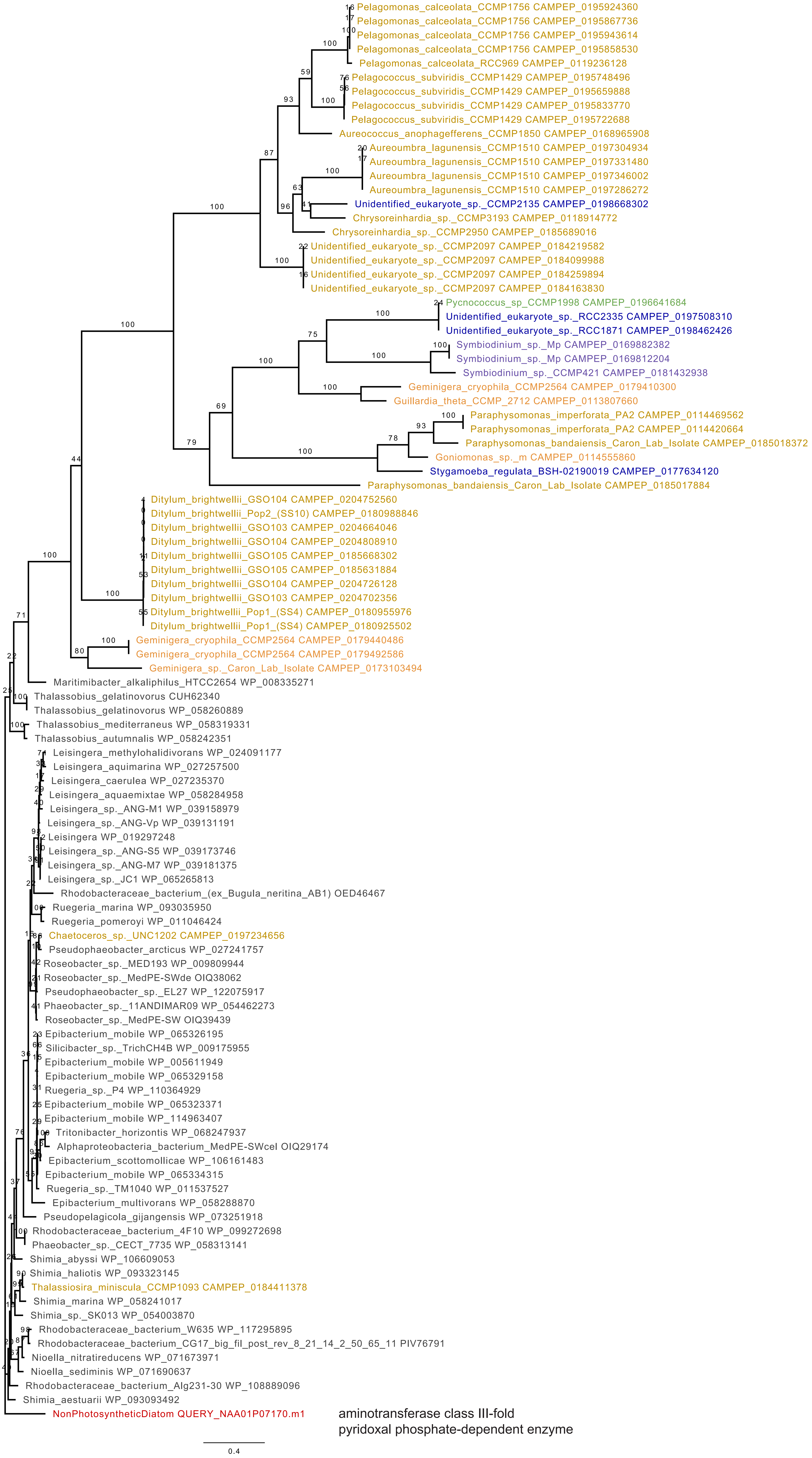

aminotransferase class III-fold
pyridoxal phosphate-dependent enzyme

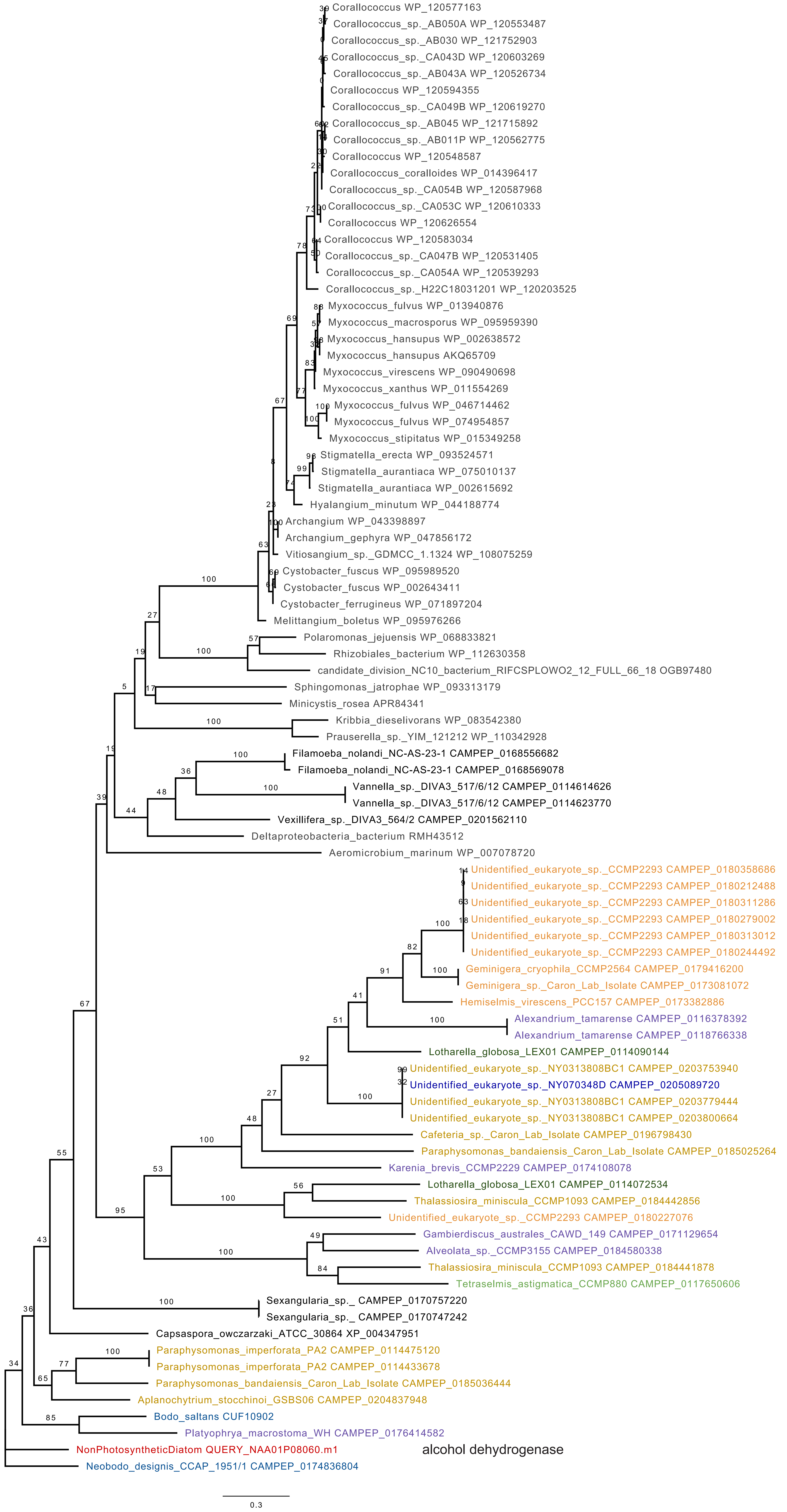
Corallococcus WP_120577163
Corallococcus_sp._AB050A WP_120553487
Corallococcus_sp._AB030 WP_121752903
Corallococcus_sp._CA043D WP_120603269
Corallococcus_sp._AB043A WP_120526734
Corallococcus WP_120594355
Corallococcus_sp._CA049B WP_120619270
Corallococcus_sp._AB045 WP_121715892
Corallococcus_sp._AB011P WP_120562775
Corallococcus WP_120548587
Corallococcus_coralloides WP_014396417
Corallococcus_sp._CA054B WP_120587968
Corallococcus_sp._CA053C WP_120610333
Corallococcus WP_120626554
Corallococcus WP_120583034
Corallococcus_sp._CA047B WP_120531405
Corallococcus_sp._CA054A WP_120539293
Corallococcus_sp._H22C18031201 WP_120203525
Myxococcus_fulvus WP_013940876
Myxococcus_macrosporus WP_095959390
Myxococcus_hansupus WP_002638572
Myxococcus_hansupus AKQ65709
Myxococcus_virescens WP_090490698
Myxococcus_xanthus WP_011554269
Myxococcus_fulvus WP_046714462
Myxococcus_fulvus WP_074954857
Myxococcus_stipitatus WP_015349258
Stigmatella_erecta WP_093524571
Stigmatella_aurantiaca WP_075010137
Stigmatella_aurantiaca WP_002615692
Hyalangium_minutum WP_044188774
Archangium WP_043398897
Archangium_gephyra WP_047856172
Vitiosangium_sp._GDMCC_1.1324 WP_108075259
Cystobacter_fuscus WP_095989520
Cystobacter_fuscus WP_002643411
Cystobacter_ferrugineus WP_071897204
Melittangium_boletus WP_095976266
Polaromonas_jejuensis WP_068833821
Rhizobiales_bacterium WP_112630358
candidate_division_NC10_bacterium_RIFCSPLOWO2_12_FULL_66_18 OGB97480
Sphingomonas_jatrophae WP_093313179
Minicystis_rosea APR84341
Kribbia_dieselivorans WP_083542380
Prauserella_sp._YIM_121212 WP_110342928
Filamoeba_nolandi_NC-AS-23-1 CAMPEP_0168556682
Filamoeba_nolandi_NC-AS-23-1 CAMPEP_0168569078
Vannella_sp._DIVA3_517/6/12 CAMPEP_0114614626
Vannella_sp._DIVA3_517/6/12 CAMPEP_0114623770
Vexillifera_sp._DIVA3_564/2 CAMPEP_0201562110
Deltaproteobacteria_bacterium RMH43512
Aeromicrobium_marinum WP_007078720
Unidentified_eukaryote_sp._CCMP2293 CAMPEP_0180358686
Unidentified_eukaryote_sp._CCMP2293 CAMPEP_0180212488
Unidentified_eukaryote_sp._CCMP2293 CAMPEP_0180311286
Unidentified_eukaryote_sp._CCMP2293 CAMPEP_0180279002
Unidentified_eukaryote_sp._CCMP2293 CAMPEP_0180313012
Unidentified_eukaryote_sp._CCMP2293 CAMPEP_0180244492
Geminigera_cryophila_CCMP2564 CAMPEP_0179416200
Geminigera_sp._Caron_Lab_Isolate CAMPEP_0173081072
Hemiselmis_virescens_PCC157 CAMPEP_0173382886
Alexandrium_tamarense CAMPEP_0116378392
Alexandrium_tamarense CAMPEP_0118766338
Lotharella_globosa_LEX01 CAMPEP_0114090144
Unidentified_eukaryote_sp._NY0313808BC1 CAMPEP_0203753940
Unidentified_eukaryote_sp._NY070348D CAMPEP_0205089720
Unidentified_eukaryote_sp._NY0313808BC1 CAMPEP_0203779444
Unidentified_eukaryote_sp._NY0313808BC1 CAMPEP_0203800664
Cafeteria_sp._Caron_Lab_Isolate CAMPEP_0196798430
Paraphysomonas_bandaiensis_Caron_Lab_Isolate CAMPEP_0185025264
Karenia_brevis_CCMP2229 CAMPEP_0174108078
Lotharella_globosa_LEX01 CAMPEP_0114072534
Thalassiosira_miniscula_CCMP1093 CAMPEP_0184442856
Unidentified_eukaryote_sp._CCMP2293 CAMPEP_0180227076
Gambierdiscus_australes_CAWD_149 CAMPEP_0171129654
Alveolata_sp._CCMP3155 CAMPEP_0184580338
Thalassiosira_miniscula_CCMP1093 CAMPEP_0184441878
Tetraselmis_astigmatica_CCMP880 CAMPEP_0117650606
Sexangularia_sp._ CAMPEP_0170757220
Sexangularia_sp._ CAMPEP_0170747242
Capsaspora_owczarzaki_ATCC_30864 XP_004347951
Paraphysomonas_imperforata_PA2 CAMPEP_0114475120
Paraphysomonas_imperforata_PA2 CAMPEP_0114433678
Paraphysomonas_bandaiensis_Caron_Lab_Isolate CAMPEP_0185036444
Aplanochytrium_stocchinoi_GSBS06 CAMPEP_0204837948
Bodo_saltans CUF10902
Platyophrya_macrostoma_WH CAMPEP_0176414582
NonPhotosyntheticDiatom QUERY_NAA01P08060.m1
alcohol dehydrogenase
Neobodo_designis_CCAP_1951/1 CAMPEP_0174836804
0.3

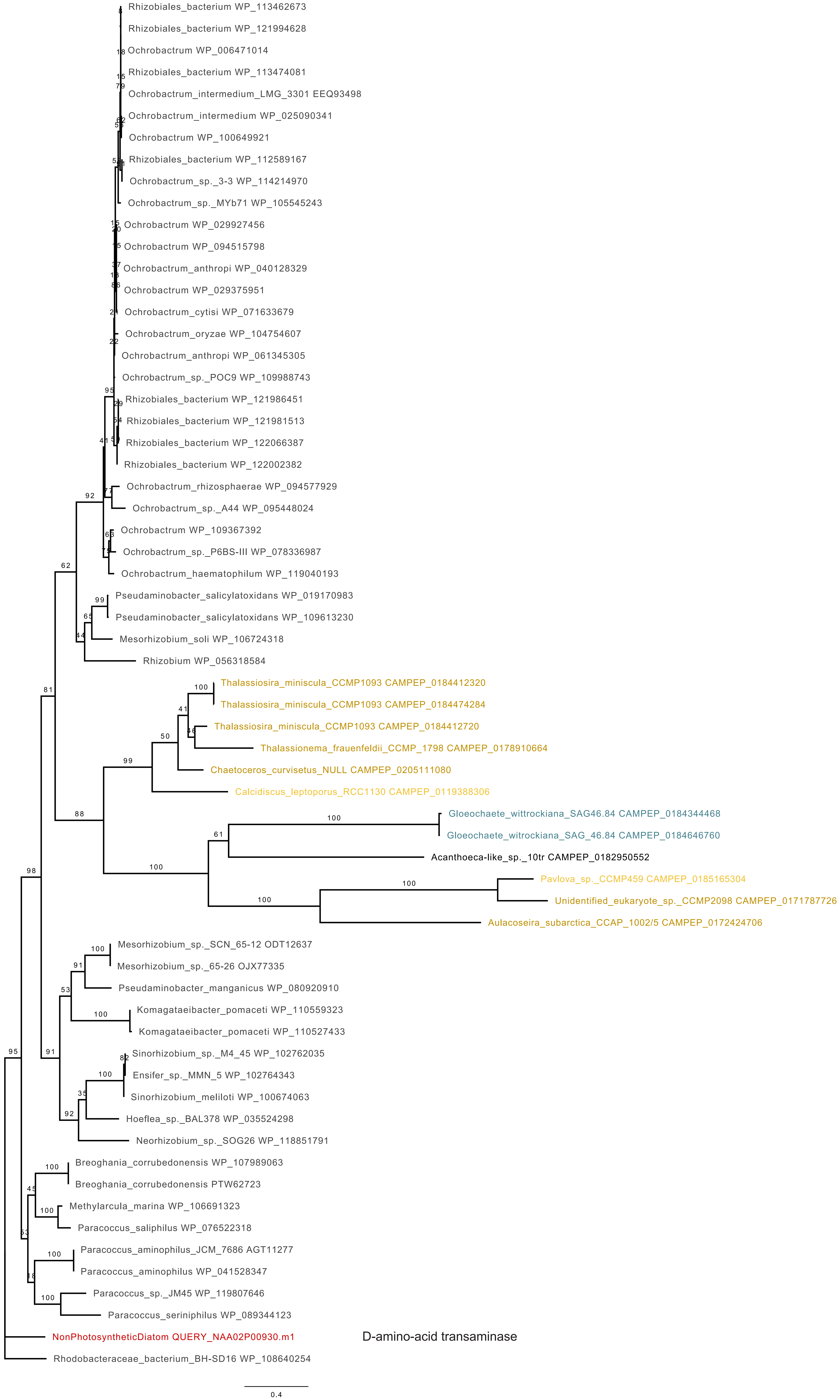
Rhizobiales_bacterium WP_113462673
Rhizobiales_bacterium WP_121994628
Ochrobactrum WP_006471014
Rhizobiales_bacterium WP_113474081
Ochrobactrum_intermedium_LMG_3301 EEQ93498
Ochrobactrum_intermedium WP_025090341
Ochrobactrum WP_100649921
Rhizobiales_bacterium WP_112589167
Ochrobactrum_sp._3-3 WP_114214970
Ochrobactrum_sp._MYb71 WP_105545243
Ochrobactrum WP_029927456
Ochrobactrum WP_094515798
Ochrobactrum_anthropi WP_040128329
Ochrobactrum WP_029375951
Ochrobactrum_cytisi WP_071633679
Ochrobactrum_oryzae WP_104754607
Ochrobactrum_anthropi WP_061345305
Ochrobactrum_sp._POC9 WP_109988743
Rhizobiales_bacterium WP_121986451
Rhizobiales_bacterium WP_121981513
Rhizobiales_bacterium WP_122066387
Rhizobiales_bacterium WP_122002382
Ochrobactrum_rhizosphaerae WP_094577929
Ochrobactrum_sp._A44 WP_095448024
Ochrobactrum WP_109367392
Ochrobactrum_sp._P6BS-III WP_078336987
Ochrobactrum_haematophilum WP_119040193
Pseudaminobacter_salicylatoxidans WP_019170983
Pseudaminobacter_salicylatoxidans WP_109613230
Mesorhizobium_soli WP_106724318
Rhizobium WP_056318584
Thalassiosira_miniscula_CCMP1093 CAMPEP_0184412320
Thalassiosira_miniscula_CCMP1093 CAMPEP_0184474284
Thalassiosira_miniscula_CCMP1093 CAMPEP_0184412720
Thalassionema_frauenfeldii_CCMP_1798 CAMPEP_0178910664
Chaetoceros_curvisetus_NULL CAMPEP_0205111080
Calcidiscus_leptoporus_RCC1130 CAMPEP_0119388306
Gloeochaete_wittrockiana_SAG46.84 CAMPEP_0184344468
Gloeochaete_witrockiana_SAG_46.84 CAMPEP_0184646760
Acanthoeca-like_sp._10tr CAMPEP_0182950552
Pavlova_sp._CCMP459 CAMPEP_0185165304
Unidentified_eukaryote_sp._CCMP2098 CAMPEP_0171787726
Aulacoseira_subarctica_CCAP_1002/5 CAMPEP_0172424706
Mesorhizobium_sp._SCN_65-12 ODT12637
Mesorhizobium_sp._65-26 OJX77335
Pseudaminobacter_manganicus WP_080920910
Komagataeibacter_pomaceti WP_110559323
Komagataeibacter_pomaceti WP_110527433
Sinorhizobium_sp._M4_45 WP_102762035
Ensifer_sp._MMN_5 WP_102764343
Sinorhizobium_meliloti WP_100674063
Hoeflea_sp._BAL378 WP_035524298
Neorhizobium_sp._SOG26 WP_118851791
Breoghania_corrubedonensis WP_107989063
Breoghania_corrubedonensis PTW62723
Methylarcula_marina WP_106691323
Paracoccus_saliphilus WP_076522318
Paracoccus_aminophilus_JCM_7686 AGT11277
Paracoccus_aminophilus WP_041528347
Paracoccus_sp._JM45 WP_119807646
Paracoccus_seriniphilus WP_089344123
NonPhotosyntheticDiatom QUERY_NAA02P00930.m1
D-amino-acid transaminase
Rhodobacteraceae_bacterium_BH-SD16 WP_108640254
0.4

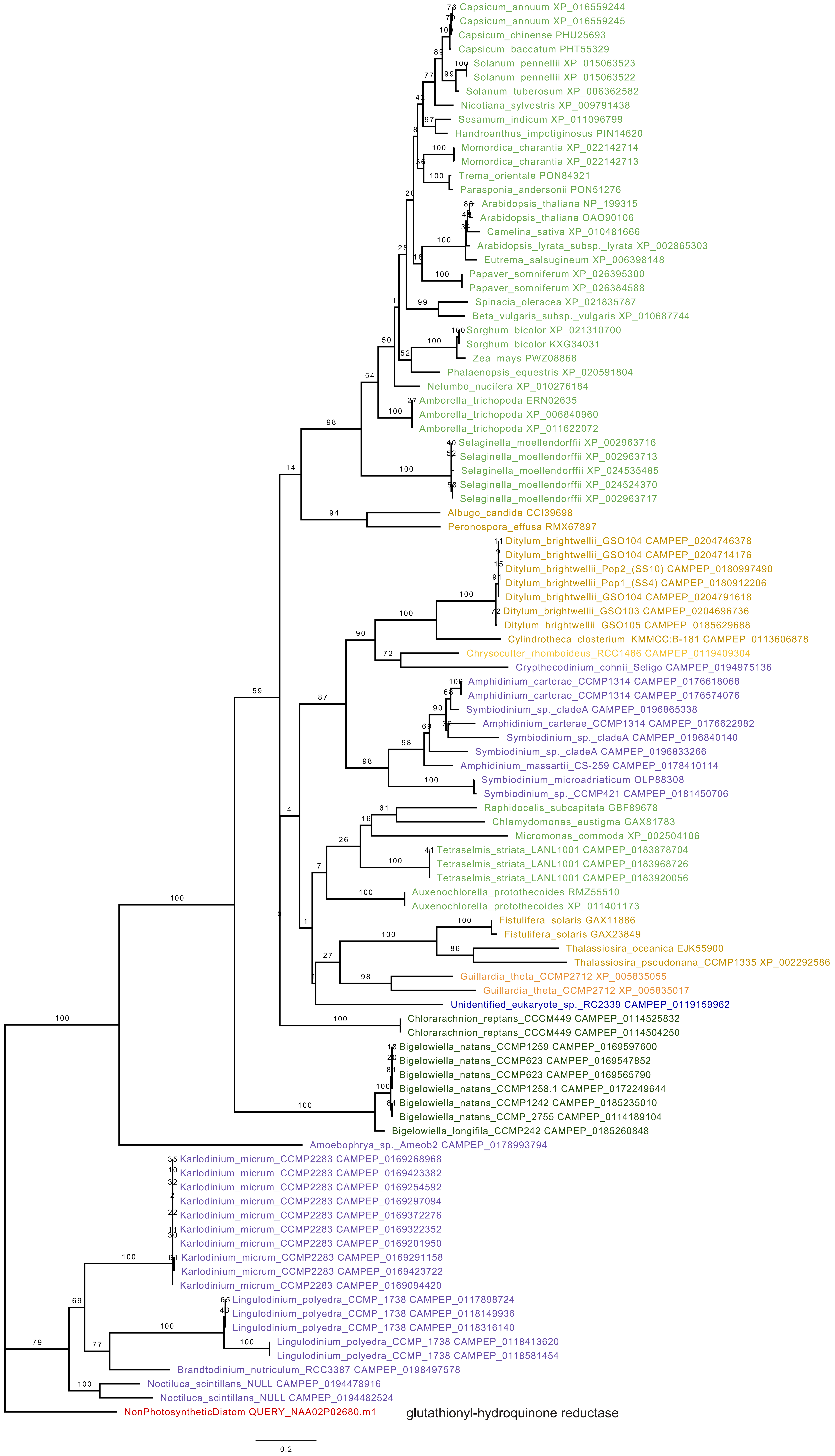
Capsicum_annuum XP_016559244
Capsicum_annuum XP_016559245
Capsicum_chinense PHU25693
Capsicum_baccatum PHT55329
Solanum_pennellii XP_015063523
Solanum_pennellii XP_015063522
Solanum_tuberosum XP_006362582
Nicotiana_sylvestris XP_009791438
Sesamum_indicum XP_011096799
Handroanthus_impetiginosus PIN14620
Momordica_charantia XP_022142714
Momordica_charantia XP_022142713
Trema_orientale PON84321
Parasponia_andersonii PON51276
Arabidopsis_thaliana NP_199315
Arabidopsis_thaliana OAO90106
Camelina_sativa XP_010481666
Arabidopsis_lyrata_subsp._lyrata XP_002865303
Eutrema_salsugineum XP_006398148
Papaver_somniferum XP_026395300
Papaver_somniferum XP_026384588
Spinacia_oleracea XP_021835787
Beta_vulgaris_subsp._vulgaris XP_010687744
Sorghum_bicolor XP_021310700
Sorghum_bicolor KXG34031
Zea_mays PWZ08868
Phalaenopsis_equestris XP_020591804
Nelumbo_nucifera XP_010276184
Amborella_trichopoda ERN02635
Amborella_trichopoda XP_006840960
Amborella_trichopoda XP_011622072
Selaginella_moellendorffii XP_002963716
Selaginella_moellendorffii XP_002963713
Selaginella_moellendorffii XP_024535485
Selaginella_moellendorffii XP_024524370
Selaginella_moellendorffii XP_002963717
Albugo_candida CCI39698
Peronospora_effusa RMX67897
Ditylum_brightwellii_GSO104 CAMPEP_0204746378
Ditylum_brightwellii_GSO104 CAMPEP_0204714176
Ditylum_brightwellii_Pop2_(SS10) CAMPEP_0180997490
Ditylum_brightwellii_Pop1_(SS4) CAMPEP_0180912206
Ditylum_brightwellii_GSO104 CAMPEP_0204791618
Ditylum_brightwellii_GSO103 CAMPEP_0204696736
Ditylum_brightwellii_GSO105 CAMPEP_0185629688
Cylindrotheca_closterium_KMMCC:B-181 CAMPEP_0113606878
Chrysoculter_rhomboideus_RCC1486 CAMPEP_0119409304
Crypthecodinium_cohnii_Seligo CAMPEP_0194975136
Amphidinium_carterae_CCMP1314 CAMPEP_0176618068
Amphidinium_carterae_CCMP1314 CAMPEP_0176574076
Symbiodinium_sp._cladeA CAMPEP_0196865338
Amphidinium_carterae_CCMP1314 CAMPEP_0176622982
Symbiodinium_sp._cladeA CAMPEP_0196840140
Symbiodinium_sp._cladeA CAMPEP_0196833266
Amphidinium_massartii_CS-259 CAMPEP_0178410114
Symbiodinium_microadriaticum OLP88308
Symbiodinium_sp._CCMP421 CAMPEP_0181450706
Raphidocelis_subcapitata GBF89678
Chlamydomonas_eustigma GAX81783
Micromonas_commoda XP_002504106
Tetraselmis_striata_LANL1001 CAMPEP_0183878704
Tetraselmis_striata_LANL1001 CAMPEP_0183968726
Tetraselmis_striata_LANL1001 CAMPEP_0183920056
Auxenochlorella_protothecoides RMZ55510
Auxenochlorella_protothecoides XP_011401173
Fistulifera_solaris GAX11886
Fistulifera_solaris GAX23849
Thalassiosira_oceanica EJK55900
Thalassiosira_pseudonana_CCMP1335 XP_002292586
Guillardia_theta_CCMP2712 XP_005835055
Guillardia_theta_CCMP2712 XP_005835017
Unidentified_eukaryote_sp._RC2339 CAMPEP_0119159962
Chlorarachnion_reptans_CCCM449 CAMPEP_0114525832
Chlorarachnion_reptans_CCCM449 CAMPEP_0114504250
Bigelowiella_natans_CCMP1259 CAMPEP_0169597600
Bigelowiella_natans_CCMP623 CAMPEP_0169547852
Bigelowiella_natans_CCMP623 CAMPEP_0169565790
Bigelowiella_natans_CCMP1258.1 CAMPEP_0172249644
Bigelowiella_natans_CCMP1242 CAMPEP_0185235010
Bigelowiella_natans_CCMP_2755 CAMPEP_0114189104
Bigelowiella_longifila_CCMP242 CAMPEP_0185260848
Amoebophrya_sp._Ameob2 CAMPEP_0178993794
Karlodinium_micrum_CCMP2283 CAMPEP_0169268968
Karlodinium_micrum_CCMP2283 CAMPEP_0169423382
Karlodinium_micrum_CCMP2283 CAMPEP_0169254592
Karlodinium_micrum_CCMP2283 CAMPEP_0169297094
Karlodinium_micrum_CCMP2283 CAMPEP_0169372276
Karlodinium_micrum_CCMP2283 CAMPEP_0169322352
Karlodinium_micrum_CCMP2283 CAMPEP_0169201950
Karlodinium_micrum_CCMP2283 CAMPEP_0169291158
Karlodinium_micrum_CCMP2283 CAMPEP_0169423722
Karlodinium_micrum_CCMP2283 CAMPEP_0169094420
Lingulodinium_polyedra_CCMP_1738 CAMPEP_0117898724
Lingulodinium_polyedra_CCMP_1738 CAMPEP_0118149936
Lingulodinium_polyedra_CCMP_1738 CAMPEP_0118316140
Lingulodinium_polyedra_CCMP_1738 CAMPEP_0118413620
Lingulodinium_polyedra_CCMP_1738 CAMPEP_0118581454
Brandtodinium_nutriculum_RCC3387 CAMPEP_0198497578
Noctiluca_scintillans_NULL CAMPEP_0194478916
Noctiluca_scintillans_NULL CAMPEP_0194482524
NonPhotosyntheticDiatom QUERY_NAA02P02680.m1
glutathionyl-hydroquinone reductase
0.2

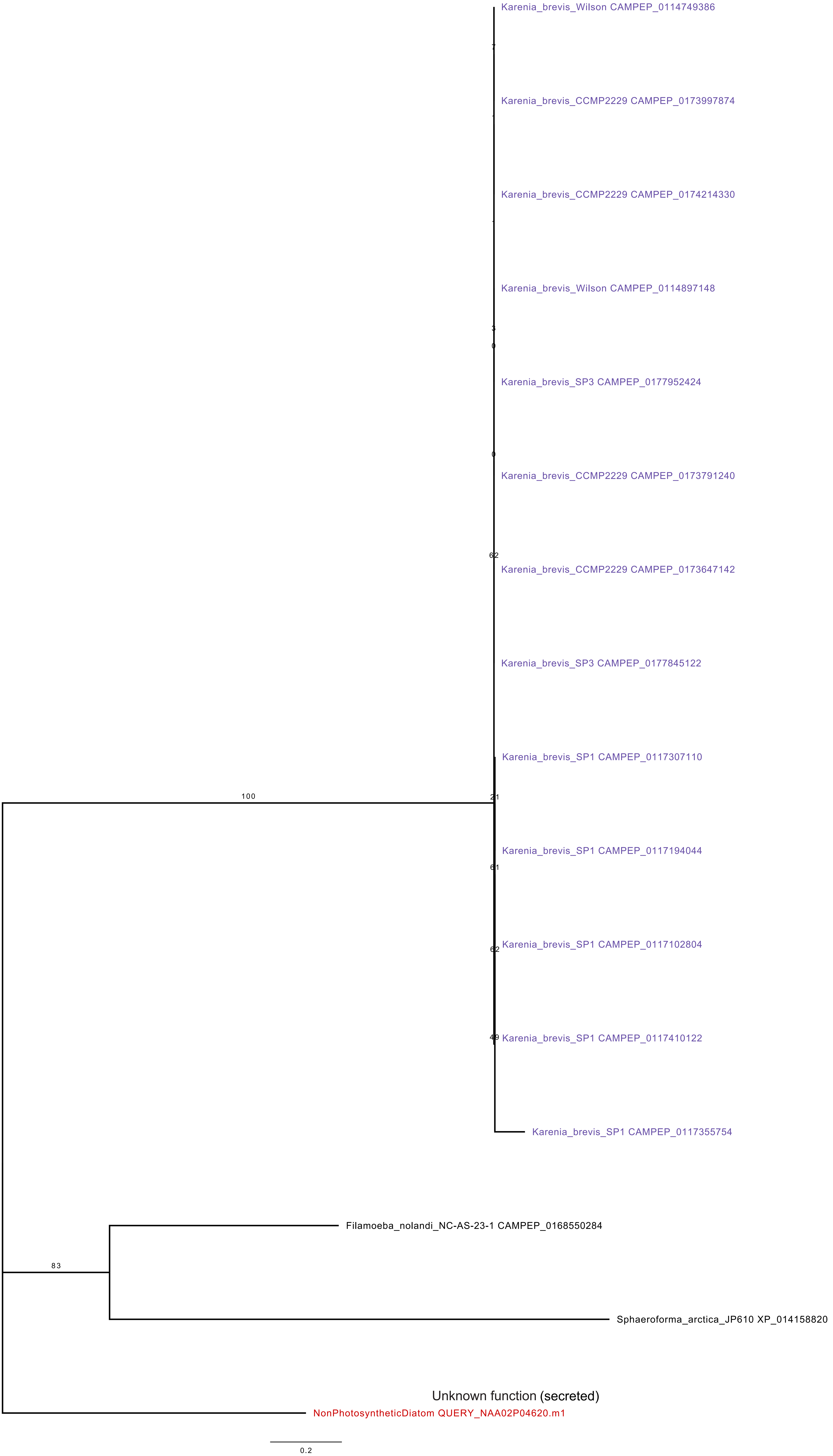
Karenia_brevis_Wilson CAMPEP_0114749386
Karenia_brevis_CCMP2229 CAMPEP_0173997874
Karenia_brevis_CCMP2229 CAMPEP_0174214330
Karenia_brevis_Wilson CAMPEP_0114897148
3
0
Karenia_brevis_SP3 CAMPEP_0177952424
0
Karenia_brevis_CCMP2229 CAMPEP_0173791240
62
Karenia_brevis_CCMP2229 CAMPEP_0173647142
Karenia_brevis_SP3 CAMPEP_0177845122
Karenia_brevis_SP1 CAMPEP_0117307110
100
21
Karenia_brevis_SP1 CAMPEP_0117194044
61
Karenia_brevis_SP1 CAMPEP_0117102804
62
Karenia_brevis_SP1 CAMPEP_0117410122
49
Karenia_brevis_SP1 CAMPEP_0117355754
Filamoeba_nolandi_NC-AS-23-1 CAMPEP_0168550284
83
Sphaeroforma_arctica_JP610 XP_014158820
Unknown function (secreted)
NonPhotosyntheticDiatom QUERY_NAA02P04620.m1
0.2

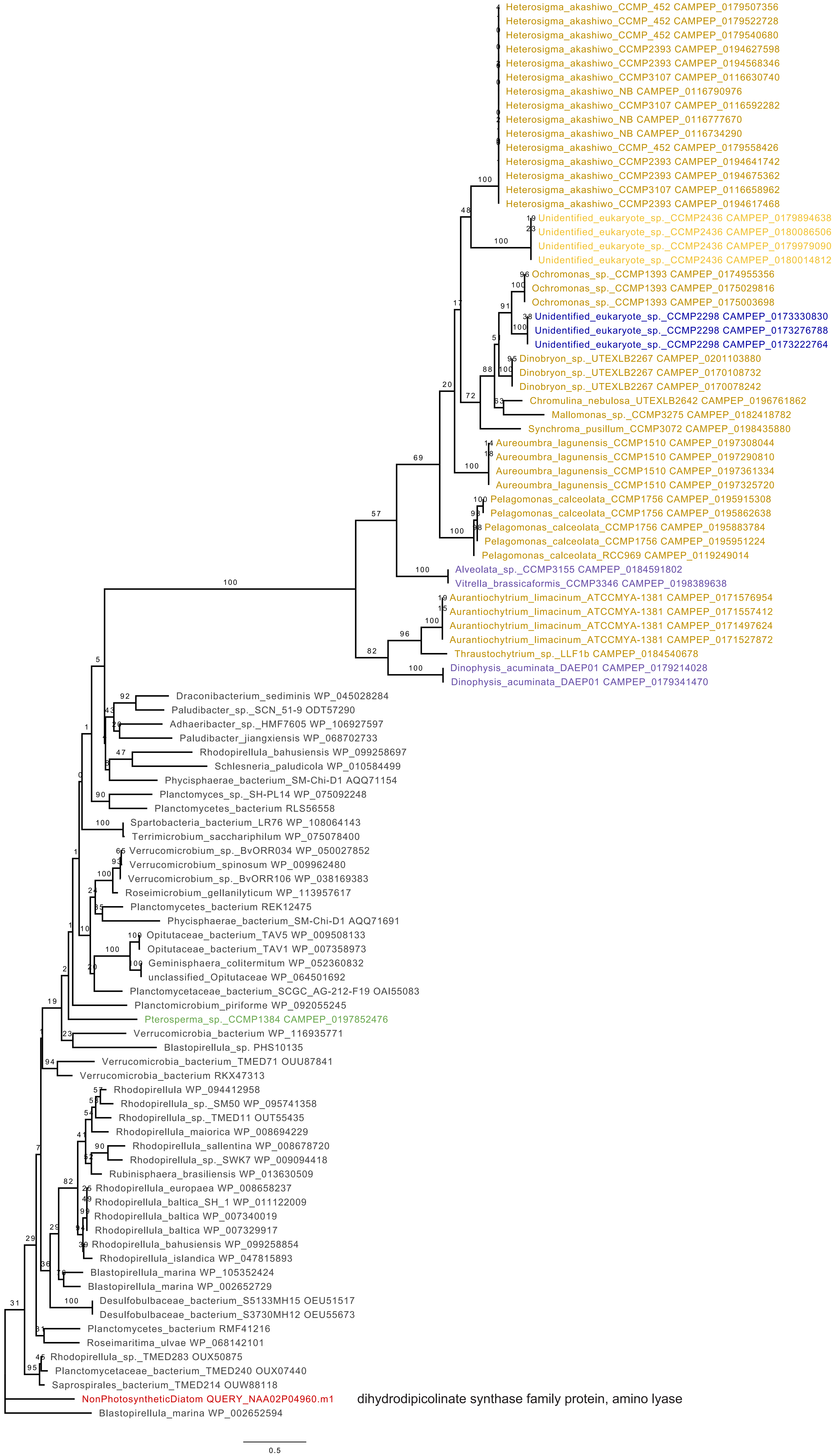
Heterosigma_akashiwo_CCMP_452 CAMPEP_0179507356
Heterosigma_akashiwo_CCMP_452 CAMPEP_0179522728
Heterosigma_akashiwo_CCMP_452 CAMPEP_0179540680
Heterosigma_akashiwo_CCMP2393 CAMPEP_0194627598
Heterosigma_akashiwo_CCMP2393 CAMPEP_0194568346
Heterosigma_akashiwo_CCMP3107 CAMPEP_0116630740
Heterosigma_akashiwo_NB CAMPEP_0116790976
Heterosigma_akashiwo_CCMP3107 CAMPEP_0116592282
Heterosigma_akashiwo_NB CAMPEP_0116777670
Heterosigma_akashiwo_NB CAMPEP_0116734290
Heterosigma_akashiwo_CCMP_452 CAMPEP_0179558426
Heterosigma_akashiwo_CCMP2393 CAMPEP_0194641742
Heterosigma_akashiwo_CCMP2393 CAMPEP_0194675362
Heterosigma_akashiwo_CCMP3107 CAMPEP_0116658962
Heterosigma_akashiwo_CCMP2393 CAMPEP_0194617468
Unidentified_eukaryote_sp._CCMP2436 CAMPEP_0179894638
Unidentified_eukaryote_sp._CCMP2436 CAMPEP_0180086506
Unidentified_eukaryote_sp._CCMP2436 CAMPEP_0179979090
Unidentified_eukaryote_sp._CCMP2436 CAMPEP_0180014812
Ochromonas_sp._CCMP1393 CAMPEP_0174955356
Ochromonas_sp._CCMP1393 CAMPEP_0175029816
Ochromonas_sp._CCMP1393 CAMPEP_0175003698
Unidentified_eukaryote_sp._CCMP2298 CAMPEP_0173330830
Unidentified_eukaryote_sp._CCMP2298 CAMPEP_0173276788
Unidentified_eukaryote_sp._CCMP2298 CAMPEP_0173222764
Dinobryon_sp._UTEXLB2267 CAMPEP_0201103880
Dinobryon_sp._UTEXLB2267 CAMPEP_0170108732
Dinobryon_sp._UTEXLB2267 CAMPEP_0170078242
Chromulina_nebulosa_UTEXLB2642 CAMPEP_0196761862
Mallomonas_sp._CCMP3275 CAMPEP_0182418782
Synchroma_pusillum_CCMP3072 CAMPEP_0198435880
Aureoumbra_lagunensis_CCMP1510 CAMPEP_0197308044
Aureoumbra_lagunensis_CCMP1510 CAMPEP_0197290810
Aureoumbra_lagunensis_CCMP1510 CAMPEP_0197361334
Aureoumbra_lagunensis_CCMP1510 CAMPEP_0197325720
Pelagomonas_calceolata_CCMP1756 CAMPEP_0195915308
Pelagomonas_calceolata_CCMP1756 CAMPEP_0195862638
Pelagomonas_calceolata_CCMP1756 CAMPEP_0195883784
Pelagomonas_calceolata_CCMP1756 CAMPEP_0195951224
Pelagomonas_calceolata_RCC969 CAMPEP_0119249014
Alveolata_sp._CCMP3155 CAMPEP_0184591802
Vitrella_brassicaformis_CCMP3346 CAMPEP_0198389638
Aurantiochytrium_limacinum_ATCCMYA-1381 CAMPEP_0171576954
Aurantiochytrium_limacinum_ATCCMYA-1381 CAMPEP_0171557412
Aurantiochytrium_limacinum_ATCCMYA-1381 CAMPEP_0171497624
Aurantiochytrium_limacinum_ATCCMYA-1381 CAMPEP_0171527872
Thraustochytrium_sp._LLF1b CAMPEP_0184540678
Dinophysis_acuminata_DAEP01 CAMPEP_0179214028
Dinophysis_acuminata_DAEP01 CAMPEP_0179341470
Draconibacterium_sediminis WP_045028284
Paludibacter_sp._SCN_51-9 ODT57290
Adhaeribacter_sp._HMF7605 WP_106927597
Paludibacter_jiangxiensis WP_068702733
Rhodopirellula_bahusiensis WP_099258697
Schlesneria_paludicola WP_010584499
Phycisphaerae_bacterium_SM-Chi-D1 AQQ71154
Planctomyces_sp._SH-PL14 WP_075092248
Planctomycetes_bacterium RLS56558
Spartobacteria_bacterium_LR76 WP_108064143
Terrimicrobium_sacchariphilum WP_075078400
Verrucomicrobium_sp._BvORR034 WP_050027852
Verrucomicrobium_spinosum WP_009962480
Verrucomicrobium_sp._BvORR106 WP_038169383
Roseimicrobium_gellanilyticum WP_113957617
Planctomycetes_bacterium REK12475
Phycisphaerae_bacterium_SM-Chi-D1 AQQ71691
Opitutaceae_bacterium_TAV5 WP_009508133
Opitutaceae_bacterium_TAV1 WP_007358973
Geminisphaera_colitermitum WP_052360832
unclassified_Opitutaceae WP_064501692
Planctomycetaceae_bacterium_SCGC_AG-212-F19 OAI55083
Planctomicrobium_piriforme WP_092055245
Pterosperma_sp._CCMP1384 CAMPEP_0197852476
Verrucomicrobia_bacterium WP_116935771
Blastopirellula_sp. PHS10135
Verrucomicrobia_bacterium_TMED71 OUU87841
Verrucomicrobia_bacterium RKX47313
Rhodopirellula WP_094412958
Rhodopirellula_sp._SM50 WP_095741358
Rhodopirellula_sp._TMED11 OUT55435
Rhodopirellula_maiorica WP_008694229
Rhodopirellula_sallentina WP_008678720
Rhodopirellula_sp._SWK7 WP_009094418
Rubinisphaera_brasiliensis WP_013630509
Rhodopirellula_europaea WP_008658237
Rhodopirellula_baltica_SH_1 WP_011122009
Rhodopirellula_baltica WP_007340019
Rhodopirellula_baltica WP_007329917
Rhodopirellula_bahusiensis WP_099258854
Rhodopirellula_islandica WP_047815893
Blastopirellula_marina WP_105352424
Blastopirellula_marina WP_002652729
Desulfobulbaceae_bacterium_S5133MH15 OEU51517
Desulfobulbaceae_bacterium_S3730MH12 OEU55673
Planctomycetes_bacterium RMF41216
Roseimaritima_ulvae WP_068142101
Rhodopirellula_sp._TMED283 OUX50875
Planctomycetaceae_bacterium_TMED240 OUX07440
Saprospirales_bacterium_TMED214 OUW88118
NonPhotosyntheticDiatom QUERY_NAA02P04960.m1
dihydrodipicolinate synthase family protein, amino lyase
Blastopirellula_marina WP_002652594
0.5

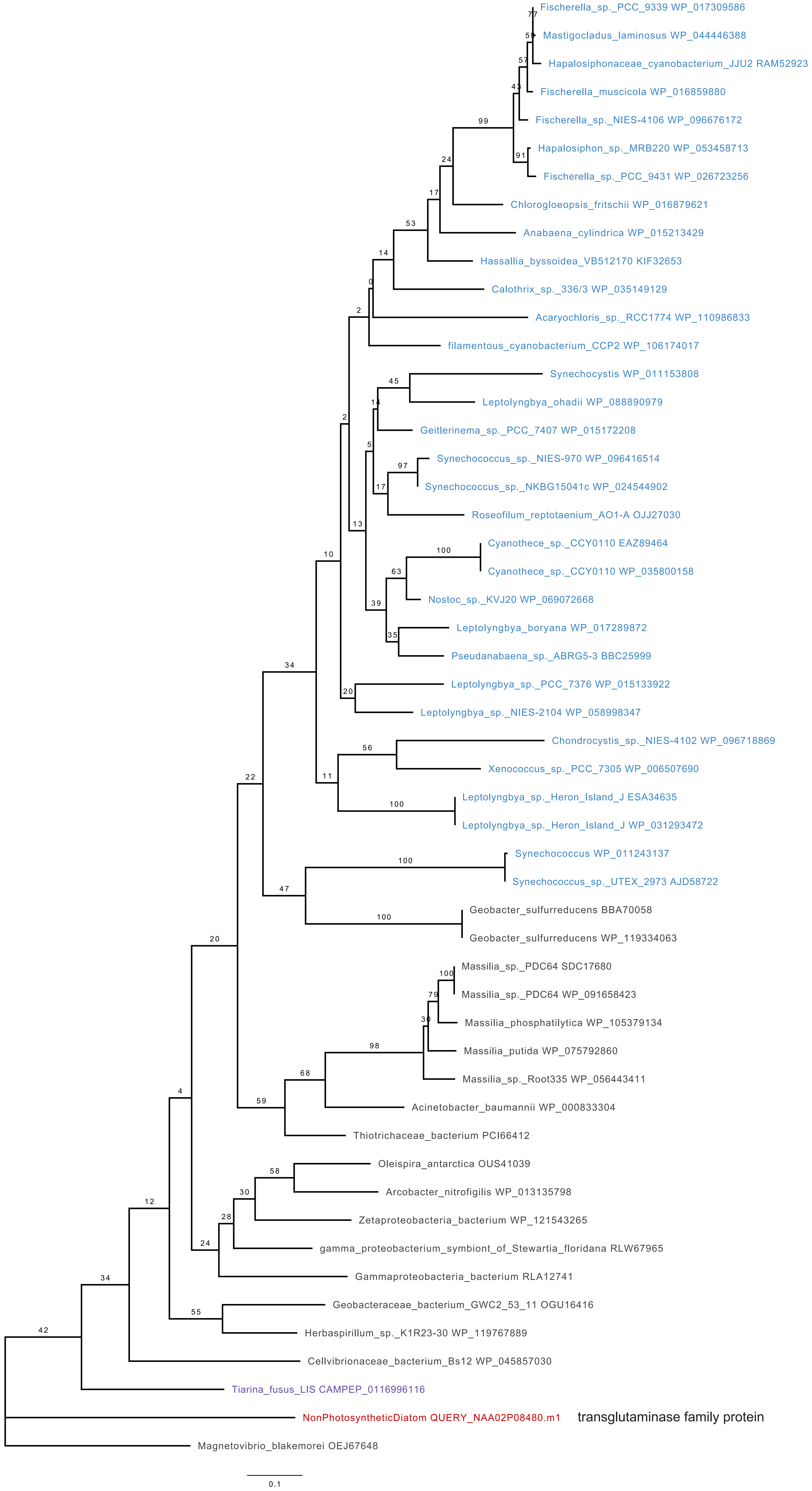
Fischerella_sp._PCC_9339 WP_017309586
Mastigocladus_laminosus WP_044446388
Hapalosiphonaceae_cyanobacterium_JJU2 RAM52923
Fischerella_muscicola WP_016859880
Fischerella_sp._NIES-4106 WP_096676172
Hapalosiphon_sp._MRB220 WP_053458713
Fischerella_sp._PCC_9431 WP_026723256
Chlorogloeopsis_fritschii WP_016879621
Anabaena_cylindrica WP_015213429
Hassallia_byssoidea_VB512170 KIF32653
Calothrix_sp._336/3 WP_035149129
Acaryochloris_sp._RCC1774 WP_110986833
filamentous_cyanobacterium_CCP2 WP_106174017
Synechocystis WP_011153808
Leptolyngbya_ohadii WP_088890979
Geitlerinema_sp._PCC_7407 WP_015172208
Synechococcus_sp._NIES-970 WP_096416514
Synechococcus_sp._NKBG15041c WP_024544902
Roseofilum_reptotaenium_AO1-A OJJ27030
Cyanothece_sp._CCY0110 EAZ89464
Cyanothece_sp._CCY0110 WP_035800158
Nostoc_sp._KVJ20 WP_069072668
Leptolyngbya_boryana WP_017289872
Pseudanabaena_sp._ABRG5-3 BBC25999
Leptolyngbya_sp._PCC_7376 WP_015133922
Leptolyngbya_sp._NIES-2104 WP_058998347
Chondrocystis_sp._NIES-4102 WP_096718869
Xenococcus_sp._PCC_7305 WP_006507690
Leptolyngbya_sp._Heron_Island_J ESA34635
Leptolyngbya_sp._Heron_Island_J WP_031293472
Synechococcus WP_011243137
Synechococcus_sp._UTEX_2973 AJD58722
Geobacter_sulfurreducens BBA70058
Geobacter_sulfurreducens WP_119334063
Massilia_sp._PDC64 SDC17680
Massilia_sp._PDC64 WP_091658423
Massilia_phosphatilytica WP_105379134
Massilia_putida WP_075792860
Massilia_sp._Root335 WP_056443411
Acinetobacter_baumannii WP_000833304
Thiotrichaceae_bacterium PCI66412
Oleispira_antarctica OUS41039
Arcobacter_nitrofigilis WP_013135798
Zetaproteobacteria_bacterium WP_121543265
gamma_proteobacterium_symbiont_of_Stewartia_floridana RLW67965
Gammaproteobacteria_bacterium RLA12741
Geobacteraceae_bacterium_GWC2_53_11 OGU16416
Herbaspirillum_sp._K1R23-30 WP_119767889
Cellvibrionaceae_bacterium_Bs12 WP_045857030
Tiarina_fusus_LIS CAMPEP_0116996116
NonPhotosyntheticDiatom QUERY_NAA02P08480.m1
transglutaminase family protein
Magnetovibrio_blakemorei OEJ67648
0.1

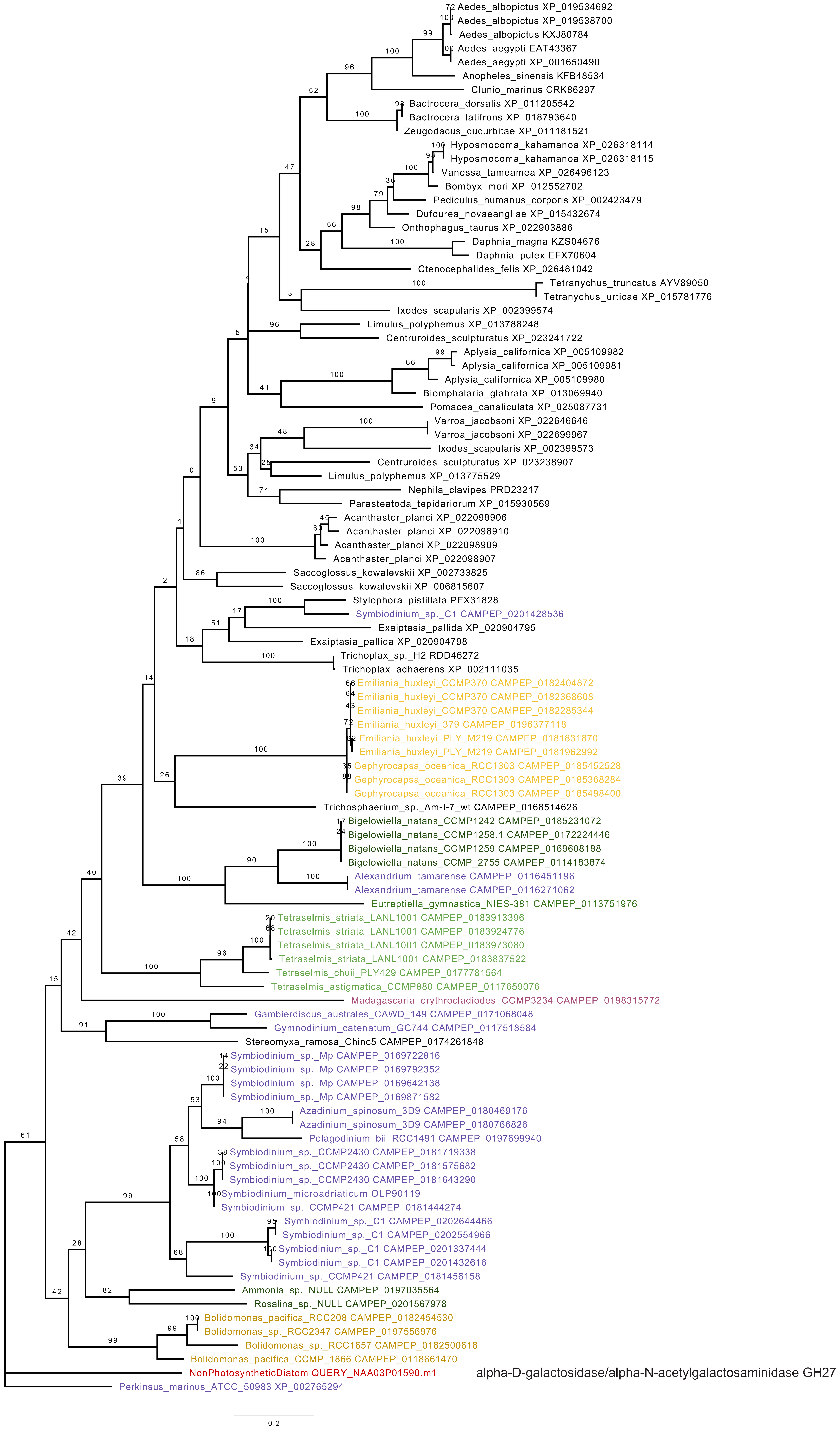

Aedes_albopictus XP_019534692
Aedes_albopictus XP_019538700
Aedes_albopictus KXJ80784
Aedes_aegypti EAT43367
Aedes_aegypti XP_001650490
Anopheles_sinensis KFB48534
Clunio_marinus CRK86297
Bactrocera_dorsalis XP_011205542
Bactrocera_latifrons XP_018793640
Zeugodacus_cucurbitae XP_011181521
Hyposmocoma_kahamanoa XP_026318114
Hyposmocoma_kahamanoa XP_026318115
Vanessa_tameamea XP_026496123
Bombyx_mori XP_012552702
Pediculus_humanus_corporis XP_002423479
Dufourea_novaeangliae XP_015432674
Onthophagus_taurus XP_022903886
Daphnia_magna KZS04676
Daphnia_pulex EFX70604
Ctenocephalides_felis XP_026481042
Tetranychus_truncatus AYV89050
Tetranychus_urticae XP_015781776
Ixodes_scapularis XP_002399574
Limulus_polyphemus XP_013788248
Centruroides_sculpturatus XP_023241722
Aplysia_californica XP_005109982
Aplysia_californica XP_005109981
Aplysia_californica XP_005109980
Biomphalaria_glabrata XP_013069940
Pomacea_canaliculata XP_025087731
Varroa_jacobsoni XP_022646646
Varroa_jacobsoni XP_022699967
Ixodes_scapularis XP_002399573
Centruroides_sculpturatus XP_023238907
Limulus_polyphemus XP_013775529
Nephila_clavipes PRD23217
Parasteatoda_tepidariorum XP_015930569
Acanthaster_planci XP_022098906
Acanthaster_planci XP_022098910
Acanthaster_planci XP_022098909
Acanthaster_planci XP_022098907
Saccoglossus_kowalevskii XP_002733825
Saccoglossus_kowalevskii XP_006815607
Stylophora_pistillata PFX31828
Symbiodinium_sp._C1 CAMPEP_0201428536
Exaiptasia_pallida XP_020904795
Exaiptasia_pallida XP_020904798
Trichoplax_sp._H2 RDD46272
Trichoplax_adhaerens XP_002111035
Emiliania_huxleyi_CCMP370 CAMPEP_0182404872
Emiliania_huxleyi_CCMP370 CAMPEP_0182368608
Emiliania_huxleyi_CCMP370 CAMPEP_0182285344
Emiliania_huxleyi_379 CAMPEP_0196377118
Emiliania_huxleyi_PLY_M219 CAMPEP_0181831870
Emiliania_huxleyi_PLY_M219 CAMPEP_0181962992
Gephyrocapsa_oceanica_RCC1303 CAMPEP_0185452528
Gephyrocapsa_oceanica_RCC1303 CAMPEP_0185368284
Gephyrocapsa_oceanica_RCC1303 CAMPEP_0185498400
Trichosphaerium_sp._Am-I-7_wt CAMPEP_0168514626
Bigelowiella_natans_CCMP1242 CAMPEP_0185231072
Bigelowiella_natans_CCMP1258.1 CAMPEP_0172224446
Bigelowiella_natans_CCMP1259 CAMPEP_0169608188
Bigelowiella_natans_CCMP_2755 CAMPEP_0114183874
Alexandrium_tamarense CAMPEP_0116451196
Alexandrium_tamarense CAMPEP_0116271062
Eutreptiella_gymnastica_NIES-381 CAMPEP_0113751976
Tetraselmis_striata_LANL1001 CAMPEP_0183913396
Tetraselmis_striata_LANL1001 CAMPEP_0183924776
Tetraselmis_striata_LANL1001 CAMPEP_0183973080
Tetraselmis_striata_LANL1001 CAMPEP_0183837522
Tetraselmis_chuii_PLY429 CAMPEP_0177781564
Tetraselmis_astigmatica_CCMP880 CAMPEP_0117659076
Madagascaria_erythrocladiodes_CCMP3234 CAMPEP_0198315772
Gambierdiscus_australes_CAWD_149 CAMPEP_0171068048
Gymnodinium_catenatum_GC744 CAMPEP_0117518584
Stereomyxa_ramosa_Chinc5 CAMPEP_0174261848
Symbiodinium_sp._Mp CAMPEP_0169722816
Symbiodinium_sp._Mp CAMPEP_0169792352
Symbiodinium_sp._Mp CAMPEP_0169642138
Symbiodinium_sp._Mp CAMPEP_0169871582
Azadinium_spinosum_3D9 CAMPEP_0180469176
Azadinium_spinosum_3D9 CAMPEP_0180766826
Pelagodinium_bii_RCC1491 CAMPEP_0197699940
Symbiodinium_sp._CCMP2430 CAMPEP_0181719338
Symbiodinium_sp._CCMP2430 CAMPEP_0181575682
Symbiodinium_sp._CCMP2430 CAMPEP_0181643290
Symbiodinium_microadriaticum OLP90119
Symbiodinium_sp._CCMP421 CAMPEP_0181444274
Symbiodinium_sp._C1 CAMPEP_0202644466
Symbiodinium_sp._C1 CAMPEP_0202554966
Symbiodinium_sp._C1 CAMPEP_0201337444
Symbiodinium_sp._C1 CAMPEP_0201432616
Symbiodinium_sp._CCMP421 CAMPEP_0181456158
Ammonia_sp._NULL CAMPEP_0197035564
Rosalina_sp._NULL CAMPEP_0201567978
Bolidomonas_pacifica_RCC208 CAMPEP_0182454530
Bolidomonas_sp._RCC2347 CAMPEP_0197556976
Bolidomonas_sp._RCC1657 CAMPEP_0182500618
Bolidomonas_pacifica_CCMP_1866 CAMPEP_0118661470
NonPhotosyntheticDiatom QUERY_NAA03P01590.m1
Perkinsus_marinus_ATCC_50983 XP_002765294
alpha-D-galactosidase/alpha-N-acetylgalactosaminidase GH27
0.2

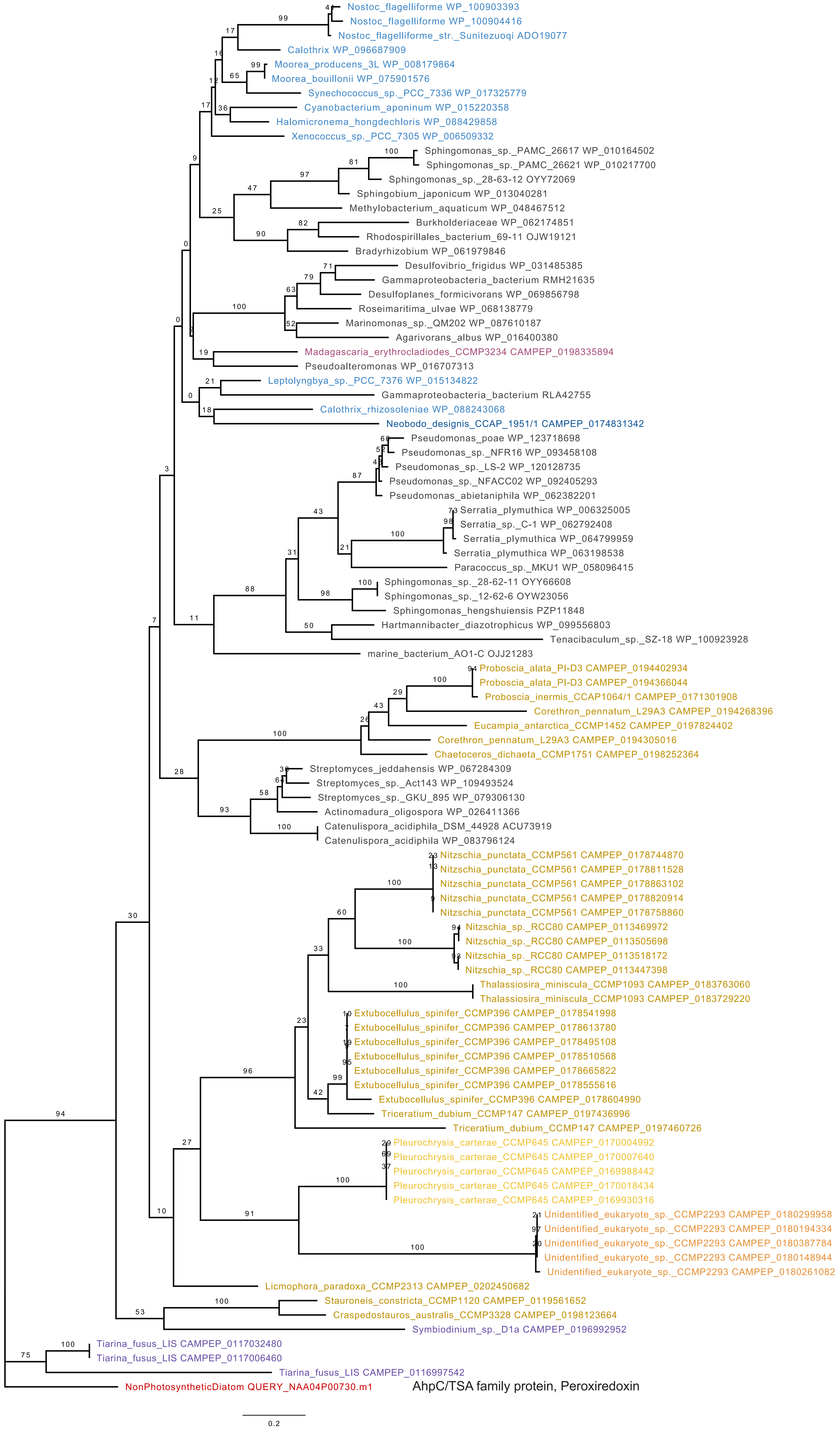
Nostoc_flagelliforme WP_100903393
Nostoc_flagelliforme WP_100904416
Nostoc_flagelliforme_str._Sunitezuoqi ADO19077
Calothrix WP_096687909
Moorea_producens_3L WP_008179864
Moorea_bouillonii WP_075901576
Synechococcus_sp._PCC_7336 WP_017325779
Cyanobacterium_aponinum WP_015220358
Halomicronema_hongdechloris WP_088429858
Xenococcus_sp._PCC_7305 WP_006509332
Sphingomonas_sp._PAMC_26617 WP_010164502
Sphingomonas_sp._PAMC_26621 WP_010217700
Sphingomonas_sp._28-63-12 OYY72069
Sphingobium_japonicum WP_013040281
Methylobacterium_aquaticum WP_048467512
Burkholderiaceae WP_062174851
Rhodospirillales_bacterium_69-11 OJW19121
Bradyrhizobium WP_061979846
Desulfovibrio_frigidus WP_031485385
Gammaproteobacteria_bacterium RMH21635
Desulfoplanes_formicivorans WP_069856798
Roseimaritima_ulvae WP_068138779
Marinomonas_sp._QM202 WP_087610187
Agarivorans_albus WP_016400380
Madagascaria_erythrocladiodes_CCMP3234 CAMPEP_0198335894
Pseudoalteromonas WP_016707313
Leptolyngbya_sp._PCC_7376 WP_015134822
Gammaproteobacteria_bacterium RLA42755
Calothrix_rhizosoleniae WP_088243068
Neobodo_designis_CCAP_1951/1 CAMPEP_0174831342
Pseudomonas_poae WP_123718698
Pseudomonas_sp._NFR16 WP_093458108
Pseudomonas_sp._LS-2 WP_120128735
Pseudomonas_sp._NFACC02 WP_092405293
Pseudomonas_abietaniphila WP_062382201
Serratia_plymuthica WP_006325005
Serratia_sp._C-1 WP_062792408
Serratia_plymuthica WP_064799959
Serratia_plymuthica WP_063198538
Paracoccus_sp._MKU1 WP_058096415
Sphingomonas_sp._28-62-11 OYY66608
Sphingomonas_sp._12-62-6 OYW23056
Sphingomonas_hengshuiensis PZP11848
Hartmannibacter_diazotrophicus WP_099556803
Tenacibaculum_sp._SZ-18 WP_100923928
marine_bacterium_AO1-C OJJ21283
Proboscia_alata_PI-D3 CAMPEP_0194402934
Proboscia_alata_PI-D3 CAMPEP_0194366044
Proboscia_inermis_CCAP1064/1 CAMPEP_0171301908
Corethron_pennatum_L29A3 CAMPEP_0194268396
Eucampia_antarctica_CCMP1452 CAMPEP_0197824402
Corethron_pennatum_L29A3 CAMPEP_0194305016
Chaetoceros_dichaeta_CCMP1751 CAMPEP_0198252364
Streptomyces_jeddahensis WP_067284309
Streptomyces_sp._Act143 WP_109493524
Streptomyces_sp._GKU_895 WP_079306130
Actinomadura_oligospora WP_026411366
Catenulispora_acidiphila_DSM_44928 ACU73919
Catenulispora_acidiphila WP_083796124
Nitzschia_punctata_CCMP561 CAMPEP_0178744870
Nitzschia_punctata_CCMP561 CAMPEP_0178811528
Nitzschia_punctata_CCMP561 CAMPEP_0178863102
Nitzschia_punctata_CCMP561 CAMPEP_0178820914
Nitzschia_punctata_CCMP561 CAMPEP_0178758860
Nitzschia_sp._RCC80 CAMPEP_0113469972
Nitzschia_sp._RCC80 CAMPEP_0113505698
Nitzschia_sp._RCC80 CAMPEP_0113518172
Nitzschia_sp._RCC80 CAMPEP_0113447398
Thalassiosira_miniscula_CCMP1093 CAMPEP_0183763060
Thalassiosira_miniscula_CCMP1093 CAMPEP_0183729220
Extubocellulus_spinifer_CCMP396 CAMPEP_0178541998
Extubocellulus_spinifer_CCMP396 CAMPEP_0178613780
Extubocellulus_spinifer_CCMP396 CAMPEP_0178495108
Extubocellulus_spinifer_CCMP396 CAMPEP_0178510568
Extubocellulus_spinifer_CCMP396 CAMPEP_0178665822
Extubocellulus_spinifer_CCMP396 CAMPEP_0178555616
Extubocellulus_spinifer_CCMP396 CAMPEP_0178604990
Triceratium_dubium_CCMP147 CAMPEP_0197436996
Triceratium_dubium_CCMP147 CAMPEP_0197460726
Pleurochrysis_carterae_CCMP645 CAMPEP_0170004992
Pleurochrysis_carterae_CCMP645 CAMPEP_0170007640
Pleurochrysis_carterae_CCMP645 CAMPEP_0169988442
Pleurochrysis_carterae_CCMP645 CAMPEP_0170018434
Pleurochrysis_carterae_CCMP645 CAMPEP_0169930316
Unidentified_eukaryote_sp._CCMP2293 CAMPEP_0180299958
Unidentified_eukaryote_sp._CCMP2293 CAMPEP_0180194334
Unidentified_eukaryote_sp._CCMP2293 CAMPEP_0180387784
Unidentified_eukaryote_sp._CCMP2293 CAMPEP_0180148944
Unidentified_eukaryote_sp._CCMP2293 CAMPEP_0180261082
Licmophora_paradoxa_CCMP2313 CAMPEP_0202450682
Stauroneis_constricta_CCMP1120 CAMPEP_0119561652
Craspedostauros_australis_CCMP3328 CAMPEP_0198123664
Symbiodinium_sp._D1a CAMPEP_0196992952
Tiarina_fusus_LIS CAMPEP_0117032480
Tiarina_fusus_LIS CAMPEP_0117006460
Tiarina_fusus_LIS CAMPEP_0116997542
NonPhotosyntheticDiatom QUERY_NAA04P00730.m1
AhpC/TSA family protein, Peroxiredoxin
0.2

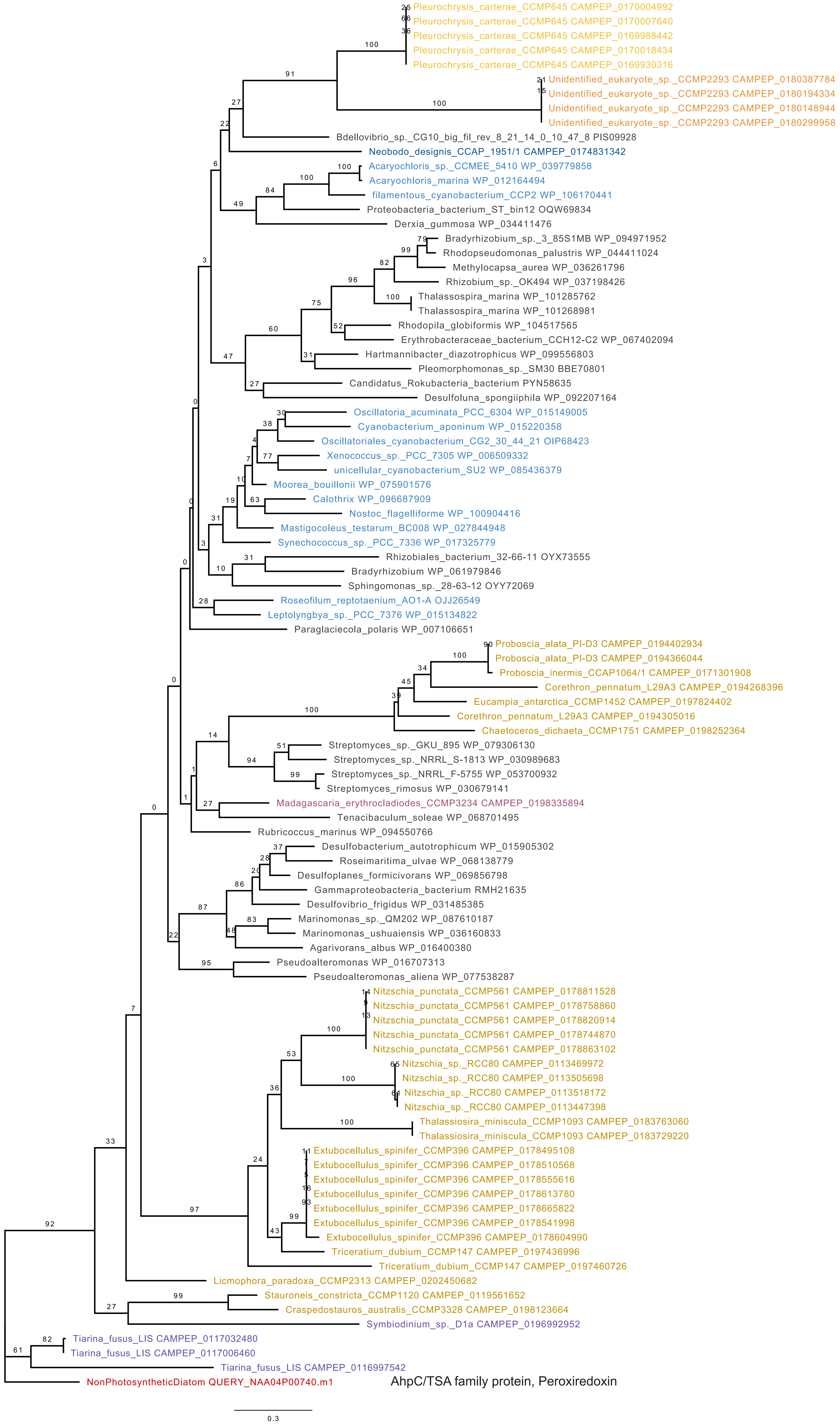
Pleurochrysis_carterae_CCMP645 CAMPEP_0170004992
Pleurochrysis_carterae_CCMP645 CAMPEP_0170007640
Pleurochrysis_carterae_CCMP645 CAMPEP_0169988442
Pleurochrysis_carterae_CCMP645 CAMPEP_0170018434
Pleurochrysis_carterae_CCMP645 CAMPEP_0169930316
Unidentified_eukaryote_sp._CCMP2293 CAMPEP_0180387784
Unidentified_eukaryote_sp._CCMP2293 CAMPEP_0180194334
Unidentified_eukaryote_sp._CCMP2293 CAMPEP_0180148944
Unidentified_eukaryote_sp._CCMP2293 CAMPEP_0180299958
Bdellovibrio_sp._CG10_big_fil_rev_8_21_14_0_10_47_8 PIS09928
Neobodo_designis_CCAP_1951/1 CAMPEP_0174831342
Acaryochloris_sp._CCMEE_5410 WP_039779858
Acaryochloris_marina WP_012164494
filamentous_cyanobacterium_CCP2 WP_106170441
Proteobacteria_bacterium_ST_bin12 OQW69834
Derxia_gummosa WP_034411476
Bradyrhizobium_sp._3_85S1MB WP_094971952
Rhodopseudomonas_palustris WP_044411024
Methylocapsa_aurea WP_036261796
Rhizobium_sp._OK494 WP_037198426
Thalassospira_marina WP_101285762
Thalassospira_marina WP_101268981
Rhodopila_globiformis WP_104517565
Erythrobacteraceae_bacterium_CCH12-C2 WP_067402094
Hartmannibacter_diazotrophicus WP_099556803
Pleomorphomonas_sp._SM30 BBE70801
Candidatus_Rokubacteria_bacterium PYN58635
Desulfoluna_spongiiphila WP_092207164
Oscillatoria_acuminata_PCC_6304 WP_015149005
Cyanobacterium_aponinum WP_015220358
Oscillatoriales_cyanobacterium_CG2_30_44_21 OIP68423
Xenococcus_sp._PCC_7305 WP_006509332
unicellular_cyanobacterium_SU2 WP_085436379
Moorea_bouillonii WP_075901576
Calothrix WP_096687909
Nostoc_flagelliforme WP_100904416
Mastigocoleus_testarum_BC008 WP_027844948
Synechococcus_sp._PCC_7336 WP_017325779
Rhizobiales_bacterium_32-66-11 OYX73555
Bradyrhizobium WP_061979846
Sphingomonas_sp._28-63-12 OYY72069
Roseofilum_reptotaenium_AO1-A OJJ26549
Leptolyngbya_sp._PCC_7376 WP_015134822
Paraglaciecola_polaris WP_007106651
Proboscia_alata_PI-D3 CAMPEP_0194402934
Proboscia_alata_PI-D3 CAMPEP_0194366044
Proboscia_inermis_CCAP1064/1 CAMPEP_0171301908
Corethron_pennatum_L29A3 CAMPEP_0194268396
Eucampia_antarctica_CCMP1452 CAMPEP_0197824402
Corethron_pennatum_L29A3 CAMPEP_0194305016
Chaetoceros_dichaeta_CCMP1751 CAMPEP_0198252364
Streptomyces_sp._GKU_895 WP_079306130
Streptomyces_sp._NRRL_S-1813 WP_030989683
Streptomyces_sp._NRRL_F-5755 WP_053700932
Streptomyces_rimosus WP_030679141
Madagascaria_erythrocladiodes_CCMP3234 CAMPEP_0198335894
Tenacibaculum_soleae WP_068701495
Rubricoccus_marinus WP_094550766
Desulfobacterium_autotrophicum WP_015905302
Roseimaritima_ulvae WP_068138779
Desulfoplanes_formicivorans WP_069856798
Gammaproteobacteria_bacterium RMH21635
Desulfovibrio_frigidus WP_031485385
Marinomonas_sp._QM202 WP_087610187
Marinomonas_ushuaiensis WP_036160833
Agarivorans_albus WP_016400380
Pseudoalteromonas WP_016707313
Pseudoalteromonas_aliena WP_077538287
Nitzschia_punctata_CCMP561 CAMPEP_0178811528
Nitzschia_punctata_CCMP561 CAMPEP_0178758860
Nitzschia_punctata_CCMP561 CAMPEP_0178820914
Nitzschia_punctata_CCMP561 CAMPEP_0178744870
Nitzschia_punctata_CCMP561 CAMPEP_0178863102
Nitzschia_sp._RCC80 CAMPEP_0113469972
Nitzschia_sp._RCC80 CAMPEP_0113505698
Nitzschia_sp._RCC80 CAMPEP_0113518172
Nitzschia_sp._RCC80 CAMPEP_0113447398
Thalassiosira_miniscula_CCMP1093 CAMPEP_0183763060
Thalassiosira_miniscula_CCMP1093 CAMPEP_0183729220
Extubocellulus_spinifer_CCMP396 CAMPEP_0178495108
Extubocellulus_spinifer_CCMP396 CAMPEP_0178510568
Extubocellulus_spinifer_CCMP396 CAMPEP_0178555616
Extubocellulus_spinifer_CCMP396 CAMPEP_0178613780
Extubocellulus_spinifer_CCMP396 CAMPEP_0178665822
Extubocellulus_spinifer_CCMP396 CAMPEP_0178541998
Extubocellulus_spinifer_CCMP396 CAMPEP_0178604990
Triceratium_dubium_CCMP147 CAMPEP_0197436996
Triceratium_dubium_CCMP147 CAMPEP_0197460726
Licmophora_paradoxa_CCMP2313 CAMPEP_0202450682
Stauroneis_constricta_CCMP1120 CAMPEP_0119561652
Craspedostauros_australis_CCMP3328 CAMPEP_0198123664
Symbiodinium_sp._D1a CAMPEP_0196992952
Tiarina_fusus_LIS CAMPEP_0117032480
Tiarina_fusus_LIS CAMPEP_0117006460
Tiarina_fusus_LIS CAMPEP_0116997542
NonPhotosyntheticDiatom QUERY_NAA04P00740.m1
AhpC/TSA family protein, Peroxiredoxin
0.3

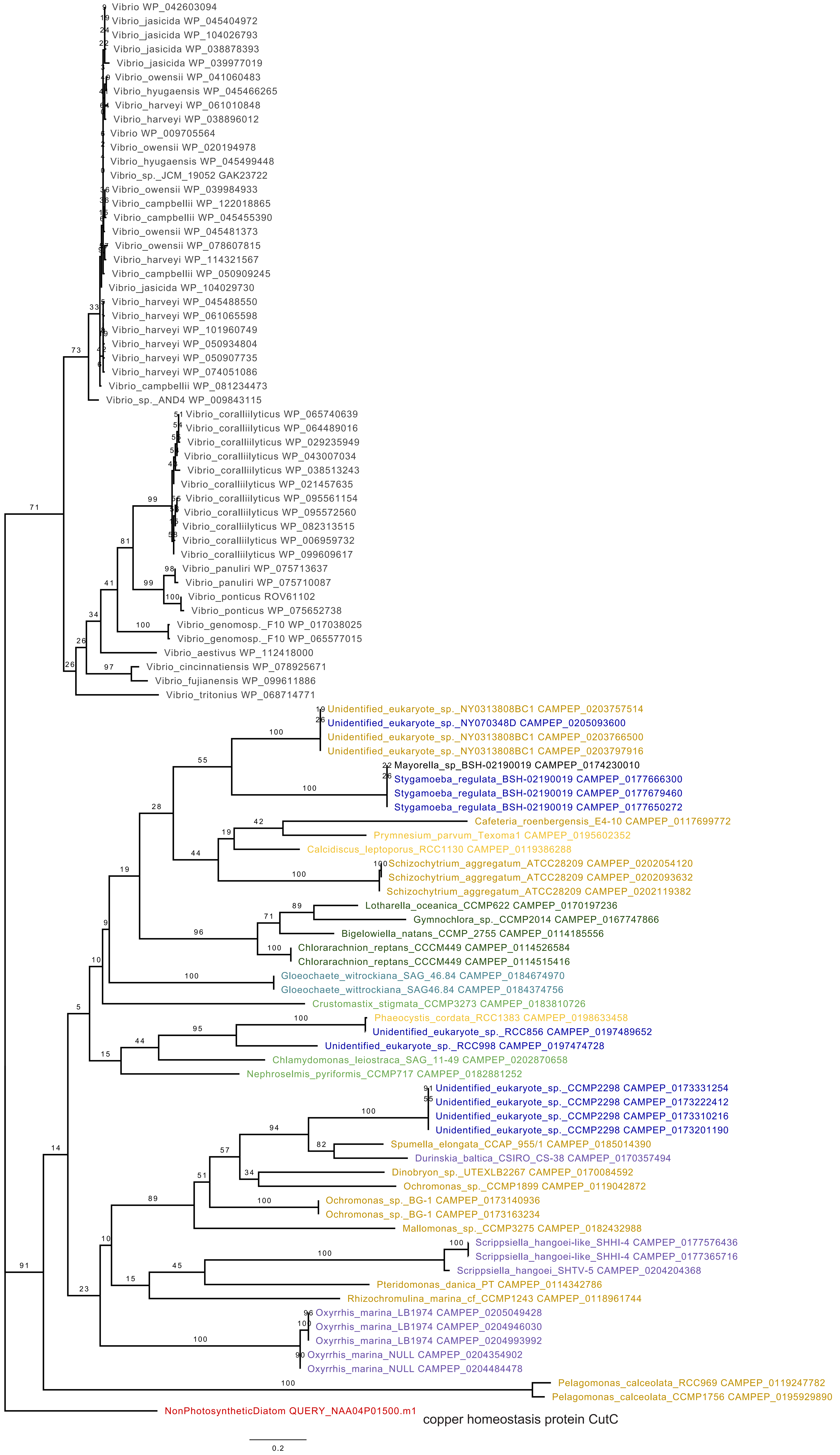
Vibrio WP_042603094
Vibrio_jasicida WP_045404972
Vibrio_jasicida WP_104026793
Vibrio_jasicida WP_038878393
Vibrio_jasicida WP_039977019
Vibrio_owensii WP_041060483
Vibrio_hyugaensis WP_045466265
Vibrio_harveyi WP_061010848
Vibrio_harveyi WP_038896012
Vibrio WP_009705564
Vibrio_owensii WP_020194978
Vibrio_hyugaensis WP_045499448
Vibrio_sp._JCM_19052 GAK23722
Vibrio_owensii WP_039984933
Vibrio_campbellii WP_122018865
Vibrio_campbellii WP_045455390
Vibrio_owensii WP_045481373
Vibrio_owensii WP_078607815
Vibrio_harveyi WP_114321567
Vibrio_campbellii WP_050909245
Vibrio_jasicida WP_104029730
Vibrio_harveyi WP_045488550
Vibrio_harveyi WP_061065598
Vibrio_harveyi WP_101960749
Vibrio_harveyi WP_050934804
Vibrio_harveyi WP_050907735
Vibrio_harveyi WP_074051086
Vibrio_campbellii WP_081234473
Vibrio_sp._AND4 WP_009843115
Vibrio_coralliilyticus WP_065740639
Vibrio_coralliilyticus WP_064489016
Vibrio_coralliilyticus WP_029235949
Vibrio_coralliilyticus WP_043007034
Vibrio_coralliilyticus WP_038513243
Vibrio_coralliilyticus WP_021457635
Vibrio_coralliilyticus WP_095561154
Vibrio_coralliilyticus WP_095572560
Vibrio_coralliilyticus WP_082313515
Vibrio_coralliilyticus WP_006959732
Vibrio_coralliilyticus WP_099609617
Vibrio_panuliri WP_075713637
Vibrio_panuliri WP_075710087
Vibrio_ponticus ROV61102
Vibrio_ponticus WP_075652738
Vibrio_genomosp._F10 WP_017038025
Vibrio_genomosp._F10 WP_065577015
Vibrio_aestivus WP_112418000
Vibrio_cincinnatiensis WP_078925671
Vibrio_fujianensis WP_099611886
Vibrio_tritonius WP_068714771
Unidentified_eukaryote_sp._NY0313808BC1 CAMPEP_0203757514
Unidentified_eukaryote_sp._NY070348D CAMPEP_0205093600
Unidentified_eukaryote_sp._NY0313808BC1 CAMPEP_0203766500
Unidentified_eukaryote_sp._NY0313808BC1 CAMPEP_0203797916
Mayorella_sp_BSH-02190019 CAMPEP_0174230010
Stygamoeba_regulata_BSH-02190019 CAMPEP_0177666300
Stygamoeba_regulata_BSH-02190019 CAMPEP_0177679460
Stygamoeba_regulata_BSH-02190019 CAMPEP_0177650272
Cafeteria_roenbergensis_E4-10 CAMPEP_0117699772
Prymnesium_parvum_Texoma1 CAMPEP_0195602352
Calcidiscus_leptoporus_RCC1130 CAMPEP_0119386288
Schizochytrium_aggregatum_ATCC28209 CAMPEP_0202054120
Schizochytrium_aggregatum_ATCC28209 CAMPEP_0202093632
Schizochytrium_aggregatum_ATCC28209 CAMPEP_0202119382
Lotharella_oceanica_CCMP622 CAMPEP_0170197236
Gymnochlora_sp._CCMP2014 CAMPEP_0167747866
Bigelowiella_natans_CCMP_2755 CAMPEP_0114185556
Chlorarachnion_reptans_CCCM449 CAMPEP_0114526584
Chlorarachnion_reptans_CCCM449 CAMPEP_0114515416
Gloeochaete_wittrockiana_SAG_46.84 CAMPEP_0184674970
Gloeochaete_wittrockiana_SAG46.84 CAMPEP_0184374756
Crustomastix_stigmata_CCMP3273 CAMPEP_0183810726
Phaeocystis_cordata_RCC1383 CAMPEP_0198633458
Unidentified_eukaryote_sp._RCC856 CAMPEP_0197489652
Unidentified_eukaryote_sp._RCC998 CAMPEP_0197474728
Chlamydomonas_leiostraca_SAG_11-49 CAMPEP_0202870658
Nephroselmis_pyriformis_CCMP717 CAMPEP_0182881252
Unidentified_eukaryote_sp._CCMP2298 CAMPEP_0173331254
Unidentified_eukaryote_sp._CCMP2298 CAMPEP_0173222412
Unidentified_eukaryote_sp._CCMP2298 CAMPEP_0173310216
Unidentified_eukaryote_sp._CCMP2298 CAMPEP_0173201190
Spumella_elongata_CCAP_955/1 CAMPEP_0185014390
Durinskia_baltica_CSIRO_CS-38 CAMPEP_0170357494
Dinobryon_sp._UTEXLB2267 CAMPEP_0170084592
Ochromonas_sp._CCMP1899 CAMPEP_0119042872
Ochromonas_sp._BG-1 CAMPEP_0173140936
Ochromonas_sp._BG-1 CAMPEP_0173163234
Mallomonas_sp._CCMP3275 CAMPEP_0182432988
Scrippsiella_hangoei-like_SHHI-4 CAMPEP_0177576436
Scrippsiella_hangoei-like_SHHI-4 CAMPEP_0177365716
Scrippsiella_hangoei_SHTV-5 CAMPEP_0204204368
Pteridomonas_danica_PT CAMPEP_0114342786
Rhizochromulina_marina_cf_CCMP1243 CAMPEP_0118961744
Oxyrrhis_marina_LB1974 CAMPEP_0205049428
Oxyrrhis_marina_LB1974 CAMPEP_0204946030
Oxyrrhis_marina_LB1974 CAMPEP_0204993992
Oxyrrhis_marina_NULL CAMPEP_0204354902
Oxyrrhis_marina_NULL CAMPEP_0204484478
Pelagomonas_calceolata_RCC969 CAMPEP_0119247782
Pelagomonas_calceolata_CCMP1756 CAMPEP_0195929890
NonPhotosyntheticDiatom QUERY_NAA04P01500.m1
copper homeostasis protein CutC
0.2

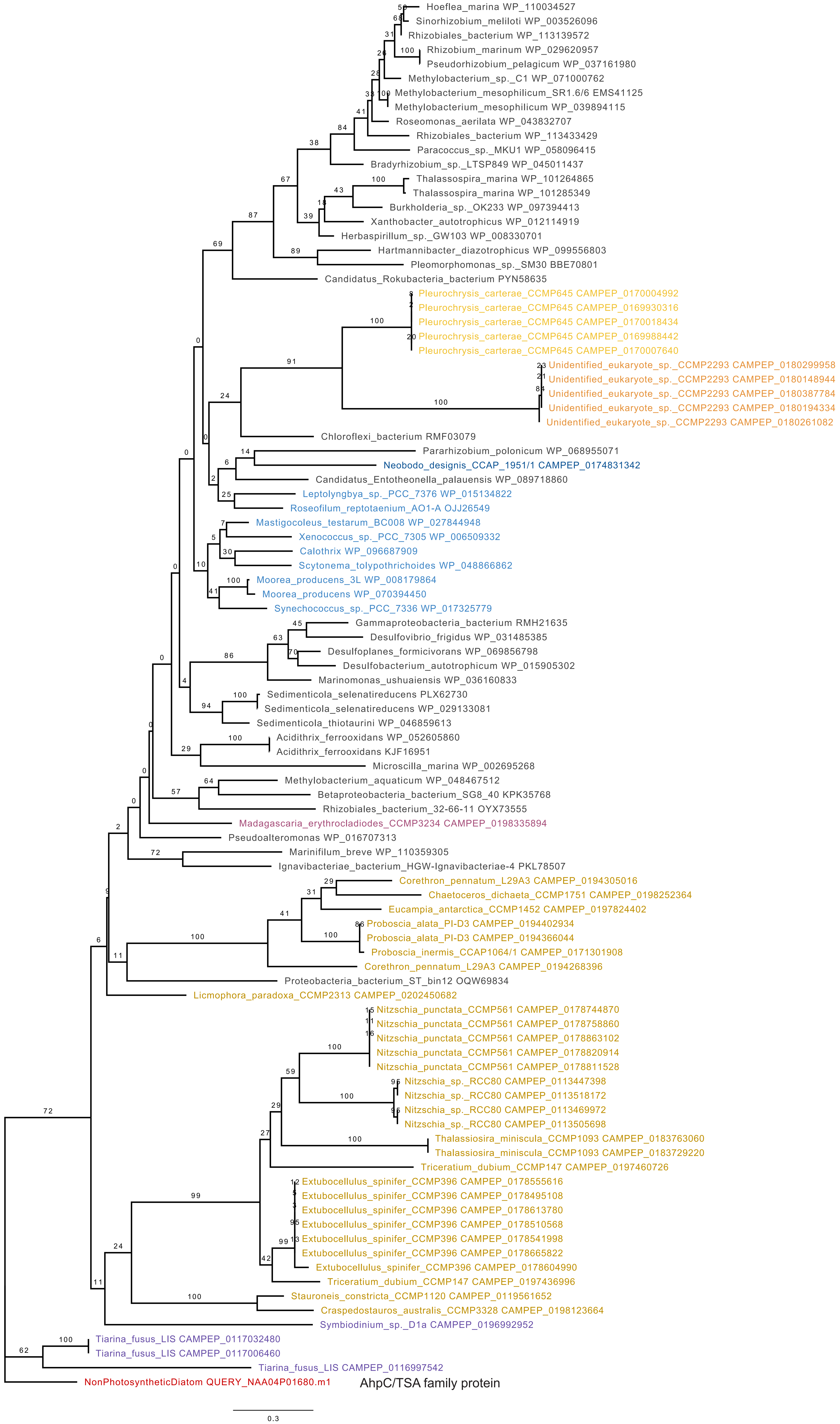
Hoeflea_marina WP_110034527
Sinorhizobium_meliloti WP_003526096
Rhizobiales_bacterium WP_113139572
Rhizobium_marinum WP_029620957
Pseudorhizobium_pelagicum WP_037161980
Methylobacterium_sp._C1 WP_071000762
Methylobacterium_mesophilicum_SR1.6/6 EMS41125
Methylobacterium_mesophilicum WP_039894115
Roseomonas_aerilata WP_043832707
Rhizobiales_bacterium WP_113433429
Paracoccus_sp._MKU1 WP_058096415
Bradyrhizobium_sp._LTSP849 WP_045011437
Thalassospira_marina WP_101264865
Thalassospira_marina WP_101285349
Burkholderia_sp._OK233 WP_097394413
Xanthobacter_autotrophicus WP_012114919
Herbaspirillum_sp._GW103 WP_008330701
Hartmannibacter_diazotrophicus WP_099556803
Pleomorphomonas_sp._SM30 BBE70801
Candidatus_Rokubacteria_bacterium PYN58635
Pleurochrysis_carterae_CCMP645 CAMPEP_0170004992
Pleurochrysis_carterae_CCMP645 CAMPEP_0169930316
Pleurochrysis_carterae_CCMP645 CAMPEP_0170018434
Pleurochrysis_carterae_CCMP645 CAMPEP_0169988442
Pleurochrysis_carterae_CCMP645 CAMPEP_0170007640
Unidentified_eukaryote_sp._CCMP2293 CAMPEP_0180299958
Unidentified_eukaryote_sp._CCMP2293 CAMPEP_0180148944
Unidentified_eukaryote_sp._CCMP2293 CAMPEP_0180387784
Unidentified_eukaryote_sp._CCMP2293 CAMPEP_0180194334
Unidentified_eukaryote_sp._CCMP2293 CAMPEP_0180261082
Chloroflexi_bacterium RMF03079
Pararhizobium_polonicum WP_068955071
Neobodo_designis_CCAP_1951/1 CAMPEP_0174831342
Candidatus_Entotheonella_palauensis WP_089718860
Leptolyngbya_sp._PCC_7376 WP_015134822
Roseofilum_reptotaenium_AO1-A OJJ26549
Mastigocoleus_testarum_BC008 WP_027844948
Xenococcus_sp._PCC_7305 WP_006509332
Calothrix WP_096687909
Scytonema_tolypothrichoides WP_048866862
Moorea_producens_3L WP_008179864
Moorea_producens WP_070394450
Synechococcus_sp._PCC_7336 WP_017325779
Gammaproteobacteria_bacterium RMH21635
Desulfovibrio_frigidus WP_031485385
Desulfoplanes_formicivorans WP_069856798
Desulfobacterium_autotrophicum WP_015905302
Marinomonas_ushuaiensis WP_036160833
Sedimenticola_selenatireducens PLX62730
Sedimenticola_selenatireducens WP_029133081
Sedimenticola_thiotaurini WP_046859613
Acidithrix_ferrooxidans WP_052605860
Acidithrix_ferrooxidans KJF16951
Microscilla_marina WP_002695268
Methylobacterium_aquaticum WP_048467512
Betaproteobacteria_bacterium_SG8_40 KPK35768
Rhizobiales_bacterium_32-66-11 OYX73555
Madagascaria_erythrocladiodes_CCMP3234 CAMPEP_0198335894
Pseudoalteromonas WP_016707313
Marinifilum_breve WP_110359305
Ignavibacteriae_bacterium_HGW-Ignavibacteriae-4 PKL78507
Corethron_pennatum_L29A3 CAMPEP_0194305016
Chaetoceros_dichaeta_CCMP1751 CAMPEP_0198252364
Eucampia_antarctica_CCMP1452 CAMPEP_0197824402
Proboscia_alata_PI-D3 CAMPEP_0194402934
Proboscia_alata_PI-D3 CAMPEP_0194366044
Proboscia_inermis_CCAP1064/1 CAMPEP_0171301908
Corethron_pennatum_L29A3 CAMPEP_0194268396
Proteobacteria_bacterium_ST_bin12 OQW69834
Licmophora_paradoxa_CCMP2313 CAMPEP_0202450682
Nitzschia_punctata_CCMP561 CAMPEP_0178744870
Nitzschia_punctata_CCMP561 CAMPEP_0178758860
Nitzschia_punctata_CCMP561 CAMPEP_0178863102
Nitzschia_punctata_CCMP561 CAMPEP_0178820914
Nitzschia_punctata_CCMP561 CAMPEP_0178811528
Nitzschia_sp._RCC80 CAMPEP_0113447398
Nitzschia_sp._RCC80 CAMPEP_0113518172
Nitzschia_sp._RCC80 CAMPEP_0113469972
Nitzschia_sp._RCC80 CAMPEP_0113505698
Thalassiosira_miniscula_CCMP1093 CAMPEP_0183763060
Thalassiosira_miniscula_CCMP1093 CAMPEP_0183729220
Triceratium_dubium_CCMP147 CAMPEP_0197460726
Extubocellulus_spinifer_CCMP396 CAMPEP_0178555616
Extubocellulus_spinifer_CCMP396 CAMPEP_0178495108
Extubocellulus_spinifer_CCMP396 CAMPEP_0178613780
Extubocellulus_spinifer_CCMP396 CAMPEP_0178510568
Extubocellulus_spinifer_CCMP396 CAMPEP_0178541998
Extubocellulus_spinifer_CCMP396 CAMPEP_0178665822
Extubocellulus_spinifer_CCMP396 CAMPEP_0178604990
Triceratium_dubium_CCMP147 CAMPEP_0197436996
Stauroneis_constricta_CCMP1120 CAMPEP_0119561652
Craspedostauros_australis_CCMP3328 CAMPEP_0198123664
Symbiodinium_sp._D1a CAMPEP_0196992952
Tiarina_fusus_LIS CAMPEP_0117032480
Tiarina_fusus_LIS CAMPEP_0117006460
Tiarina_fusus_LIS CAMPEP_0116997542
NonPhotosyntheticDiatom QUERY_NAA04P01680.m1
AhpC/TSA family protein
0.3

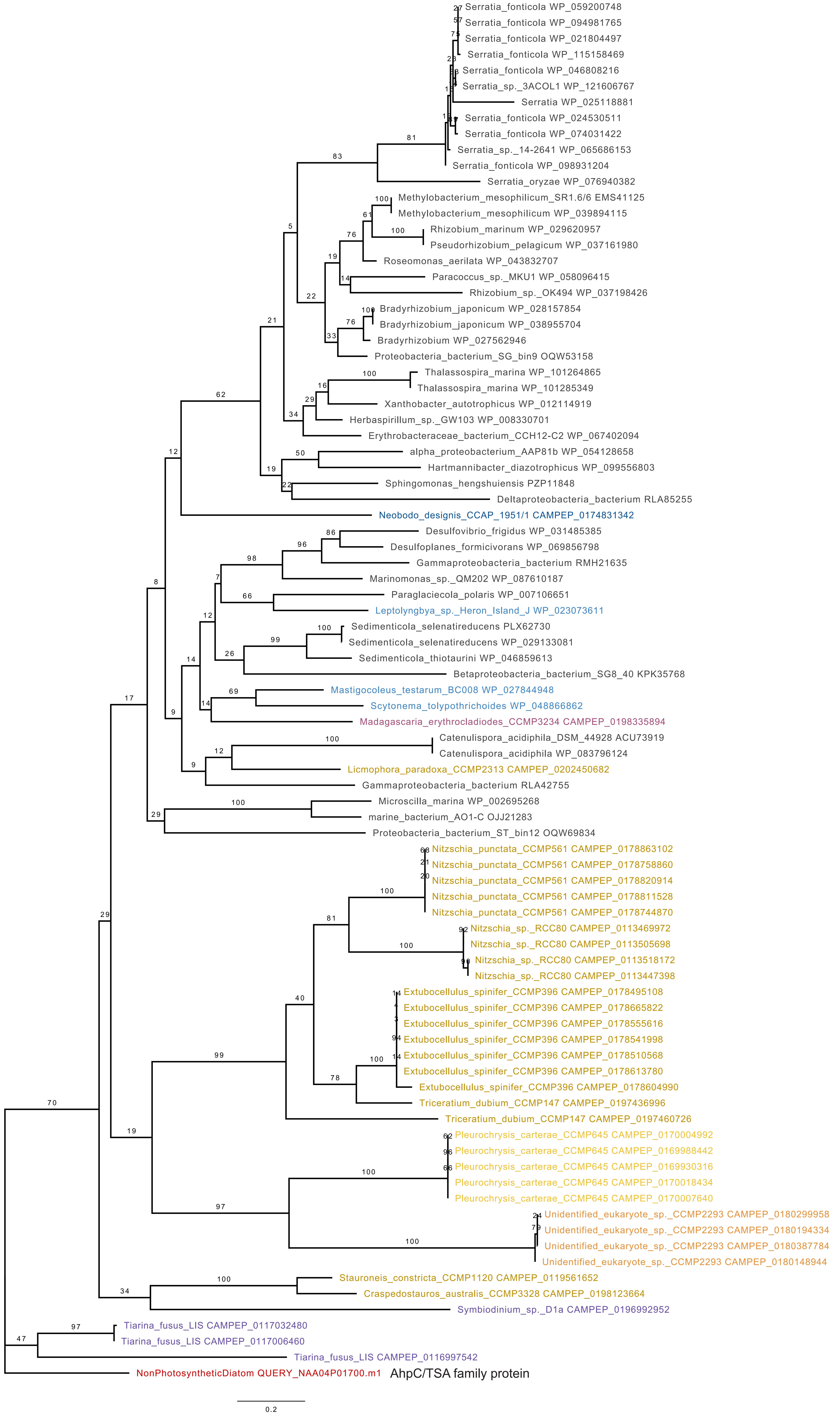
Serratia_fonticola WP_059200748
Serratia_fonticola WP_094981765
Serratia_fonticola WP_021804497
Serratia_fonticola WP_115158469
Serratia_fonticola WP_046808216
Serratia_sp._3ACOL1 WP_121606767
Serratia WP_025118881
Serratia_fonticola WP_024530511
Serratia_fonticola WP_074031422
Serratia_sp._14-2641 WP_065686153
Serratia_fonticola WP_098931204
Serratia_oryzae WP_076940382
Methylobacterium_mesophilicum_SR1.6/6 EMS41125
Methylobacterium_mesophilicum WP_039894115
Rhizobium_marinum WP_029620957
Pseudorhizobium_pelagicum WP_037161980
Roseomonas_aerilata WP_043832707
Paracoccus_sp._MKU1 WP_058096415
Rhizobium_sp._OK494 WP_037198426
Bradyrhizobium_japonicum WP_028157854
Bradyrhizobium_japonicum WP_038955704
Bradyrhizobium WP_027562946
Proteobacteria_bacterium_SG_bin9 OQW53158
Thalassospira_marina WP_101264865
Thalassospira_marina WP_101285349
Xanthobacter_autotrophicus WP_012114919
Herbaspirillum_sp._GW103 WP_008330701
Erythrobacteraceae_bacterium_CCH12-C2 WP_067402094
alpha_proteobacterium_AAP81b WP_054128658
Hartmannibacter_diazotrophicus WP_099556803
Sphingomonas_hengshuiensis PZP11848
Deltaproteobacteria_bacterium RLA85255
Neobodo_designis_CCAP_1951/1 CAMPEP_0174831342
Desulfovibrio_frigidus WP_031485385
Desulfoplanes_formicivorans WP_069856798
Gammaproteobacteria_bacterium RMH21635
Marinomonas_sp._QM202 WP_087610187
Paraglaciecola_polaris WP_007106651
Leptolyngbya_sp._Heron_Island_J WP_023073611
Sedimenticola_selenatireducens PLX62730
Sedimenticola_selenatireducens WP_029133081
Sedimenticola_thiotaurini WP_046859613
Betaproteobacteria_bacterium_SG8_40 KPK35768
Mastigocoleus_testarum_BC008 WP_027844948
Scytonema_tolypothrichoides WP_048866862
Madagascaria_erythrocladiodes_CCMP3234 CAMPEP_0198335894
Catenulispora_acidiphila_DSM_44928 ACU73919
Catenulispora_acidiphila WP_083796124
Licmophora_paradoxa_CCMP2313 CAMPEP_0202450682
Gammaproteobacteria_bacterium RLA42755
Microscilla_marina WP_002695268
marine_bacterium_AO1-C OJJ21283
Proteobacteria_bacterium_ST_bin12 OQW69834
Nitzschia_punctata_CCMP561 CAMPEP_0178863102
Nitzschia_punctata_CCMP561 CAMPEP_0178758860
Nitzschia_punctata_CCMP561 CAMPEP_0178820914
Nitzschia_punctata_CCMP561 CAMPEP_0178811528
Nitzschia_punctata_CCMP561 CAMPEP_0178744870
Nitzschia_sp._RCC80 CAMPEP_0113469972
Nitzschia_sp._RCC80 CAMPEP_0113505698
Nitzschia_sp._RCC80 CAMPEP_0113518172
Nitzschia_sp._RCC80 CAMPEP_0113447398
Extubocellulus_spinifer_CCMP396 CAMPEP_0178495108
Extubocellulus_spinifer_CCMP396 CAMPEP_0178665822
Extubocellulus_spinifer_CCMP396 CAMPEP_0178555616
Extubocellulus_spinifer_CCMP396 CAMPEP_0178541998
Extubocellulus_spinifer_CCMP396 CAMPEP_0178510568
Extubocellulus_spinifer_CCMP396 CAMPEP_0178613780
Extubocellulus_spinifer_CCMP396 CAMPEP_0178604990
Triceratium_dubium_CCMP147 CAMPEP_0197436996
Triceratium_dubium_CCMP147 CAMPEP_0197460726
Pleurochrysis_carterae_CCMP645 CAMPEP_0170004992
Pleurochrysis_carterae_CCMP645 CAMPEP_0169988442
Pleurochrysis_carterae_CCMP645 CAMPEP_0169930316
Pleurochrysis_carterae_CCMP645 CAMPEP_0170018434
Pleurochrysis_carterae_CCMP645 CAMPEP_0170007640
Unidentified_eukaryote_sp._CCMP2293 CAMPEP_0180299958
Unidentified_eukaryote_sp._CCMP2293 CAMPEP_0180194334
Unidentified_eukaryote_sp._CCMP2293 CAMPEP_0180387784
Unidentified_eukaryote_sp._CCMP2293 CAMPEP_0180148944
Stauroneis_constricta_CCMP1120 CAMPEP_0119561652
Craspedostauros_australis_CCMP3328 CAMPEP_0198123664
Symbiodinium_sp._D1a CAMPEP_0196992952
Tiarina_fusus_LIS CAMPEP_0117032480
Tiarina_fusus_LIS CAMPEP_0117006460
Tiarina_fusus_LIS CAMPEP_0116997542
NonPhotosyntheticDiatom QUERY_NAA04P01700.m1
AhpC/TSA family protein
0.2

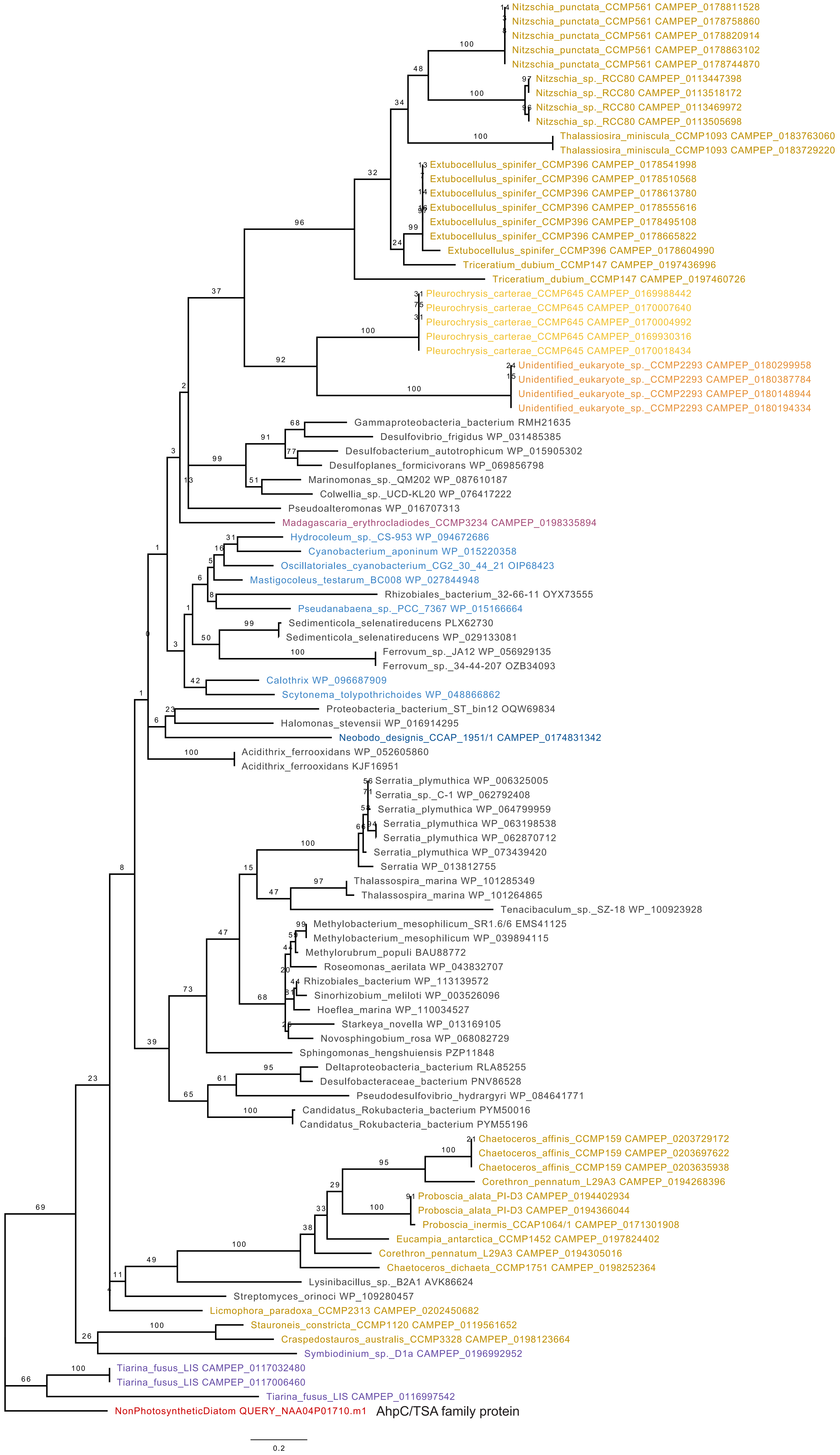
Nitzschia_punctata_CCMP561 CAMPEP_0178811528
Nitzschia_punctata_CCMP561 CAMPEP_0178758860
Nitzschia_punctata_CCMP561 CAMPEP_0178820914
Nitzschia_punctata_CCMP561 CAMPEP_0178863102
Nitzschia_punctata_CCMP561 CAMPEP_0178744870
Nitzschia_sp._RCC80 CAMPEP_0113447398
Nitzschia_sp._RCC80 CAMPEP_0113518172
Nitzschia_sp._RCC80 CAMPEP_0113469972
Nitzschia_sp._RCC80 CAMPEP_0113505698
Thalassiosira_miniscula_CCMP1093 CAMPEP_0183763060
Thalassiosira_miniscula_CCMP1093 CAMPEP_0183729220
Extubocellulus_spinifer_CCMP396 CAMPEP_0178541998
Extubocellulus_spinifer_CCMP396 CAMPEP_0178510568
Extubocellulus_spinifer_CCMP396 CAMPEP_0178613780
Extubocellulus_spinifer_CCMP396 CAMPEP_0178555616
Extubocellulus_spinifer_CCMP396 CAMPEP_0178495108
Extubocellulus_spinifer_CCMP396 CAMPEP_0178665822
Extubocellulus_spinifer_CCMP396 CAMPEP_0178604990
Triceratium_dubium_CCMP147 CAMPEP_0197436996
Triceratium_dubium_CCMP147 CAMPEP_0197460726
Pleurochrysis_carterae_CCMP645 CAMPEP_0169988442
Pleurochrysis_carterae_CCMP645 CAMPEP_0170007640
Pleurochrysis_carterae_CCMP645 CAMPEP_0170004992
Pleurochrysis_carterae_CCMP645 CAMPEP_0169930316
Pleurochrysis_carterae_CCMP645 CAMPEP_0170018434
Unidentified_eukaryote_sp._CCMP2293 CAMPEP_0180299958
Unidentified_eukaryote_sp._CCMP2293 CAMPEP_0180387784
Unidentified_eukaryote_sp._CCMP2293 CAMPEP_0180148944
Unidentified_eukaryote_sp._CCMP2293 CAMPEP_0180194334
Gammaproteobacteria_bacterium RMH21635
Desulfovibrio_frigidus WP_031485385
Desulfobacterium_autotrophicum WP_015905302
Desulfoplanes_formicivorans WP_069856798
Marinomonas_sp._QM202 WP_087610187
Colwellia_sp._UCD-KL20 WP_076417222
Pseudoalteromonas WP_016707313
Madagascaria_erythrocladiodes_CCMP3234 CAMPEP_0198335894
Hydrocoleum_sp._CS-953 WP_094672686
Cyanobacterium_aponinum WP_015220358
Oscillatoriales_cyanobacterium_CG2_30_44_21 OIP68423
Mastigocoleus_testarum_BC008 WP_027844948
Rhizobiales_bacterium_32-66-11 OYX73555
Pseudanabaena_sp._PCC_7367 WP_015166664
Sedimenticola_selenatireducens PLX62730
Sedimenticola_selenatireducens WP_029133081
Ferrovum_sp._JA12 WP_056929135
Ferrovum_sp._34-44-207 OZB34093
Calothrix WP_096687909
Scytonema_tolypothrichoides WP_048866862
Proteobacteria_bacterium_ST_bin12 OQW69834
Halomonas_stevensii WP_016914295
Neobodo_designis_CCAP_1951/1 CAMPEP_0174831342
Acidithrix_ferrooxidans WP_052605860
Acidithrix_ferrooxidans KJF16951
Serratia_plymuthica WP_006325005
Serratia_sp._C-1 WP_062792408
Serratia_plymuthica WP_064799959
Serratia_plymuthica WP_063198538
Serratia_plymuthica WP_062870712
Serratia_plymuthica WP_073439420
Serratia WP_013812755
Thalassospira_marina WP_101285349
Thalassospira_marina WP_101264865
Tenacibaculum_sp._SZ-18 WP_100923928
Methylobacterium_mesophilicum_SR1.6/6 EMS41125
Methylobacterium_mesophilicum WP_039894115
Methylorubrum_populi BAU88772
Roseomonas_aerilata WP_043832707
Rhizobiales_bacterium WP_113139572
Sinorhizobium_meliloti WP_003526096
Hoeflea_marina WP_110034527
Starkeya_novella WP_013169105
Novosphingobium_rosa WP_068082729
Sphingomonas_hengshuiensis PZP11848
Deltaproteobacteria_bacterium RLA85255
Desulfobacteraceae_bacterium PNV86528
Pseudodesulfovibrio_hydrargyri WP_084641771
Candidatus_Rokubacteria_bacterium PYM50016
Candidatus_Rokubacteria_bacterium PYM55196
Chaetoceros_affinis_CCMP159 CAMPEP_0203729172
Chaetoceros_affinis_CCMP159 CAMPEP_0203697622
Chaetoceros_affinis_CCMP159 CAMPEP_0203635938
Corethron_pennatum_L29A3 CAMPEP_0194268396
Proboscia_alata_PI-D3 CAMPEP_0194402934
Proboscia_alata_PI-D3 CAMPEP_0194366044
Proboscia_inermis_CCAP1064/1 CAMPEP_0171301908
Eucampia_antarctica_CCMP1452 CAMPEP_0197824402
Corethron_pennatum_L29A3 CAMPEP_0194305016
Chaetoceros_dichaeta_CCMP1751 CAMPEP_0198252364
Lysinibacillus_sp._B2A1 AVK86624
Streptomyces_orinoci WP_109280457
Licmophora_paradoxa_CCMP2313 CAMPEP_0202450682
Stauroneis_constricta_CCMP1120 CAMPEP_0119561652
Craspedostauros_australis_CCMP3328 CAMPEP_0198123664
Symbiodinium_sp._D1a CAMPEP_0196992952
Tiarina_fusus_LIS CAMPEP_0117032480
Tiarina_fusus_LIS CAMPEP_0117006460
Tiarina_fusus_LIS CAMPEP_0116997542
NonPhotosyntheticDiatom QUERY_NAA04P01710.m1
AhpC/TSA family protein
0.2

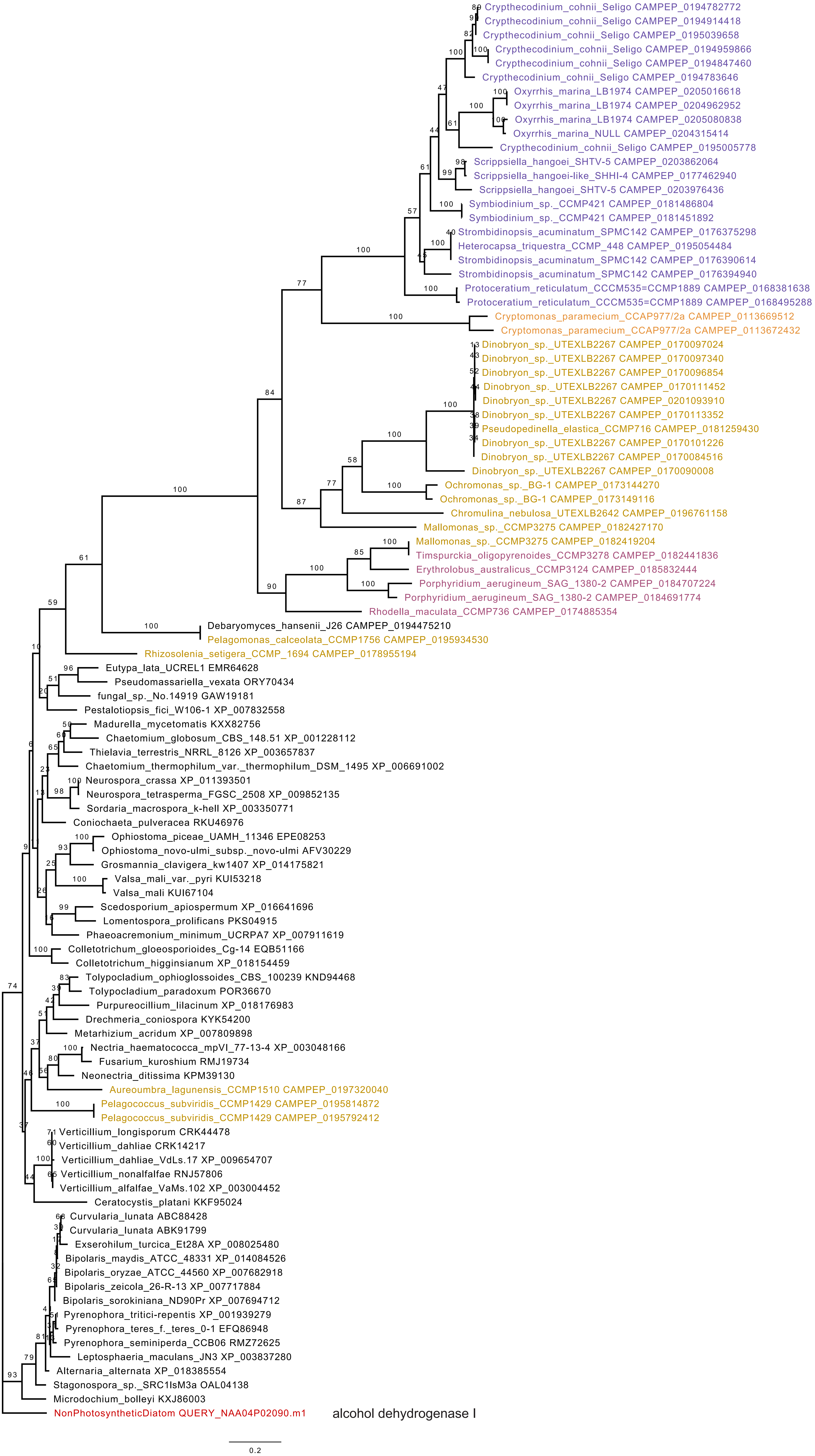

Crypthecodinium_cohnii_Seligo CAMPEP_0194782772
Crypthecodinium_cohnii_Seligo CAMPEP_0194914418
Crypthecodinium_cohnii_Seligo CAMPEP_0195039658
Crypthecodinium_cohnii_Seligo CAMPEP_0194959866
Crypthecodinium_cohnii_Seligo CAMPEP_0194847460
Crypthecodinium_cohnii_Seligo CAMPEP_0194783646
Oxyrrhis_marina_LB1974 CAMPEP_0205016618
Oxyrrhis_marina_LB1974 CAMPEP_0204962952
Oxyrrhis_marina_LB1974 CAMPEP_0205080838
Oxyrrhis_marina_NULL CAMPEP_0204315414
Crypthecodinium_cohnii_Seligo CAMPEP_0195005778
Scrippsiella_hangoei_SHTV-5 CAMPEP_0203862064
Scrippsiella_hangoei-like_SHHI-4 CAMPEP_0177462940
Scrippsiella_hangoei_SHTV-5 CAMPEP_0203976436
Symbiodinium_sp._CCMP421 CAMPEP_0181486804
Symbiodinium_sp._CCMP421 CAMPEP_0181451892
Strombidinopsis_acuminatum_SPMC142 CAMPEP_0176375298
Heterocapsa_triquestra_CCMP_448 CAMPEP_0195054484
Strombidinopsis_acuminatum_SPMC142 CAMPEP_0176390614
Strombidinopsis_acuminatum_SPMC142 CAMPEP_0176394940
Protoceratium_reticulatum_CCCM535=CCMP1889 CAMPEP_0168381638
Protoceratium_reticulatum_CCCM535=CCMP1889 CAMPEP_0168495288
Cryptomonas_paramecium_CCAP977/2a CAMPEP_0113669512
Cryptomonas_paramecium_CCAP977/2a CAMPEP_0113672432
Dinobryon_sp._UTEXLB2267 CAMPEP_0170097024
Dinobryon_sp._UTEXLB2267 CAMPEP_0170097340
Dinobryon_sp._UTEXLB2267 CAMPEP_0170096854
Dinobryon_sp._UTEXLB2267 CAMPEP_0170111452
Dinobryon_sp._UTEXLB2267 CAMPEP_0201093910
Dinobryon_sp._UTEXLB2267 CAMPEP_0170113352
Pseudopedinella_elastica_CCMP716 CAMPEP_0181259430
Dinobryon_sp._UTEXLB2267 CAMPEP_0170101226
Dinobryon_sp._UTEXLB2267 CAMPEP_0170084516
Dinobryon_sp._UTEXLB2267 CAMPEP_0170090008
Ochromonas_sp._BG-1 CAMPEP_0173144270
Ochromonas_sp._BG-1 CAMPEP_0173149116
Chromulina_nebulosa_UTEXLB2642 CAMPEP_0196761158
Mallomonas_sp._CCMP3275 CAMPEP_0182427170
Mallomonas_sp._CCMP3275 CAMPEP_0182419204
Timspurckia_oligopyrenoides_CCMP3278 CAMPEP_0182441836
Erythrolobus_australicus_CCMP3124 CAMPEP_0185832444
Porphyridium_aerugineum_SAG_1380-2 CAMPEP_0184707224
Porphyridium_aerugineum_SAG_1380-2 CAMPEP_0184691774
Rhodella_maculata_CCMP736 CAMPEP_0174885354
Debaryomyces_hansenii_J26 CAMPEP_0194475210
Pelagomonas_calceolata_CCMP1756 CAMPEP_0195934530
Rhizosolenia_setigera_CCMP_1694 CAMPEP_0178955194
Eutypa_lata_UCREL1 EMR64628
Pseudomassariella_vexata ORY70434
fungal_sp._No.14919 GAW19181
Pestalotiopsis_fici_W106-1 XP_007832558
Madurella_mycetomatis KXX82756
Chaetomium_globosum_CBS_148.51 XP_001228112
Thielavia_terrestris_NRRL_8126 XP_003657837
Chaetomium_thermophilum_var._thermophilum_DSM_1495 XP_006691002
Neurospora_crassa XP_011393501
Neurospora_tetrasperma_FGSC_2508 XP_009852135
Sordaria_macrospora_k-hell XP_003350771
Coniochaeta_pulveracea RKU46976
Ophiostoma_piceae_UAMH_11346 EPE08253
Ophiostoma_novo-ulmi_subsp._novo-ulmi AFV30229
Grosmannia_clavigera_kw1407 XP_014175821
Valsa_mali_var._pyri KUI53218
Valsa_mali KUI67104
Scedosporium_apiospermum XP_016641696
Lomentospora_prolificans PKS04915
Phaeoacremonium_minimum_UCRPA7 XP_007911619
Colletotrichum_gloeosporioides_Cg-14 EQB51166
Colletotrichum_higginsianum XP_018154459
Tolypocladium_ophioglossoides_CBS_100239 KND94468
Tolypocladium_paradoxum POR36670
Purpureocillium_lilacinum XP_018176983
Drechmeria_coniospora KYK54200
Metarhizium_acridum XP_007809898
Nectria_haematococca_mpVI_77-13-4 XP_003048166
Fusarium_kuroshium RMJ19734
Neonectria_ditissima KPM39130
Aureoumbra_lagunensis_CCMP1510 CAMPEP_0197320040
Pelagococcus_subviridis_CCMP1429 CAMPEP_0195814872
Pelagococcus_subviridis_CCMP1429 CAMPEP_0195792412
Verticillium_longisporum CRK44478
Verticillium_dahliae CRK14217
Verticillium_dahliae_VdLs.17 XP_009654707
Verticillium_nonalfalfae RNJ57806
Verticillium_alfalfae_VaMs.102 XP_003004452
Ceratocystis_platani KKF95024
Curvularia_lunata ABC88428
Curvularia_lunata ABK91799
Exserohilum_turcica_Et28A XP_008025480
Bipolaris_maydis_ATCC_48331 XP_014084526
Bipolaris_oryzae_ATCC_44560 XP_007682918
Bipolaris_zeicola_26-R-13 XP_007717884
Bipolaris_sorokiniana_ND90Pr XP_007694712
Pyrenophora_tritici-repentis XP_001939279
Pyrenophora_teres_f._teres_0-1 EFQ86948
Pyrenophora_seminiperda_CCB06 RMZ72625
Leptosphaeria_maculans_JN3 XP_003837280
Alternaria_alternata XP_018385554
Stagonospora_sp._SRC1lsM3a OAL04138
Microdochium_bolleyi KXJ86003
NonPhotosyntheticDiatom QUERY_NAA04P02090.m1
alcohol dehydrogenase I
0.2

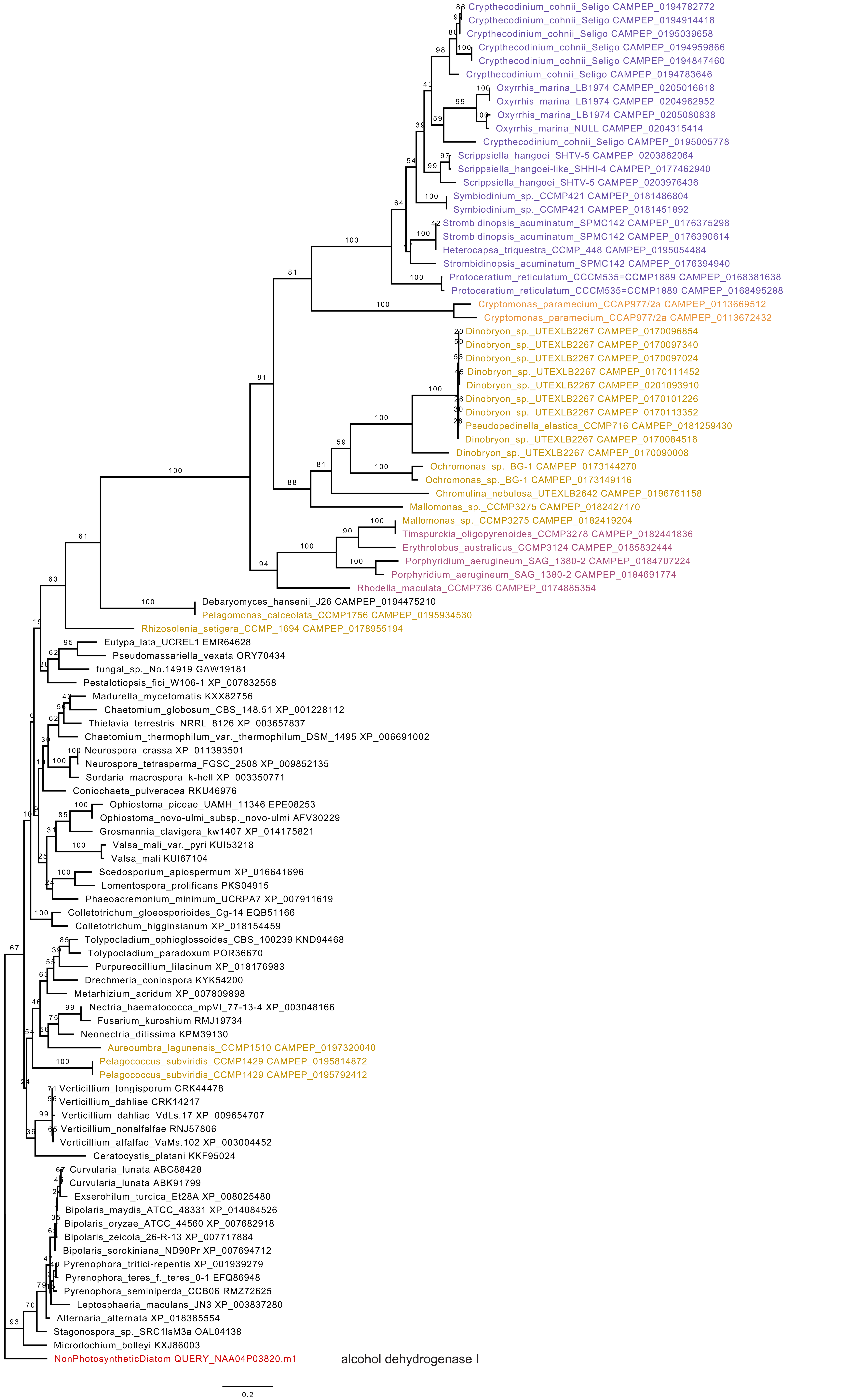

Crypthecodinium_cohnii_Seligo CAMPEP_0194782772
Crypthecodinium_cohnii_Seligo CAMPEP_0194914418
Crypthecodinium_cohnii_Seligo CAMPEP_0195039658
Crypthecodinium_cohnii_Seligo CAMPEP_0194959866
Crypthecodinium_cohnii_Seligo CAMPEP_0194847460
Crypthecodinium_cohnii_Seligo CAMPEP_0194783646
Oxyrrhis_marina_LB1974 CAMPEP_0205016618
Oxyrrhis_marina_LB1974 CAMPEP_0204962952
Oxyrrhis_marina_LB1974 CAMPEP_0205080838
Oxyrrhis_marina_NULL CAMPEP_0204315414
Crypthecodinium_cohnii_Seligo CAMPEP_0195005778
Scrippsiella_hangoei_SHTV-5 CAMPEP_0203862064
Scrippsiella_hangoei-like_SHHI-4 CAMPEP_0177462940
Scrippsiella_hangoei_SHTV-5 CAMPEP_0203976436
Symbiodinium_sp._CCMP421 CAMPEP_0181486804
Symbiodinium_sp._CCMP421 CAMPEP_0181451892
Strombidinopsis_acuminatum_SPMC142 CAMPEP_0176375298
Strombidinopsis_acuminatum_SPMC142 CAMPEP_0176390614
Heterocapsa_triquestra_CCMP_448 CAMPEP_0195054484
Strombidinopsis_acuminatum_SPMC142 CAMPEP_0176394940
Protoceratium_reticulatum_CCCM535=CCMP1889 CAMPEP_0168381638
Protoceratium_reticulatum_CCCM535=CCMP1889 CAMPEP_0168495288
Cryptomonas_paramecium_CCAP977/2a CAMPEP_0113669512
Cryptomonas_paramecium_CCAP977/2a CAMPEP_0113672432
Dinobryon_sp._UTEXLB2267 CAMPEP_0170096854
Dinobryon_sp._UTEXLB2267 CAMPEP_0170097340
Dinobryon_sp._UTEXLB2267 CAMPEP_0170097024
Dinobryon_sp._UTEXLB2267 CAMPEP_0170111452
Dinobryon_sp._UTEXLB2267 CAMPEP_0201093910
Dinobryon_sp._UTEXLB2267 CAMPEP_0170101226
Dinobryon_sp._UTEXLB2267 CAMPEP_0170113352
Pseudopedinella_elastica_CCMP716 CAMPEP_0181259430
Dinobryon_sp._UTEXLB2267 CAMPEP_0170084516
Dinobryon_sp._UTEXLB2267 CAMPEP_0170090008
Ochromonas_sp._BG-1 CAMPEP_0173144270
Ochromonas_sp._BG-1 CAMPEP_0173149116
Chromulina_nebulosa_UTEXLB2642 CAMPEP_0196761158
Mallomonas_sp._CCMP3275 CAMPEP_0182427170
Mallomonas_sp._CCMP3275 CAMPEP_0182419204
Timspurckia_oligopyrenoides_CCMP3278 CAMPEP_0182441836
Erythrolobus_australicus_CCMP3124 CAMPEP_0185832444
Porphyridium_aerugineum_SAG_1380-2 CAMPEP_0184707224
Porphyridium_aerugineum_SAG_1380-2 CAMPEP_0184691774
Rhodella_maculata_CCMP736 CAMPEP_0174885354
Debaryomyces_hansenii_J26 CAMPEP_0194475210
Pelagomonas_calceolata_CCMP1756 CAMPEP_0195934530
Rhizosolenia_setigera_CCMP_1694 CAMPEP_0178955194
Eutypa_lata_UCREL1 EMR64628
Pseudomassariella_vexata ORY70434
fungal_sp._No.14919 GAW19181
Pestalotiopsis_fici_W106-1 XP_007832558
Madurella_mycetomatis KXX82756
Chaetomium_globosum_CBS_148.51 XP_001228112
Thielavia_terrestris_NRRL_8126 XP_003657837
Chaetomium_thermophilum_var._thermophilum_DSM_1495 XP_006691002
Neurospora_crassa XP_011393501
Neurospora_tetrasperma_FGSC_2508 XP_009852135
Sordaria_macrospora_k-hell XP_003350771
Coniochaeta_pulveracea RKU46976
Ophiostoma_piceae_UAMH_11346 EPE08253
Ophiostoma_novo-ulmi_subsp._novo-ulmi AFV30229
Grosmannia_clavigera_kw1407 XP_014175821
Valsa_mali_var._pyri KUI53218
Valsa_mali KUI67104
Scedosporium_apiospermum XP_016641696
Lomentospora_prolificans PKS04915
Phaeoacremonium_minimum_UCRPA7 XP_007911619
Colletotrichum_gloeosporioides_Cg-14 EQB51166
Colletotrichum_higginsianum XP_018154459
Tolypocladium_ophioglossoides_CBS_100239 KND94468
Tolypocladium_paradoxum POR36670
Purpureocillium_lilacinum XP_018176983
Drechmeria_coniospora KYK54200
Metarhizium_acridum XP_007809898
Nectria_haematococca_mpVI_77-13-4 XP_003048166
Fusarium_kuroshium RMJ19734
Neonectria_ditissima KPM39130
Aureoumbra_lagunensis_CCMP1510 CAMPEP_0197320040
Pelagococcus_subviridis_CCMP1429 CAMPEP_0195814872
Pelagococcus_subviridis_CCMP1429 CAMPEP_0195792412
Verticillium_longisporum CRK44478
Verticillium_dahliae CRK14217
Verticillium_dahliae_VdLs.17 XP_009654707
Verticillium_nonalfalfae RNJ57806
Verticillium_alfalfae_VaMs.102 XP_003004452
Ceratocystis_platani KKF95024
Curvularia_lunata ABC88428
Curvularia_lunata ABK91799
Exserohilum_turcica_Et28A XP_008025480
Bipolaris_maydis_ATCC_48331 XP_014084526
Bipolaris_oryzae_ATCC_44560 XP_007682918
Bipolaris_zeicola_26-R-13 XP_007717884
Bipolaris_sorokiniana_ND90Pr XP_007694712
Pyrenophora_tritici-repentis XP_001939279
Pyrenophora_teres_f._teres_0-1 EFQ86948
Pyrenophora_seminiperda_CCB06 RMZ72625
Leptosphaeria_maculans_JN3 XP_003837280
Alternaria_alternata XP_018385554
Stagonospora_sp._SRC1lsM3a OAL04138
Microdochium_bolleyi KXJ86003
NonPhotosyntheticDiatom QUERY_NAA04P03820.m1
alcohol dehydrogenase I
0.2

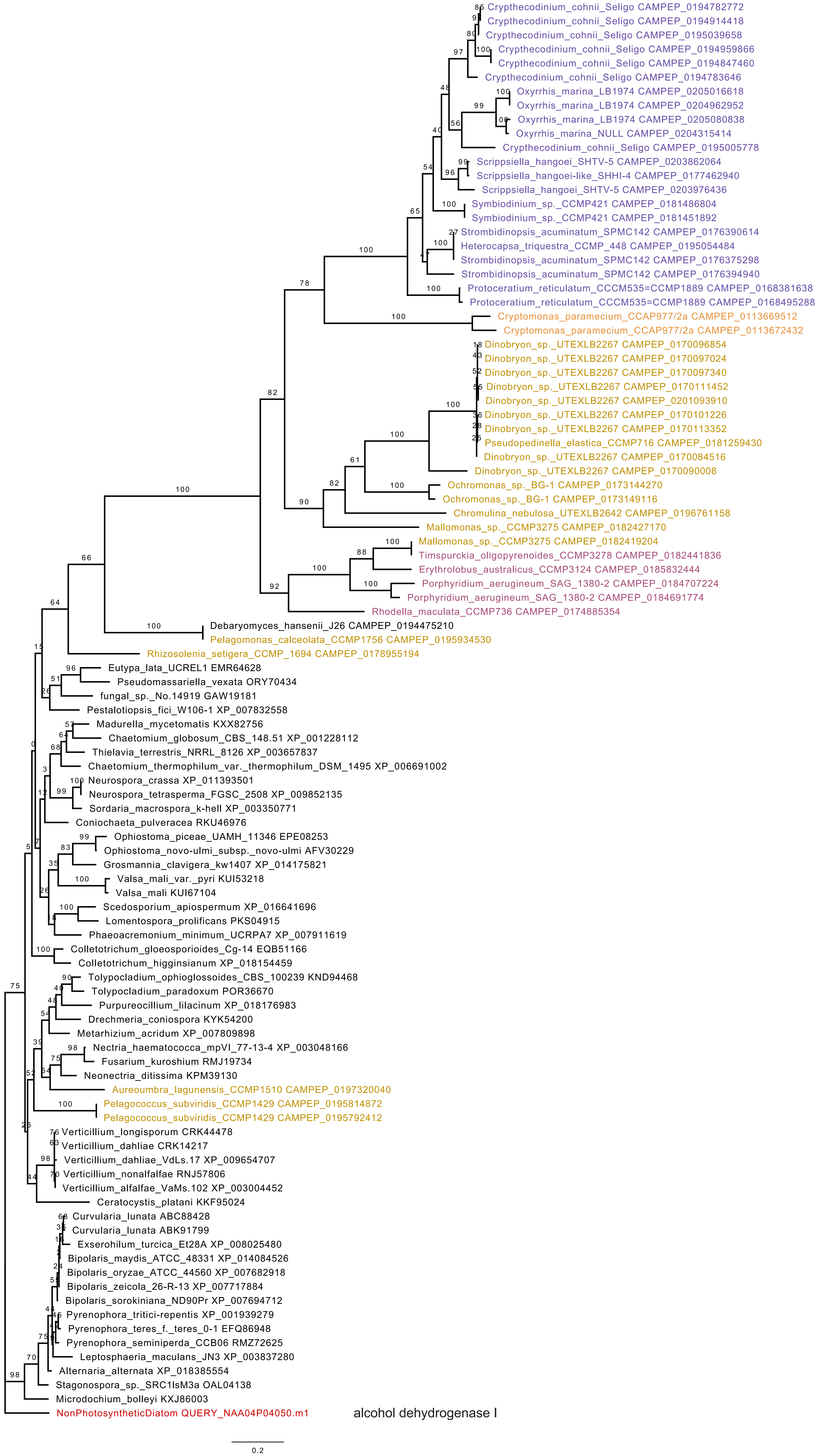
Crypthecodinium_cohnii_Seligo CAMPEP_0194782772
Crypthecodinium_cohnii_Seligo CAMPEP_0194914418
Crypthecodinium_cohnii_Seligo CAMPEP_0195039658
Crypthecodinium_cohnii_Seligo CAMPEP_0194959866
Crypthecodinium_cohnii_Seligo CAMPEP_0194847460
Crypthecodinium_cohnii_Seligo CAMPEP_0194783646
Oxyrrhis_marina_LB1974 CAMPEP_0205016618
Oxyrrhis_marina_LB1974 CAMPEP_0204962952
Oxyrrhis_marina_LB1974 CAMPEP_0205080838
Oxyrrhis_marina_NULL CAMPEP_0204315414
Crypthecodinium_cohnii_Seligo CAMPEP_0195005778
Scrippsiella_hangoei_SHTV-5 CAMPEP_0203862064
Scrippsiella_hangoei-like_SHHI-4 CAMPEP_0177462940
Scrippsiella_hangoei_SHTV-5 CAMPEP_0203976436
Symbiodinium_sp._CCMP421 CAMPEP_0181486804
Symbiodinium_sp._CCMP421 CAMPEP_0181451892
Strombidinopsis_acuminatum_SPMC142 CAMPEP_0176390614
Heterocapsa_triquestra_CCMP_448 CAMPEP_0195054484
Strombidinopsis_acuminatum_SPMC142 CAMPEP_0176375298
Strombidinopsis_acuminatum_SPMC142 CAMPEP_0176394940
Protoceratium_reticulatum_CCCM535=CCMP1889 CAMPEP_0168381638
Protoceratium_reticulatum_CCCM535=CCMP1889 CAMPEP_0168495288
Cryptomonas_paramecium_CCAP977/2a CAMPEP_0113669512
Cryptomonas_paramecium_CCAP977/2a CAMPEP_0113672432
Dinobryon_sp._UTEXLB2267 CAMPEP_0170096854
Dinobryon_sp._UTEXLB2267 CAMPEP_0170097024
Dinobryon_sp._UTEXLB2267 CAMPEP_0170097340
Dinobryon_sp._UTEXLB2267 CAMPEP_0170111452
Dinobryon_sp._UTEXLB2267 CAMPEP_0201093910
Dinobryon_sp._UTEXLB2267 CAMPEP_0170101226
Dinobryon_sp._UTEXLB2267 CAMPEP_0170113352
Pseudopedinella_elastica_CCMP716 CAMPEP_0181259430
Dinobryon_sp._UTEXLB2267 CAMPEP_0170084516
Dinobryon_sp._UTEXLB2267 CAMPEP_0170090008
Ochromonas_sp._BG-1 CAMPEP_0173144270
Ochromonas_sp._BG-1 CAMPEP_0173149116
Chromulina_nebulosa_UTEXLB2642 CAMPEP_0196761158
Mallomonas_sp._CCMP3275 CAMPEP_0182427170
Mallomonas_sp._CCMP3275 CAMPEP_0182419204
Timspurckia_oligopyrenoides_CCMP3278 CAMPEP_0182441836
Erythrolobus_australicus_CCMP3124 CAMPEP_0185832444
Porphyridium_aerugineum_SAG_1380-2 CAMPEP_0184707224
Porphyridium_aerugineum_SAG_1380-2 CAMPEP_0184691774
Rhodella_maculata_CCMP736 CAMPEP_0174885354
Debaryomyces_hansenii_J26 CAMPEP_0194475210
Pelagomonas_calceolata_CCMP1756 CAMPEP_0195934530
Rhizosolenia_setigera_CCMP_1694 CAMPEP_0178955194
Eutypa_lata_UCREL1 EMR64628
Pseudomassariella_vexata ORY70434
fungal_sp._No.14919 GAW19181
Pestalotiopsis_fici_W106-1 XP_007832558
Madurella_mycetomatis KXX82756
Chaetomium_globosum_CBS_148.51 XP_001228112
Thielavia_terrestris_NRRL_8126 XP_003657837
Chaetomium_thermophilum_var._thermophilum_DSM_1495 XP_006691002
Neurospora_crassa XP_011393501
Neurospora_tetrasperma_FGSC_2508 XP_009852135
Sordaria_macrospora_k-hell XP_003350771
Coniochaeta_pulveracea RKU46976
Ophiostoma_piceae_UAMH_11346 EPE08253
Ophiostoma_novo-ulmi_subsp._novo-ulmi AFV30229
Grosmannia_clavigera_kw1407 XP_014175821
Valsa_mali_var._pyri KUI53218
Valsa_mali KUI67104
Scedosporium_apiospermum XP_016641696
Lomentospora_prolificans PKS04915
Phaeoacremonium_minimum_UCRPA7 XP_007911619
Colletotrichum_gloeosporioides_Cg-14 EQB51166
Colletotrichum_higginsianum XP_018154459
Tolypocladium_ophioglossoides_CBS_100239 KND94468
Tolypocladium_paradoxum POR36670
Purpureocillium_lilacinum XP_018176983
Drechmeria_coniospora KYK54200
Metarhizium_acridum XP_007809898
Nectria_haematococca_mpVI_77-13-4 XP_003048166
Fusarium_kuroshium RMJ19734
Neonectria_ditissima KPM39130
Aureoumbra_lagunensis_CCMP1510 CAMPEP_0197320040
Pelagococcus_subviridis_CCMP1429 CAMPEP_0195814872
Pelagococcus_subviridis_CCMP1429 CAMPEP_0195792412
Verticillium_longisporum CRK44478
Verticillium_dahliae CRK14217
Verticillium_dahliae_VdLs.17 XP_009654707
Verticillium_nonalfalfae RNJ57806
Verticillium_alfalfae_VaMs.102 XP_003004452
Ceratocystis_platani KKF95024
Curvularia_lunata ABC88428
Curvularia_lunata ABK91799
Exserohilum_turcica_Et28A XP_008025480
Bipolaris_maydis_ATCC_48331 XP_014084526
Bipolaris_oryzae_ATCC_44560 XP_007682918
Bipolaris_zeicola_26-R-13 XP_007717884
Bipolaris_sorokiniana_ND90Pr XP_007694712
Pyrenophora_tritici-repentis XP_001939279
Pyrenophora_teres_f._teres_0-1 EFQ86948
Pyrenophora_seminiperda_CCB06 RMZ72625
Leptosphaeria_maculans_JN3 XP_003837280
Alternaria_alternata XP_018385554
Stagonospora_sp._SRC1lsM3a OAL04138
Microdochium_bolleyi KXJ86003
NonPhotosyntheticDiatom QUERY_NAA04P04050.m1
alcohol dehydrogenase I
0.2

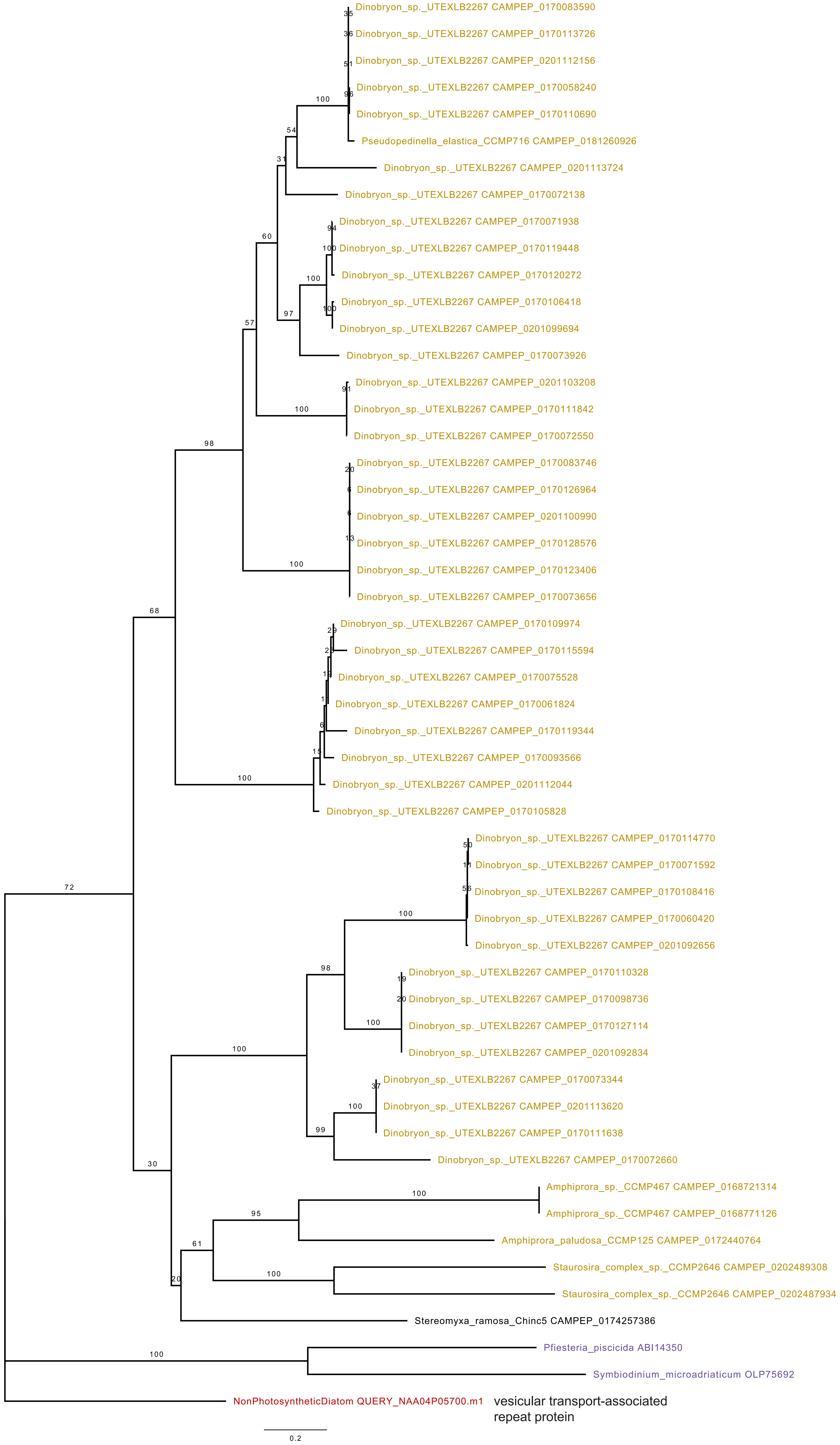
Dinobryon_sp._UTEXLB2267 CAMPEP_0170083590
Dinobryon_sp._UTEXLB2267 CAMPEP_0170113726
Dinobryon_sp._UTEXLB2267 CAMPEP_0201112156
Dinobryon_sp._UTEXLB2267 CAMPEP_0170058240
Dinobryon_sp._UTEXLB2267 CAMPEP_0170110690
Pseudopedinella_elastica_CCMP716 CAMPEP_0181260926
Dinobryon_sp._UTEXLB2267 CAMPEP_0201113724
Dinobryon_sp._UTEXLB2267 CAMPEP_0170072138
Dinobryon_sp._UTEXLB2267 CAMPEP_0170071938
Dinobryon_sp._UTEXLB2267 CAMPEP_0170119448
Dinobryon_sp._UTEXLB2267 CAMPEP_0170120272
Dinobryon_sp._UTEXLB2267 CAMPEP_0170106418
Dinobryon_sp._UTEXLB2267 CAMPEP_0201099694
Dinobryon_sp._UTEXLB2267 CAMPEP_0170073926
Dinobryon_sp._UTEXLB2267 CAMPEP_0201103208
Dinobryon_sp._UTEXLB2267 CAMPEP_0170111842
Dinobryon_sp._UTEXLB2267 CAMPEP_0170072550
Dinobryon_sp._UTEXLB2267 CAMPEP_0170083746
Dinobryon_sp._UTEXLB2267 CAMPEP_0170126964
Dinobryon_sp._UTEXLB2267 CAMPEP_0201100990
Dinobryon_sp._UTEXLB2267 CAMPEP_0170128576
Dinobryon_sp._UTEXLB2267 CAMPEP_0170123406
Dinobryon_sp._UTEXLB2267 CAMPEP_0170073656
Dinobryon_sp._UTEXLB2267 CAMPEP_0170109974
Dinobryon_sp._UTEXLB2267 CAMPEP_0170115594
Dinobryon_sp._UTEXLB2267 CAMPEP_0170075528
Dinobryon_sp._UTEXLB2267 CAMPEP_0170061824
Dinobryon_sp._UTEXLB2267 CAMPEP_0170119344
Dinobryon_sp._UTEXLB2267 CAMPEP_0170093566
Dinobryon_sp._UTEXLB2267 CAMPEP_0201112044
Dinobryon_sp._UTEXLB2267 CAMPEP_0170105828
Dinobryon_sp._UTEXLB2267 CAMPEP_0170114770
Dinobryon_sp._UTEXLB2267 CAMPEP_0170071592
Dinobryon_sp._UTEXLB2267 CAMPEP_0170108416
Dinobryon_sp._UTEXLB2267 CAMPEP_0170060420
Dinobryon_sp._UTEXLB2267 CAMPEP_0201092656
Dinobryon_sp._UTEXLB2267 CAMPEP_0170110328
Dinobryon_sp._UTEXLB2267 CAMPEP_0170098736
Dinobryon_sp._UTEXLB2267 CAMPEP_0170127114
Dinobryon_sp._UTEXLB2267 CAMPEP_0201092834
Dinobryon_sp._UTEXLB2267 CAMPEP_0170073344
Dinobryon_sp._UTEXLB2267 CAMPEP_0201113620
Dinobryon_sp._UTEXLB2267 CAMPEP_0170111638
Dinobryon_sp._UTEXLB2267 CAMPEP_0170072660
Amphiprora_sp._CCMP467 CAMPEP_0168721314
Amphiprora_sp._CCMP467 CAMPEP_0168771126
Amphiprora_paludosa_CCMP125 CAMPEP_0172440764
Staurosira_complex_sp._CCMP2646 CAMPEP_0202489308
Staurosira_complex_sp._CCMP2646 CAMPEP_0202487934
Stereomyxa_ramosa_Chinc5 CAMPEP_0174257386
Pfiesteria_piscicida ABI14350
Symbiodinium_microadriaticum OLP75692
NonPhotosyntheticDiatom QUERY_NAA04P05700.m1
vesicular transport-associated repeat protein
0.2

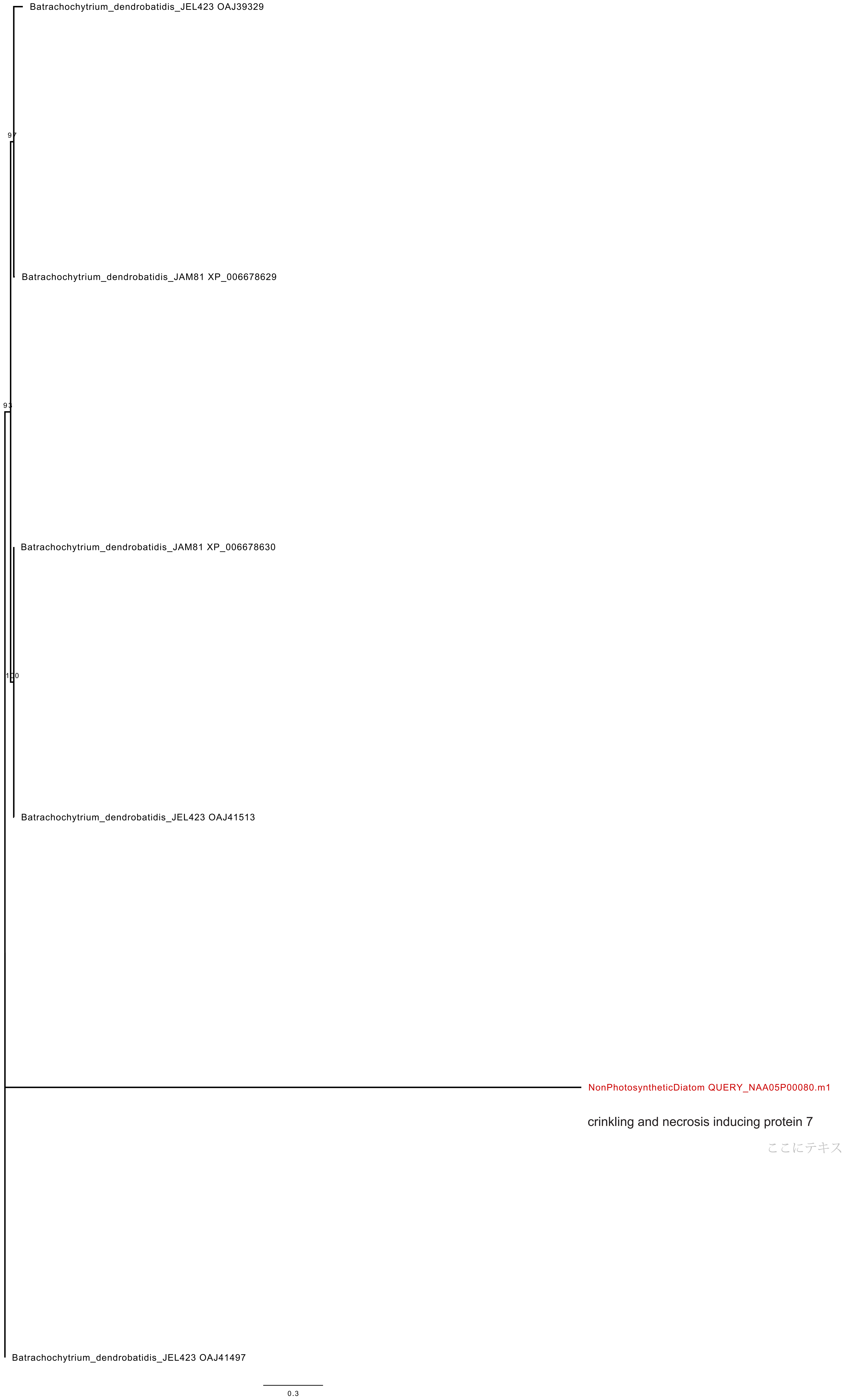
Batrachochytrium_dendrobatidis_JEL423 OAJ39329
97
Batrachochytrium_dendrobatidis_JAM81 XP_006678629
93
Batrachochytrium_dendrobatidis_JAM81 XP_006678630
100
Batrachochytrium_dendrobatidis_JEL423 OAJ41513
NonPhotosyntheticDiatom QUERY_NAA05P00080.m1
crinkling and necrosis inducing protein 7
ここにテキス
Batrachochytrium_dendrobatidis_JEL423 OAJ41497
0.3

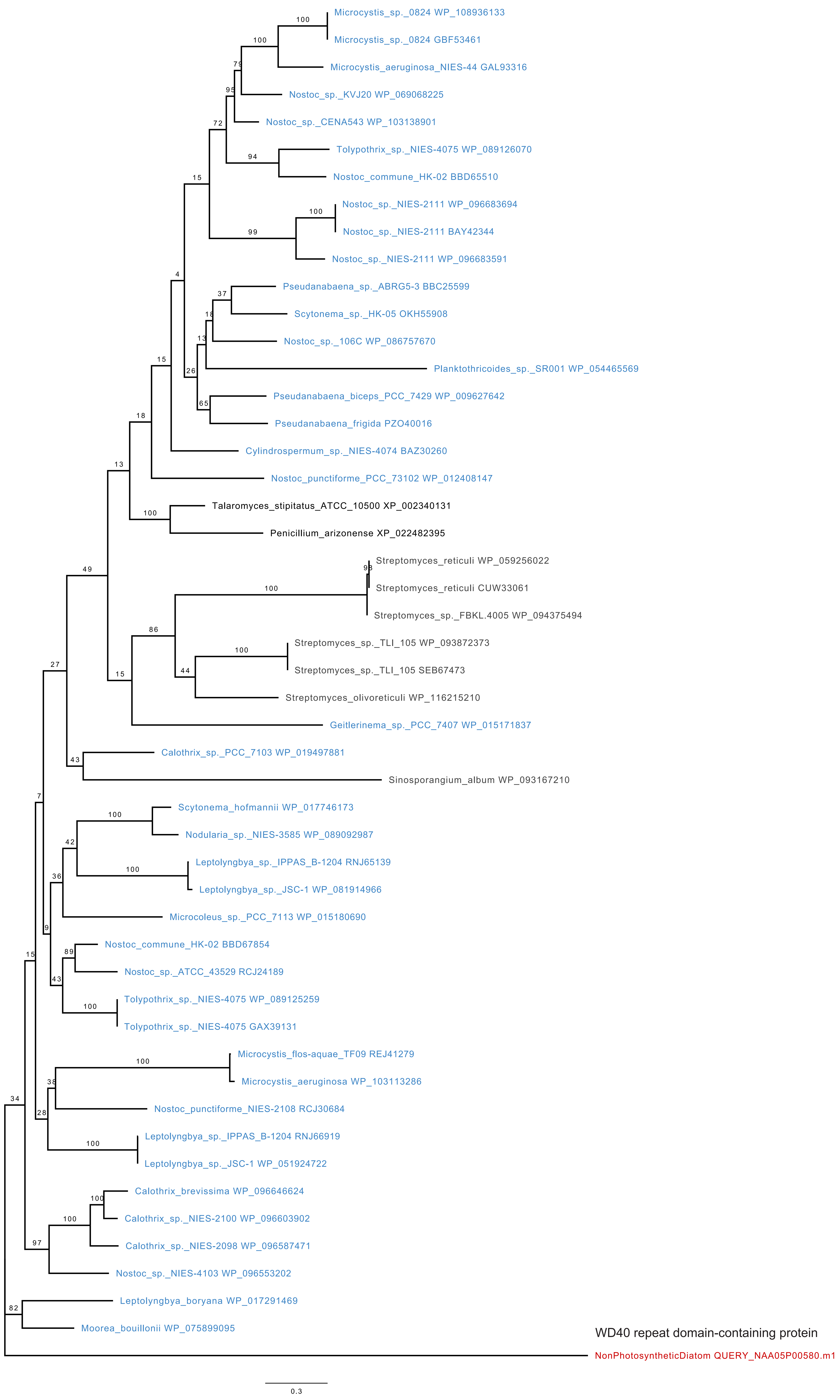

Microcystis_sp._0824 WP_108936133
Microcystis_sp._0824 GBF53461
Microcystis_aeruginosa_NIES-44 GAL93316
Nostoc_sp._KVJ20 WP_069068225
Nostoc_sp._CENA543 WP_103138901
Tolypothrix_sp._NIES-4075 WP_089126070
Nostoc_commune_HK-02 BBD65510
Nostoc_sp._NIES-2111 WP_096683694
Nostoc_sp._NIES-2111 BAY42344
Nostoc_sp._NIES-2111 WP_096683591
Pseudanabaena_sp._ABRG5-3 BBC25599
Scytonema_sp._HK-05 OKH55908
Nostoc_sp._106C WP_086757670
Planktothricoides_sp._SR001 WP_054465569
Pseudanabaena_biceps_PCC_7429 WP_009627642
Pseudanabaena_frigida PZO40016
Cylindrospermum_sp._NIES-4074 BAZ30260
Nostoc_punctiforme_PCC_73102 WP_012408147
Talaromyces_stipitatus_ATCC_10500 XP_002340131
Penicillium_arizonense XP_022482395
Streptomyces_reticuli WP_059256022
Streptomyces_reticuli CUW33061
Streptomyces_sp._FBKL.4005 WP_094375494
Streptomyces_sp._TLI_105 WP_093872373
Streptomyces_sp._TLI_105 SEB67473
Streptomyces_olivoreticuli WP_116215210
Geitlerinema_sp._PCC_7407 WP_015171837
Calothrix_sp._PCC_7103 WP_019497881
Sinosporangium_album WP_093167210
Scytonema_hofmannii WP_017746173
Nodularia_sp._NIES-3585 WP_089092987
Leptolyngbya_sp._IPPAS_B-1204 RNJ65139
Leptolyngbya_sp._JSC-1 WP_081914966
Microcoleus_sp._PCC_7113 WP_015180690
Nostoc_commune_HK-02 BBD67854
Nostoc_sp._ATCC_43529 RCJ24189
Tolypothrix_sp._NIES-4075 WP_089125259
Tolypothrix_sp._NIES-4075 GAX39131
Microcystis_flos-aquae_TF09 REJ41279
Microcystis_aeruginosa WP_103113286
Nostoc_punctiforme_NIES-2108 RCJ30684
Leptolyngbya_sp._IPPAS_B-1204 RNJ66919
Leptolyngbya_sp._JSC-1 WP_051924722
Calothrix_brevissima WP_096646624
Calothrix_sp._NIES-2100 WP_096603902
Calothrix_sp._NIES-2098 WP_096587471
Nostoc_sp._NIES-4103 WP_096553202
Leptolyngbya_boryana WP_017291469
Moorea_bouillonii WP_075899095
WD40 repeat domain-containing protein
NonPhotosyntheticDiatom QUERY_NAA05P00580.m1
0.3

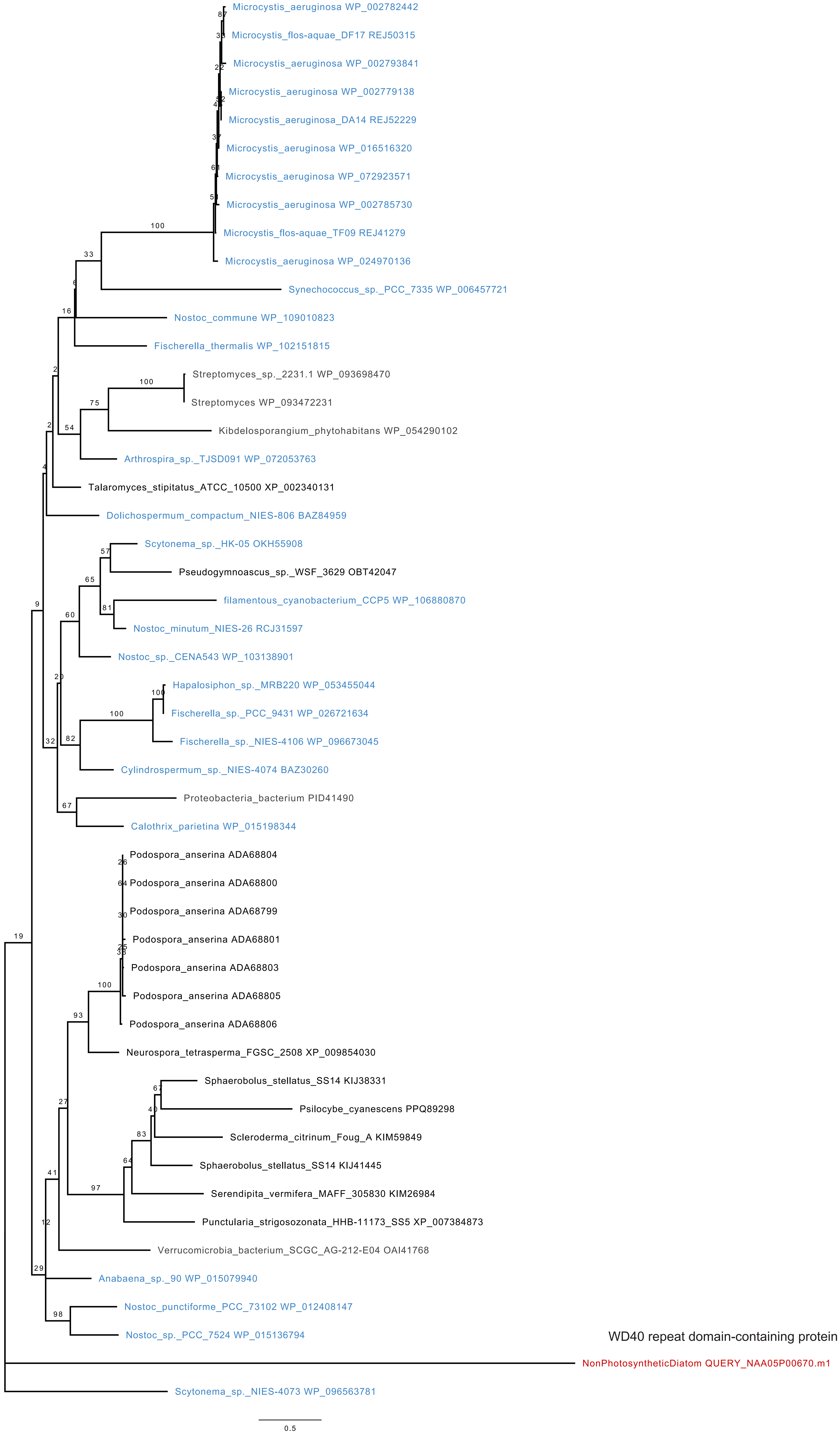
Microcystis_aeruginosa WP_002782442
Microcystis_flos-aquae_DF17 REJ50315
Microcystis_aeruginosa WP_002793841
Microcystis_aeruginosa WP_002779138
Microcystis_aeruginosa_DA14 REJ52229
Microcystis_aeruginosa WP_016516320
Microcystis_aeruginosa WP_072923571
Microcystis_aeruginosa WP_002785730
Microcystis_flos-aquae_TF09 REJ41279
Microcystis_aeruginosa WP_024970136
Synechococcus_sp._PCC_7335 WP_006457721
Nostoc_commune WP_109010823
Fischerella_thermalis WP_102151815
Streptomyces_sp._2231.1 WP_093698470
Streptomyces WP_093472231
Kibdelosporangium_phytohabitans WP_054290102
Arthrospira_sp._TJSD091 WP_072053763
Talaromyces_stipitatus_ATCC_10500 XP_002340131
Dolichospermum_compactum_NIES-806 BAZ84959
Scytonema_sp._HK-05 OKH55908
Pseudogymnoascus_sp._WSF_3629 OBT42047
filamentous_cyanobacterium_CCP5 WP_106880870
Nostoc_minutum_NIES-26 RCJ31597
Nostoc_sp._CENA543 WP_103138901
Hapalosiphon_sp._MRB220 WP_053455044
Fischerella_sp._PCC_9431 WP_026721634
Fischerella_sp._NIES-4106 WP_096673045
Cylindrospermum_sp._NIES-4074 BAZ30260
Proteobacteria_bacterium PID41490
Calothrix_parietina WP_015198344
Podospora_anserina ADA68804
Podospora_anserina ADA68800
Podospora_anserina ADA68799
Podospora_anserina ADA68801
Podospora_anserina ADA68803
Podospora_anserina ADA68805
Podospora_anserina ADA68806
Neurospora_tetrasperma_FGSC_2508 XP_009854030
Sphaerobolus_stellatus_SS14 KIJ38331
Psilocybe_cyanescens PPQ89298
Scleroderma_citrinum_Foug_A KIM59849
Sphaerobolus_stellatus_SS14 KIJ41445
Serendipita_vermifera_MAFF_305830 KIM26984
Punctularia_strigosozonata_HHB-11173_SS5 XP_007384873
Verrucomicrobia_bacterium_SCGC_AG-212-E04 OAI41768
Anabaena_sp._90 WP_015079940
Nostoc_punctiforme_PCC_73102 WP_012408147
Nostoc_sp._PCC_7524 WP_015136794
WD40 repeat domain-containing protein
NonPhotosyntheticDiatom QUERY_NAA05P00670.m1
Scytonema_sp._NIES-4073 WP_096563781
0.5

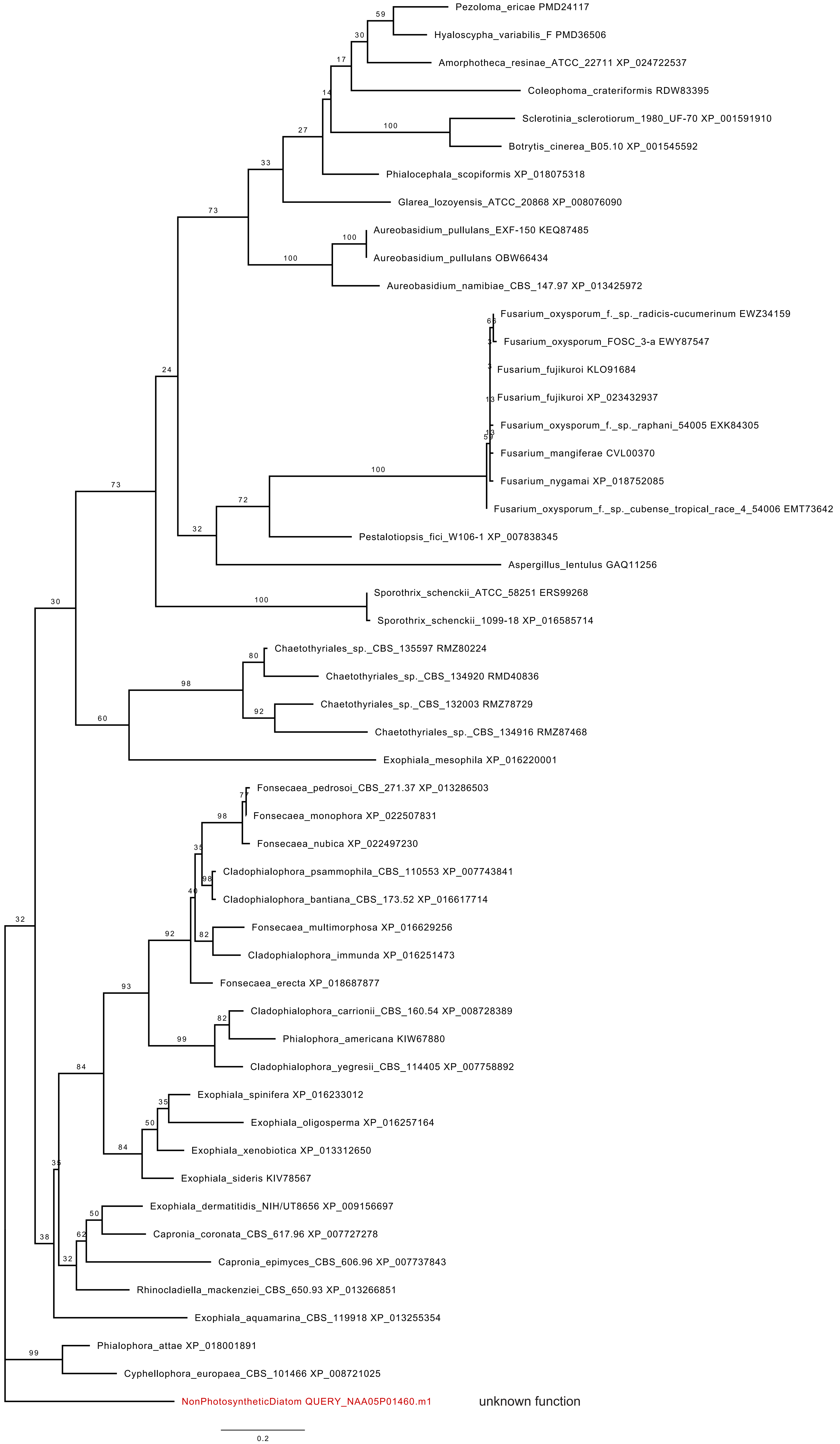

Pezoloma_ericae PMD24117
Hyaloscypha_variabilis_F PMD36506
Amorphotheca_resinae_ATCC_22711 XP_024722537
Coleophoma_crateriformis RDW83395
Sclerotinia_sclerotiorum_1980_UF-70 XP_001591910
Botrytis_cinerea_B05.10 XP_001545592
Phialocephala_scopiformis XP_018075318
Glarea_lozoyensis_ATCC_20868 XP_008076090
Aureobasidium_pullulans_EXF-150 KEQ87485
Aureobasidium_pullulans OBW66434
Aureobasidium_namibiae_CBS_147.97 XP_013425972
Fusarium_oxysporum_f._sp._radicis-cucumerinum EWZ34159
Fusarium_oxysporum_FOSC_3-a EWY87547
Fusarium_fujikuroi KLO91684
Fusarium_fujikuroi XP_023432937
Fusarium_oxysporum_f._sp._raphani_54005 EXK84305
Fusarium_mangiferae CVL00370
Fusarium_nygamai XP_018752085
Fusarium_oxysporum_f._sp._cubense_tropical_race_4_54006 EMT73642
Pestalotiopsis_fici_W106-1 XP_007838345
Aspergillus_lentulus GAQ11256
Sporothrix_schenckii_ATCC_58251 ERS99268
Sporothrix_schenckii_1099-18 XP_016585714
Chaetothyriales_sp._CBS_135597 RMZ80224
Chaetothyriales_sp._CBS_134920 RMD40836
Chaetothyriales_sp._CBS_132003 RMZ78729
Chaetothyriales_sp._CBS_134916 RMZ87468
Exophiala_mesophila XP_016220001
Fonsecaea_pedrosoi_CBS_271.37 XP_013286503
Fonsecaea_monophora XP_022507831
Fonsecaea_nubica XP_022497230
Cladophialophora_psammophila_CBS_110553 XP_007743841
Cladophialophora_bantiana_CBS_173.52 XP_016617714
Fonsecaea_multimorphosa XP_016629256
Cladophialophora_immunda XP_016251473
Fonsecaea_erecta XP_018687877
Cladophialophora_carrionii_CBS_160.54 XP_008728389
Phialophora_americana KIW67880
Cladophialophora_yegresii_CBS_114405 XP_007758892
Exophiala_spinifera XP_016233012
Exophiala_oligosperma XP_016257164
Exophiala_xenobiotica XP_013312650
Exophiala_sideris KIV78567
Exophiala_dermatitidis_NIH/UT8656 XP_009156697
Capronia_coronata_CBS_617.96 XP_007727278
Capronia_epimyces_CBS_606.96 XP_007737843
Rhinocladiella_mackenziei_CBS_650.93 XP_013266851
Exophiala_aquamarina_CBS_119918 XP_013255354
Phialophora_attae XP_018001891
Cyphellophora_europaea_CBS_101466 XP_008721025
NonPhotosyntheticDiatom QUERY_NAA05P01460.m1
unknown function
0.2

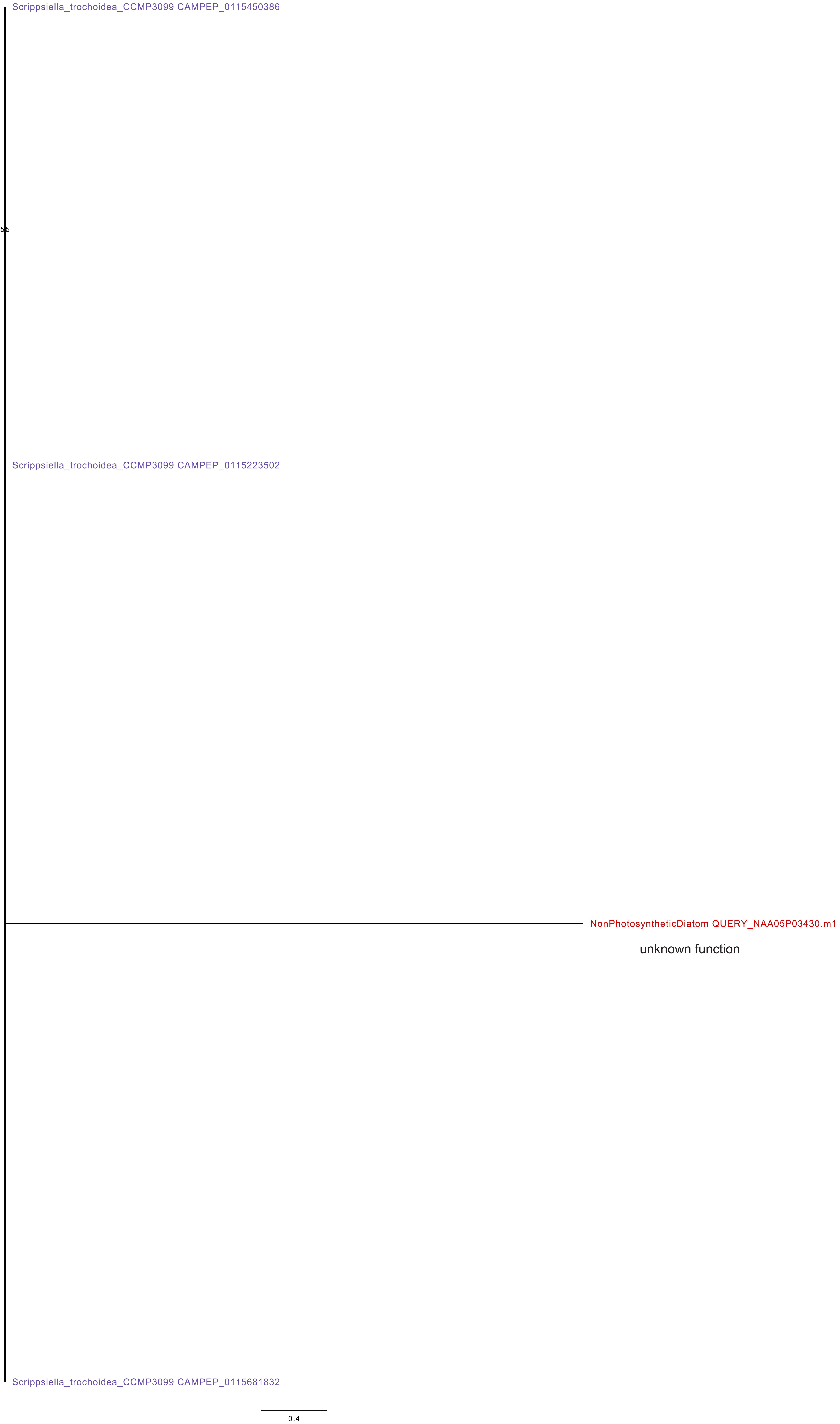
Scrippsiella_trochoidea_CCMP3099 CAMPEP_0115450386
55
Scrippsiella_trochoidea_CCMP3099 CAMPEP_0115223502
NonPhotosyntheticDiatom QUERY_NAA05P03430.m1
unknown function
Scrippsiella_trochoidea_CCMP3099 CAMPEP_0115681832
0.4

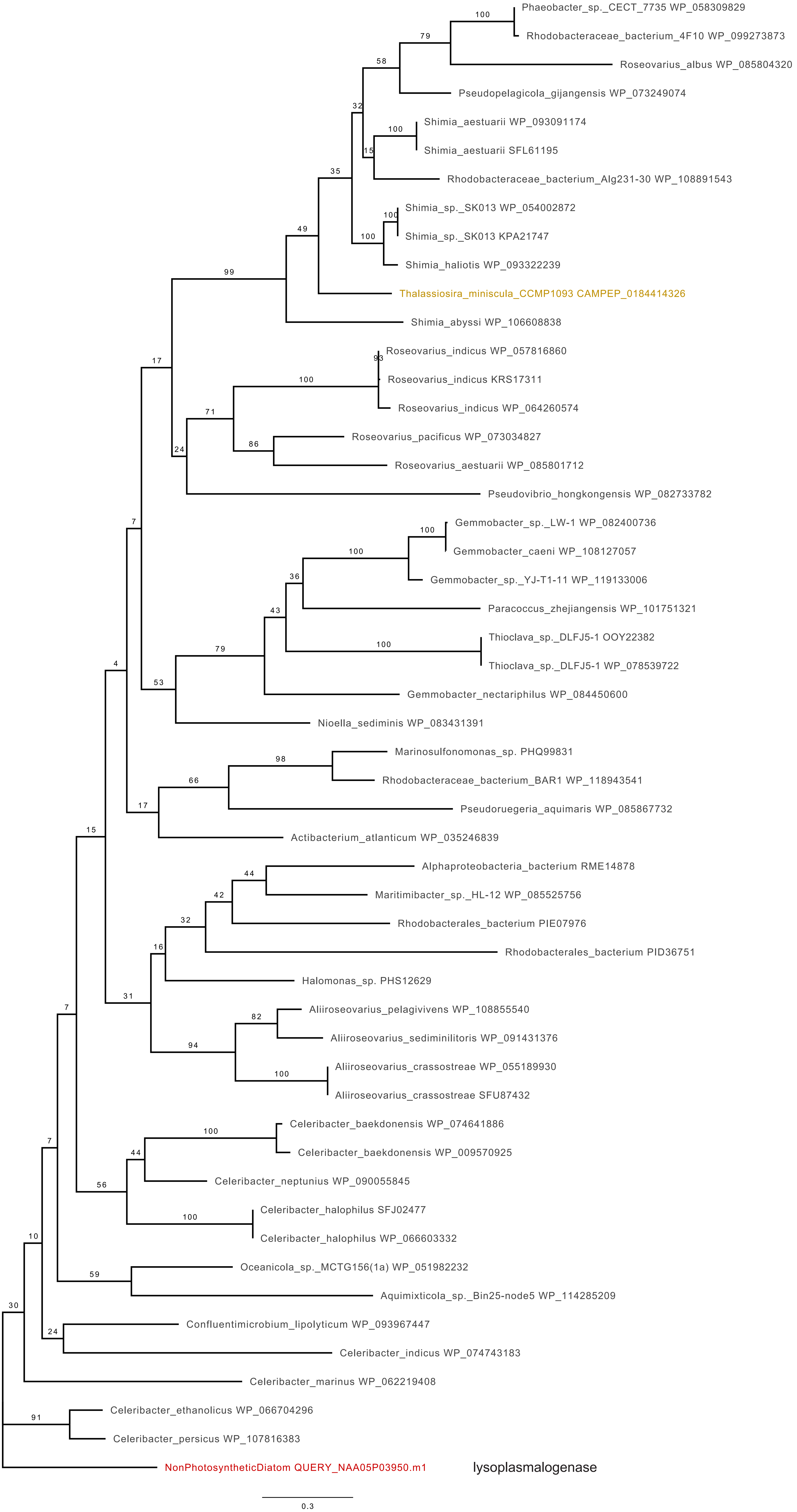
Phaeobacter_sp._CECT_7735 WP_058309829
Rhodobacteraceae_bacterium_4F10 WP_099273873
Roseovarius_albus WP_085804320
Pseudopelagicola_gijangensis WP_073249074
Shimia_aestuarii WP_093091174
Shimia_aestuarii SFL61195
Rhodobacteraceae_bacterium_Alg231-30 WP_108891543
Shimia_sp._SK013 WP_054002872
Shimia_sp._SK013 KPA21747
Shimia_haliotis WP_093322239
Thalassiosira_miniscula_CCMP1093 CAMPEP_0184414326
Shimia_abyssi WP_106608838
Roseovarius_indicus WP_057816860
Roseovarius_indicus KRS17311
Roseovarius_indicus WP_064260574
Roseovarius_pacificus WP_073034827
Roseovarius_aestuarii WP_085801712
Pseudovibrio_hongkongensis WP_082733782
Gemmobacter_sp._LW-1 WP_082400736
Gemmobacter_caeni WP_108127057
Gemmobacter_sp._YJ-T1-11 WP_119133006
Paracoccus_zhejiangensis WP_101751321
Thioclava_sp._DLFJ5-1 OOY22382
Thioclava_sp._DLFJ5-1 WP_078539722
Gemmobacter_nectariphilus WP_084450600
Nioella_sediminis WP_083431391
Marinosulfonomonas_sp. PHQ99831
Rhodobacteraceae_bacterium_BAR1 WP_118943541
Pseudoruegeria_aquimaris WP_085867732
Actibacterium_atlanticum WP_035246839
Alphaproteobacteria_bacterium RME14878
Maritimibacter_sp._HL-12 WP_085525756
Rhodobacterales_bacterium PIE07976
Rhodobacterales_bacterium PID36751
Halomonas_sp. PHS12629
Aliiroseovarius_pelagivivens WP_108855540
Aliiroseovarius_sediminilitoris WP_091431376
Aliiroseovarius_crassostreae WP_055189930
Aliiroseovarius_crassostreae SFU87432
Celeribacter_baekdonensis WP_074641886
Celeribacter_baekdonensis WP_009570925
Celeribacter_neptunius WP_090055845
Celeribacter_halophilus SFJ02477
Celeribacter_halophilus WP_066603332
Oceanicola_sp._MCTG156(1a) WP_051982232
Aquimixticola_sp._Bin25-node5 WP_114285209
Confluentimicrobium_lipolyticum WP_093967447
Celeribacter_indicus WP_074743183
Celeribacter_marinus WP_062219408
Celeribacter_ethanolicus WP_066704296
Celeribacter_persicus WP_107816383
NonPhotosyntheticDiatom QUERY_NAA05P03950.m1
lysoplasmalogenase
0.3

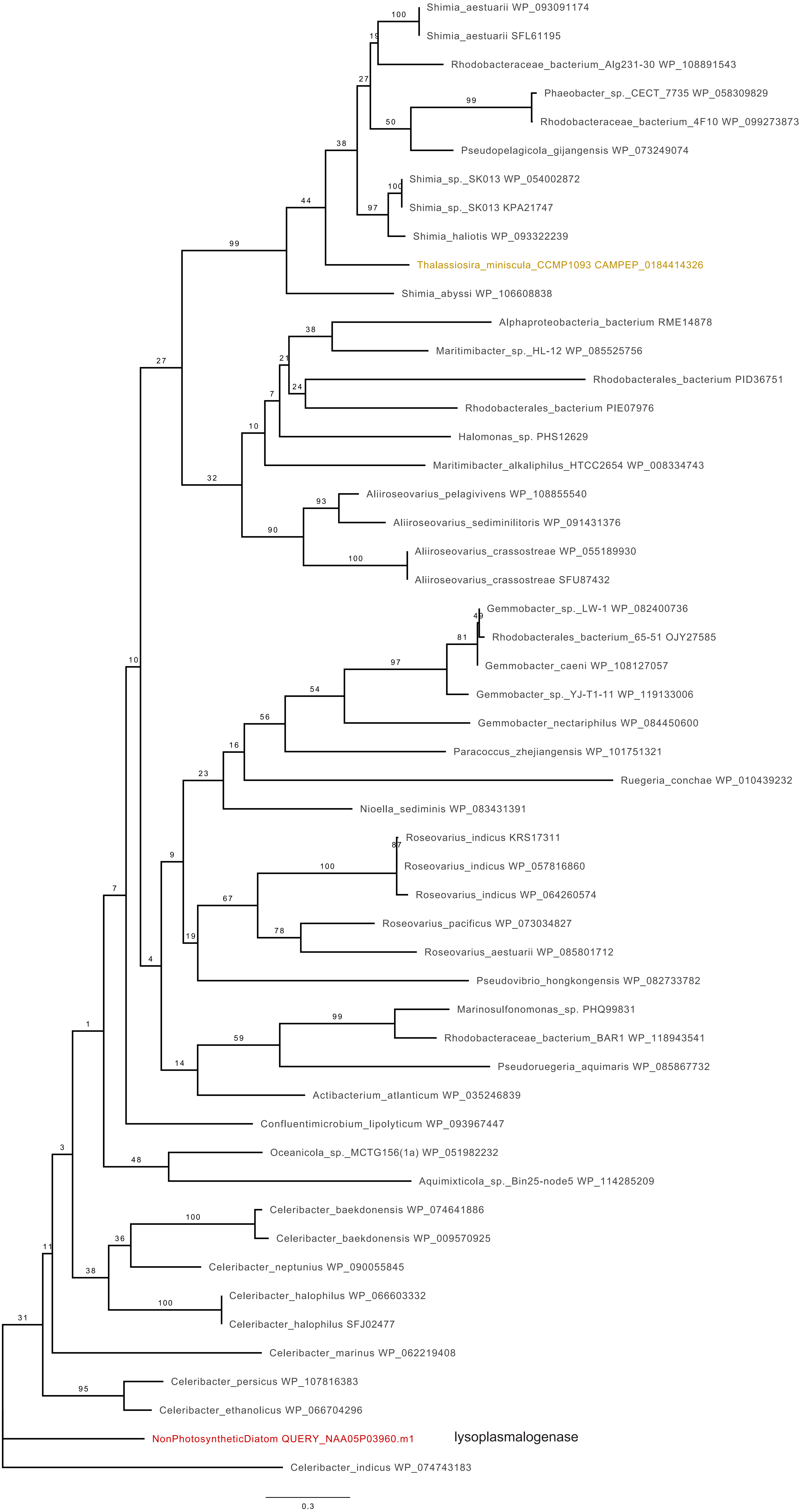

Shimia_aestuarii WP_093091174
Shimia_aestuarii SFL61195
Rhodobacteraceae_bacterium_Alg231-30 WP_108891543
Phaeobacter_sp._CECT_7735 WP_058309829
Rhodobacteraceae_bacterium_4F10 WP_099273873
Pseudopelagicola_gijangensis WP_073249074
Shimia_sp._SK013 WP_054002872
Shimia_sp._SK013 KPA21747
Shimia_haliotis WP_093322239
Thalassiosira_miniscula_CCMP1093 CAMPEP_0184414326
Shimia_abyssi WP_106608838
Alphaproteobacteria_bacterium RME14878
Maritimibacter_sp._HL-12 WP_085525756
Rhodobacterales_bacterium PID36751
Rhodobacterales_bacterium PIE07976
Halomonas_sp. PHS12629
Maritimibacter_alkaliphilus_HTCC2654 WP_008334743
Aliiroseovarius_pelagivivens WP_108855540
Aliiroseovarius_sediminilitoris WP_091431376
Aliiroseovarius_crassostreae WP_055189930
Aliiroseovarius_crassostreae SFU87432
Gemmobacter_sp._LW-1 WP_082400736
Rhodobacterales_bacterium_65-51 OJY27585
Gemmobacter_caeni WP_108127057
Gemmobacter_sp._YJ-T1-11 WP_119133006
Gemmobacter_nectariphilus WP_084450600
Paracoccus_zhejiangensis WP_101751321
Ruegeria_conchae WP_010439232
Nioella_sediminis WP_083431391
Roseovarius_indicus KRS17311
Roseovarius_indicus WP_057816860
Roseovarius_indicus WP_064260574
Roseovarius_pacificus WP_073034827
Roseovarius_aestuarii WP_085801712
Pseudovibrio_hongkongensis WP_082733782
Marinosulfonomonas_sp. PHQ99831
Rhodobacteraceae_bacterium_BAR1 WP_118943541
Pseudoruegeria_aquimaris WP_085867732
Actibacterium_atlanticum WP_035246839
Confluentimicrobium_lipolyticum WP_093967447
Oceanicola_sp._MCTG156(1a) WP_051982232
Aquimixticola_sp._Bin25-node5 WP_114285209
Celeribacter_baekdonensis WP_074641886
Celeribacter_baekdonensis WP_009570925
Celeribacter_neptunius WP_090055845
Celeribacter_halophilus WP_066603332
Celeribacter_halophilus SFJ02477
Celeribacter_marinus WP_062219408
Celeribacter_persicus WP_107816383
Celeribacter_ethanolicus WP_066704296
NonPhotosyntheticDiatom QUERY_NAA05P03960.m1
lysoplasmalogenase
Celeribacter_indicus WP_074743183
0.3

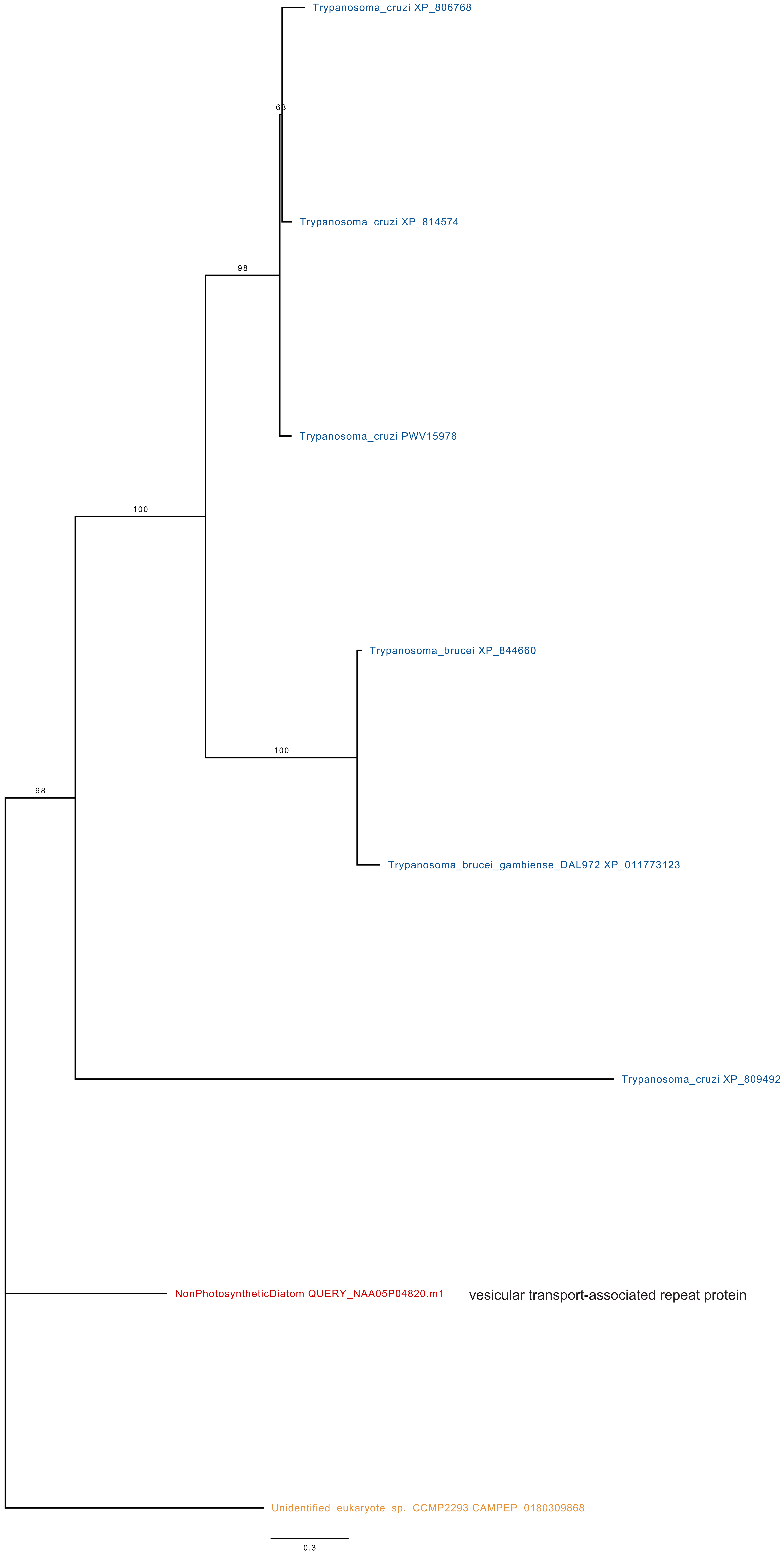
Trypanosoma_cruzi XP_806768
63
Trypanosoma_cruzi XP_814574
98
Trypanosoma_cruzi PWV15978
100
Trypanosoma_brucei XP_844660
100
98
Trypanosoma_brucei_gambiense_DAL972 XP_011773123
Trypanosoma_cruzi XP_809492
NonPhotosyntheticDiatom QUERY_NAA05P04820.m1
vesicular transport-associated repeat protein
Unidentified_eukaryote_sp._CCMP2293 CAMPEP_0180309868
0.3

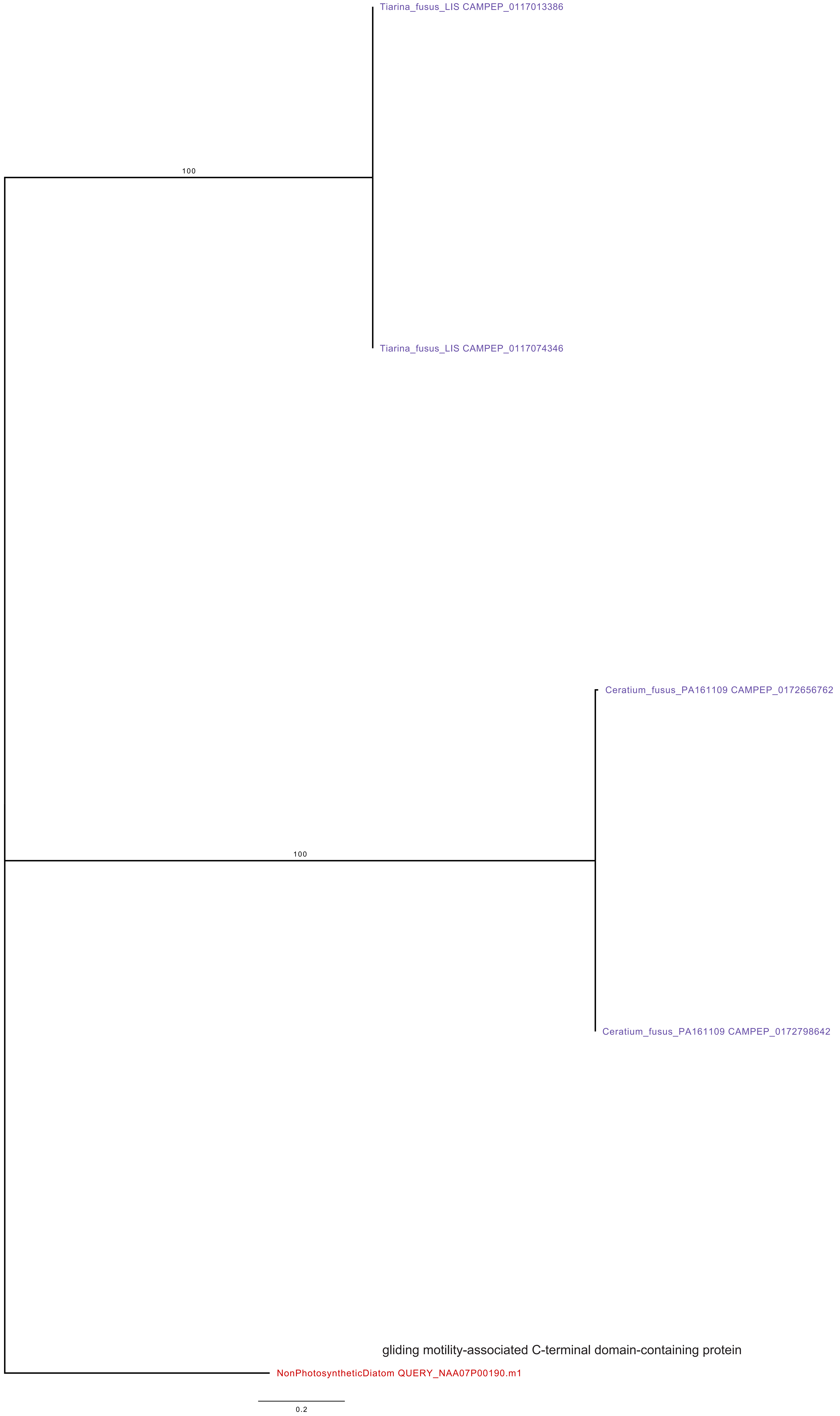
Tiarina_fusus_LIS CAMPEP_0117013386
100
Tiarina_fusus_LIS CAMPEP_0117074346
Ceratium_fusus_PA161109 CAMPEP_0172656762
100
Ceratium_fusus_PA161109 CAMPEP_0172798642
gliding motility-associated C-terminal domain-containing protein
NonPhotosyntheticDiatom QUERY_NAA07P00190.m1
0.2

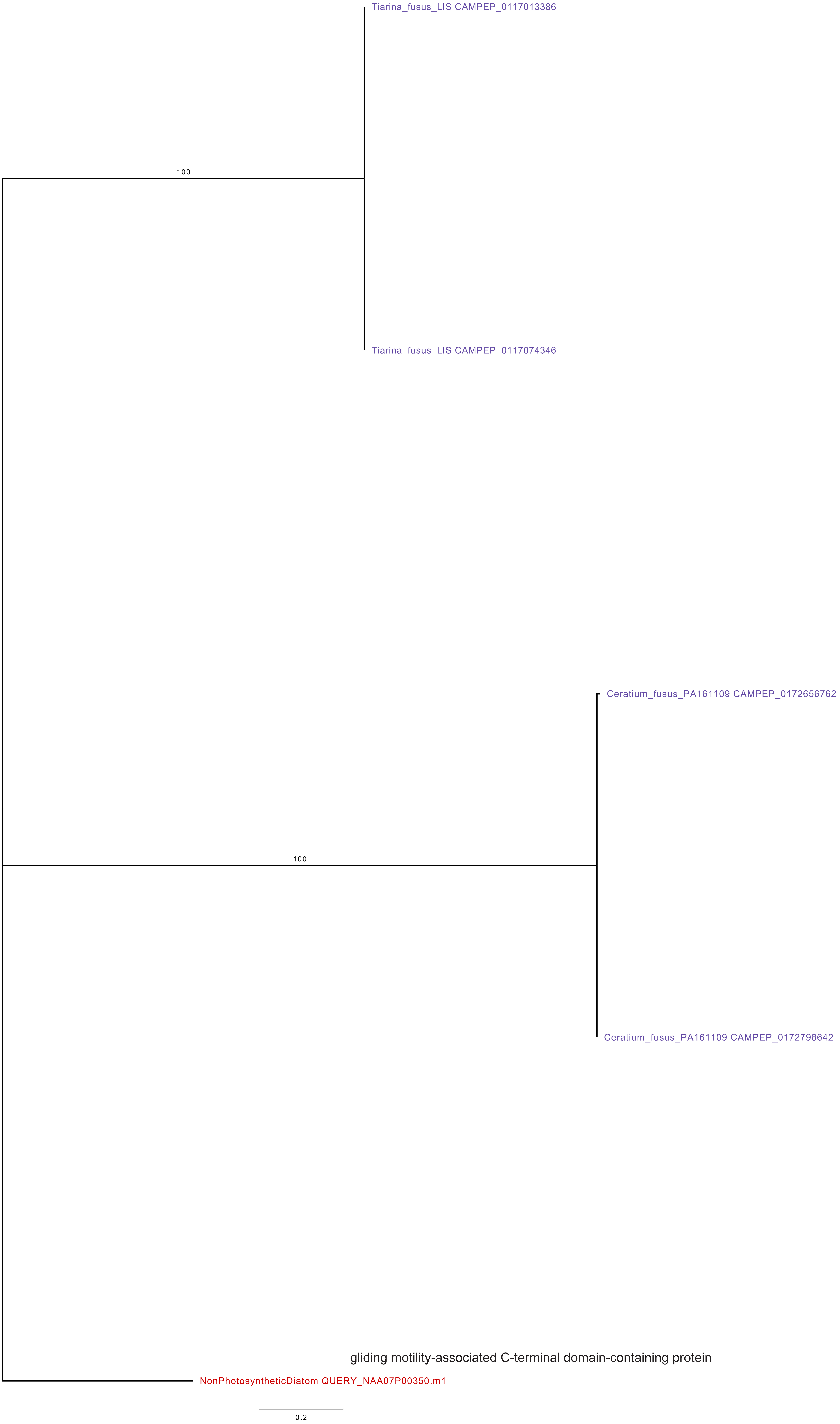
Tiarina_fusus_LIS CAMPEP_0117013386
100
Tiarina_fusus_LIS CAMPEP_0117074346
Ceratium_fusus_PA161109 CAMPEP_0172656762
100
Ceratium_fusus_PA161109 CAMPEP_0172798642
gliding motility-associated C-terminal domain-containing protein
NonPhotosyntheticDiatom QUERY_NAA07P00350.m1
0.2

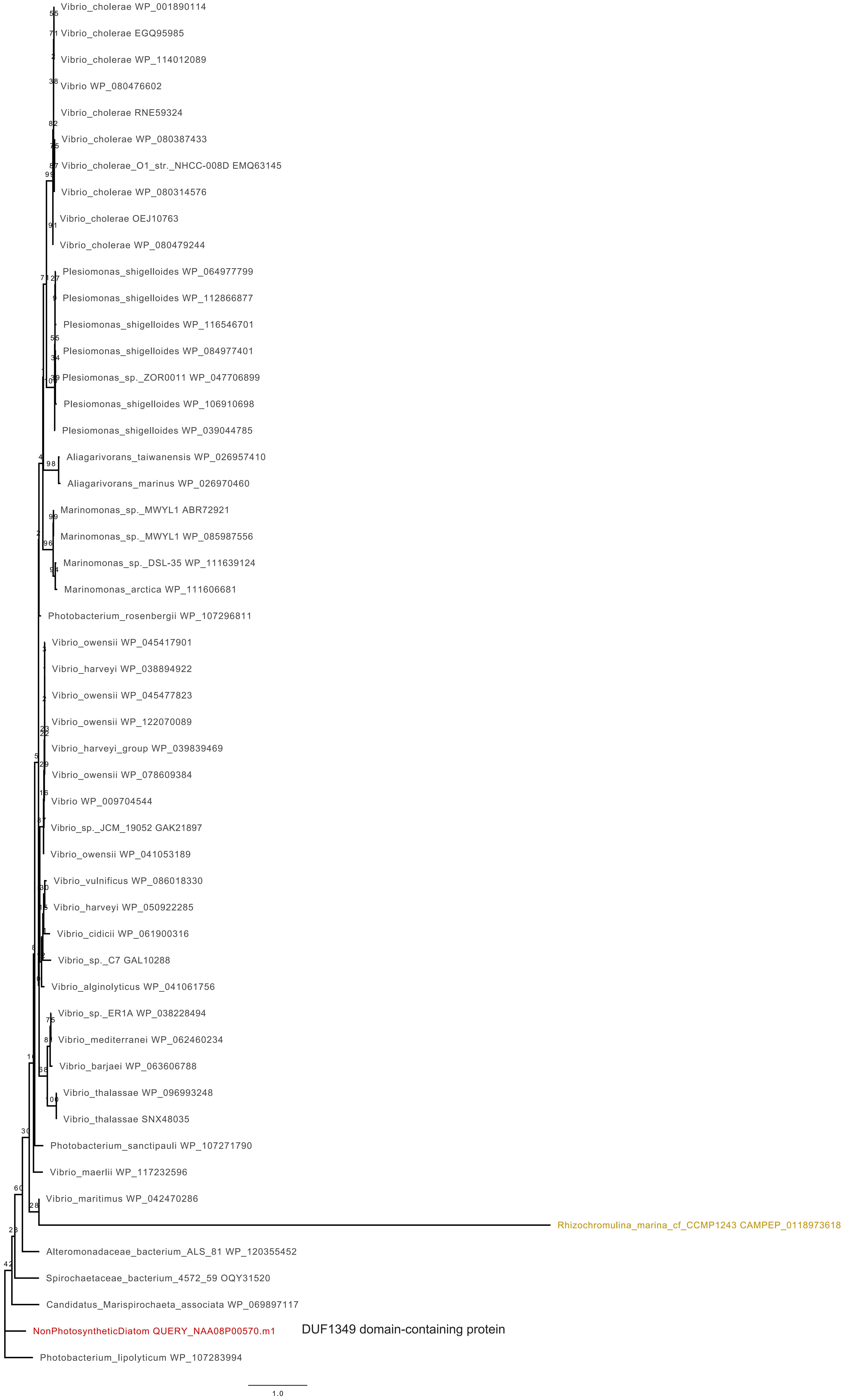

Vibrio_cholerae WP_001890114
Vibrio_cholerae EGQ95985
Vibrio_cholerae WP_114012089
Vibrio WP_080476602
Vibrio_cholerae RNE59324
Vibrio_cholerae WP_080387433
Vibrio_cholerae_O1_str._NHCC-008D EMQ63145
Vibrio_cholerae WP_080314576
Vibrio_cholerae OEJ10763
Vibrio_cholerae WP_080479244
Plesiomonas_shigelloides WP_064977799
Plesiomonas_shigelloides WP_112866877
Plesiomonas_shigelloides WP_116546701
Plesiomonas_shigelloides WP_084977401
Plesiomonas_sp._ZOR0011 WP_047706899
Plesiomonas_shigelloides WP_106910698
Plesiomonas_shigelloides WP_039044785
Aliagarivorans_taiwanensis WP_026957410
Aliagarivorans_marinus WP_026970460
Marinomonas_sp._MWYL1 ABR72921
Marinomonas_sp._MWYL1 WP_085987556
Marinomonas_sp._DSL-35 WP_111639124
Marinomonas_arctica WP_111606681
Photobacterium_rosenbergii WP_107296811
Vibrio_owensii WP_045417901
Vibrio_harveyi WP_038894922
Vibrio_owensii WP_045477823
Vibrio_owensii WP_122070089
Vibrio_harveyi_group WP_039839469
Vibrio_owensii WP_078609384
Vibrio WP_009704544
Vibrio_sp._JCM_19052 GAK21897
Vibrio_owensii WP_041053189
Vibrio_vulnificus WP_086018330
Vibrio_harveyi WP_050922285
Vibrio_cidicii WP_061900316
Vibrio_sp._C7 GAL10288
Vibrio_alginolyticus WP_041061756
Vibrio_sp._ER1A WP_038228494
Vibrio_mediterranei WP_062460234
Vibrio_barjaei WP_063606788
Vibrio_thalassae WP_096993248
Vibrio_thalassae SNX48035
Photobacterium_sanctipauli WP_107271790
Vibrio_maerlii WP_117232596
Vibrio_maritimus WP_042470286
Rhizochromulina_marina_cf_CCMP1243 CAMPEP_0118973618
Alteromonadaceae_bacterium_ALS_81 WP_120355452
Spirochaetaceae_bacterium_4572_59 OQY31520
Candidatus_Marispirochaeta_associata WP_069897117
NonPhotosyntheticDiatom QUERY_NAA08P00570.m1
DUF1349 domain-containing protein
Photobacterium_lipolyticum WP_107283994
1.0

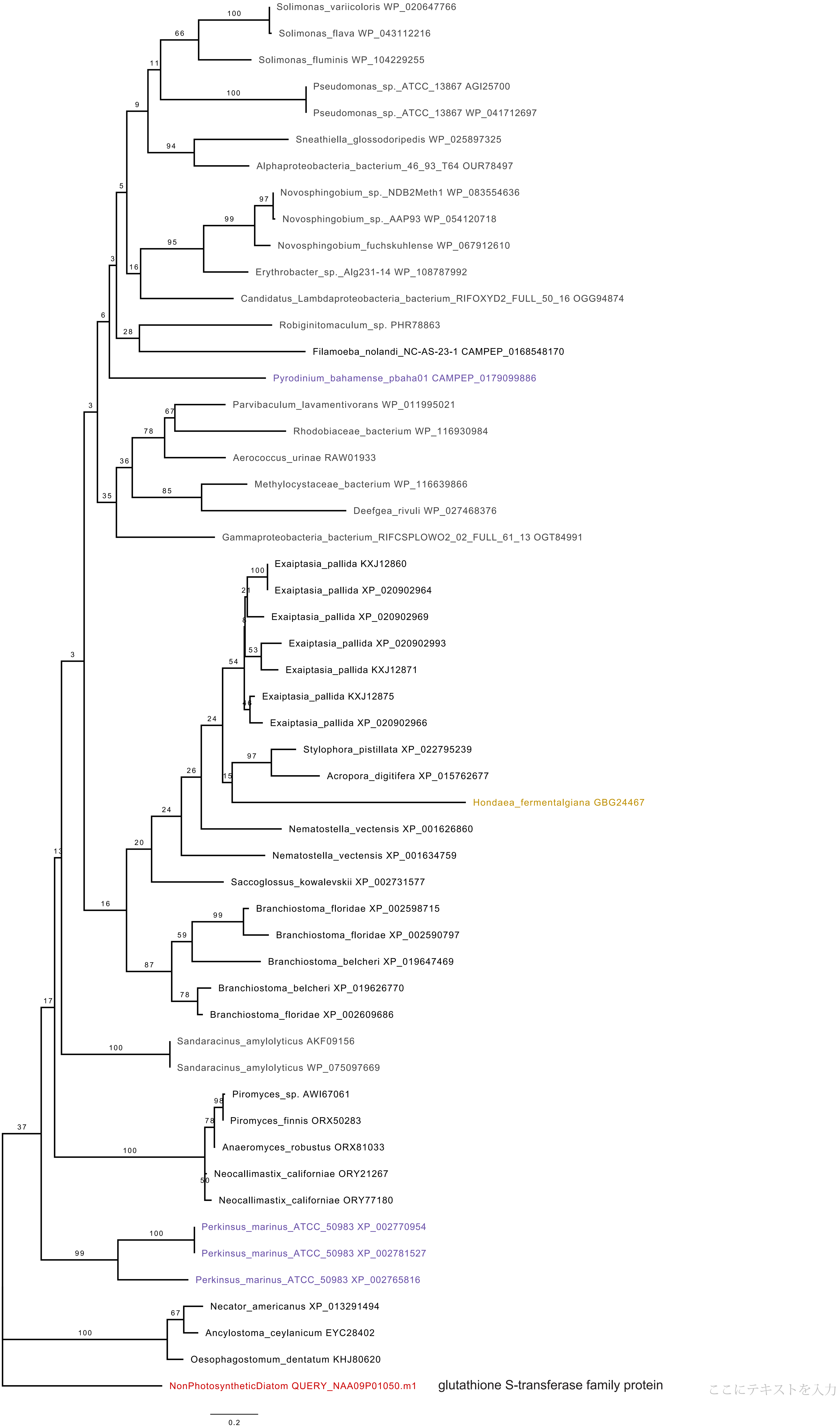
Solimonas_variicoloris WP_020647766
Solimonas_flava WP_043112216
Solimonas_fluminis WP_104229255
Pseudomonas_sp._ATCC_13867 AGI25700
Pseudomonas_sp._ATCC_13867 WP_041712697
Sneathiella_glossodoripedis WP_025897325
Alphaproteobacteria_bacterium_46_93_T64 OUR78497
Novosphingobium_sp._NDB2Meth1 WP_083554636
Novosphingobium_sp._AAP93 WP_054120718
Novosphingobium_fuchskuhlense WP_067912610
Erythrobacter_sp._Alg231-14 WP_108787992
Candidatus_Lambdaproteobacteria_bacterium_RIFOXYD2_FULL_50_16 OGG94874
Robiginitomaculum_sp. PHR78863
Filamoeba_nolandi_NC-AS-23-1 CAMPEP_0168548170
Pyrodinium_bahamense_pbaha01 CAMPEP_0179099886
Parvibaculum_lavamentivorans WP_011995021
Rhodobiaceae_bacterium WP_116930984
Aerococcus_urinae RAW01933
Methylocystaceae_bacterium WP_116639866
Deefgea_rivuli WP_027468376
Gammaproteobacteria_bacterium_RIFCSPLOWO2_02_FULL_61_13 OGT84991
Exaiptasia_pallida KXJ12860
Exaiptasia_pallida XP_020902964
Exaiptasia_pallida XP_020902969
Exaiptasia_pallida XP_020902993
Exaiptasia_pallida KXJ12871
Exaiptasia_pallida KXJ12875
Exaiptasia_pallida XP_020902966
Stylophora_pistillata XP_022795239
Acropora_digitifera XP_015762677
Hondaea_fermentalgiana GBG24467
Nematostella_vectensis XP_001626860
Nematostella_vectensis XP_001634759
Saccoglossus_kowalevskii XP_002731577
Branchiostoma_floridae XP_002598715
Branchiostoma_floridae XP_002590797
Branchiostoma_belcheri XP_019647469
Branchiostoma_belcheri XP_019626770
Branchiostoma_floridae XP_002609686
Sandaracinus_amylolyticus AKF09156
Sandaracinus_amylolyticus WP_075097669
Piromyces_sp. AWI67061
Piromyces_finnis ORX50283
Anaeromyces_robustus ORX81033
Neocallimastix_californiae ORY21267
Neocallimastix_californiae ORY77180
Perkinsus_marinus_ATCC_50983 XP_002770954
Perkinsus_marinus_ATCC_50983 XP_002781527
Perkinsus_marinus_ATCC_50983 XP_002765816
Necator_americanus XP_013291494
Ancylostoma_ceylanicum EYC28402
Oesophagostomum_dentatum KHJ80620
NonPhotosyntheticDiatom QUERY_NAA09P01050.m1
glutathione S-transferase family protein
ここにテキストを入力
0.2

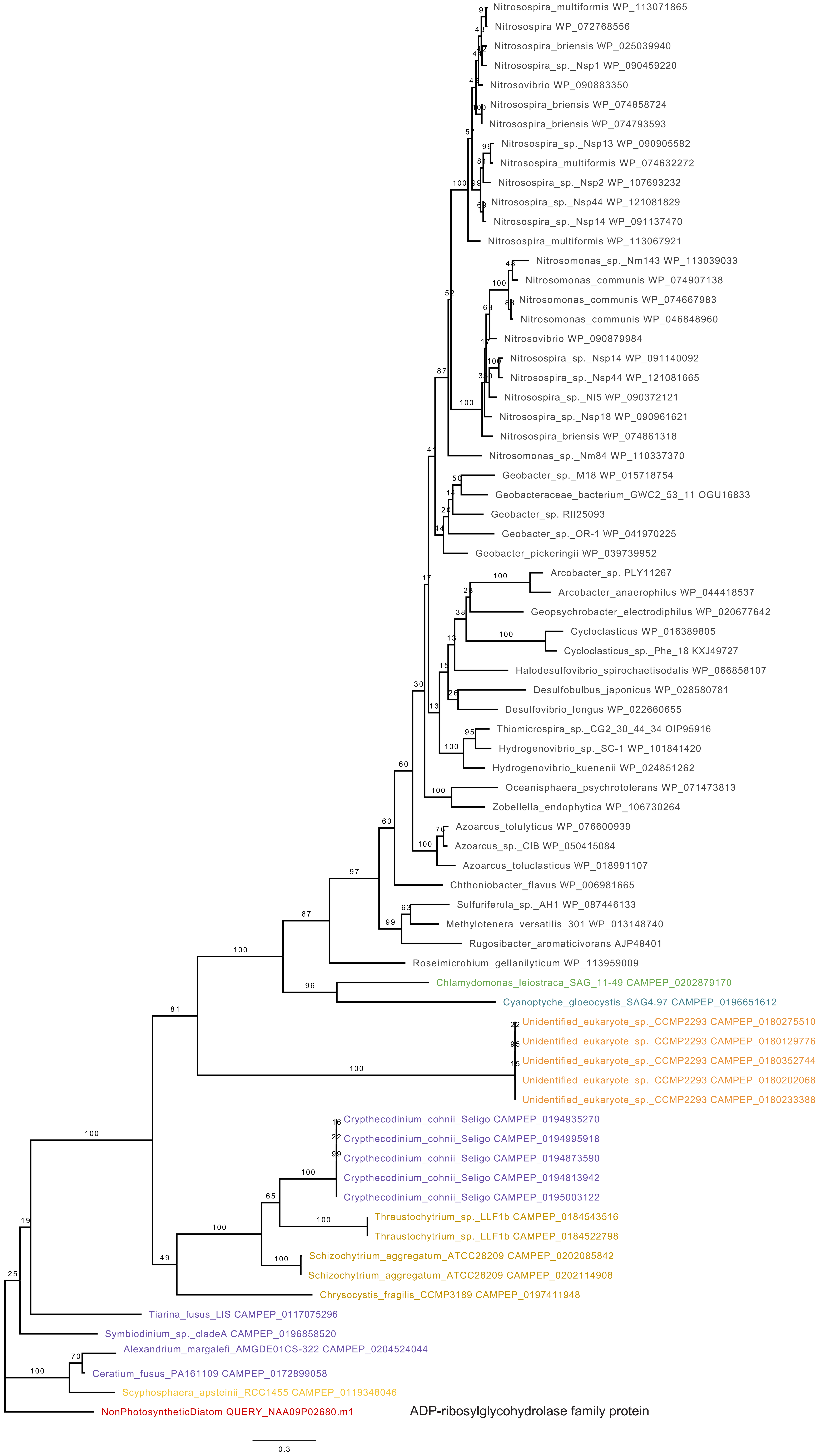

Nitrosospira_multiformis WP_113071865
Nitrosospira WP_072768556
Nitrosospira_briensis WP_025039940
Nitrosospira_sp._Nsp1 WP_090459220
Nitrosovibrio WP_090883350
Nitrosospira_briensis WP_074858724
Nitrosospira_briensis WP_074793593
Nitrosospira_sp._Nsp13 WP_090905582
Nitrosospira_multiformis WP_074632272
Nitrosospira_sp._Nsp2 WP_107693232
Nitrosospira_sp._Nsp44 WP_121081829
Nitrosospira_sp._Nsp14 WP_091137470
Nitrosospira_multiformis WP_113067921
Nitrosomonas_sp._Nm143 WP_113039033
Nitrosomonas_communis WP_074907138
Nitrosomonas_communis WP_074667983
Nitrosomonas_communis WP_046848960
Nitrosovibrio WP_090879984
Nitrosospira_sp._Nsp14 WP_091140092
Nitrosospira_sp._Nsp44 WP_121081665
Nitrosospira_sp._Nl5 WP_090372121
Nitrosospira_sp._Nsp18 WP_090961621
Nitrosospira_briensis WP_074861318
Nitrosomonas_sp._Nm84 WP_110337370
Geobacter_sp._M18 WP_015718754
Geobacteraceae_bacterium_GWC2_53_11 OGU16833
Geobacter_sp. RII25093
Geobacter_sp._OR-1 WP_041970225
Geobacter_pickeringii WP_039739952
Arcobacter_sp. PLY11267
Arcobacter_anaerophilus WP_044418537
Geopsychrobacter_electrodiphilus WP_020677642
Cycloclasticus WP_016389805
Cycloclasticus_sp._Phe_18 KXJ49727
Halodesulfovibrio_spirochaetisodalis WP_066858107
Desulfobulbus_japonicus WP_028580781
Desulfovibrio_longus WP_022660655
Thiomicrospira_sp._CG2_30_44_34 OIP95916
Hydrogenovibrio_sp._SC-1 WP_101841420
Hydrogenovibrio_kuenenii WP_024851262
Oceanisphaera_psychrotolerans WP_071473813
Zobellella_endophytica WP_106730264
Azoarcus_tolulyticus WP_076600939
Azoarcus_sp._CIB WP_050415084
Azoarcus_toluclasticus WP_018991107
Chthoniobacter_flavus WP_006981665
Sulfuriferula_sp._AH1 WP_087446133
Methylotenera_versatilis_301 WP_013148740
Rugosibacter_aromaticivorans AJP48401
Roseimicrobium_gellanilyticum WP_113959009
Chlamydomonas_leiostraca_SAG_11-49 CAMPEP_0202879170
Cyanoptyche_gloeocystis_SAG4.97 CAMPEP_0196651612
Unidentified_eukaryote_sp._CCMP2293 CAMPEP_0180275510
Unidentified_eukaryote_sp._CCMP2293 CAMPEP_0180129776
Unidentified_eukaryote_sp._CCMP2293 CAMPEP_0180352744
Unidentified_eukaryote_sp._CCMP2293 CAMPEP_0180202068
Unidentified_eukaryote_sp._CCMP2293 CAMPEP_0180233388
Crypthecodinium_cohnii_Seligo CAMPEP_0194935270
Crypthecodinium_cohnii_Seligo CAMPEP_0194995918
Crypthecodinium_cohnii_Seligo CAMPEP_0194873590
Crypthecodinium_cohnii_Seligo CAMPEP_0194813942
Crypthecodinium_cohnii_Seligo CAMPEP_0195003122
Thraustochytrium_sp._LLF1b CAMPEP_0184543516
Thraustochytrium_sp._LLF1b CAMPEP_0184522798
Schizochytrium_aggregatum_ATCC28209 CAMPEP_0202085842
Schizochytrium_aggregatum_ATCC28209 CAMPEP_0202114908
Chrysocystis_fragilis_CCMP3189 CAMPEP_0197411948
Tiarina_fusus_LIS CAMPEP_0117075296
Symbiodinium_sp._cladeA CAMPEP_0196858520
Alexandrium_margalefi_AMGDE01CS-322 CAMPEP_0204524044
Ceratium_fusus_PA161109 CAMPEP_0172899058
Scyphosphaera_apsteinii_RCC1455 CAMPEP_0119348046
NonPhotosyntheticDiatom QUERY_NAA09P02680.m1
ADP-ribosylglycohydrolase family protein
0.3

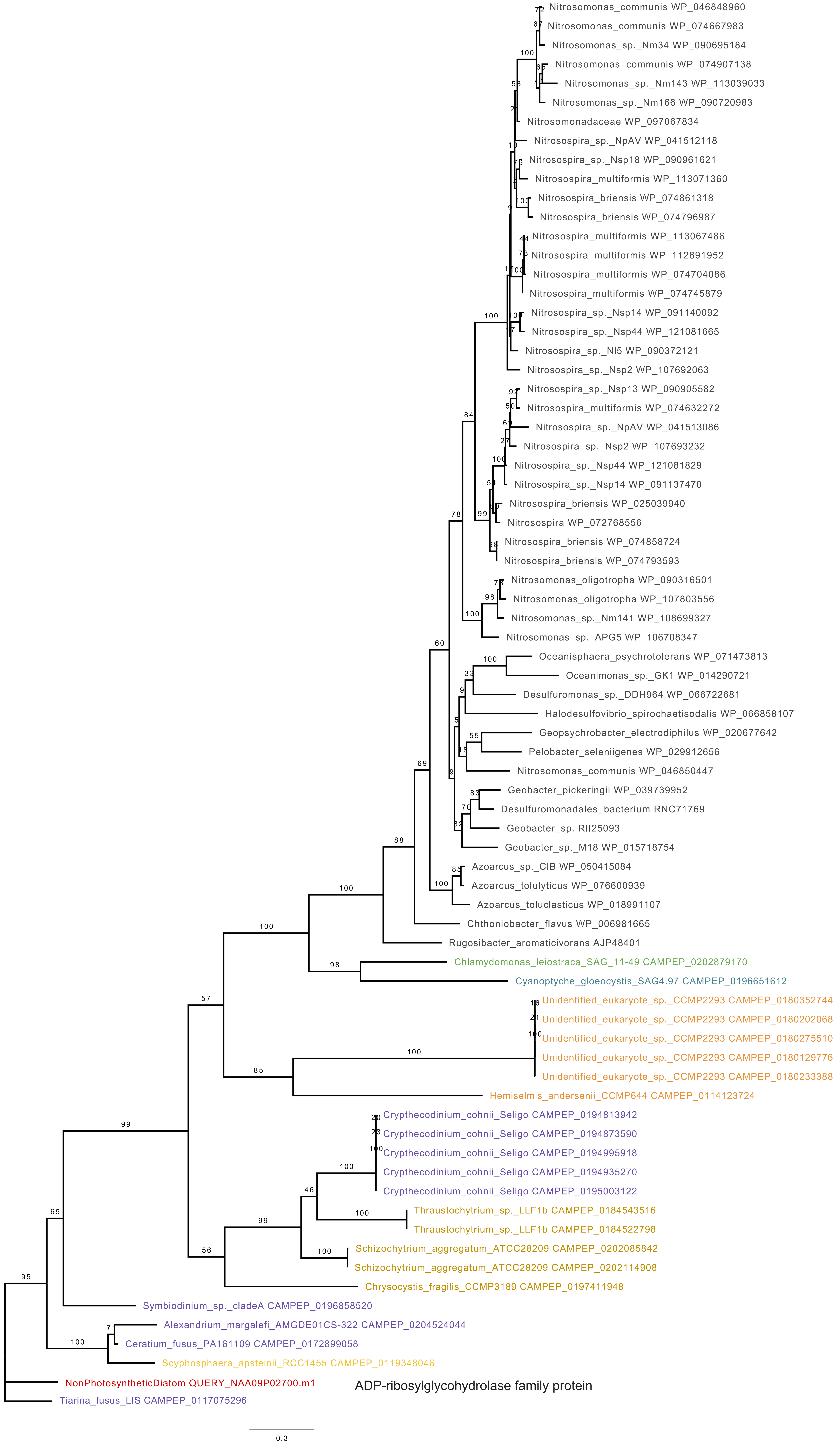
Nitrosomonas_communis WP_046848960
Nitrosomonas_communis WP_074667983
Nitrosomonas_sp._Nm34 WP_090695184
Nitrosomonas_communis WP_074907138
Nitrosomonas_sp._Nm143 WP_113039033
Nitrosomonas_sp._Nm166 WP_090720983
Nitrosomonadaceae WP_097067834
Nitrosospira_sp._NpAV WP_041512118
Nitrosospira_sp._Nsp18 WP_090961621
Nitrosospira_multiformis WP_113071360
Nitrosospira_briensis WP_074861318
Nitrosospira_briensis WP_074796987
Nitrosospira_multiformis WP_113067486
Nitrosospira_multiformis WP_112891952
Nitrosospira_multiformis WP_074704086
Nitrosospira_multiformis WP_074745879
Nitrosospira_sp._Nsp14 WP_091140092
Nitrosospira_sp._Nsp44 WP_121081665
Nitrosospira_sp._NI5 WP_090372121
Nitrosospira_sp._Nsp2 WP_107692063
Nitrosospira_sp._Nsp13 WP_090905582
Nitrosospira_multiformis WP_074632272
Nitrosospira_sp._NpAV WP_041513086
Nitrosospira_sp._Nsp2 WP_107693232
Nitrosospira_sp._Nsp44 WP_121081829
Nitrosospira_sp._Nsp14 WP_091137470
Nitrosospira_briensis WP_025039940
Nitrosospira WP_072768556
Nitrosospira_briensis WP_074858724
Nitrosospira_briensis WP_074793593
Nitrosomonas_oligotropha WP_090316501
Nitrosomonas_oligotropha WP_107803556
Nitrosomonas_sp._Nm141 WP_108699327
Nitrosomonas_sp._APG5 WP_106708347
Oceanisphaera_psychrotolerans WP_071473813
Oceanimonas_sp._GK1 WP_014290721
Desulfuromonas_sp._DDH964 WP_066722681
Halodesulfovibrio_spirochaetisodalis WP_066858107
Geopsychrobacter_electrodiphilus WP_020677642
Pelobacter_seleniigenes WP_029912656
Nitrosomonas_communis WP_046850447
Geobacter_pickeringii WP_039739952
Desulfuromonadales_bacterium RNC71769
Geobacter_sp. RII25093
Geobacter_sp._M18 WP_015718754
Azoarcus_sp._CIB WP_050415084
Azoarcus_tolulyticus WP_076600939
Azoarcus_toluclasticus WP_018991107
Chthoniobacter_flavus WP_006981665
Rugosibacter_aromaticivorans AJP48401
Chlamydomonas_leiostraca_SAG_11-49 CAMPEP_0202879170
Cyanoptyche_gloeocystis_SAG4.97 CAMPEP_0196651612
Unidentified_eukaryote_sp._CCMP2293 CAMPEP_0180352744
Unidentified_eukaryote_sp._CCMP2293 CAMPEP_0180202068
Unidentified_eukaryote_sp._CCMP2293 CAMPEP_0180275510
Unidentified_eukaryote_sp._CCMP2293 CAMPEP_0180129776
Unidentified_eukaryote_sp._CCMP2293 CAMPEP_0180233388
Hemiselmis_andersenii_CCMP644 CAMPEP_0114123724
Crypthecodinium_cohnii_Seligo CAMPEP_0194813942
Crypthecodinium_cohnii_Seligo CAMPEP_0194873590
Crypthecodinium_cohnii_Seligo CAMPEP_0194995918
Crypthecodinium_cohnii_Seligo CAMPEP_0194935270
Crypthecodinium_cohnii_Seligo CAMPEP_0195003122
Thraustochytrium_sp._LLF1b CAMPEP_0184543516
Thraustochytrium_sp._LLF1b CAMPEP_0184522798
Schizochytrium_aggregatum_ATCC28209 CAMPEP_0202085842
Schizochytrium_aggregatum_ATCC28209 CAMPEP_0202114908
Chrysocystis_fragilis_CCMP3189 CAMPEP_0197411948
Symbiodinium_sp._cladeA CAMPEP_0196858520
Alexandrium_margalefi_AMGDE01CS-322 CAMPEP_0204524044
Ceratium_fusus_PA161109 CAMPEP_0172899058
Scyphosphaera_apsteinii_RCC1455 CAMPEP_0119348046
NonPhotosyntheticDiatom QUERY_NAA09P02700.m1
ADP-ribosylglycohydrolase family protein
Tiarina_fusus_LIS CAMPEP_0117075296
0.3

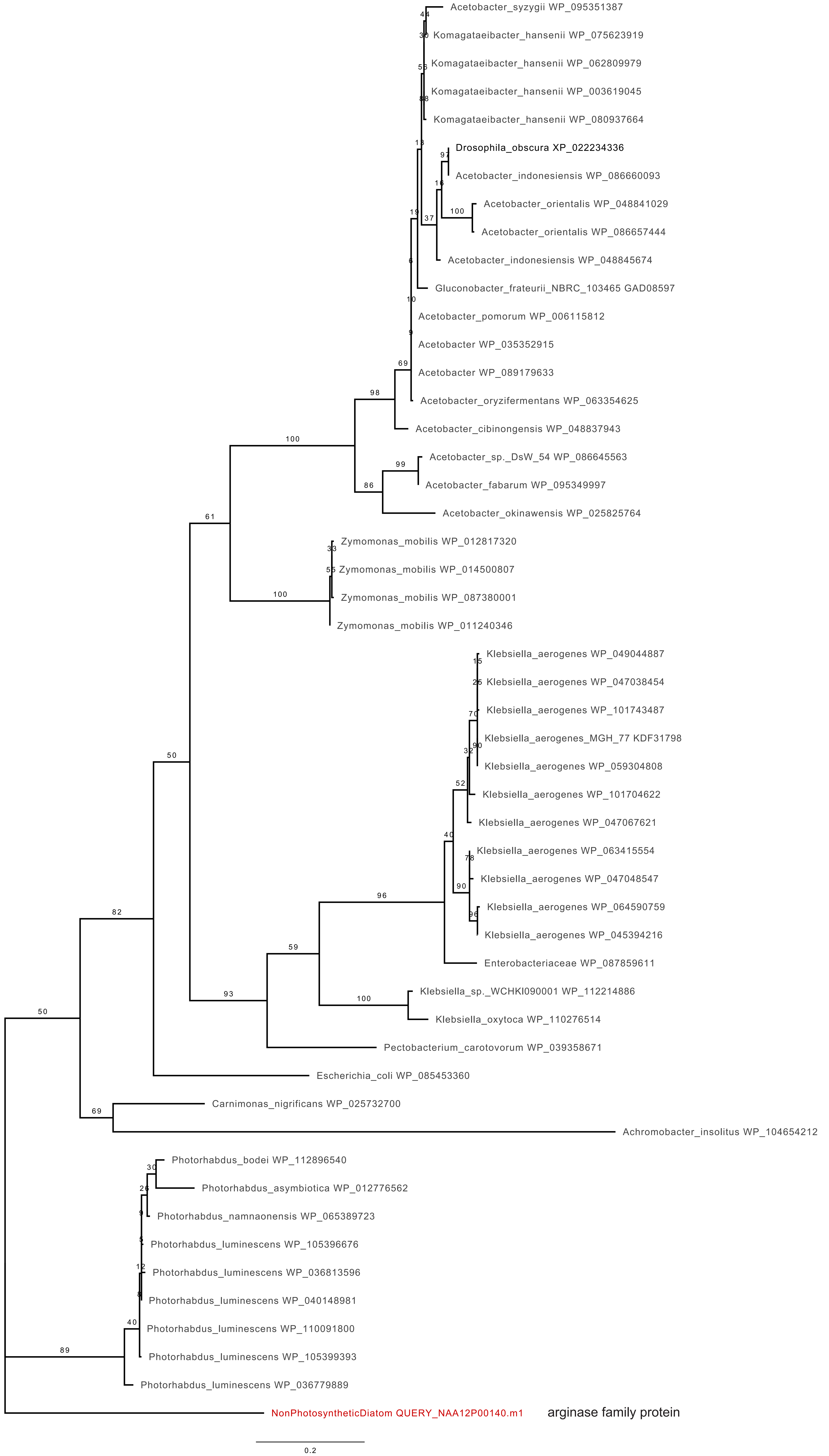
Acetobacter_syzygii WP_095351387
Komagataeibacter_hansenii WP_075623919
Komagataeibacter_hansenii WP_062809979
Komagataeibacter_hansenii WP_003619045
Komagataeibacter_hansenii WP_080937664
Drosophila_obscura XP_022234336
Acetobacter_indonesiensis WP_086660093
Acetobacter_orientalis WP_048841029
Acetobacter_orientalis WP_086657444
Acetobacter_indonesiensis WP_048845674
Gluconobacter_frateurii_NBRC_103465 GAD08597
Acetobacter_pomorum WP_006115812
Acetobacter WP_035352915
Acetobacter WP_089179633
Acetobacter_oryzifermentans WP_063354625
Acetobacter_cibinongensis WP_048837943
Acetobacter_sp._DsW_54 WP_086645563
Acetobacter_fabarum WP_095349997
Acetobacter_okinawensis WP_025825764
Zymomonas_mobilis WP_012817320
Zymomonas_mobilis WP_014500807
Zymomonas_mobilis WP_087380001
Zymomonas_mobilis WP_011240346
Klebsiella_aerogenes WP_049044887
Klebsiella_aerogenes WP_047038454
Klebsiella_aerogenes WP_101743487
Klebsiella_aerogenes_MGH_77 KDF31798
Klebsiella_aerogenes WP_059304808
Klebsiella_aerogenes WP_101704622
Klebsiella_aerogenes WP_047067621
Klebsiella_aerogenes WP_063415554
Klebsiella_aerogenes WP_047048547
Klebsiella_aerogenes WP_064590759
Klebsiella_aerogenes WP_045394216
Enterobacteriaceae WP_087859611
Klebsiella_sp._WCHKI090001 WP_112214886
Klebsiella_oxytoca WP_110276514
Pectobacterium_carotovorum WP_039358671
Escherichia_coli WP_085453360
Carnimonas_nigrificans WP_025732700
Achromobacter_insolitus WP_104654212
Photorhabdus_bodei WP_112896540
Photorhabdus_asymbiotica WP_012776562
Photorhabdus_namnaonensis WP_065389723
Photorhabdus_luminescens WP_105396676
Photorhabdus_luminescens WP_036813596
Photorhabdus_luminescens WP_040148981
Photorhabdus_luminescens WP_110091800
Photorhabdus_luminescens WP_105399393
Photorhabdus_luminescens WP_036779889
NonPhotosyntheticDiatom QUERY_NAA12P00140.m1
arginase family protein
0.2

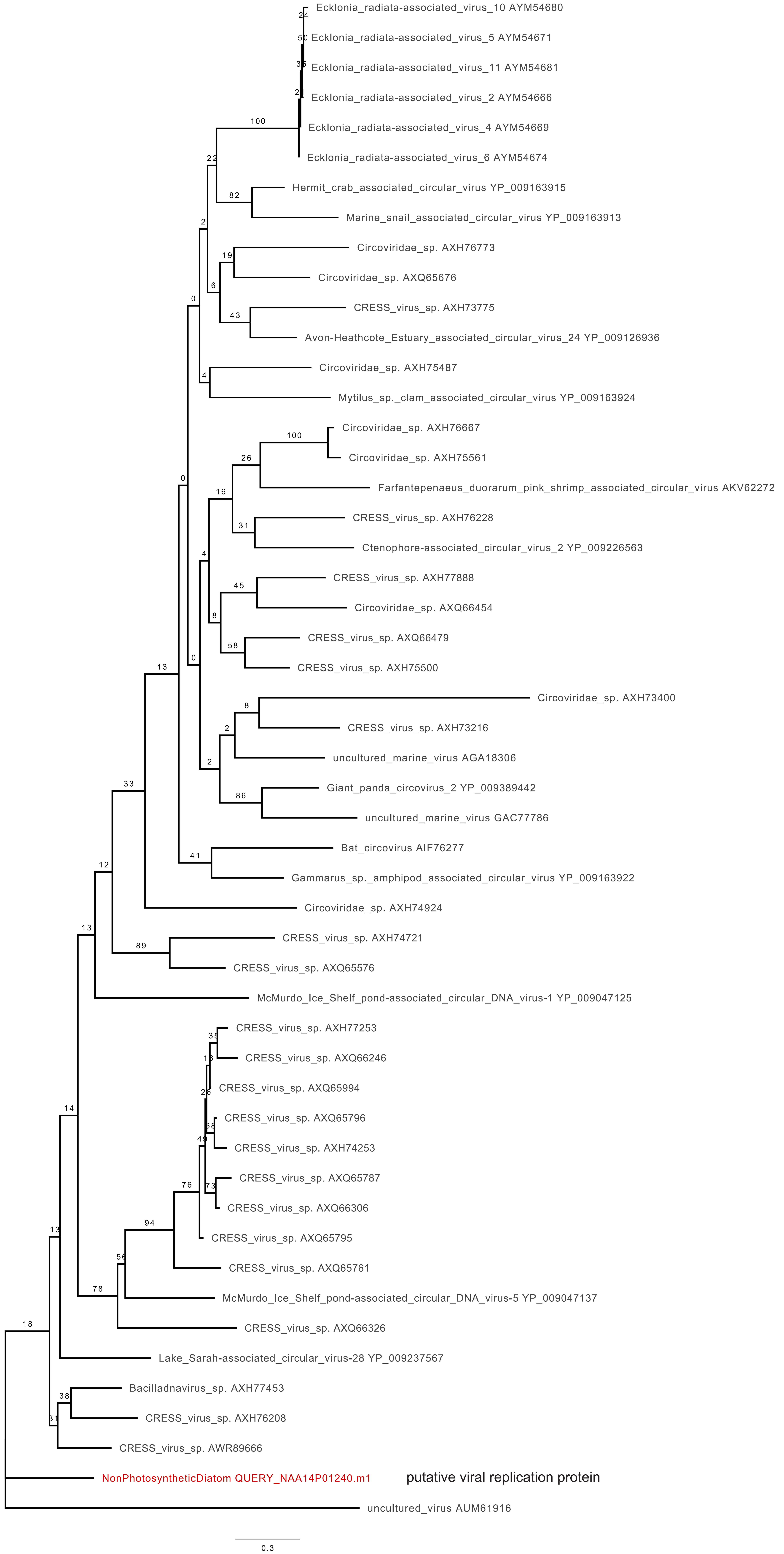
Ecklonia_radiata-associated_virus_10 AYM54680
Ecklonia_radiata-associated_virus_5 AYM54671
Ecklonia_radiata-associated_virus_11 AYM54681
Ecklonia_radiata-associated_virus_2 AYM54666
Ecklonia_radiata-associated_virus_4 AYM54669
Ecklonia_radiata-associated_virus_6 AYM54674
Hermit_crab_associated_circular_virus YP_009163915
Marine_snail_associated_circular_virus YP_009163913
Circoviridae_sp. AXH76773
Circoviridae_sp. AXQ65676
CRESS_virus_sp. AXH73775
Avon-Heathcote_Estuary_associated_circular_virus_24 YP_009126936
Circoviridae_sp. AXH75487
Mytilus_sp._clam_associated_circular_virus YP_009163924
Circoviridae_sp. AXH76667
Circoviridae_sp. AXH75561
Farfantepenaeus_duorarum_pink_shrimp_associated_circular_virus AKV62272
CRESS_virus_sp. AXH76228
Ctenophore-associated_circular_virus_2 YP_009226563
CRESS_virus_sp. AXH77888
Circoviridae_sp. AXQ66454
CRESS_virus_sp. AXQ66479
CRESS_virus_sp. AXH75500
Circoviridae_sp. AXH73400
CRESS_virus_sp. AXH73216
uncultured_marine_virus AGA18306
Giant_panda_circovirus_2 YP_009389442
uncultured_marine_virus GAC77786
Bat_circovirus AIF76277
Gammarus_sp._amphipod_associated_circular_virus YP_009163922
Circoviridae_sp. AXH74924
CRESS_virus_sp. AXH74721
CRESS_virus_sp. AXQ65576
McMurdo_Ice_Shelf_pond-associated_circular_DNA_virus-1 YP_009047125
CRESS_virus_sp. AXH77253
CRESS_virus_sp. AXQ66246
CRESS_virus_sp. AXQ65994
CRESS_virus_sp. AXQ65796
CRESS_virus_sp. AXH74253
CRESS_virus_sp. AXQ65787
CRESS_virus_sp. AXQ66306
CRESS_virus_sp. AXQ65795
CRESS_virus_sp. AXQ65761
McMurdo_Ice_Shelf_pond-associated_circular_DNA_virus-5 YP_009047137
CRESS_virus_sp. AXQ66326
Lake_Sarah-associated_circular_virus-28 YP_009237567
Bacilladnavirus_sp. AXH77453
CRESS_virus_sp. AXH76208
CRESS_virus_sp. AWR89666
NonPhotosyntheticDiatom QUERY_NAA14P01240.m1
putative viral replication protein
uncultured_virus AUM61916
0.3

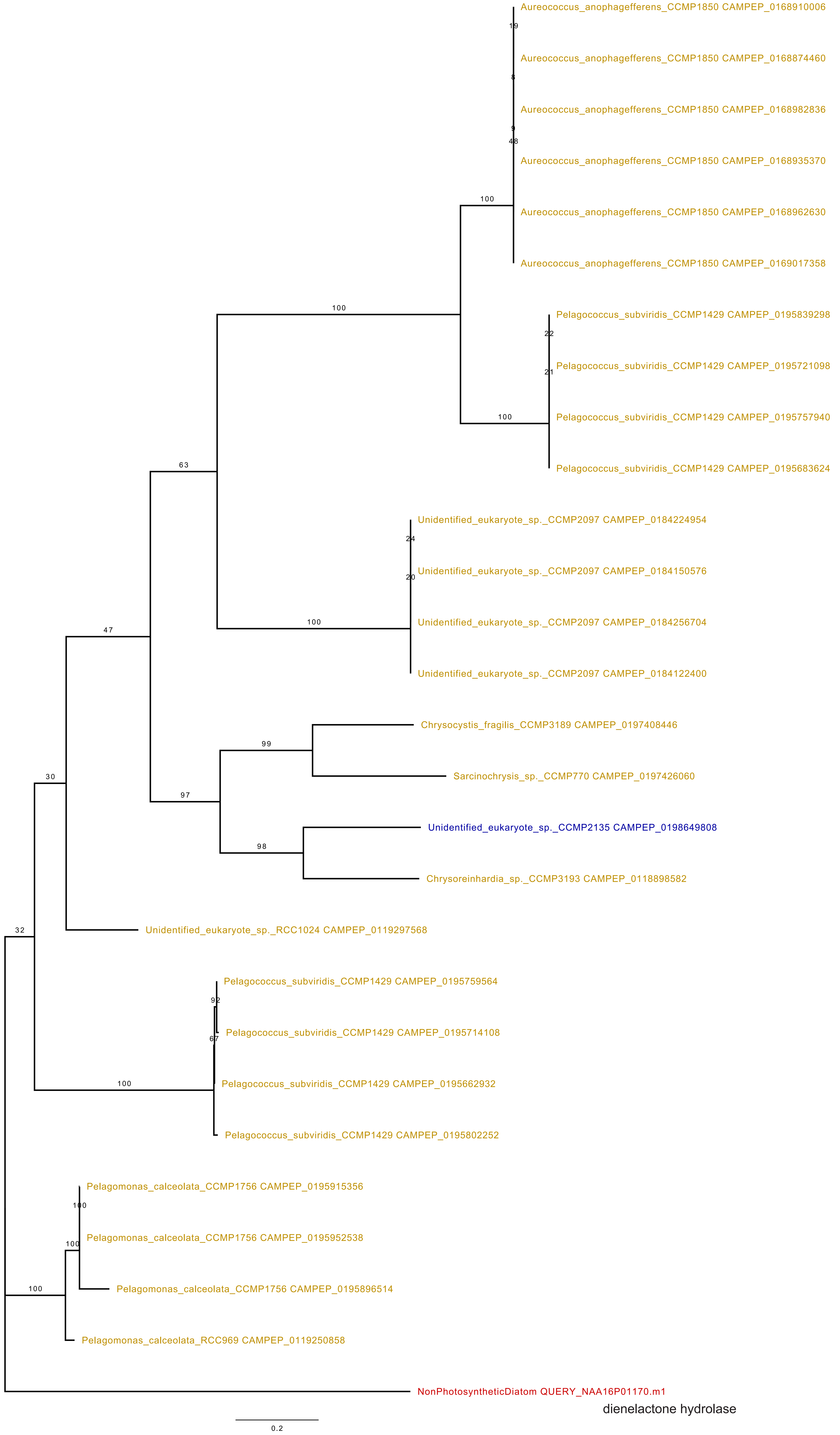
Aureococcus_anophagefferens_CCMP1850 CAMPEP_0168910006
Aureococcus_anophagefferens_CCMP1850 CAMPEP_0168874460
Aureococcus_anophagefferens_CCMP1850 CAMPEP_0168982836
Aureococcus_anophagefferens_CCMP1850 CAMPEP_0168935370
Aureococcus_anophagefferens_CCMP1850 CAMPEP_0168962630
Aureococcus_anophagefferens_CCMP1850 CAMPEP_0169017358
Pelagococcus_subviridis_CCMP1429 CAMPEP_0195839298
Pelagococcus_subviridis_CCMP1429 CAMPEP_0195721098
Pelagococcus_subviridis_CCMP1429 CAMPEP_0195757940
Pelagococcus_subviridis_CCMP1429 CAMPEP_0195683624
Unidentified_eukaryote_sp._CCMP2097 CAMPEP_0184224954
Unidentified_eukaryote_sp._CCMP2097 CAMPEP_0184150576
Unidentified_eukaryote_sp._CCMP2097 CAMPEP_0184256704
Unidentified_eukaryote_sp._CCMP2097 CAMPEP_0184122400
Chrysocystis_fragilis_CCMP3189 CAMPEP_0197408446
Sarcinochrysis_sp._CCMP770 CAMPEP_0197426060
Unidentified_eukaryote_sp._CCMP2135 CAMPEP_0198649808
Chrysoreinhardia_sp._CCMP3193 CAMPEP_0118898582
Unidentified_eukaryote_sp._RCC1024 CAMPEP_0119297568
Pelagococcus_subviridis_CCMP1429 CAMPEP_0195759564
Pelagococcus_subviridis_CCMP1429 CAMPEP_0195714108
Pelagococcus_subviridis_CCMP1429 CAMPEP_0195662932
Pelagococcus_subviridis_CCMP1429 CAMPEP_0195802252
Pelagomonas_calceolata_CCMP1756 CAMPEP_0195915356
Pelagomonas_calceolata_CCMP1756 CAMPEP_0195952538
Pelagomonas_calceolata_CCMP1756 CAMPEP_0195896514
Pelagomonas_calceolata_RCC969 CAMPEP_0119250858
NonPhotosyntheticDiatom QUERY_NAA16P01170.m1
0.2
dienelactone hydrolase

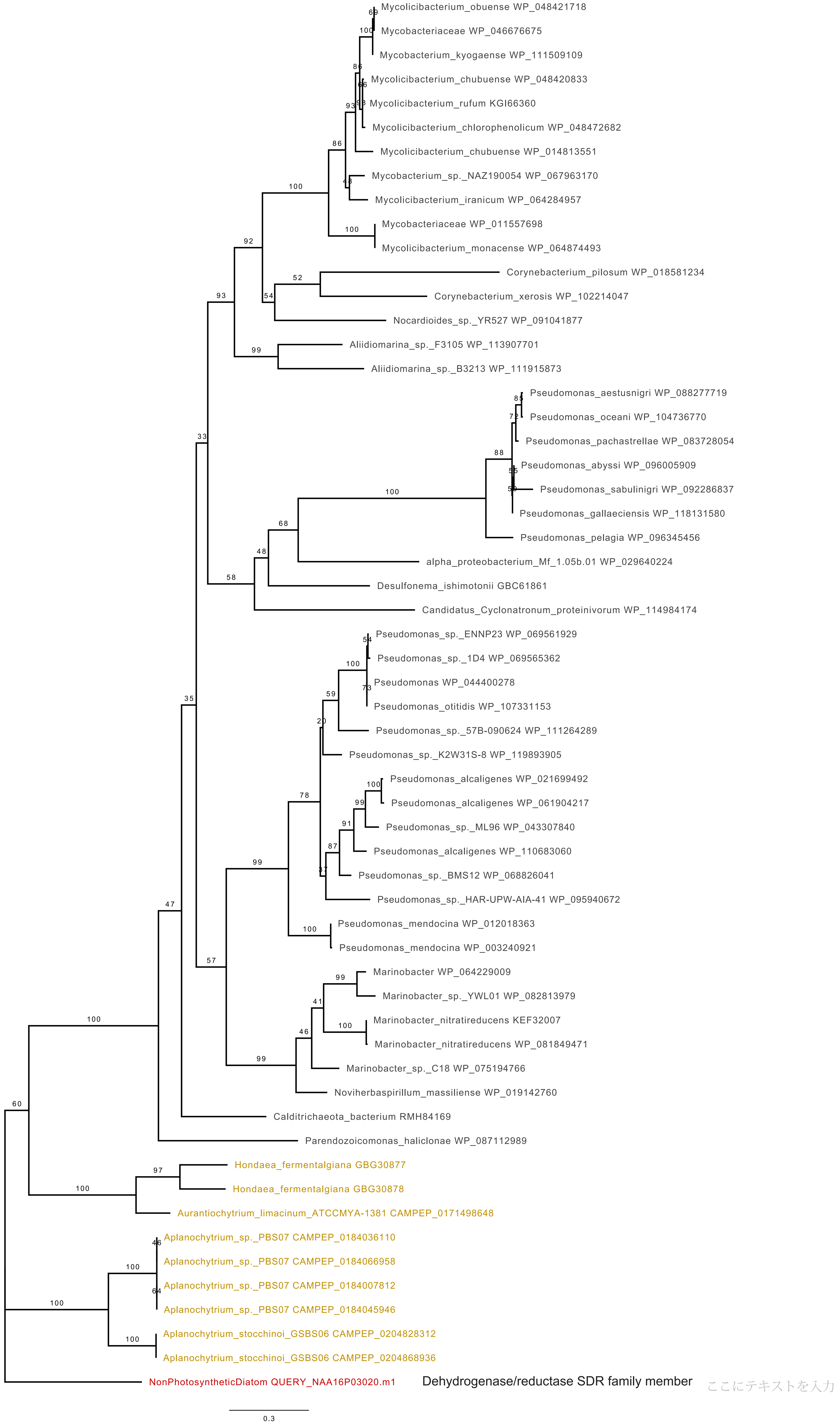
Mycolicibacterium_obuense WP_048421718
Mycobacteriaceae WP_046676675
Mycobacterium_kyogaense WP_111509109
Mycolicibacterium_chubuense WP_048420833
Mycolicibacterium_rufum KGI66360
Mycolicibacterium_chlorophenolicum WP_048472682
Mycolicibacterium_chubuense WP_014813551
Mycobacterium_sp._NAZ190054 WP_067963170
Mycolicibacterium_iranicum WP_064284957
Mycobacteriaceae WP_011557698
Mycolicibacterium_monacense WP_064874493
Corynebacterium_pilosum WP_018581234
Corynebacterium_xerosis WP_102214047
Nocardioides_sp._YR527 WP_091041877
Aliidiomarina_sp._F3105 WP_113907701
Aliidiomarina_sp._B3213 WP_111915873
Pseudomonas_aestusnigri WP_088277719
Pseudomonas_oceani WP_104736770
Pseudomonas_pachastrellae WP_083728054
Pseudomonas_abyssi WP_096005909
Pseudomonas_sabulinigri WP_092286837
Pseudomonas_gallaeciensis WP_118131580
Pseudomonas_pelagia WP_096345456
alpha_proteobacterium_Mf_1.05b.01 WP_029640224
Desulfonema_ishimotonii GBC61861
Candidatus_Cyclonatronum_proteinivorum WP_114984174
Pseudomonas_sp._ENNP23 WP_069561929
Pseudomonas_sp._1D4 WP_069565362
Pseudomonas WP_044400278
Pseudomonas_otitidis WP_107331153
Pseudomonas_sp._57B-090624 WP_111264289
Pseudomonas_sp._K2W31S-8 WP_119893905
Pseudomonas_alcaligenes WP_021699492
Pseudomonas_alcaligenes WP_061904217
Pseudomonas_sp._ML96 WP_043307840
Pseudomonas_alcaligenes WP_110683060
Pseudomonas_sp._BMS12 WP_068826041
Pseudomonas_sp._HAR-UPW-AIA-41 WP_095940672
Pseudomonas_mendocina WP_012018363
Pseudomonas_mendocina WP_003240921
Marinobacter WP_064229009
Marinobacter_sp._YWL01 WP_082813979
Marinobacter_nitratireducens KEF32007
Marinobacter_nitratireducens WP_081849471
Marinobacter_sp._C18 WP_075194766
Noviherbaspirillum_massiliense WP_019142760
Calditrichaeota_bacterium RMH84169
Parendozoicomonas_haliclonae WP_087112989
Hondaea_fermentalgiana GBG30877
Hondaea_fermentalgiana GBG30878
Aurantiochytrium_limacinum_ATCCMYA-1381 CAMPEP_0171498648
Aplanochytrium_sp._PBS07 CAMPEP_0184036110
Aplanochytrium_sp._PBS07 CAMPEP_0184066958
Aplanochytrium_sp._PBS07 CAMPEP_0184007812
Aplanochytrium_sp._PBS07 CAMPEP_0184045946
Aplanochytrium_stocchinoi_GSBS06 CAMPEP_0204828312
Aplanochytrium_stocchinoi_GSBS06 CAMPEP_0204868936
NonPhotosyntheticDiatom QUERY_NAA16P03020.m1
Dehydrogenase/reductase SDR family member
ここにテキストを入力
0.3

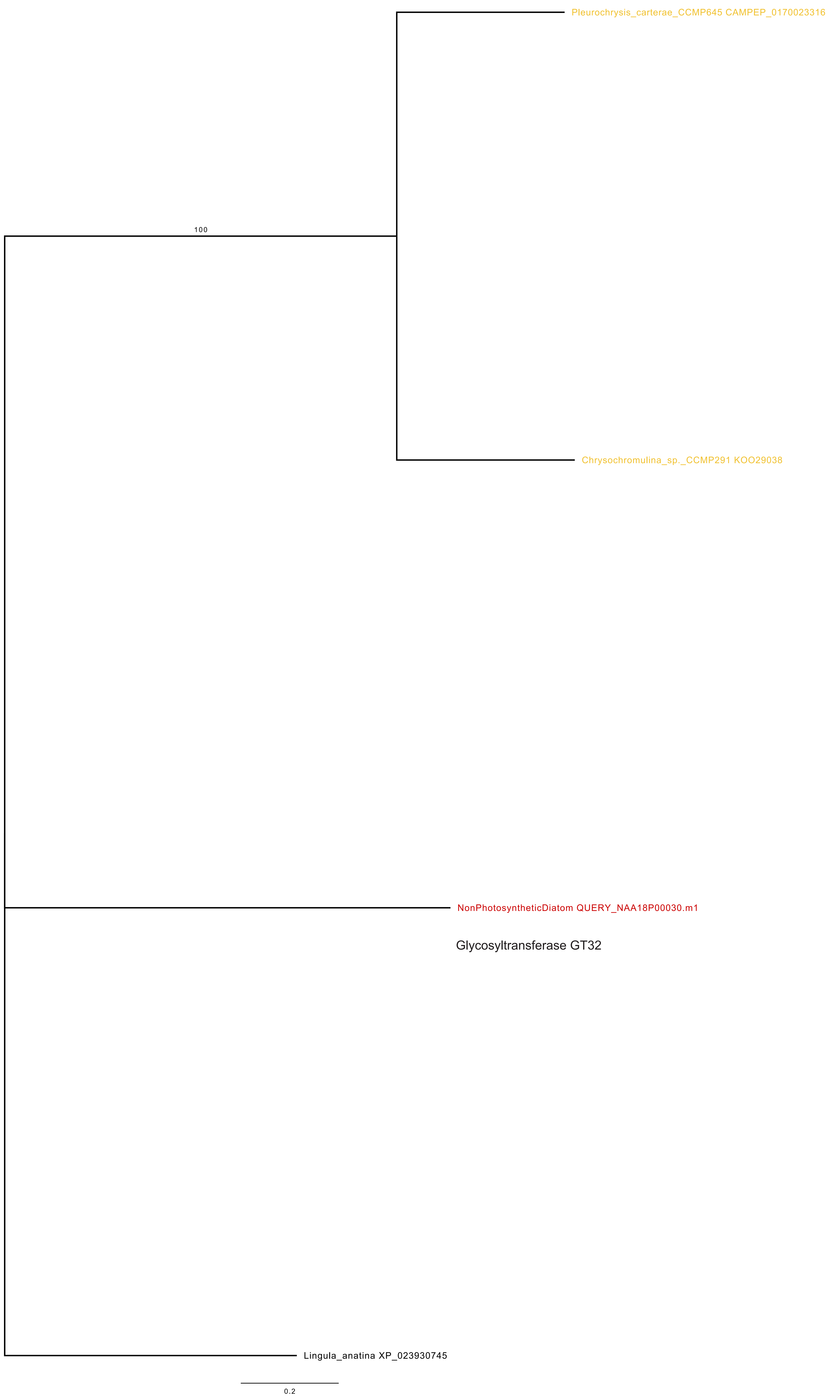
Pleurochrysis_carterae_CCMP645 CAMPEP_0170023316
100
Chrysochromulina_sp._CCMP291 KOO29038
NonPhotosyntheticDiatom QUERY_NAA18P00030.m1
Glycosyltransferase GT32
Lingula_anatina XP_023930745
0.2

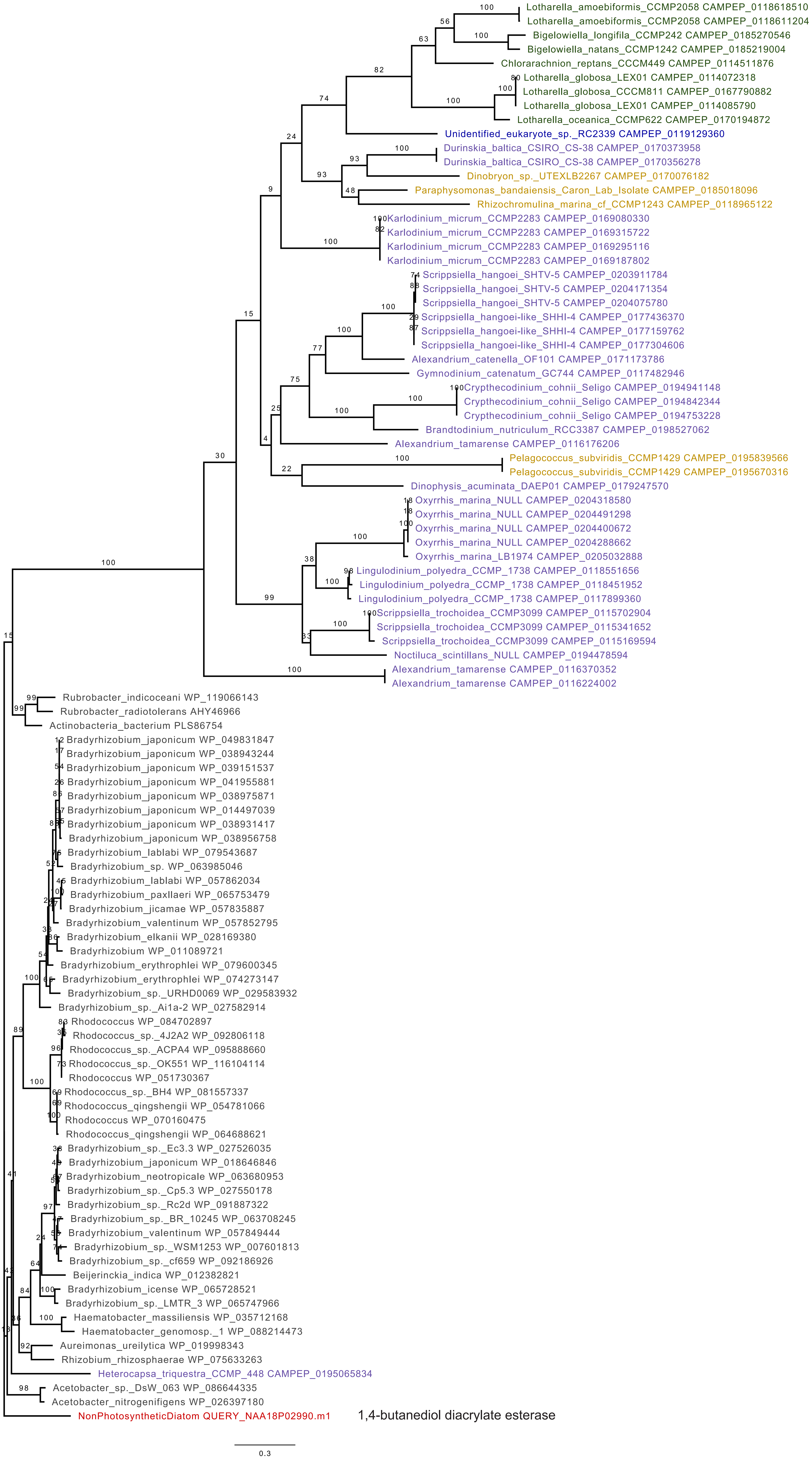
Lotharella_amoebiformis_CCMP2058 CAMPEP_0118618510
Lotharella_amoebiformis_CCMP2058 CAMPEP_0118611204
Bigelowiella_longifila_CCMP242 CAMPEP_0185270546
Bigelowiella_natans_CCMP1242 CAMPEP_0185219004
Chlorarachnion_reptans_CCCM449 CAMPEP_0114511876
Lotharella_globosa_LEX01 CAMPEP_0114072318
Lotharella_globosa_CCCM811 CAMPEP_0167790882
Lotharella_globosa_LEX01 CAMPEP_0114085790
Lotharella_oceanica_CCMP622 CAMPEP_0170194872
Unidentified_eukaryote_sp._RC2339 CAMPEP_0119129360
Durinskia_baltica_CSIRO_CS-38 CAMPEP_0170373958
Durinskia_baltica_CSIRO_CS-38 CAMPEP_0170356278
Dinobryon_sp._UTEXLB2267 CAMPEP_0170076182
Paraphysomonas_bandaiensis_Caron_Lab_Isolate CAMPEP_0185018096
Rhizochromulina_marina_cf_CCMP1243 CAMPEP_0118965122
Karlodinium_micrum_CCMP2283 CAMPEP_0169080330
Karlodinium_micrum_CCMP2283 CAMPEP_0169315722
Karlodinium_micrum_CCMP2283 CAMPEP_0169295116
Karlodinium_micrum_CCMP2283 CAMPEP_0169187802
Scrippsiella_hangoei_SHTV-5 CAMPEP_0203911784
Scrippsiella_hangoei_SHTV-5 CAMPEP_0204171354
Scrippsiella_hangoei_SHTV-5 CAMPEP_0204075780
Scrippsiella_hangoei-like_SHHI-4 CAMPEP_0177436370
Scrippsiella_hangoei-like_SHHI-4 CAMPEP_0177159762
Scrippsiella_hangoei-like_SHHI-4 CAMPEP_0177304606
Alexandrium_catenella_OF101 CAMPEP_0171173786
Gymnodinium_catenatum_GC744 CAMPEP_0117482946
Crypthecodinium_cohnii_Seligo CAMPEP_0194941148
Crypthecodinium_cohnii_Seligo CAMPEP_0194842344
Crypthecodinium_cohnii_Seligo CAMPEP_0194753228
Brandtodinium_nutriculum_RCC3387 CAMPEP_0198527062
Alexandrium_tamarense CAMPEP_0116176206
Pelagococcus_subviridis_CCMP1429 CAMPEP_0195839566
Pelagococcus_subviridis_CCMP1429 CAMPEP_0195670316
Dinophysis_acuminata_DAEP01 CAMPEP_0179247570
Oxyrrhis_marina_NULL CAMPEP_0204318580
Oxyrrhis_marina_NULL CAMPEP_0204491298
Oxyrrhis_marina_NULL CAMPEP_0204400672
Oxyrrhis_marina_NULL CAMPEP_0204288662
Oxyrrhis_marina_LB1974 CAMPEP_0205032888
Lingulodinium_polyedra_CCMP_1738 CAMPEP_0118551656
Lingulodinium_polyedra_CCMP_1738 CAMPEP_0118451952
Lingulodinium_polyedra_CCMP_1738 CAMPEP_0117899360
Scrippsiella_trochoidea_CCMP3099 CAMPEP_0115702904
Scrippsiella_trochoidea_CCMP3099 CAMPEP_0115341652
Scrippsiella_trochoidea_CCMP3099 CAMPEP_0115169594
Noctiluca_scintillans_NULL CAMPEP_0194478594
Alexandrium_tamarense CAMPEP_0116370352
Alexandrium_tamarense CAMPEP_0116224002
Rubrobacter_indicoceani WP_119066143
Rubrobacter_radiotolerans AHY46966
Actinobacteria_bacterium PLS86754
Bradyrhizobium_japonicum WP_049831847
Bradyrhizobium_japonicum WP_038943244
Bradyrhizobium_japonicum WP_039151537
Bradyrhizobium_japonicum WP_041955881
Bradyrhizobium_japonicum WP_038975871
Bradyrhizobium_japonicum WP_014497039
Bradyrhizobium_japonicum WP_038931417
Bradyrhizobium_japonicum WP_038956758
Bradyrhizobium_lablabi WP_079543687
Bradyrhizobium_sp. WP_063985046
Bradyrhizobium_lablabi WP_057862034
Bradyrhizobium_paxllaeri WP_065753479
Bradyrhizobium_jicamae WP_057835887
Bradyrhizobium_valentinum WP_057852795
Bradyrhizobium_elkanii WP_028169380
Bradyrhizobium WP_011089721
Bradyrhizobium_erythrophlei WP_079600345
Bradyrhizobium_erythrophlei WP_074273147
Bradyrhizobium_sp._URHD0069 WP_029583932
Bradyrhizobium_sp._Ai1a-2 WP_027582914
Rhodococcus WP_084702897
Rhodococcus_sp._4J2A2 WP_092806118
Rhodococcus_sp._ACPA4 WP_095888660
Rhodococcus_sp._OK551 WP_116104114
Rhodococcus WP_051730367
Rhodococcus_sp._BH4 WP_081557337
Rhodococcus_qingshengii WP_054781066
Rhodococcus WP_070160475
Rhodococcus_qingshengii WP_064688621
Bradyrhizobium_sp._Ec3.3 WP_027526035
Bradyrhizobium_japonicum WP_018646846
Bradyrhizobium_neotropicale WP_063680953
Bradyrhizobium_sp._Cp5.3 WP_027550178
Bradyrhizobium_sp._Rc2d WP_091887322
Bradyrhizobium_sp._BR_10245 WP_063708245
Bradyrhizobium_valentinum WP_057849444
Bradyrhizobium_sp._WSM1253 WP_007601813
Bradyrhizobium_sp._cf659 WP_092186926
Beijerinckia_indica WP_012382821
Bradyrhizobium_icense WP_065728521
Bradyrhizobium_sp._LMTR_3 WP_065747966
Haematobacter_massiliensis WP_035712168
Haematobacter_genomosp._1 WP_088214473
Aureimonas_ureilytica WP_019998343
Rhizobium_rhizosphaerae WP_075633263
Heterocapsa_triquestra_CCMP_448 CAMPEP_0195065834
Acetobacter_sp._DsW_063 WP_086644335
Acetobacter_nitrogenifigens WP_026397180
NonPhotosyntheticDiatom QUERY_NAA18P02990.m1
1,4-butanediol diacrylate esterase
0.3

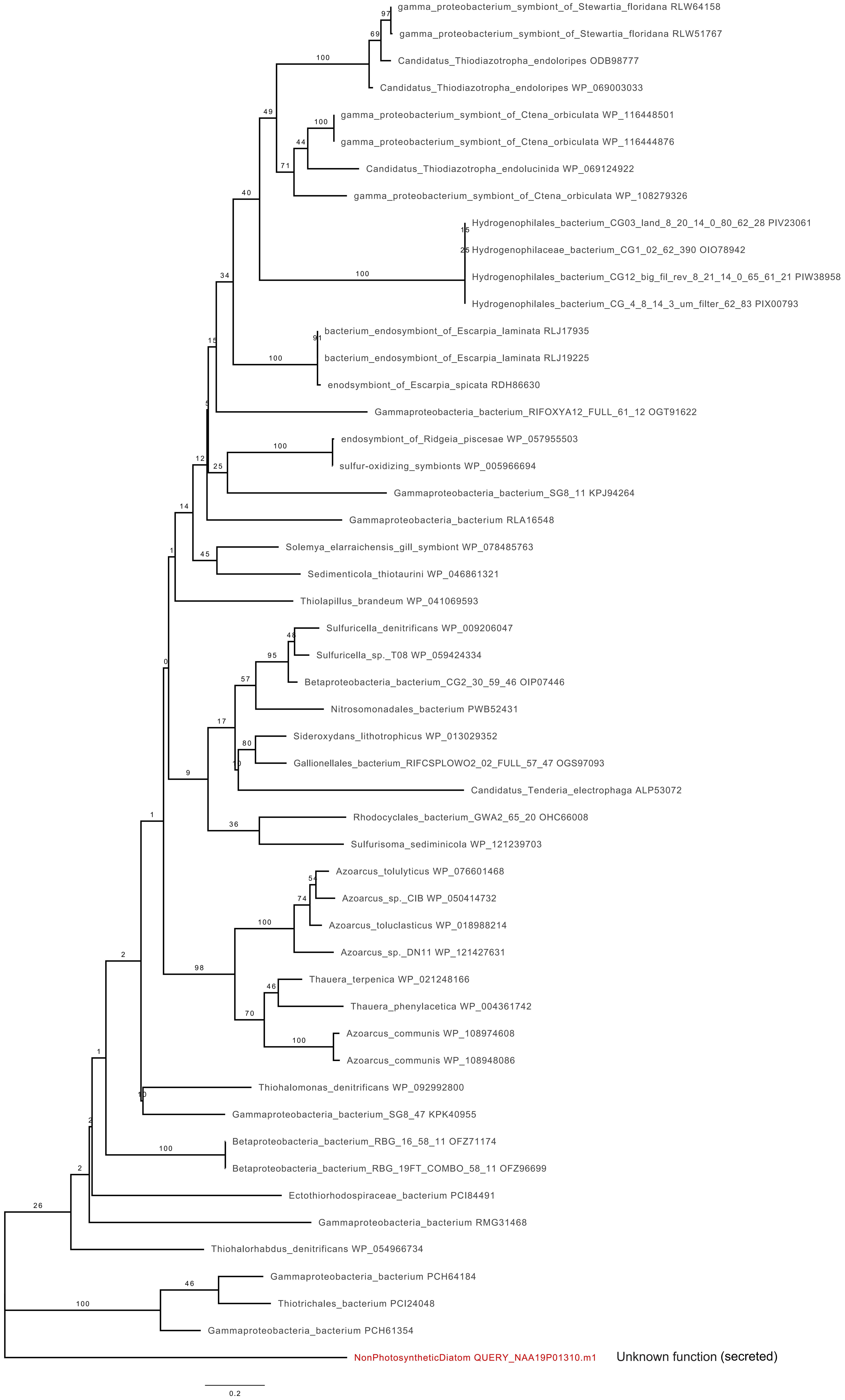
gamma_proteobacterium_symbiont_of_Stewartia_floridana RLW64158
gamma_proteobacterium_symbiont_of_Stewartia_floridana RLW51767
Candidatus_Thiodiazotropha_endoloripes ODB98777
Candidatus_Thiodiazotropha_endoloripes WP_069003033
gamma_proteobacterium_symbiont_of_Ctena_orbiculata WP_116448501
gamma_proteobacterium_symbiont_of_Ctena_orbiculata WP_116444876
Candidatus_Thiodiazotropha_endolucinida WP_069124922
gamma_proteobacterium_symbiont_of_Ctena_orbiculata WP_108279326
Hydrogenophilales_bacterium_CG03_land_8_20_14_0_80_62_28 PIV23061
Hydrogenophilaceae_bacterium_CG1_02_62_390 OIO78942
Hydrogenophilales_bacterium_CG12_big_fil_rev_8_21_14_0_65_61_21 PIW38958
Hydrogenophilales_bacterium_CG_4_8_14_3_um_filter_62_83 PIX00793
bacterium_endosymbiont_of_Escarpia_laminata RLJ17935
bacterium_endosymbiont_of_Escarpia_laminata RLJ19225
enodsymbiont_of_Escarpia_spicata RDH86630
Gammaproteobacteria_bacterium_RIFOXYA12_FULL_61_12 OGT91622
endosymbiont_of_Ridgeia_piscesae WP_057955503
sulfur-oxidizing_symbionts WP_005966694
Gammaproteobacteria_bacterium_SG8_11 KPJ94264
Gammaproteobacteria_bacterium RLA16548
Solemya_elarraichensis_gill_symbiont WP_078485763
Sedimenticola_thiotaurini WP_046861321
Thiolapillus_brandeum WP_041069593
Sulfuricella_denitrificans WP_009206047
Sulfuricella_sp._T08 WP_059424334
Betaproteobacteria_bacterium_CG2_30_59_46 OIP07446
Nitrosomonadales_bacterium PWB52431
Sideroxydans_lithotrophicus WP_013029352
Gallionellales_bacterium_RIFCSPLOWO2_02_FULL_57_47 OGS97093
Candidatus_Tenderia_electrophaga ALP53072
Rhodocyclales_bacterium_GWA2_65_20 OHC66008
Sulfurisoma_sediminicola WP_121239703
Azoarcus_tolulyticus WP_076601468
Azoarcus_sp._CIB WP_050414732
Azoarcus_toluclasticus WP_018988214
Azoarcus_sp._DN11 WP_121427631
Thauera_terpenica WP_021248166
Thauera_phenylacetica WP_004361742
Azoarcus_communis WP_108974608
Azoarcus_communis WP_108948086
Thiohalomonas_denitrificans WP_092992800
Gammaproteobacteria_bacterium_SG8_47 KPK40955
Betaproteobacteria_bacterium_RBG_16_58_11 OFZ71174
Betaproteobacteria_bacterium_RBG_19FT_COMBO_58_11 OFZ96699
Ectothiorhodospiraceae_bacterium PCI84491
Gammaproteobacteria_bacterium RMG31468
Thiohalorhabdus_denitrificans WP_054966734
Gammaproteobacteria_bacterium PCH64184
Thiotrichales_bacterium PCI24048
Gammaproteobacteria_bacterium PCH61354
NonPhotosyntheticDiatom QUERY_NAA19P01310.m1
Unknown function (secreted)
0.2

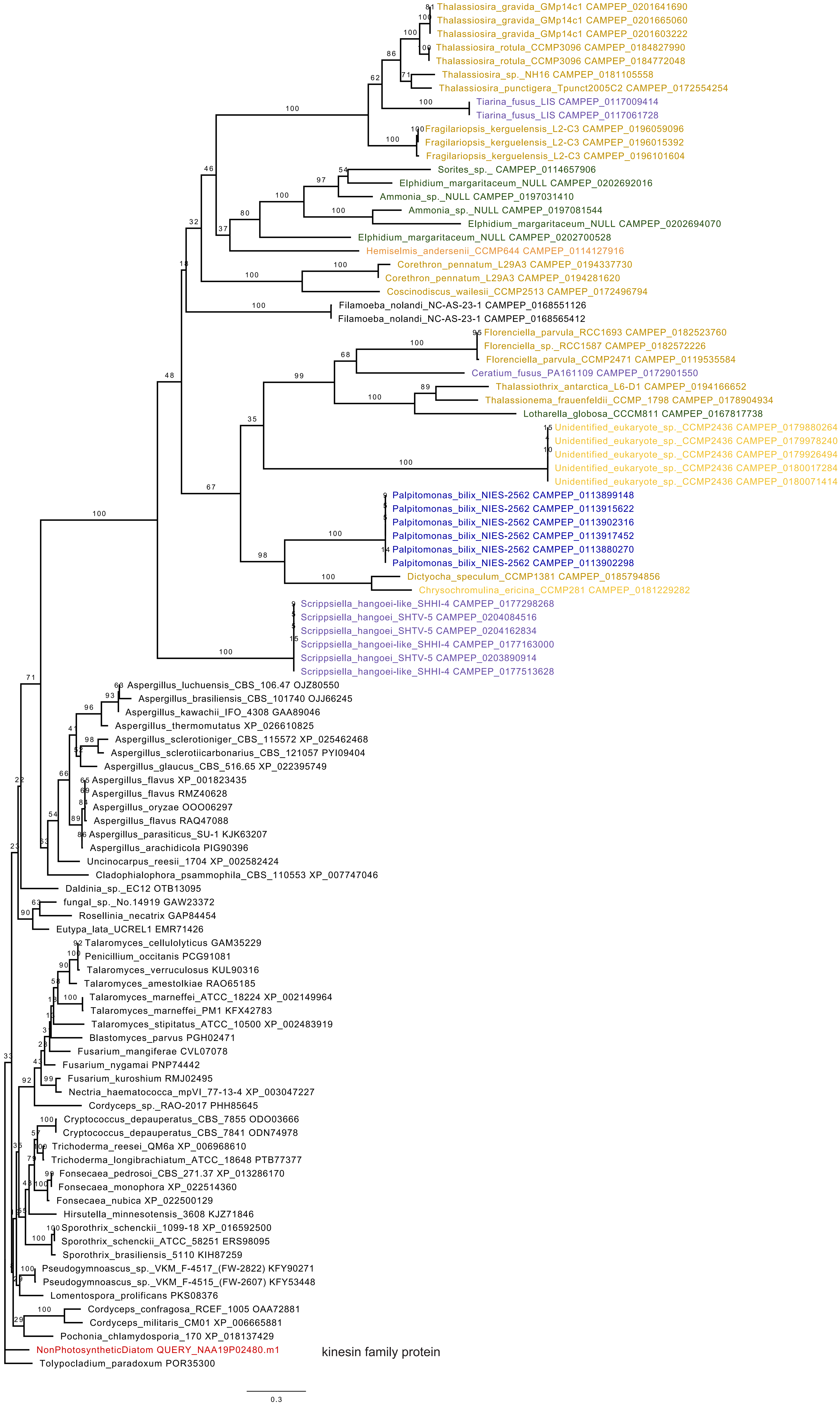
Thalassiosira_gravida_GMp14c1 CAMPEP_0201641690
Thalassiosira_gravida_GMp14c1 CAMPEP_0201665060
Thalassiosira_gravida_GMp14c1 CAMPEP_0201603222
Thalassiosira_rotula_CCMP3096 CAMPEP_0184827990
Thalassiosira_rotula_CCMP3096 CAMPEP_0184772048
Thalassiosira_sp._NH16 CAMPEP_0181105558
Thalassiosira_punctigera_Tpunct2005C2 CAMPEP_0172554254
Tiarina_fusus_LIS CAMPEP_0117009414
Tiarina_fusus_LIS CAMPEP_0117061728
Fragilariopsis_kerguelensis_L2-C3 CAMPEP_0196059096
Fragilariopsis_kerguelensis_L2-C3 CAMPEP_0196015392
Fragilariopsis_kerguelensis_L2-C3 CAMPEP_0196101604
Sorites_sp._ CAMPEP_0114657906
Elphidium_margaritaceum_NULL CAMPEP_0202692016
Ammonia_sp._NULL CAMPEP_0197031410
Ammonia_sp._NULL CAMPEP_0197081544
Elphidium_margaritaceum_NULL CAMPEP_0202694070
Elphidium_margaritaceum_NULL CAMPEP_0202700528
Hemiselmis_andersenii_CCMP644 CAMPEP_0114127916
Corethron_pennatum_L29A3 CAMPEP_0194337730
Corethron_pennatum_L29A3 CAMPEP_0194281620
Coscinodiscus_wailesii_CCMP2513 CAMPEP_0172496794
Filamoeba_nolandi_NC-AS-23-1 CAMPEP_0168551126
Filamoeba_nolandi_NC-AS-23-1 CAMPEP_0168565412
Florenciella_parvula_RCC1693 CAMPEP_0182523760
Florenciella_sp._RCC1587 CAMPEP_0182572226
Florenciella_parvula_CCMP2471 CAMPEP_0119535584
Ceratium_fusus_PA161109 CAMPEP_0172901550
Thalassiothrix_antarctica_L6-D1 CAMPEP_0194166652
Thalassionema_frauenfeldii_CCMP_1798 CAMPEP_0178904934
Lotharella_globosa_CCCM811 CAMPEP_0167817738
Unidentified_eukaryote_sp._CCMP2436 CAMPEP_0179880264
Unidentified_eukaryote_sp._CCMP2436 CAMPEP_0179978240
Unidentified_eukaryote_sp._CCMP2436 CAMPEP_0179926494
Unidentified_eukaryote_sp._CCMP2436 CAMPEP_0180017284
Unidentified_eukaryote_sp._CCMP2436 CAMPEP_0180071414
Palpitomonas_bilix_NIES-2562 CAMPEP_0113899148
Palpitomonas_bilix_NIES-2562 CAMPEP_0113915622
Palpitomonas_bilix_NIES-2562 CAMPEP_0113902316
Palpitomonas_bilix_NIES-2562 CAMPEP_0113917452
Palpitomonas_bilix_NIES-2562 CAMPEP_0113880270
Palpitomonas_bilix_NIES-2562 CAMPEP_0113902298
Dictyocha_speculum_CCMP1381 CAMPEP_0185794856
Chrysochromulina_ericina_CCMP281 CAMPEP_0181229282
Scrippsiella_hangoei-like_SHHI-4 CAMPEP_0177298268
Scrippsiella_hangoei_SHTV-5 CAMPEP_0204084516
Scrippsiella_hangoei_SHTV-5 CAMPEP_0204162834
Scrippsiella_hangoei-like_SHHI-4 CAMPEP_0177163000
Scrippsiella_hangoei_SHTV-5 CAMPEP_0203890914
Scrippsiella_hangoei-like_SHHI-4 CAMPEP_0177513628
Aspergillus_luchuensis_CBS_106.47 OJZ80550
Aspergillus_brasiliensis_CBS_101740 OJJ66245
Aspergillus_kawachii_IFO_4308 GAA89046
Aspergillus_thermomutatus XP_026610825
Aspergillus_sclerotioniger_CBS_115572 XP_025462468
Aspergillus_sclerotiicarbonarius_CBS_121057 PYI09404
Aspergillus_glaucus_CBS_516.65 XP_022395749
Aspergillus_flavus XP_001823435
Aspergillus_flavus RMZ40628
Aspergillus_oryzae OOO06297
Aspergillus_flavus RAQ47088
Aspergillus_parasiticus_SU-1 KJK63207
Aspergillus_arachidicola PIG90396
Uncinocarpus_reesii_1704 XP_002582424
Cladophialophora_psammophila_CBS_110553 XP_007747046
Daldinia_sp._EC12 OTB13095
fungal_sp._No.14919 GAW23372
Rosellinia_necatrix GAP84454
Eutypa_lata_UCREL1 EMR71426
Talaromyces_cellulolyticus GAM35229
Penicillium_occitanis PCG91081
Talaromyces_verruculosus KUL90316
Talaromyces_amestolkiae RAO65185
Talaromyces_marneffei_ATCC_18224 XP_002149964
Talaromyces_marneffei_PM1 KFX42783
Talaromyces_stipitatus_ATCC_10500 XP_002483919
Blastomyces_parvus PGH02471
Fusarium_mangiferae CVL07078
Fusarium_nygamai PNP74442
Fusarium_kuroshium RMJ02495
Nectria_haematococca_mpVI_77-13-4 XP_003047227
Cordyceps_sp._RAO-2017 PHH85645
Cryptococcus_depauperatus_CBS_7855 ODO03666
Cryptococcus_depauperatus_CBS_7841 ODN74978
Trichoderma_reesei_QM6a XP_006968610
Trichoderma_longibrachiatum_ATCC_18648 PTB77377
Fonsecaea_pedrosoi_CBS_271.37 XP_013286170
Fonsecaea_monophora XP_022514360
Fonsecaea_nubica XP_022500129
Hirsutella_minnesotensis_3608 KJZ71846
Sporothrix_schenckii_1099-18 XP_016592500
Sporothrix_schenckii_ATCC_58251 ERS98095
Sporothrix_brasiliensis_5110 KIH87259
Pseudogymnoascus_sp._VKM_F-4517_(FW-2822) KFY90271
Pseudogymnoascus_sp._VKM_F-4515_(FW-2607) KFY53448
Lomentospora_prolificans PKS08376
Cordyceps_confragosa_RCEF_1005 OAA72881
Cordyceps_militaris_CM01 XP_006665881
Pochonia_chlamydosporia_170 XP_018137429
NonPhotosyntheticDiatom QUERY_NAA19P02480.m1
Tolypocladium_paradoxum POR35300
kinesin family protein
0.3

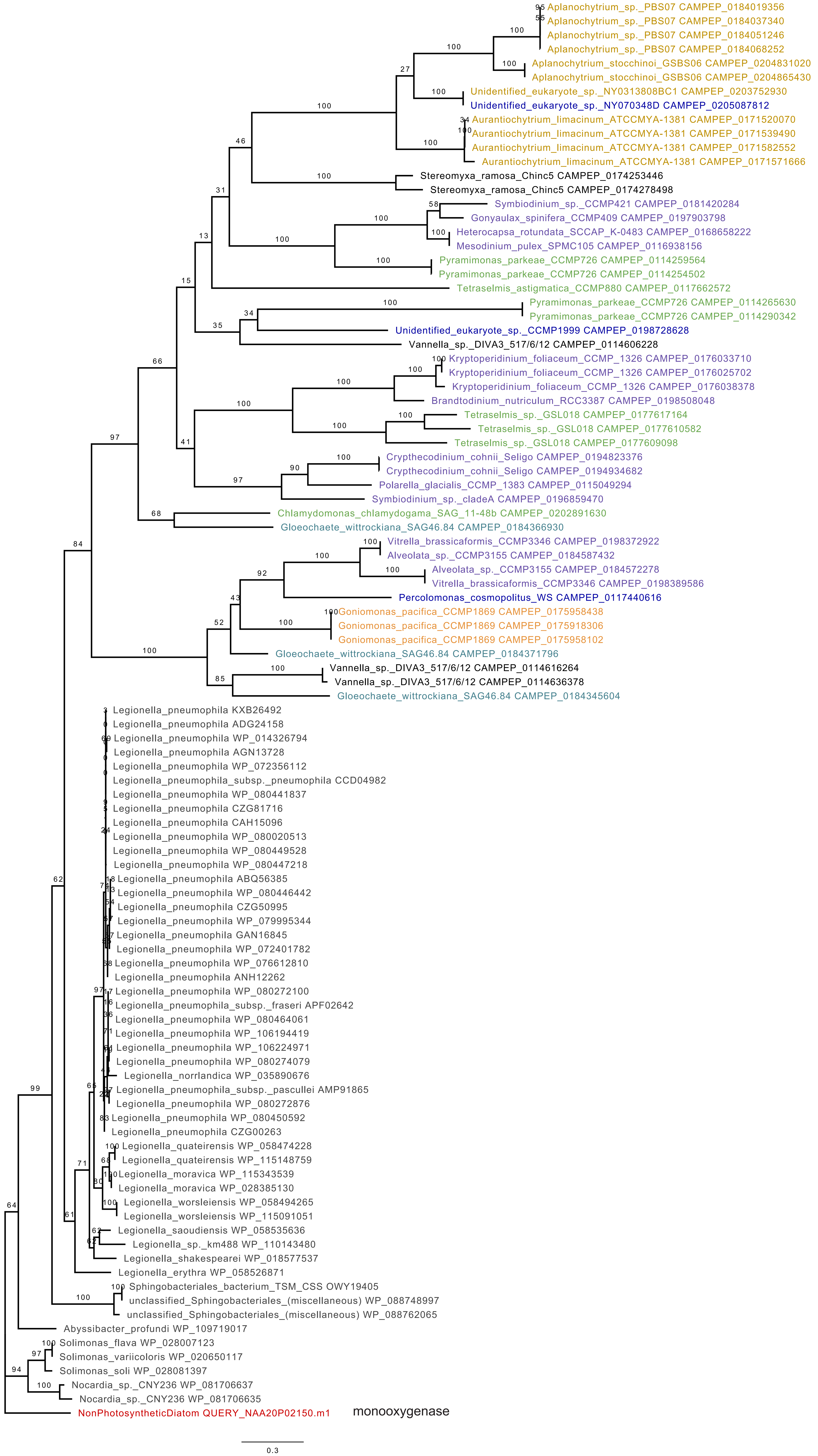

Aplanochytrium_sp._PBS07 CAMPEP_0184019356
Aplanochytrium_sp._PBS07 CAMPEP_0184037340
Aplanochytrium_sp._PBS07 CAMPEP_0184051246
Aplanochytrium_sp._PBS07 CAMPEP_0184068252
Aplanochytrium_stocchinoi_GSBS06 CAMPEP_0204831020
Aplanochytrium_stocchinoi_GSBS06 CAMPEP_0204865430
Unidentified_eukaryote_sp._NY0313808BC1 CAMPEP_0203752930
Unidentified_eukaryote_sp._NY070348D CAMPEP_0205087812
Aurantiochytrium_limacinum_ATCCMYA-1381 CAMPEP_0171520070
Aurantiochytrium_limacinum_ATCCMYA-1381 CAMPEP_0171539490
Aurantiochytrium_limacinum_ATCCMYA-1381 CAMPEP_0171582552
Aurantiochytrium_limacinum_ATCCMYA-1381 CAMPEP_0171571666
Stereomyxa_ramosa_Chinc5 CAMPEP_0174253446
Stereomyxa_ramosa_Chinc5 CAMPEP_0174278498
Symbiodinium_sp._CCMP421 CAMPEP_0181420284
Gonyaulax_spinifera_CCMP409 CAMPEP_0197903798
Heterocapsa_rotundata_SCCAP_K-0483 CAMPEP_0168658222
Mesodinium_pulex_SPMC105 CAMPEP_0116938156
Pyramimonas_parkeae_CCMP726 CAMPEP_0114259564
Pyramimonas_parkeae_CCMP726 CAMPEP_0114254502
Tetraselmis_astigmatica_CCMP880 CAMPEP_0117662572
Pyramimonas_parkeae_CCMP726 CAMPEP_0114265630
Pyramimonas_parkeae_CCMP726 CAMPEP_0114290342
Unidentified_eukaryote_sp._CCMP1999 CAMPEP_0198728628
Vannella_sp._DIVA3_517/6/12 CAMPEP_0114606228
Kryptoperidinium_foliaceum_CCMP_1326 CAMPEP_0176033710
Kryptoperidinium_foliaceum_CCMP_1326 CAMPEP_0176025702
Kryptoperidinium_foliaceum_CCMP_1326 CAMPEP_0176038378
Brandtodinium_nutriculum_RCC3387 CAMPEP_0198508048
Tetraselmis_sp._GSL018 CAMPEP_0177617164
Tetraselmis_sp._GSL018 CAMPEP_0177610582
Tetraselmis_sp._GSL018 CAMPEP_0177609098
Crypthecodinium_cohnii_Seligo CAMPEP_0194823376
Crypthecodinium_cohnii_Seligo CAMPEP_0194934682
Polarella_glacialis_CCMP_1383 CAMPEP_0115049294
Symbiodinium_sp._cladeA CAMPEP_0196859470
Chlamydomonas_chlamydogama_SAG_11-48b CAMPEP_0202891630
Gloeochaete_wittrockiana_SAG46.84 CAMPEP_0184366930
Vitrella_brassicaformis_CCMP3346 CAMPEP_0198372922
Alveolata_sp._CCMP3155 CAMPEP_0184587432
Alveolata_sp._CCMP3155 CAMPEP_0184572278
Vitrella_brassicaformis_CCMP3346 CAMPEP_0198389586
Percolomonas_cosmopolitus_WS CAMPEP_0117440616
Goniomonas_pacifica_CCMP1869 CAMPEP_0175958438
Goniomonas_pacifica_CCMP1869 CAMPEP_0175918306
Goniomonas_pacifica_CCMP1869 CAMPEP_0175958102
Gloeochaete_wittrockiana_SAG46.84 CAMPEP_0184371796
Vannella_sp._DIVA3_517/6/12 CAMPEP_0114616264
Vannella_sp._DIVA3_517/6/12 CAMPEP_0114636378
Gloeochaete_wittrockiana_SAG46.84 CAMPEP_0184345604
Legionella_pneumophila KXB26492
Legionella_pneumophila ADG24158
Legionella_pneumophila WP_014326794
Legionella_pneumophila AGN13728
Legionella_pneumophila WP_072356112
Legionella_pneumophila_subsp._pneumophila CCD04982
Legionella_pneumophila WP_080441837
Legionella_pneumophila CZG81716
Legionella_pneumophila CAH15096
Legionella_pneumophila WP_080020513
Legionella_pneumophila WP_080449528
Legionella_pneumophila WP_080447218
Legionella_pneumophila ABQ56385
Legionella_pneumophila WP_080446442
Legionella_pneumophila CZG50995
Legionella_pneumophila WP_079995344
Legionella_pneumophila GAN16845
Legionella_pneumophila WP_072401782
Legionella_pneumophila WP_076612810
Legionella_pneumophila ANH12262
Legionella_pneumophila WP_080272100
Legionella_pneumophila_subsp._fraseri APF02642
Legionella_pneumophila WP_080464061
Legionella_pneumophila WP_106194419
Legionella_pneumophila WP_106224971
Legionella_pneumophila WP_080274079
Legionella_norrlandica WP_035890676
Legionella_pneumophila_subsp._pascullei AMP91865
Legionella_pneumophila WP_080272876
Legionella_pneumophila WP_080450592
Legionella_pneumophila CZG00263
Legionella_quateirensis WP_058474228
Legionella_quateirensis WP_115148759
Legionella_moravica WP_115343539
Legionella_moravica WP_028385130
Legionella_worsleiensis WP_058494265
Legionella_worsleiensis WP_115091051
Legionella_saoudiensis WP_058535636
Legionella_sp._km488 WP_110143480
Legionella_shakespearei WP_018577537
Legionella_erythra WP_058526871
Sphingobacteriales_bacterium_TSM_CSS OWY19405
unclassified_Sphingobacteriales_(miscellaneous) WP_088748997
unclassified_Sphingobacteriales_(miscellaneous) WP_088762065
Abyssibacter_profundi WP_109719017
Solimonas_flava WP_028007123
Solimonas_variicoloris WP_020650117
Solimonas_soli WP_028081397
Nocardia_sp._CNY236 WP_081706637
Nocardia_sp._CNY236 WP_081706635
NonPhotosyntheticDiatom QUERY_NAA20P02150.m1
monooxygenase
0.3

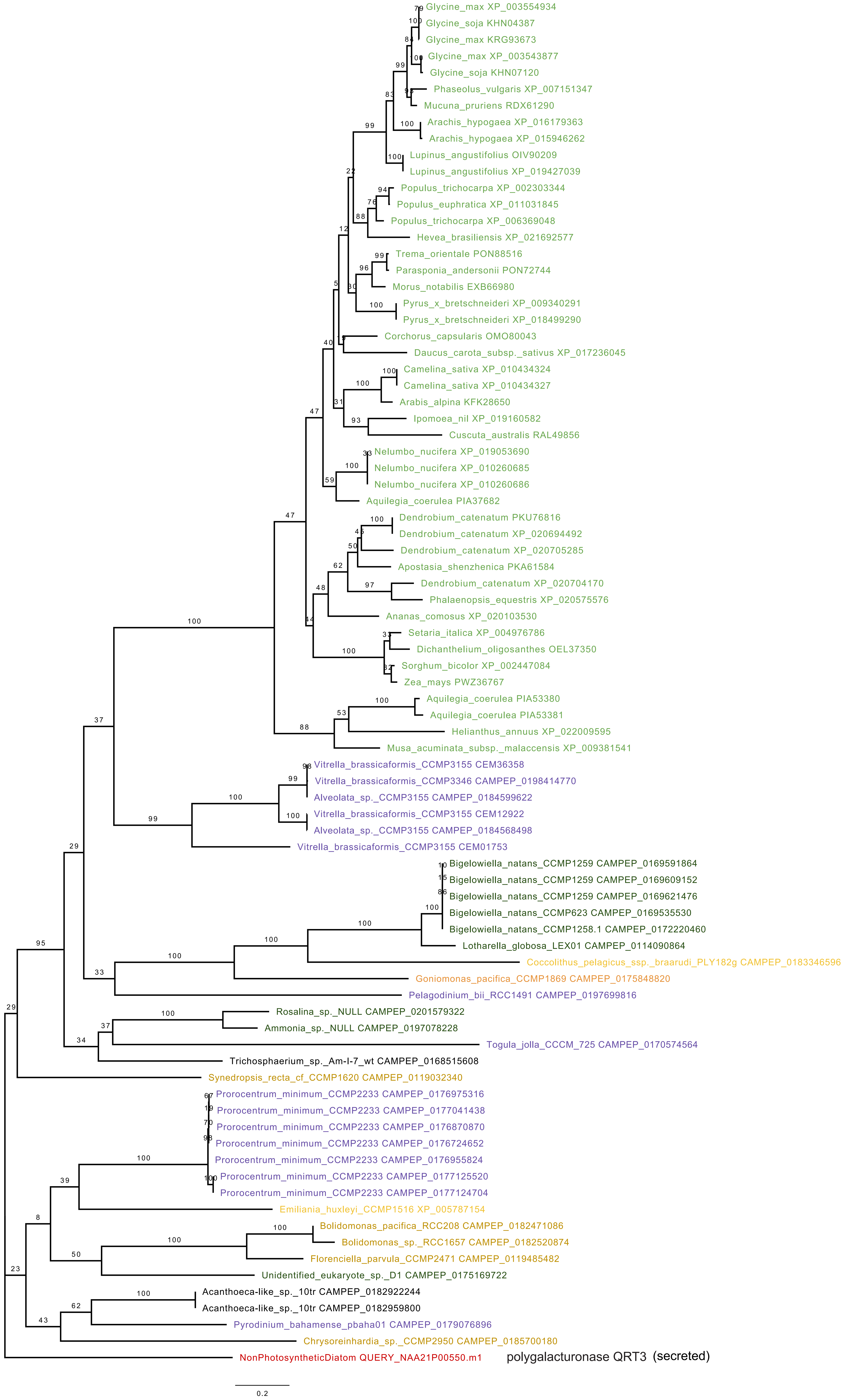
Glycine_max XP_003554934
Glycine_soja KHN04387
Glycine_max KRG93673
Glycine_max XP_003543877
Glycine_soja KHN07120
Phaseolus_vulgaris XP_007151347
Mucuna_pruriens RDX61290
Arachis_hypogaea XP_016179363
Arachis_hypogaea XP_015946262
Lupinus_angustifolius OIV90209
Lupinus_angustifolius XP_019427039
Populus_trichocarpa XP_002303344
Populus_euphratica XP_011031845
Populus_trichocarpa XP_006369048
Hevea_brasiliensis XP_021692577
Trema_orientale PON88516
Parasponia_andersonii PON72744
Morus_notabilis EXB66980
Pyrus_x_bretschneideri XP_009340291
Pyrus_x_bretschneideri XP_018499290
Corchorus_capsularis OMO80043
Daucus_carota_subsp._sativus XP_017236045
Camelina_sativa XP_010434324
Camelina_sativa XP_010434327
Arabis_alpina KFK28650
Ipomoea_nil XP_019160582
Cuscuta_australis RAL49856
Nelumbo_nucifera XP_019053690
Nelumbo_nucifera XP_010260685
Nelumbo_nucifera XP_010260686
Aquilegia_coerulea PIA37682
Dendrobium_catenatum PKU76816
Dendrobium_catenatum XP_020694492
Dendrobium_catenatum XP_020705285
Apostasia_shenzhenica PKA61584
Dendrobium_catenatum XP_020704170
Phalaenopsis_equestris XP_020575576
Ananas_comosus XP_020103530
Setaria_italica XP_004976786
Dichanthelium_oligosanthes OEL37350
Sorghum_bicolor XP_002447084
Zea_mays PWZ36767
Aquilegia_coerulea PIA53380
Aquilegia_coerulea PIA53381
Helianthus_annuus XP_022009595
Musa_acuminata_subsp._malaccensis XP_009381541
Vitrella_brassicaformis_CCMP3155 CEM36358
Vitrella_brassicaformis_CCMP3346 CAMPEP_0198414770
Alveolata_sp._CCMP3155 CAMPEP_0184599622
Vitrella_brassicaformis_CCMP3155 CEM12922
Alveolata_sp._CCMP3155 CAMPEP_0184568498
Vitrella_brassicaformis_CCMP3155 CEM01753
Bigelowiella_natans_CCMP1259 CAMPEP_0169591864
Bigelowiella_natans_CCMP1259 CAMPEP_0169609152
Bigelowiella_natans_CCMP1259 CAMPEP_0169621476
Bigelowiella_natans_CCMP623 CAMPEP_0169535530
Bigelowiella_natans_CCMP1258.1 CAMPEP_0172220460
Lotharella_globosa_LEX01 CAMPEP_0114090864
Coccolithus_pelagicus_ssp._braarudi_PLY182g CAMPEP_0183346596
Goniomonas_pacifica_CCMP1869 CAMPEP_0175848820
Pelagodinium_bii_RCC1491 CAMPEP_0197699816
Rosalina_sp._NULL CAMPEP_0201579322
Ammonia_sp._NULL CAMPEP_0197078228
Togula_jolla_CCCM_725 CAMPEP_0170574564
Trichosphaerium_sp._Am-I-7_wt CAMPEP_0168515608
Synedropsis_recta_cf_CCMP1620 CAMPEP_0119032340
Prorocentrum_minimum_CCMP2233 CAMPEP_0176975316
Prorocentrum_minimum_CCMP2233 CAMPEP_0177041438
Prorocentrum_minimum_CCMP2233 CAMPEP_0176870870
Prorocentrum_minimum_CCMP2233 CAMPEP_0176724652
Prorocentrum_minimum_CCMP2233 CAMPEP_0176955824
Prorocentrum_minimum_CCMP2233 CAMPEP_0177125520
Prorocentrum_minimum_CCMP2233 CAMPEP_0177124704
Emiliania_huxleyi_CCMP1516 XP_005787154
Bolidomonas_pacifica_RCC208 CAMPEP_0182471086
Bolidomonas_sp._RCC1657 CAMPEP_0182520874
Florenciella_parvula_CCMP2471 CAMPEP_0119485482
Unidentified_eukaryote_sp._D1 CAMPEP_0175169722
Acanthoeca-like_sp._10tr CAMPEP_0182922244
Acanthoeca-like_sp._10tr CAMPEP_0182959800
Pyrodinium_bahamense_pbaha01 CAMPEP_0179076896
Chrysoreinhardia_sp._CCMP2950 CAMPEP_0185700180
NonPhotosyntheticDiatom QUERY_NAA21P00550.m1
polygalacturonase QRT3 (secreted)
0.2

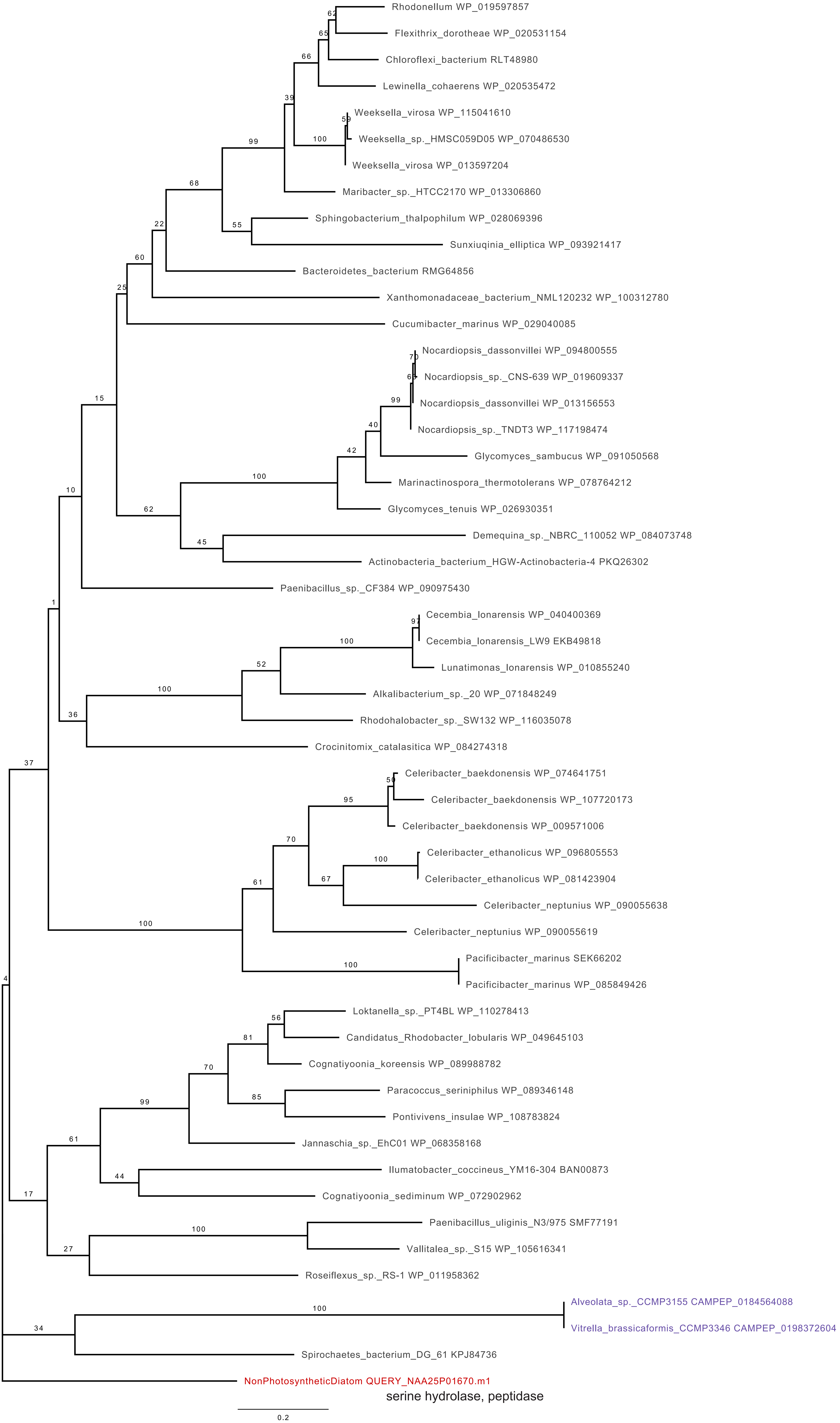

serine hydrolase, peptidase

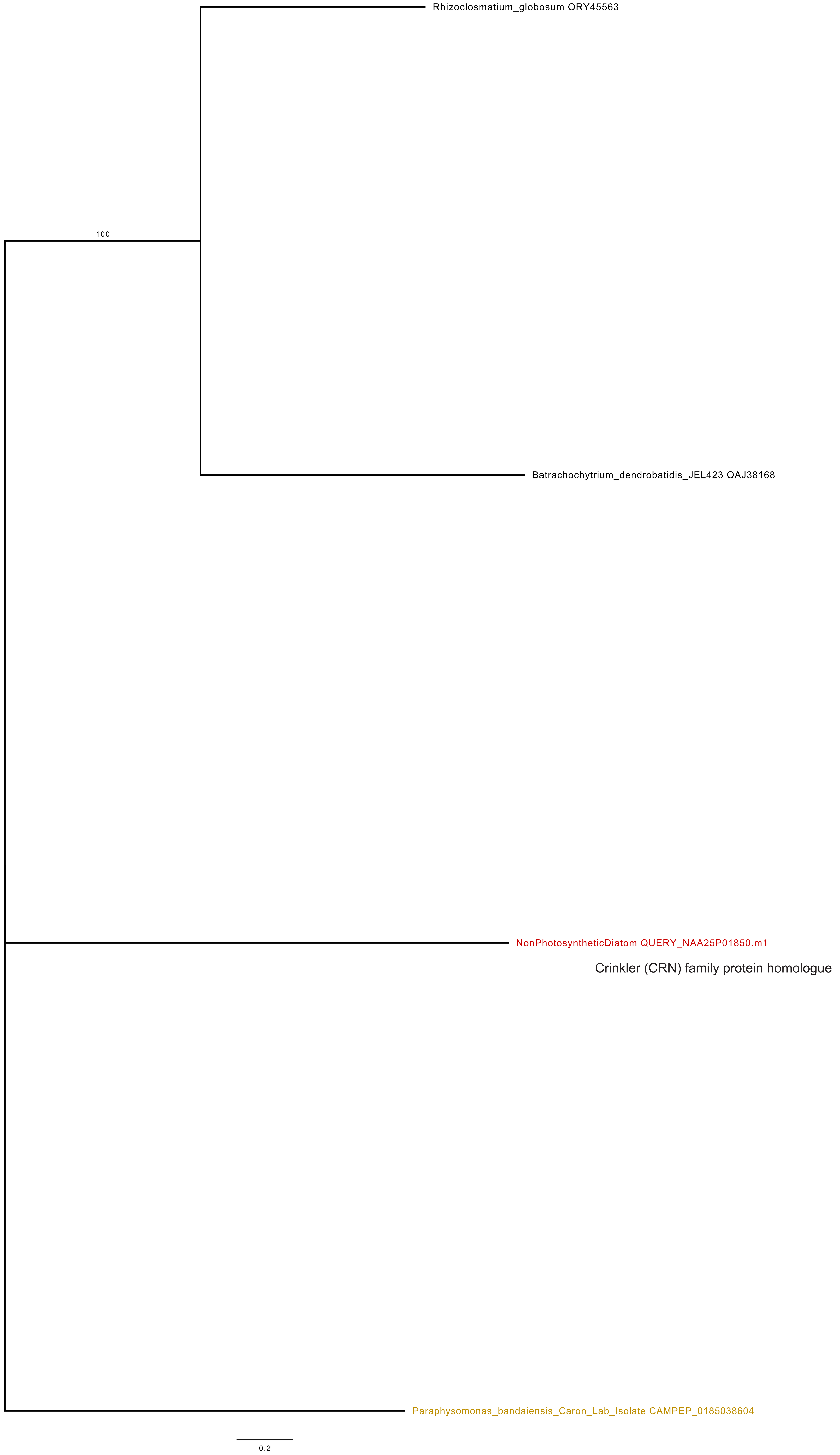
Rhizoclosmatium_globosum ORY45563
100
Batrachochytrium_dendrobatidis_JEL423 OAJ38168
NonPhotosyntheticDiatom QUERY_NAA25P01850.m1
Crinkler (CRN) family protein homologue
Paraphysomonas_bandaiensis_Caron_Lab_Isolate CAMPEP_0185038604
0.2

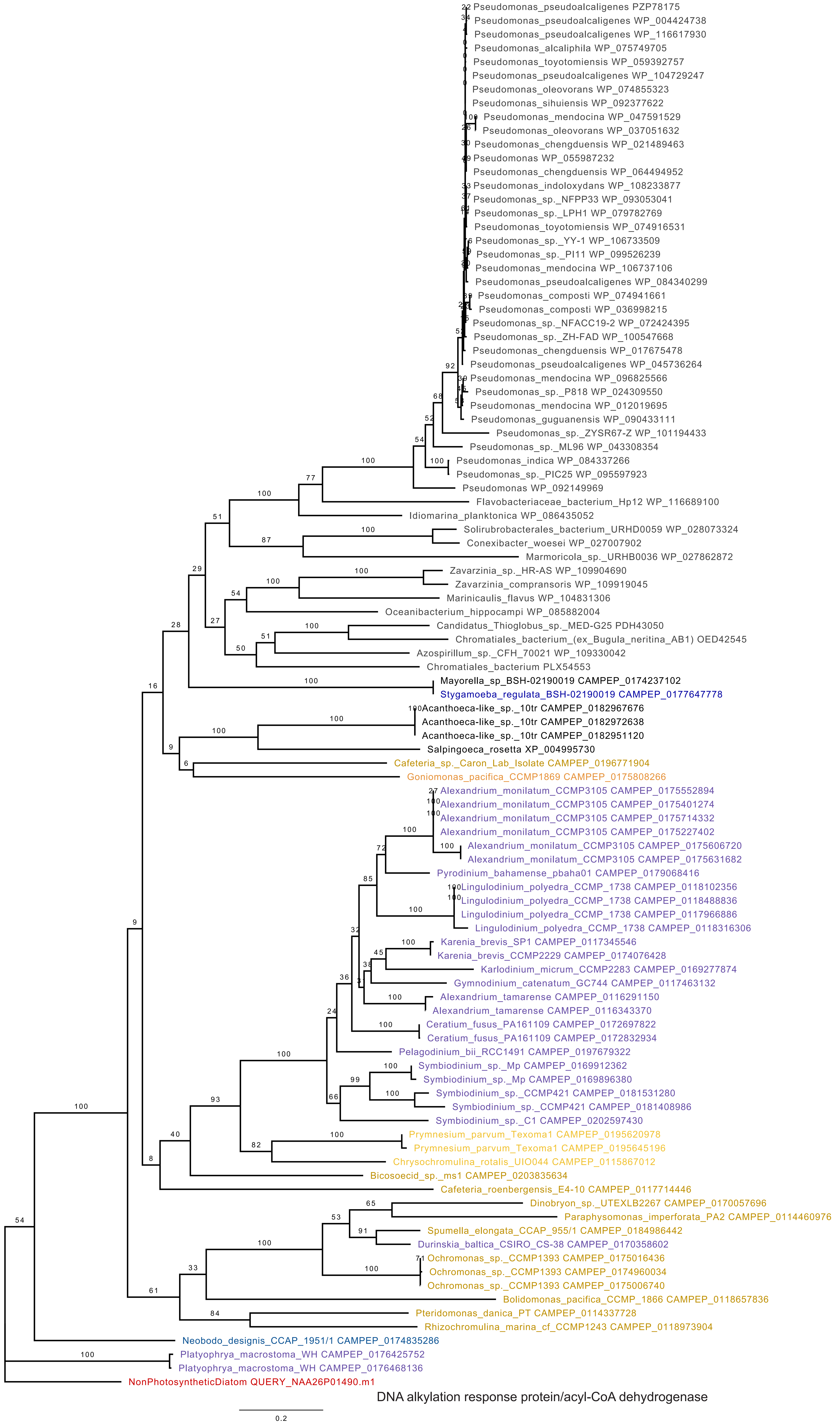

Pseudomonas_pseudoalcaligenes PZP78175
Pseudomonas_pseudoalcaligenes WP_004424738
Pseudomonas_pseudoalcaligenes WP_116617930
Pseudomonas_alcaliphila WP_075749705
Pseudomonas_toyotomiensis WP_059392757
Pseudomonas_pseudoalcaligenes WP_104729247
Pseudomonas_oleovorans WP_074855323
Pseudomonas_sihuiensis WP_092377622
Pseudomonas_mendocina WP_047591529
Pseudomonas_oleovorans WP_037051632
Pseudomonas_chengduensis WP_021489463
Pseudomonas WP_055987232
Pseudomonas_chengduensis WP_064494952
Pseudomonas_indoloxydans WP_108233877
Pseudomonas_sp._NFPP33 WP_093053041
Pseudomonas_sp._LPH1 WP_079782769
Pseudomonas_toyotomiensis WP_074916531
Pseudomonas_sp._YY-1 WP_106733509
Pseudomonas_sp._PI11 WP_099526239
Pseudomonas_mendocina WP_106737106
Pseudomonas_pseudoalcaligenes WP_084340299
Pseudomonas_composti WP_074941661
Pseudomonas_composti WP_036998215
Pseudomonas_sp._NFACC19-2 WP_072424395
Pseudomonas_sp._ZH-FAD WP_100547668
Pseudomonas_chengduensis WP_017675478
Pseudomonas_pseudoalcaligenes WP_045736264
Pseudomonas_mendocina WP_096825566
Pseudomonas_sp._P818 WP_024309550
Pseudomonas_mendocina WP_012019695
Pseudomonas_guguanensis WP_090433111
Pseudomonas_sp._ZYSR67-Z WP_101194433
Pseudomonas_sp._ML96 WP_043308354
Pseudomonas_indica WP_084337266
Pseudomonas_sp._PIC25 WP_095597923
Pseudomonas WP_092149969
Flavobacteriaceae_bacterium_Hp12 WP_116689100
Idiomarina_planktonica WP_086435052
Solirubrobacterales_bacterium_URHD0059 WP_028073324
Conexibacter_woesei WP_027007902
Marmoricola_sp._URHB0036 WP_027862872
Zavarzinia_sp._HR-AS WP_109904690
Zavarzinia_compransoris WP_109919045
Marinicaulis_flavus WP_104831306
Oceanibacterium_hippocampi WP_085882004
Candidatus_Thioglobus_sp._MED-G25 PDH43050
Chromatiales_bacterium_(ex_Bugula_neritina_AB1) OED42545
Azospirillum_sp._CFH_70021 WP_109330042
Chromatiales_bacterium PLX54553
Mayorella_sp_BSH-02190019 CAMPEP_0174237102
Stygamoeba_regulata_BSH-02190019 CAMPEP_0177647778
Acanthoeca-like_sp._10tr CAMPEP_0182967676
Acanthoeca-like_sp._10tr CAMPEP_0182972638
Acanthoeca-like_sp._10tr CAMPEP_0182951120
Salpingoeca_rosetta XP_004995730
Cafeteria_sp._Caron_Lab_Isolate CAMPEP_0196771904
Goniomonas_pacifica_CCMP1869 CAMPEP_0175808266
Alexandrium_monilatum_CCMP3105 CAMPEP_0175552894
Alexandrium_monilatum_CCMP3105 CAMPEP_0175401274
Alexandrium_monilatum_CCMP3105 CAMPEP_0175714332
Alexandrium_monilatum_CCMP3105 CAMPEP_0175227402
Alexandrium_monilatum_CCMP3105 CAMPEP_0175606720
Alexandrium_monilatum_CCMP3105 CAMPEP_0175631682
Pyrodinium_bahamense_pbaha01 CAMPEP_0179068416
Lingulodinium_polyedra_CCMP_1738 CAMPEP_0118102356
Lingulodinium_polyedra_CCMP_1738 CAMPEP_0118488836
Lingulodinium_polyedra_CCMP_1738 CAMPEP_0117966886
Lingulodinium_polyedra_CCMP_1738 CAMPEP_0118316306
Karenia_brevis_SP1 CAMPEP_0117345546
Karenia_brevis_CCMP2229 CAMPEP_0174076428
Karlodinium_micrum_CCMP2283 CAMPEP_0169277874
Gymnodinium_catenatum_GC744 CAMPEP_0117463132
Alexandrium_tamarense CAMPEP_0116291150
Alexandrium_tamarense CAMPEP_0116343370
Ceratium_fusus_PA161109 CAMPEP_0172697822
Ceratium_fusus_PA161109 CAMPEP_0172832934
Pelagodinium_bii_RCC1491 CAMPEP_0197679322
Symbiodinium_sp._Mp CAMPEP_0169912362
Symbiodinium_sp._Mp CAMPEP_0169896380
Symbiodinium_sp._CCMP421 CAMPEP_0181531280
Symbiodinium_sp._CCMP421 CAMPEP_0181408986
Symbiodinium_sp._C1 CAMPEP_0202597430
Prymnesium_parvum_Texoma1 CAMPEP_0195620978
Prymnesium_parvum_Texoma1 CAMPEP_0195645196
Chrysochromulina_rotalis_UIO044 CAMPEP_0115867012
Bicosoecid_sp._ms1 CAMPEP_0203835634
Cafeteria_roenbergensis_E4-10 CAMPEP_0117714446
Dinobryon_sp._UTEXLB2267 CAMPEP_0170057696
Paraphysomonas_imperforata_PA2 CAMPEP_0114460976
Spumella_elongata_CCAP_955/1 CAMPEP_0184986442
Durinskia_baltica_CSIRO_CS-38 CAMPEP_0170358602
Ochromonas_sp._CCMP1393 CAMPEP_0175016436
Ochromonas_sp._CCMP1393 CAMPEP_0174960034
Ochromonas_sp._CCMP1393 CAMPEP_0175006740
Bolidomonas_pacifica_CCMP_1866 CAMPEP_0118657836
Pteridomonas_danica_PT CAMPEP_0114337728
Rhizochromulina_marina_cf_CCMP1243 CAMPEP_0118973904
Neobodo_designis_CCAP_1951/1 CAMPEP_0174835286
Platyophrya_macrostoma_WH CAMPEP_0176425752
Platyophrya_macrostoma_WH CAMPEP_0176468136
NonPhotosyntheticDiatom QUERY_NAA26P01490.m1
DNA alkylation response protein/acyl-CoA dehydrogenase
0.2

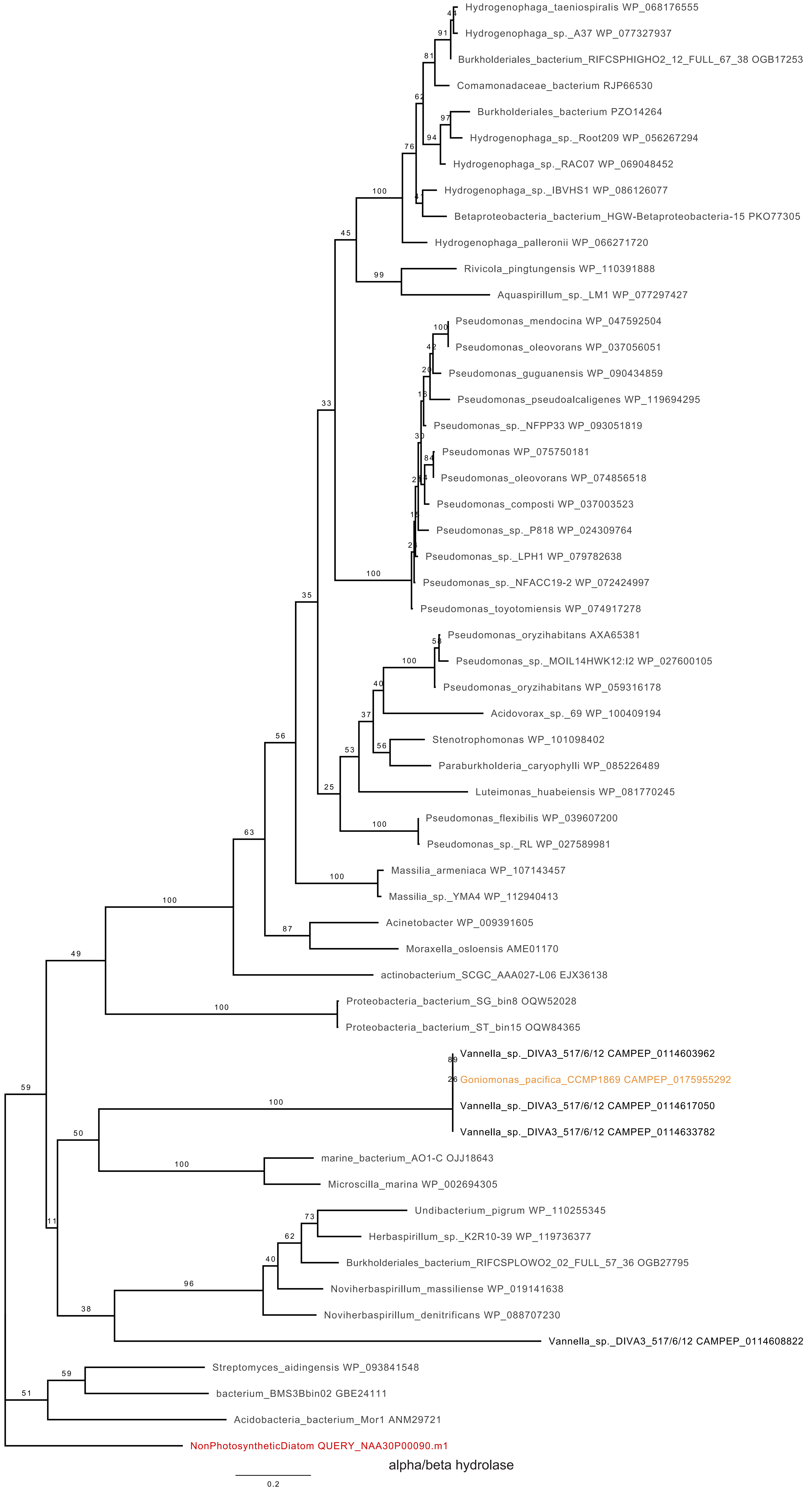

alpha/beta hydrolase

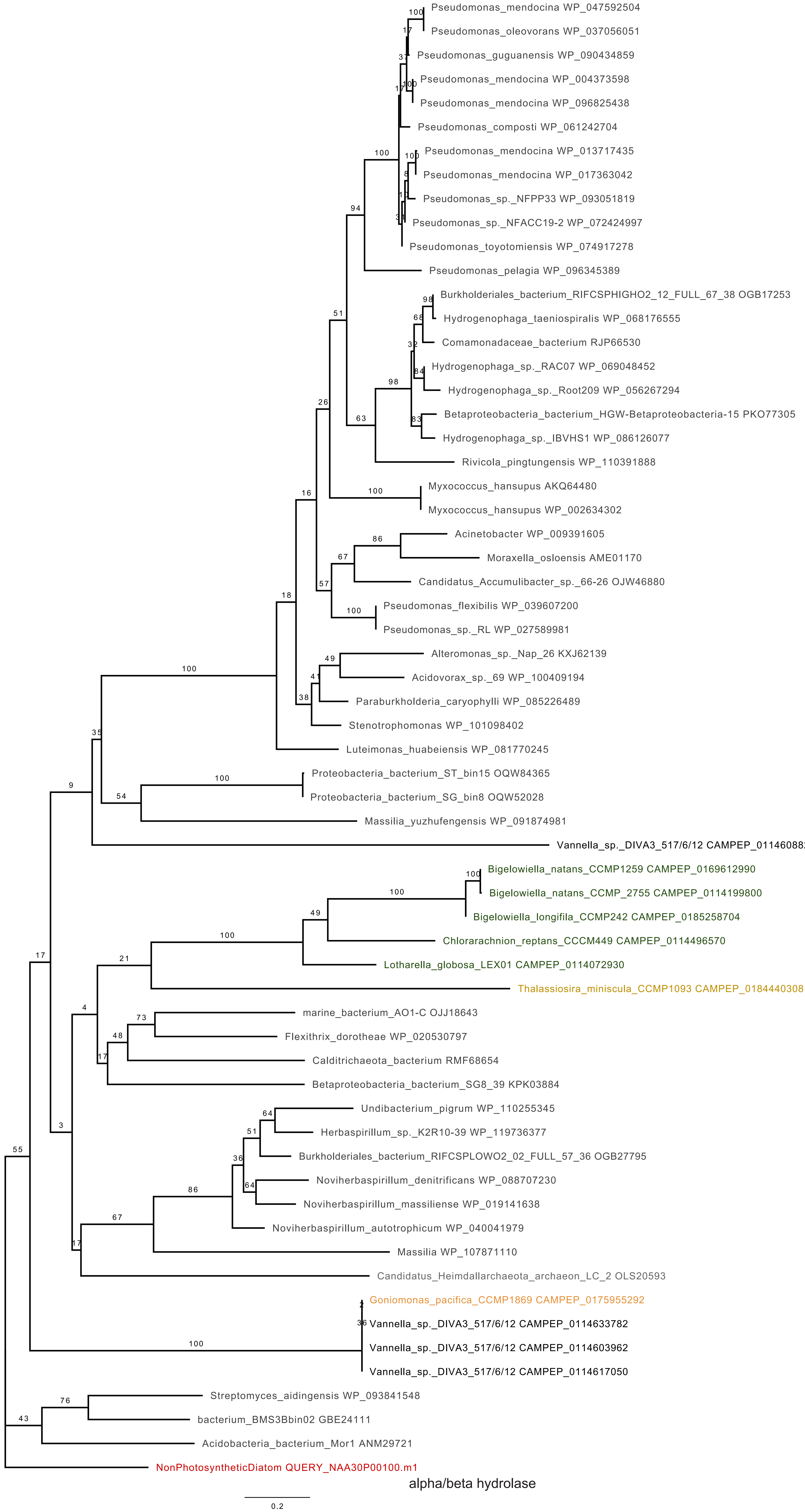
Pseudomonas_mendocina WP_047592504
Pseudomonas_oleovorans WP_037056051
Pseudomonas_guguanensis WP_090434859
Pseudomonas_mendocina WP_004373598
Pseudomonas_mendocina WP_096825438
Pseudomonas_composti WP_061242704
Pseudomonas_mendocina WP_013717435
Pseudomonas_mendocina WP_017363042
Pseudomonas_sp._NFPP33 WP_093051819
Pseudomonas_sp._NFACC19-2 WP_072424997
Pseudomonas_toyotomiensis WP_074917278
Pseudomonas_pelagia WP_096345389
Burkholderiales_bacterium_RIFCSPHIGHO2_12_FULL_67_38 OGB17253
Hydrogenophaga_taeniospiralis WP_068176555
Comamonadaceae_bacterium RJP66530
Hydrogenophaga_sp._RAC07 WP_069048452
Hydrogenophaga_sp._Root209 WP_056267294
Betaproteobacteria_bacterium_HGW-Betaproteobacteria-15 PKO77305
Hydrogenophaga_sp._IBVHS1 WP_086126077
Rivicola_pingtungensis WP_110391888
Myxococcus_hansupus AKQ64480
Myxococcus_hansupus WP_002634302
Acinetobacter WP_009391605
Moraxella_osloensis AME01170
Candidatus_Accumulibacter_sp._66-26 OJW46880
Pseudomonas_flexibilis WP_039607200
Pseudomonas_sp._RL WP_027589981
Alteromonas_sp._Nap_26 KXJ62139
Acidovorax_sp._69 WP_100409194
Paraburkholderia_caryophylli WP_085226489
Stenotrophomonas WP_101098402
Luteimonas_huabeiensis WP_081770245
Proteobacteria_bacterium_ST_bin15 OQW84365
Proteobacteria_bacterium_SG_bin8 OQW52028
Massilia_yuzhufengensis WP_091874981
Vannella_sp._DIVA3_517/6/12 CAMPEP_0114608822
Bigelowiella_natans_CCMP1259 CAMPEP_0169612990
Bigelowiella_natans_CCMP_2755 CAMPEP_0114199800
Bigelowiella_longifila_CCMP242 CAMPEP_0185258704
Chlorarachnion_reptans_CCCM449 CAMPEP_0114496570
Lotharella_globosa_LEX01 CAMPEP_0114072930
Thalassiosira_miniscula_CCMP1093 CAMPEP_0184440308
marine_bacterium_AO1-C OJJ18643
Flexithrix_dorotheae WP_020530797
Calditrichaeota_bacterium RMF68654
Betaproteobacteria_bacterium_SG8_39 KPK03884
Undibacterium_pigrum WP_110255345
Herbaspirillum_sp._K2R10-39 WP_119736377
Burkholderiales_bacterium_RIFCSPLOWO2_02_FULL_57_36 OGB27795
Noviherbaspirillum_denitrificans WP_088707230
Noviherbaspirillum_massiliense WP_019141638
Noviherbaspirillum_autotrophicum WP_040041979
Massilia WP_107871110
Candidatus_Heimdallarchaeota_archaeon_LC_2 OLS20593
Goniomonas_pacifica_CCMP1869 CAMPEP_0175955292
Vannella_sp._DIVA3_517/6/12 CAMPEP_0114633782
Vannella_sp._DIVA3_517/6/12 CAMPEP_0114603962
Vannella_sp._DIVA3_517/6/12 CAMPEP_0114617050
Streptomyces_aidingensis WP_093841548
bacterium_BMS3Bbin02 GBE24111
Acidobacteria_bacterium_Mor1 ANM29721
NonPhotosyntheticDiatom QUERY_NAA30P00100.m1
alpha/beta hydrolase
0.2

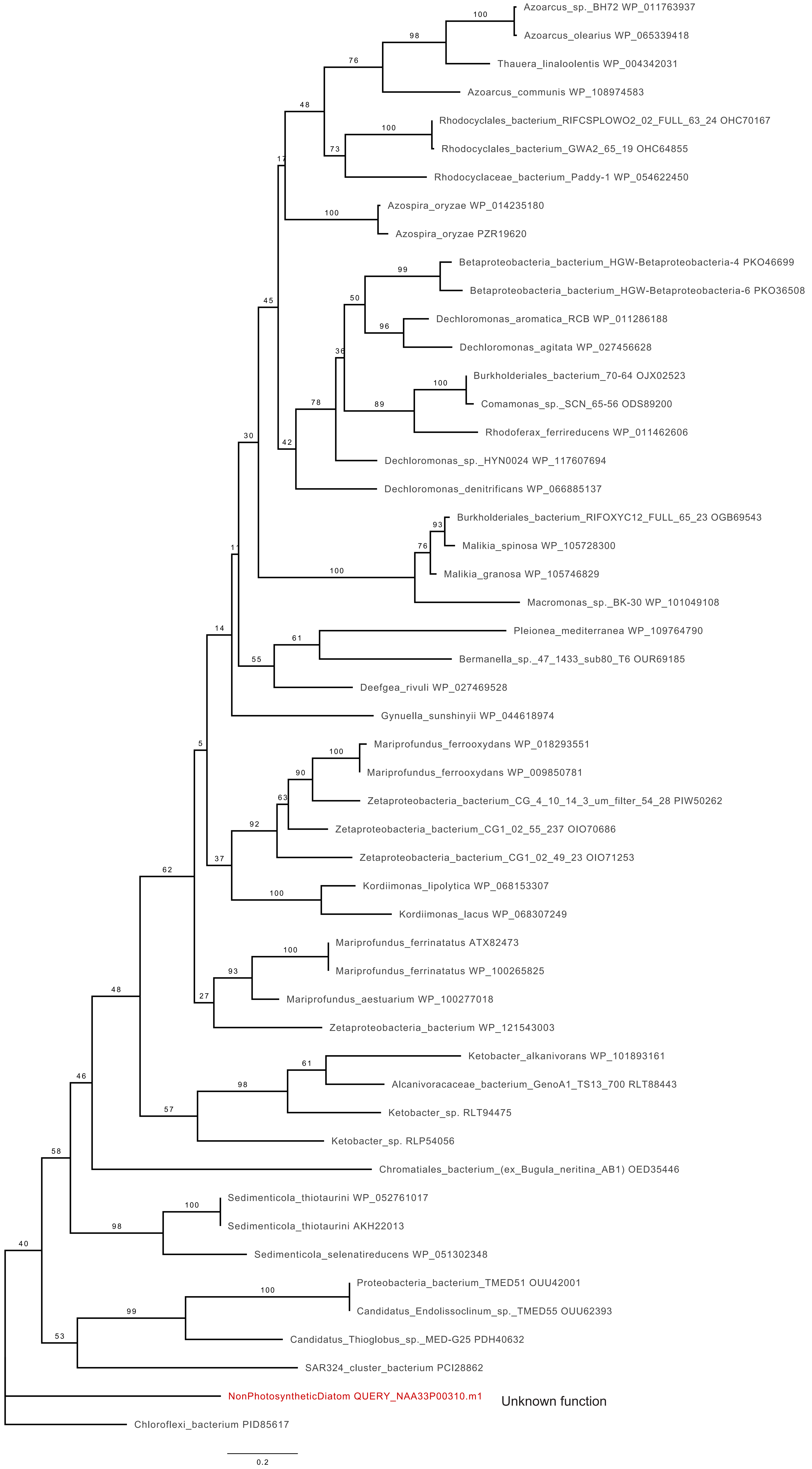
Azoarcus_sp._BH72 WP_011763937
Azoarcus_olearius WP_065339418
Thauera_linaloolentis WP_004342031
Azoarcus_communis WP_108974583
Rhodocyclales_bacterium_RIFCSPLOWO2_02_FULL_63_24 OHC70167
Rhodocyclales_bacterium_GWA2_65_19 OHC64855
Rhodocyclaceae_bacterium_Paddy-1 WP_054622450
Azospira_oryzae WP_014235180
Azospira_oryzae PZR19620
Betaproteobacteria_bacterium_HGW-Betaproteobacteria-4 PKO46699
Betaproteobacteria_bacterium_HGW-Betaproteobacteria-6 PKO36508
Dechloromonas_aromatica_RCB WP_011286188
Dechloromonas_agitata WP_027456628
Burkholderiales_bacterium_70-64 OJX02523
Comamonas_sp._SCN_65-56 ODS89200
Rhodoferax_ferrireducens WP_011462606
Dechloromonas_sp._HYN0024 WP_117607694
Dechloromonas_denitrificans WP_066885137
Burkholderiales_bacterium_RIFOXYC12_FULL_65_23 OGB69543
Malikia_spinosa WP_105728300
Malikia_granosa WP_105746829
Macromonas_sp._BK-30 WP_101049108
Pleionea_mediterranea WP_109764790
Bermanella_sp._47_1433_sub80_T6 OUR69185
Deefgea_rivuli WP_027469528
Gynuella_sunshinyii WP_044618974
Mariprofundus_ferrooxydans WP_018293551
Mariprofundus_ferrooxydans WP_009850781
Zetaproteobacteria_bacterium_CG_4_10_14_3_um_filter_54_28 PIW50262
Zetaproteobacteria_bacterium_CG1_02_55_237 OIO70686
Zetaproteobacteria_bacterium_CG1_02_49_23 OIO71253
Kordiimonas_lipolytica WP_068153307
Kordiimonas_lacus WP_068307249
Mariprofundus_ferrinatatus ATX82473
Mariprofundus_ferrinatatus WP_100265825
Mariprofundus_aestuarium WP_100277018
Zetaproteobacteria_bacterium WP_121543003
Ketobacter_alkanivorans WP_101893161
Alcanivoracaceae_bacterium_GenoA1_TS13_700 RLT88443
Ketobacter_sp. RLT94475
Ketobacter_sp. RLP54056
Chromatiales_bacterium_(ex_Bugula_neritina_AB1) OED35446
Sedimenticola_thiotaurini WP_052761017
Sedimenticola_thiotaurini AKH22013
Sedimenticola_selenatireducens WP_051302348
Proteobacteria_bacterium_TMED51 OUU42001
Candidatus_Endolissoclinum_sp._TMED55 OUU62393
Candidatus_Thioglobus_sp._MED-G25 PDH40632
SAR324_cluster_bacterium PCI28862
NonPhotosyntheticDiatom QUERY_NAA33P00310.m1
Unknown function
Chloroflexi_bacterium PID85617
0.2

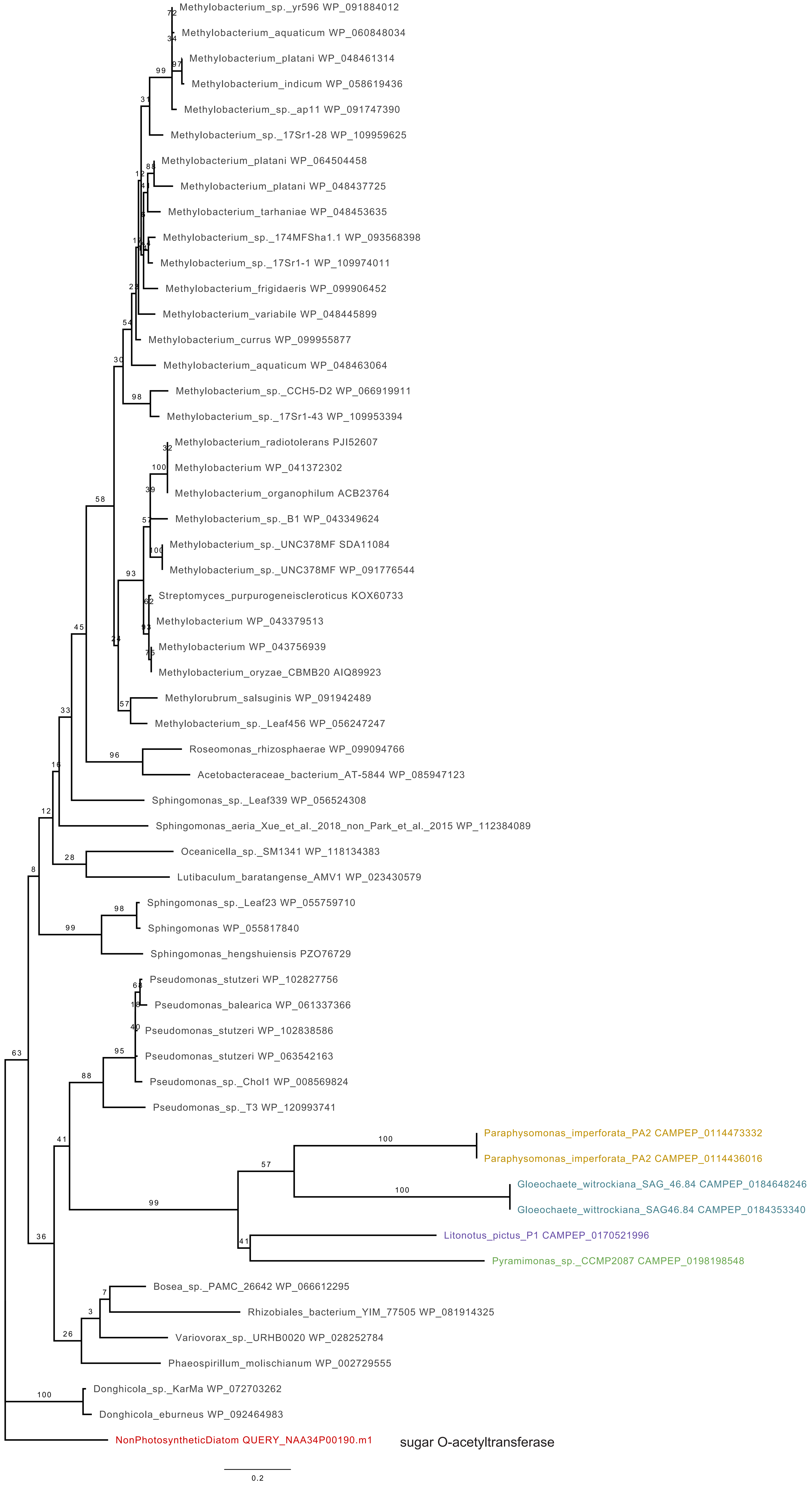
Methylobacterium_sp._yr596 WP_091884012
Methylobacterium_aquaticum WP_060848034
Methylobacterium_platani WP_048461314
Methylobacterium_indicum WP_058619436
Methylobacterium_sp._ap11 WP_091747390
Methylobacterium_sp._17Sr1-28 WP_109959625
Methylobacterium_platani WP_064504458
Methylobacterium_platani WP_048437725
Methylobacterium_tarhaniae WP_048453635
Methylobacterium_sp._174MFSha1.1 WP_093568398
Methylobacterium_sp._17Sr1-1 WP_109974011
Methylobacterium_frigidaeris WP_099906452
Methylobacterium_variabile WP_048445899
Methylobacterium_currus WP_099955877
Methylobacterium_aquaticum WP_048463064
Methylobacterium_sp._CCH5-D2 WP_066919911
Methylobacterium_sp._17Sr1-43 WP_109953394
Methylobacterium_radiotolerans PJI52607
Methylobacterium WP_041372302
Methylobacterium_organophilum ACB23764
Methylobacterium_sp._B1 WP_043349624
Methylobacterium_sp._UNC378MF SDA11084
Methylobacterium_sp._UNC378MF WP_091776544
Streptomyces_purpurogeneiscleroticus KOX60733
Methylobacterium WP_043379513
Methylobacterium WP_043756939
Methylobacterium_oryzae_CBMB20 AIQ89923
Methylorubrum_salsuginis WP_091942489
Methylobacterium_sp._Leaf456 WP_056247247
Roseomonas_rhizosphaerae WP_099094766
Acetobacteraceae_bacterium_AT-5844 WP_085947123
Sphingomonas_sp._Leaf339 WP_056524308
Sphingomonas_aeria_Xue_et_al._2018_non_Park_et_al._2015 WP_112384089
Oceanicella_sp._SM1341 WP_118134383
Lutibaculum_baratangense_AMV1 WP_023430579
Sphingomonas_sp._Leaf23 WP_055759710
Sphingomonas WP_055817840
Sphingomonas_hengshuiensis PZO76729
Pseudomonas_stutzeri WP_102827756
Pseudomonas_balearica WP_061337366
Pseudomonas_stutzeri WP_102838586
Pseudomonas_stutzeri WP_063542163
Pseudomonas_sp._Chol1 WP_008569824
Pseudomonas_sp._T3 WP_120993741
Paraphysomonas_imperforata_PA2 CAMPEP_0114473332
Paraphysomonas_imperforata_PA2 CAMPEP_0114436016
Gloeochaete_witrockiana_SAG_46.84 CAMPEP_0184648246
Gloeochaete_wittrockiana_SAG46.84 CAMPEP_0184353340
Litonotus_pictus_P1 CAMPEP_0170521996
Pyramimonas_sp._CCMP2087 CAMPEP_0198198548
Bosea_sp._PAMC_26642 WP_066612295
Rhizobiales_bacterium_YIM_77505 WP_081914325
Variovorax_sp._URHB0020 WP_028252784
Phaeospirillum_molischianum WP_002729555
Donghicola_sp._KarMa WP_072703262
Donghicola_eburneus WP_092464983
NonPhotosyntheticDiatom QUERY_NAA34P00190.m1
sugar O-acetyltransferase
0.2

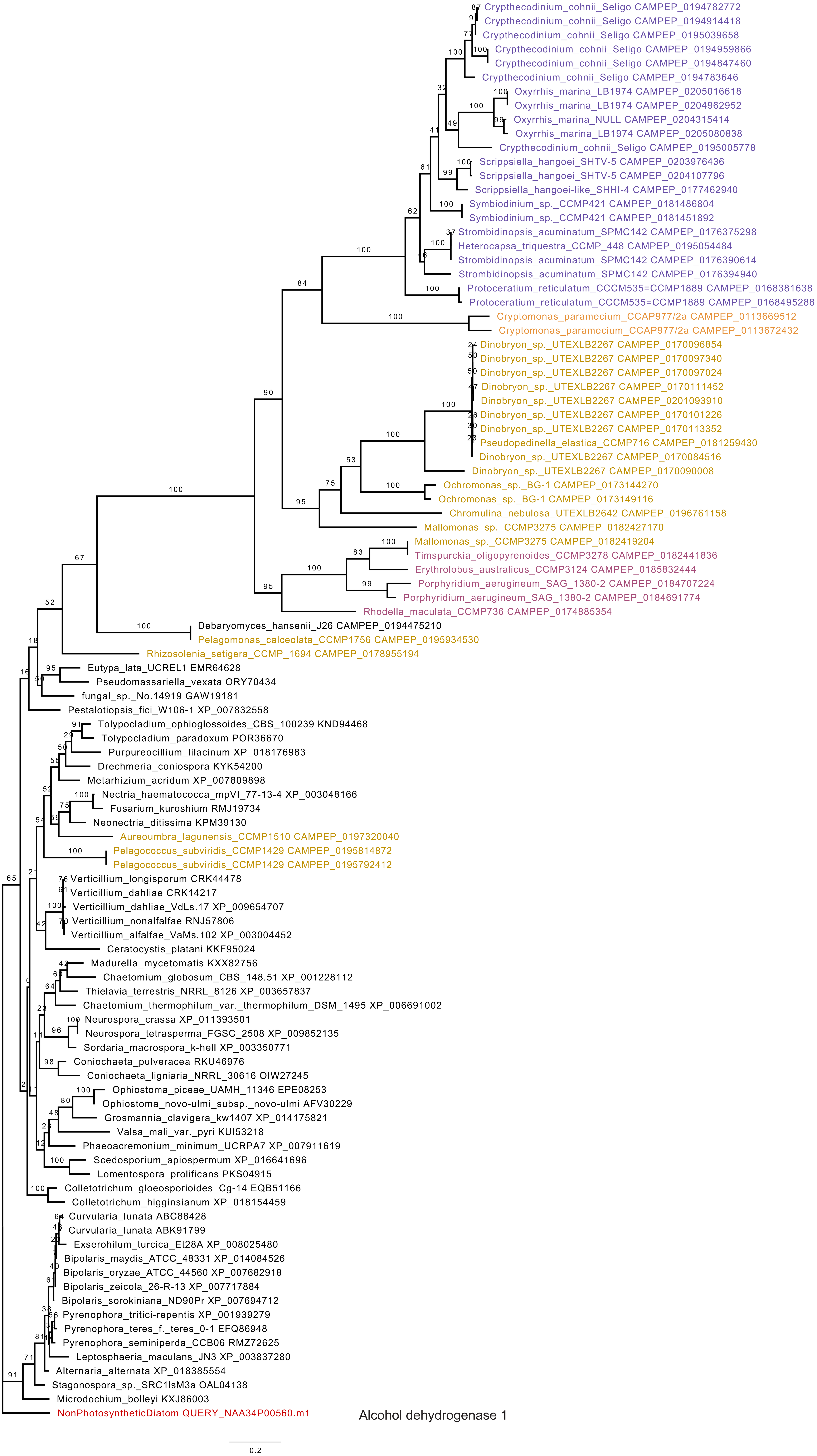
Crypthecodinium_cohnii_Seligo CAMPEP_0194782772
Crypthecodinium_cohnii_Seligo CAMPEP_0194914418
Crypthecodinium_cohnii_Seligo CAMPEP_0195039658
Crypthecodinium_cohnii_Seligo CAMPEP_0194959866
Crypthecodinium_cohnii_Seligo CAMPEP_0194847460
Crypthecodinium_cohnii_Seligo CAMPEP_0194783646
Oxyrrhis_marina_LB1974 CAMPEP_0205016618
Oxyrrhis_marina_LB1974 CAMPEP_0204962952
Oxyrrhis_marina_NULL CAMPEP_0204315414
Oxyrrhis_marina_LB1974 CAMPEP_0205080838
Crypthecodinium_cohnii_Seligo CAMPEP_0195005778
Scrippsiella_hangoei_SHTV-5 CAMPEP_0203976436
Scrippsiella_hangoei_SHTV-5 CAMPEP_0204107796
Scrippsiella_hangoei-like_SHHI-4 CAMPEP_0177462940
Symbiodinium_sp._CCMP421 CAMPEP_0181486804
Symbiodinium_sp._CCMP421 CAMPEP_0181451892
Strombidinopsis_acuminatum_SPMC142 CAMPEP_0176375298
Heterocapsa_triquestra_CCMP_448 CAMPEP_0195054484
Strombidinopsis_acuminatum_SPMC142 CAMPEP_0176390614
Strombidinopsis_acuminatum_SPMC142 CAMPEP_0176394940
Protoceratium_reticulatum_CCCM535=CCMP1889 CAMPEP_0168381638
Protoceratium_reticulatum_CCCM535=CCMP1889 CAMPEP_0168495288
Cryptomonas_paramecium_CCAP977/2a CAMPEP_0113669512
Cryptomonas_paramecium_CCAP977/2a CAMPEP_0113672432
Dinobryon_sp._UTEXLB2267 CAMPEP_0170096854
Dinobryon_sp._UTEXLB2267 CAMPEP_0170097340
Dinobryon_sp._UTEXLB2267 CAMPEP_0170097024
Dinobryon_sp._UTEXLB2267 CAMPEP_0170111452
Dinobryon_sp._UTEXLB2267 CAMPEP_0201093910
Dinobryon_sp._UTEXLB2267 CAMPEP_0170101226
Dinobryon_sp._UTEXLB2267 CAMPEP_0170113352
Pseudopedinella_elastica_CCMP716 CAMPEP_0181259430
Dinobryon_sp._UTEXLB2267 CAMPEP_0170084516
Dinobryon_sp._UTEXLB2267 CAMPEP_0170090008
Ochromonas_sp._BG-1 CAMPEP_0173144270
Ochromonas_sp._BG-1 CAMPEP_0173149116
Chromulina_nebulosa_UTEXLB2642 CAMPEP_0196761158
Mallomonas_sp._CCMP3275 CAMPEP_0182427170
Mallomonas_sp._CCMP3275 CAMPEP_0182419204
Timspurckia_oligopyrenoides_CCMP3278 CAMPEP_0182441836
Erythrolobus_australicus_CCMP3124 CAMPEP_0185832444
Porphyridium_aerugineum_SAG_1380-2 CAMPEP_0184707224
Porphyridium_aerugineum_SAG_1380-2 CAMPEP_0184691774
Rhodella_maculata_CCMP736 CAMPEP_0174885354
Debaryomyces_hansenii_J26 CAMPEP_0194475210
Pelagomonas_calceolata_CCMP1756 CAMPEP_0195934530
Rhizosolenia_setigera_CCMP_1694 CAMPEP_0178955194
Eutypa_lata_UCREL1 EMR64628
Pseudomassariella_vexata ORY70434
fungal_sp._No.14919 GAW19181
Pestalotiopsis_fici_W106-1 XP_007832558
Tolypocladium_ophioglossoides_CBS_100239 KND94468
Tolypocladium_paradoxum POR36670
Purpureocillium_lilacinum XP_018176983
Drechmeria_coniospora KYK54200
Metarhizium_acridum XP_007809898
Nectria_haematococca_mpVI_77-13-4 XP_003048166
Fusarium_kuroshium RMJ19734
Neonectria_ditissima KPM39130
Aureoumbra_lagunensis_CCMP1510 CAMPEP_0197320040
Pelagococcus_subviridis_CCMP1429 CAMPEP_0195814872
Pelagococcus_subviridis_CCMP1429 CAMPEP_0195792412
Verticillium_longisporum CRK44478
Verticillium_dahliae CRK14217
Verticillium_dahliae_VdLs.17 XP_009654707
Verticillium_nonalfalfae RNJ57806
Verticillium_alfalfae_VaMs.102 XP_003004452
Ceratocystis_platani KKF95024
Madurella_mycetomatis KXX82756
Chaetomium_globosum_CBS_148.51 XP_001228112
Thielavia_terrestris_NRRL_8126 XP_003657837
Chaetomium_thermophilum_var._thermophilum_DSM_1495 XP_006691002
Neurospora_crassa XP_011393501
Neurospora_tetrasperma_FGSC_2508 XP_009852135
Sordaria_macrospora_k-hell XP_003350771
Coniochaeta_pulveracea RKU46976
Coniochaeta_ligniaria_NRRL_30616 OIW27245
Ophiostoma_piceae_UAMH_11346 EPE08253
Ophiostoma_novo-ulmi_subsp._novo-ulmi AFV30229
Grosmannia_clavigera_kw1407 XP_014175821
Valsa_mali_var._pyri KUI53218
Phaeoacremonium_minimum_UCRPA7 XP_007911619
Scedosporium_apiospermum XP_016641696
Lomentospora_prolificans PKS04915
Colletotrichum_gloeosporioides_Cg-14 EQB51166
Colletotrichum_higginsianum XP_018154459
Curvularia_lunata ABC88428
Curvularia_lunata ABK91799
Exserohilum_turcica_Et28A XP_008025480
Bipolaris_maydis_ATCC_48331 XP_014084526
Bipolaris_oryzae_ATCC_44560 XP_007682918
Bipolaris_zeicola_26-R-13 XP_007717884
Bipolaris_sorokiniana_ND90Pr XP_007694712
Pyrenophora_tritici-repentis XP_001939279
Pyrenophora_teres_f._teres_0-1 EFQ86948
Pyrenophora_seminiperda_CCB06 RMZ72625
Leptosphaeria_maculans_JN3 XP_003837280
Alternaria_alternata XP_018385554
Stagonospora_sp._SRC1lsM3a OAL04138
Microdochium_bolleyi KXJ86003
NonPhotosyntheticDiatom QUERY_NAA34P00560.m1
Alcohol dehydrogenase 1
0.2

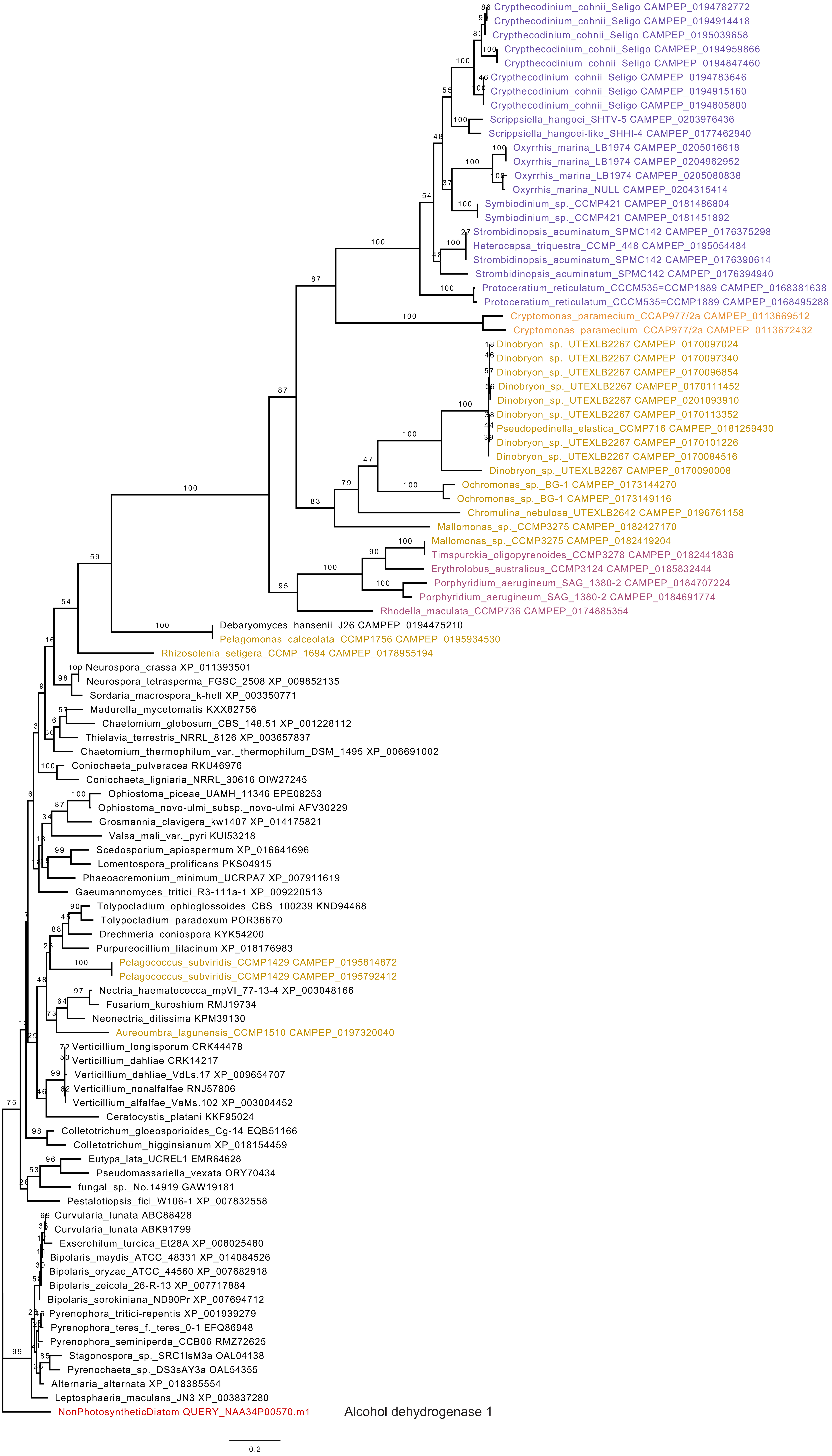

Crypthecodinium_cohnii_Seligo CAMPEP_0194782772
Crypthecodinium_cohnii_Seligo CAMPEP_0194914418
Crypthecodinium_cohnii_Seligo CAMPEP_0195039658
Crypthecodinium_cohnii_Seligo CAMPEP_0194959866
Crypthecodinium_cohnii_Seligo CAMPEP_0194847460
Crypthecodinium_cohnii_Seligo CAMPEP_0194783646
Crypthecodinium_cohnii_Seligo CAMPEP_0194915160
Crypthecodinium_cohnii_Seligo CAMPEP_0194805800
Scrippsiella_hangoei_SHTV-5 CAMPEP_0203976436
Scrippsiella_hangoei-like_SHHI-4 CAMPEP_0177462940
Oxyrrhis_marina_LB1974 CAMPEP_0205016618
Oxyrrhis_marina_LB1974 CAMPEP_0204962952
Oxyrrhis_marina_LB1974 CAMPEP_0205080838
Oxyrrhis_marina_NULL CAMPEP_0204315414
Symbiodinium_sp._CCMP421 CAMPEP_0181486804
Symbiodinium_sp._CCMP421 CAMPEP_0181451892
Strombidinopsis_acuminatum_SPMC142 CAMPEP_0176375298
Heterocapsa_triquestra_CCMP_448 CAMPEP_0195054484
Strombidinopsis_acuminatum_SPMC142 CAMPEP_0176390614
Strombidinopsis_acuminatum_SPMC142 CAMPEP_0176394940
Protoceratium_reticulatum_CCCM535=CCMP1889 CAMPEP_0168381638
Protoceratium_reticulatum_CCCM535=CCMP1889 CAMPEP_0168495288
Cryptomonas_paramecium_CCAP977/2a CAMPEP_0113669512
Cryptomonas_paramecium_CCAP977/2a CAMPEP_0113672432
Dinobryon_sp._UTEXLB2267 CAMPEP_0170097024
Dinobryon_sp._UTEXLB2267 CAMPEP_0170097340
Dinobryon_sp._UTEXLB2267 CAMPEP_0170096854
Dinobryon_sp._UTEXLB2267 CAMPEP_0170111452
Dinobryon_sp._UTEXLB2267 CAMPEP_0201093910
Dinobryon_sp._UTEXLB2267 CAMPEP_0170113352
Pseudopedinella_elastica_CCMP716 CAMPEP_0181259430
Dinobryon_sp._UTEXLB2267 CAMPEP_0170101226
Dinobryon_sp._UTEXLB2267 CAMPEP_0170084516
Dinobryon_sp._UTEXLB2267 CAMPEP_0170090008
Ochromonas_sp._BG-1 CAMPEP_0173144270
Ochromonas_sp._BG-1 CAMPEP_0173149116
Chromulina_nebulosa_UTEXLB2642 CAMPEP_0196761158
Mallomonas_sp._CCMP3275 CAMPEP_0182427170
Mallomonas_sp._CCMP3275 CAMPEP_0182419204
Timspurckia_oligopyrenoides_CCMP3278 CAMPEP_0182441836
Erythrolobus_australicus_CCMP3124 CAMPEP_0185832444
Porphyridium_aerugineum_SAG_1380-2 CAMPEP_0184707224
Porphyridium_aerugineum_SAG_1380-2 CAMPEP_0184691774
Rhodella_maculata_CCMP736 CAMPEP_0174885354
Debaryomyces_hansenii_J26 CAMPEP_0194475210
Pelagomonas_calceolata_CCMP1756 CAMPEP_0195934530
Rhizosolenia_setigera_CCMP_1694 CAMPEP_0178955194
Neurospora_crassa XP_011393501
Neurospora_tetrasperma_FGSC_2508 XP_009852135
Sordaria_macrospora_k-hell XP_003350771
Madurella_mycetomatis KXX82756
Chaetomium_globosum_CBS_148.51 XP_001228112
Thielavia_terrestris_NRRL_8126 XP_003657837
Chaetomium_thermophilum_var._thermophilum_DSM_1495 XP_006691002
Coniochaeta_pulveracea RKU46976
Coniochaeta_ligniaria_NRRL_30616 OIW27245
Ophiostoma_piceae_UAMH_11346 EPE08253
Ophiostoma_novo-ulmi_subsp._novo-ulmi AFV30229
Grosmannia_clavigera_kw1407 XP_014175821
Valsa_mali_var._pyri KUI53218
Scedosporium_apiospermum XP_016641696
Lomentospora_prolificans PKS04915
Phaeoacremonium_minimum_UCRPA7 XP_007911619
Gaeumannomyces_tritici_R3-111a-1 XP_009220513
Tolypocladium_ophioglossoides_CBS_100239 KND94468
Tolypocladium_paradoxum POR36670
Drechmeria_coniospora KYK54200
Purpureocillium_lilacinum XP_018176983
Pelagococcus_subviridis_CCMP1429 CAMPEP_0195814872
Pelagococcus_subviridis_CCMP1429 CAMPEP_0195792412
Nectria_haematococca_mpVI_77-13-4 XP_003048166
Fusarium_kuroshium RMJ19734
Neonectria_ditissima KPM39130
Aureoumbra_lagunensis_CCMP1510 CAMPEP_0197320040
Verticillium_longisporum CRK44478
Verticillium_dahliae CRK14217
Verticillium_dahliae_VdLs.17 XP_009654707
Verticillium_nonalfalfae RNJ57806
Verticillium_alfalfae_VaMs.102 XP_003004452
Ceratocystis_platani KKF95024
Colletotrichum_gloeosporioides_Cg-14 EQB51166
Colletotrichum_higginsianum XP_018154459
Eutypa_lata_UCREL1 EMR64628
Pseudomassariella_vexata ORY70434
fungal_sp._No.14919 GAW19181
Pestalotiopsis_fici_W106-1 XP_007832558
Curvularia_lunata ABC88428
Curvularia_lunata ABK91799
Exserohilum_turcica_Et28A XP_008025480
Bipolaris_maydis_ATCC_48331 XP_014084526
Bipolaris_oryzae_ATCC_44560 XP_007682918
Bipolaris_zeicola_26-R-13 XP_007717884
Bipolaris_sorokiniana_ND90Pr XP_007694712
Pyrenophora_tritici-repentis XP_001939279
Pyrenophora_teres_f._teres_0-1 EFQ86948
Pyrenophora_seminiperda_CCB06 RMZ72625
Stagonospora_sp._SRC1lsM3a OAL04138
Pyrenochaeta_sp._DS3sAY3a OAL54355
Alternaria_alternata XP_018385554
Leptosphaeria_maculans_JN3 XP_003837280
NonPhotosyntheticDiatom QUERY_NAA34P00570.m1
Alcohol dehydrogenase 1
0.2

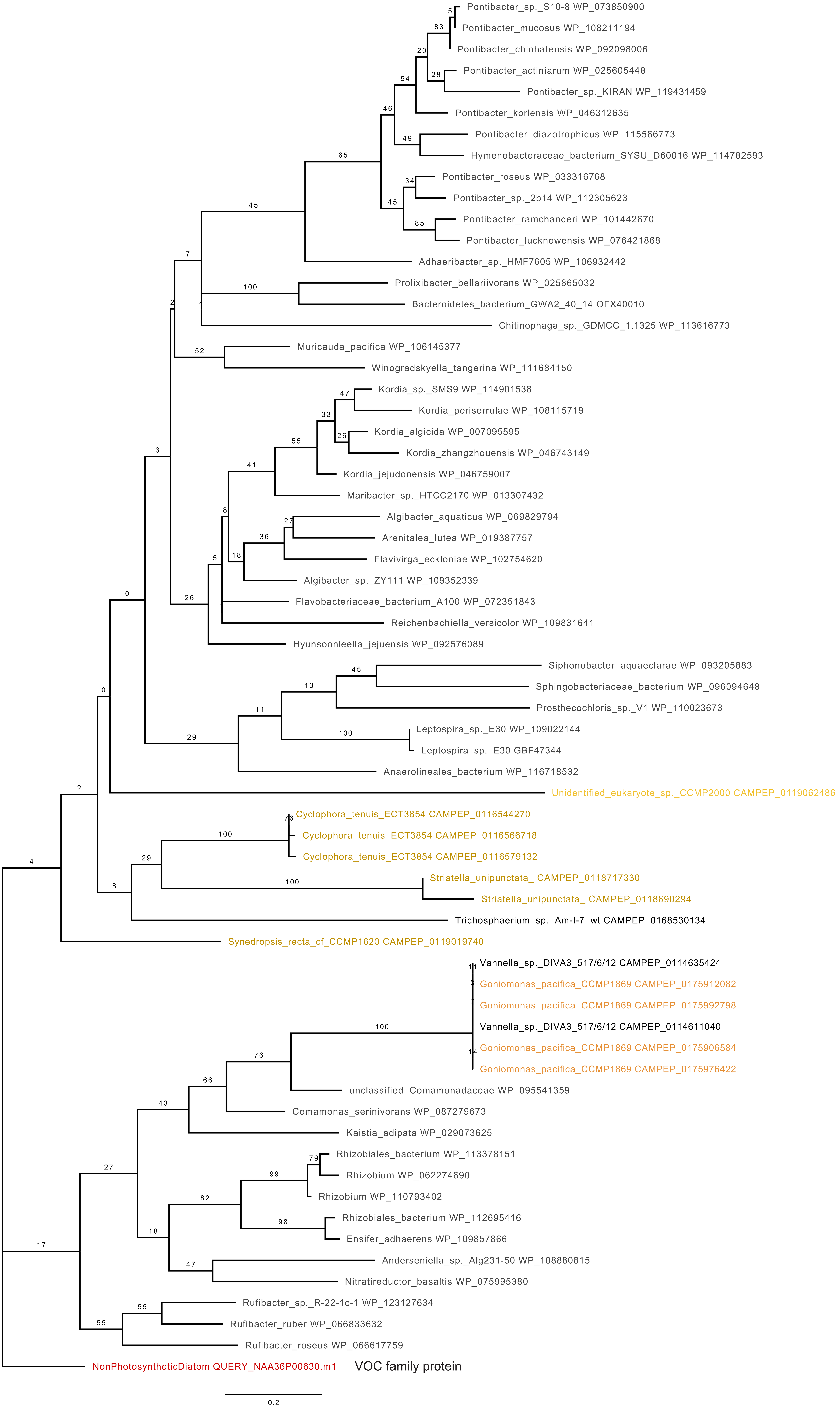
Pontibacter_sp._S10-8 WP_073850900
Pontibacter_mucosus WP_108211194
Pontibacter_chinhatensis WP_092098006
Pontibacter_actiniarum WP_025605448
Pontibacter_sp._KIRAN WP_119431459
Pontibacter_korlensis WP_046312635
Pontibacter_diazotrophicus WP_115566773
Hymenobacteraceae_bacterium_SYSU_D60016 WP_114782593
Pontibacter_roseus WP_033316768
Pontibacter_sp._2b14 WP_112305623
Pontibacter_ramchanderi WP_101442670
Pontibacter_lucknowensis WP_076421868
Adhaeribacter_sp._HMF7605 WP_106932442
Prolixibacter_bellariivorans WP_025865032
Bacteroidetes_bacterium_GWA2_40_14 OFX40010
Chitinophaga_sp._GDMCC_1.1325 WP_113616773
Muricauda_pacifica WP_106145377
Winogradskyella_tangerina WP_111684150
Kordia_sp._SMS9 WP_114901538
Kordia_periserrulae WP_108115719
Kordia_algicida WP_007095595
Kordia_zhangzhouensis WP_046743149
Kordia_jejudonensis WP_046759007
Maribacter_sp._HTCC2170 WP_013307432
Algibacter_aquaticus WP_069829794
Arenitalea_lutea WP_019387757
Flavivirga_eckloniae WP_102754620
Algibacter_sp._ZY111 WP_109352339
Flavobacteriaceae_bacterium_A100 WP_072351843
Reichenbachiella_versicolor WP_109831641
Hyunsoonleella_jejuensis WP_092576089
Siphonobacter_aquaeclarae WP_093205883
Sphingobacteriaceae_bacterium WP_096094648
Prosthecochloris_sp._V1 WP_110023673
Leptospira_sp._E30 WP_109022144
Leptospira_sp._E30 GBF47344
Anaerolineales_bacterium WP_116718532
Unidentified_eukaryote_sp._CCMP2000 CAMPEP_0119062486
Cyclophora_tenuis_ECT3854 CAMPEP_0116544270
Cyclophora_tenuis_ECT3854 CAMPEP_0116566718
Cyclophora_tenuis_ECT3854 CAMPEP_0116579132
Striatella_unipunctata_ CAMPEP_0118717330
Striatella_unipunctata_ CAMPEP_0118690294
Trichosphaerium_sp._Am-I-7_wt CAMPEP_0168530134
Synedropsis_recta_cf_CCMP1620 CAMPEP_0119019740
Vannella_sp._DIVA3_517/6/12 CAMPEP_0114635424
Goniomonas_pacifica_CCMP1869 CAMPEP_0175912082
Goniomonas_pacifica_CCMP1869 CAMPEP_0175992798
Vannella_sp._DIVA3_517/6/12 CAMPEP_0114611040
Goniomonas_pacifica_CCMP1869 CAMPEP_0175906584
Goniomonas_pacifica_CCMP1869 CAMPEP_0175976422
unclassified_Comamonadaceae WP_095541359
Comamonas_serinivorans WP_087279673
Kaistia_adipata WP_029073625
Rhizobiales_bacterium WP_113378151
Rhizobium WP_062274690
Rhizobium WP_110793402
Rhizobiales_bacterium WP_112695416
Ensifer_adhaerens WP_109857866
Anderseniella_sp._Alg231-50 WP_108880815
Nitratireductor_basaltis WP_075995380
Rufibacter_sp._R-22-1c-1 WP_123127634
Rufibacter_ruber WP_066833632
Rufibacter_roseus WP_066617759
NonPhotosyntheticDiatom QUERY_NAA36P00630.m1
VOC family protein
0.2

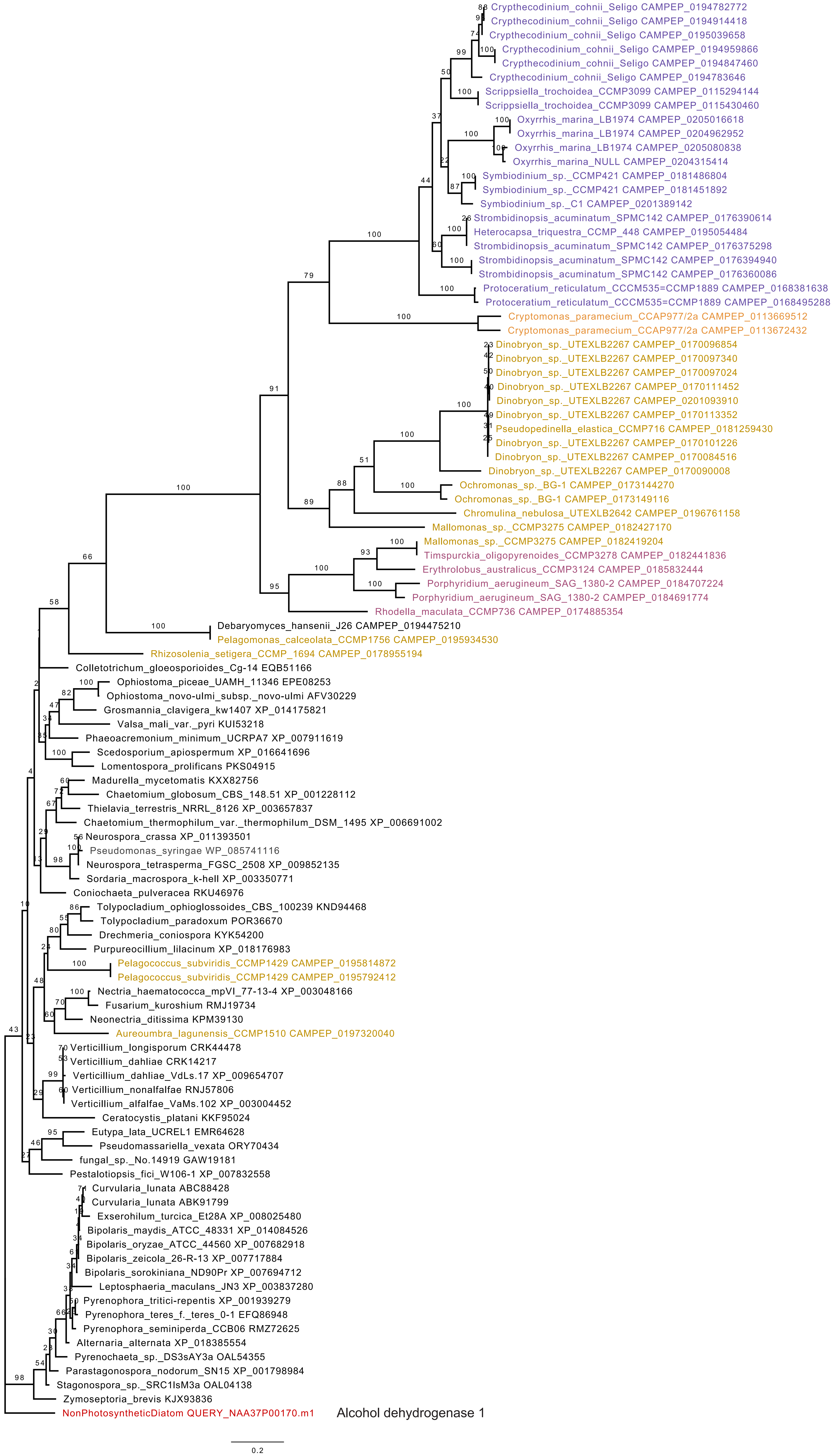
Crypthecodinium_cohnii_Seligo CAMPEP_0194782772
Crypthecodinium_cohnii_Seligo CAMPEP_0194914418
Crypthecodinium_cohnii_Seligo CAMPEP_0195039658
Crypthecodinium_cohnii_Seligo CAMPEP_0194959866
Crypthecodinium_cohnii_Seligo CAMPEP_0194847460
Crypthecodinium_cohnii_Seligo CAMPEP_0194783646
Scrippsiella_trochoidea_CCMP3099 CAMPEP_0115294144
Scrippsiella_trochoidea_CCMP3099 CAMPEP_0115430460
Oxyrrhis_marina_LB1974 CAMPEP_0205016618
Oxyrrhis_marina_LB1974 CAMPEP_0204962952
Oxyrrhis_marina_LB1974 CAMPEP_0205080838
Oxyrrhis_marina_NULL CAMPEP_0204315414
Symbiodinium_sp._CCMP421 CAMPEP_0181486804
Symbiodinium_sp._CCMP421 CAMPEP_0181451892
Symbiodinium_sp._C1 CAMPEP_0201389142
Strombidinopsis_acuminatum_SPMC142 CAMPEP_0176390614
Heterocapsa_triquestra_CCMP_448 CAMPEP_0195054484
Strombidinopsis_acuminatum_SPMC142 CAMPEP_0176375298
Strombidinopsis_acuminatum_SPMC142 CAMPEP_0176394940
Strombidinopsis_acuminatum_SPMC142 CAMPEP_0176360086
Protoceratium_reticulatum_CCCM535=CCMP1889 CAMPEP_0168381638
Protoceratium_reticulatum_CCCM535=CCMP1889 CAMPEP_0168495288
Cryptomonas_paramecium_CCAP977/2a CAMPEP_0113669512
Cryptomonas_paramecium_CCAP977/2a CAMPEP_0113672432
Dinobryon_sp._UTEXLB2267 CAMPEP_0170096854
Dinobryon_sp._UTEXLB2267 CAMPEP_0170097340
Dinobryon_sp._UTEXLB2267 CAMPEP_0170097024
Dinobryon_sp._UTEXLB2267 CAMPEP_0170111452
Dinobryon_sp._UTEXLB2267 CAMPEP_0201093910
Dinobryon_sp._UTEXLB2267 CAMPEP_0170113352
Pseudopedinella_elastica_CCMP716 CAMPEP_0181259430
Dinobryon_sp._UTEXLB2267 CAMPEP_0170101226
Dinobryon_sp._UTEXLB2267 CAMPEP_0170084516
Dinobryon_sp._UTEXLB2267 CAMPEP_0170090008
Ochromonas_sp._BG-1 CAMPEP_0173144270
Ochromonas_sp._BG-1 CAMPEP_0173149116
Chromulina_nebulosa_UTEXLB2642 CAMPEP_0196761158
Mallomonas_sp._CCMP3275 CAMPEP_0182427170
Mallomonas_sp._CCMP3275 CAMPEP_0182419204
Timspurckia_oligopyrenoides_CCMP3278 CAMPEP_0182441836
Erythrolobus_australicus_CCMP3124 CAMPEP_0185832444
Porphyridium_aerugineum_SAG_1380-2 CAMPEP_0184707224
Porphyridium_aerugineum_SAG_1380-2 CAMPEP_0184691774
Rhodella_maculata_CCMP736 CAMPEP_0174885354
Debaryomyces_hansenii_J26 CAMPEP_0194475210
Pelagomonas_calceolata_CCMP1756 CAMPEP_0195934530
Rhizosolenia_setigera_CCMP_1694 CAMPEP_0178955194
Colletotrichum_gloeosporioides_Cg-14 EQB51166
Ophiostoma_piceae_UAMH_11346 EPE08253
Ophiostoma_novo-ulmi_subsp._novo-ulmi AFV30229
Grosmannia_clavigera_kw1407 XP_014175821
Valsa_mali_var._pyri KUI53218
Phaeoacremonium_minimum_UCRPA7 XP_007911619
Scedosporium_apiospermum XP_016641696
Lomentospora_prolificans PKS04915
Madurella_mycetomatis KXX82756
Chaetomium_globosum_CBS_148.51 XP_001228112
Thielavia_terrestris_NRRL_8126 XP_003657837
Chaetomium_thermophilum_var._thermophilum_DSM_1495 XP_006691002
Neurospora_crassa XP_011393501
Pseudomonas_syringae WP_085741116
Neurospora_tetrasperma_FGSC_2508 XP_009852135
Sordaria_macrospora_k-hell XP_003350771
Coniochaeta_pulveracea RKU46976
Tolypocladium_ophioglossoides_CBS_100239 KND94468
Tolypocladium_paradoxum POR36670
Drechmeria_coniospora KYK54200
Purpureocillium_lilacinum XP_018176983
Pelagococcus_subviridis_CCMP1429 CAMPEP_0195814872
Pelagococcus_subviridis_CCMP1429 CAMPEP_0195792412
Nectria_haematococca_mpVI_77-13-4 XP_003048166
Fusarium_kuroshium RMJ19734
Neonectria_ditissima KPM39130
Aureoumbra_lagunensis_CCMP1510 CAMPEP_0197320040
Verticillium_longisporum CRK44478
Verticillium_dahliae CRK14217
Verticillium_dahliae_VdLs.17 XP_009654707
Verticillium_nonalfalfae RNJ57806
Verticillium_alfalfae_VaMs.102 XP_003004452
Ceratocystis_platani KKF95024
Eutypa_lata_UCREL1 EMR64628
Pseudomassariella_vexata ORY70434
fungal_sp._No.14919 GAW19181
Pestalotiopsis_fici_W106-1 XP_007832558
Curvularia_lunata ABC88428
Curvularia_lunata ABK91799
Exserohilum_turcica_Et28A XP_008025480
Bipolaris_maydis_ATCC_48331 XP_014084526
Bipolaris_oryzae_ATCC_44560 XP_007682918
Bipolaris_zeicola_26-R-13 XP_007717884
Bipolaris_sorokiniana_ND90Pr XP_007694712
Leptosphaeria_maculans_JN3 XP_003837280
Pyrenophora_tritici-repentis XP_001939279
Pyrenophora_teres_f._teres_0-1 EFQ86948
Pyrenophora_seminiperda_CCB06 RMZ72625
Alternaria_alternata XP_018385554
Pyrenochaeta_sp._DS3sAY3a OAL54355
Parastagonospora_nodorum_SN15 XP_001798984
Stagonospora_sp._SRC1lsM3a OAL04138
Zymoseptoria_brevis KJX93836
NonPhotosyntheticDiatom QUERY_NAA37P00170.m1
Alcohol dehydrogenase 1
0.2

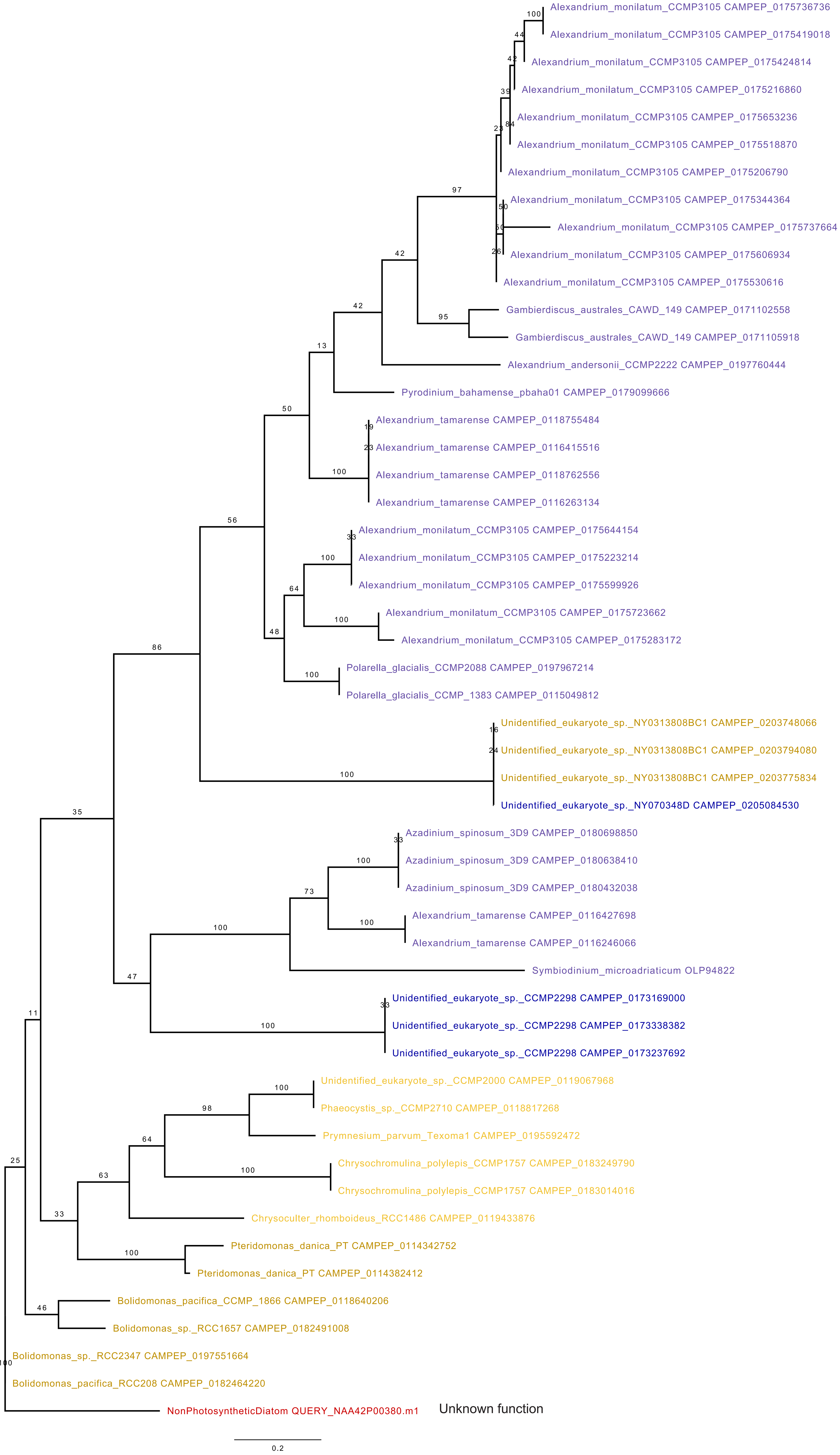
Alexandrium_monilatum_CCMP3105 CAMPEP_0175736736
Alexandrium_monilatum_CCMP3105 CAMPEP_0175419018
Alexandrium_monilatum_CCMP3105 CAMPEP_0175424814
Alexandrium_monilatum_CCMP3105 CAMPEP_0175216860
Alexandrium_monilatum_CCMP3105 CAMPEP_0175653236
Alexandrium_monilatum_CCMP3105 CAMPEP_0175518870
Alexandrium_monilatum_CCMP3105 CAMPEP_0175206790
Alexandrium_monilatum_CCMP3105 CAMPEP_0175344364
Alexandrium_monilatum_CCMP3105 CAMPEP_0175737664
Alexandrium_monilatum_CCMP3105 CAMPEP_0175606934
Alexandrium_monilatum_CCMP3105 CAMPEP_0175530616
Gambierdiscus_australes_CAWD_149 CAMPEP_0171102558
Gambierdiscus_australes_CAWD_149 CAMPEP_0171105918
Alexandrium_andersonii_CCMP2222 CAMPEP_0197760444
Pyrodinium_bahamense_pbaha01 CAMPEP_0179099666
Alexandrium_tamarense CAMPEP_0118755484
Alexandrium_tamarense CAMPEP_0116415516
Alexandrium_tamarense CAMPEP_0118762556
Alexandrium_tamarense CAMPEP_0116263134
Alexandrium_monilatum_CCMP3105 CAMPEP_0175644154
Alexandrium_monilatum_CCMP3105 CAMPEP_0175223214
Alexandrium_monilatum_CCMP3105 CAMPEP_0175599926
Alexandrium_monilatum_CCMP3105 CAMPEP_0175723662
Alexandrium_monilatum_CCMP3105 CAMPEP_0175283172
Polarella_glacialis_CCMP2088 CAMPEP_0197967214
Polarella_glacialis_CCMP_1383 CAMPEP_0115049812
Unidentified_eukaryote_sp._NY0313808BC1 CAMPEP_0203748066
Unidentified_eukaryote_sp._NY0313808BC1 CAMPEP_0203794080
Unidentified_eukaryote_sp._NY0313808BC1 CAMPEP_0203775834
Unidentified_eukaryote_sp._NY070348D CAMPEP_0205084530
Azadinium_spinosum_3D9 CAMPEP_0180698850
Azadinium_spinosum_3D9 CAMPEP_0180638410
Azadinium_spinosum_3D9 CAMPEP_0180432038
Alexandrium_tamarense CAMPEP_0116427698
Alexandrium_tamarense CAMPEP_0116246066
Symbiodinium_microadriaticum OLP94822
Unidentified_eukaryote_sp._CCMP2298 CAMPEP_0173169000
Unidentified_eukaryote_sp._CCMP2298 CAMPEP_0173338382
Unidentified_eukaryote_sp._CCMP2298 CAMPEP_0173237692
Unidentified_eukaryote_sp._CCMP2000 CAMPEP_0119067968
Phaeocystis_sp._CCMP2710 CAMPEP_0118817268
Prymnesium_parvum_Texoma1 CAMPEP_0195592472
Chrysochromulina_polylepis_CCMP1757 CAMPEP_0183249790
Chrysochromulina_polylepis_CCMP1757 CAMPEP_0183014016
Chrysoculter_rhomboideus_RCC1486 CAMPEP_0119433876
Pteridomonas_danica_PT CAMPEP_0114342752
Pteridomonas_danica_PT CAMPEP_0114382412
Bolidomonas_pacifica_CCMP_1866 CAMPEP_0118640206
Bolidomonas_sp._RCC1657 CAMPEP_0182491008
Bolidomonas_sp._RCC2347 CAMPEP_0197551664
Bolidomonas_pacifica_RCC208 CAMPEP_0182464220
NonPhotosyntheticDiatom QUERY_NAA42P00380.m1
Unknown function
0.2

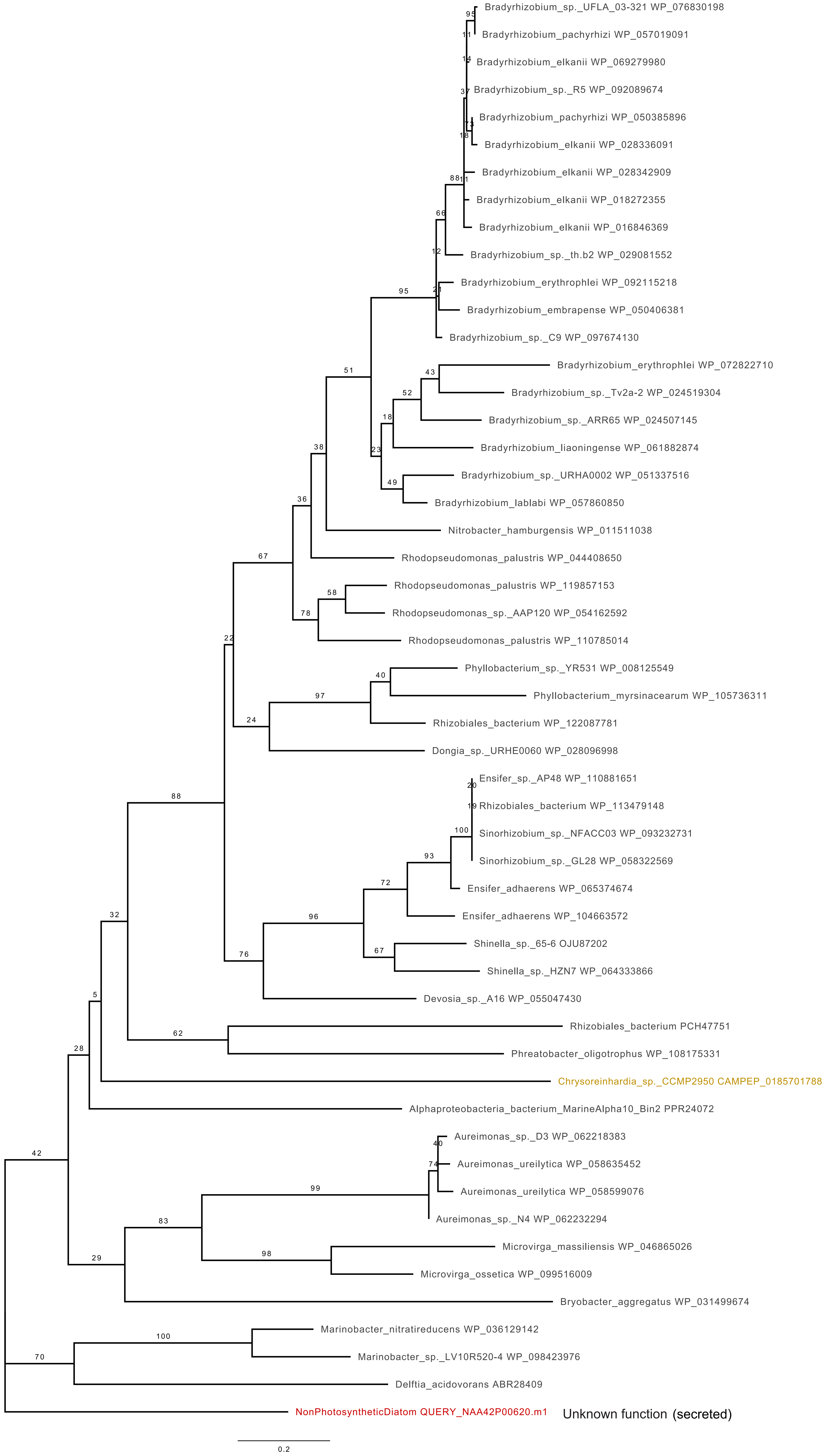
Bradyrhizobium_sp._UFLA_03-321 WP_076830198
Bradyrhizobium_pachyrhizi WP_057019091
Bradyrhizobium_elkanii WP_069279980
Bradyrhizobium_sp._R5 WP_092089674
Bradyrhizobium_pachyrhizi WP_050385896
Bradyrhizobium_elkanii WP_028336091
Bradyrhizobium_elkanii WP_028342909
Bradyrhizobium_elkanii WP_018272355
Bradyrhizobium_elkanii WP_016846369
Bradyrhizobium_sp._th.b2 WP_029081552
Bradyrhizobium_erythrophlei WP_092115218
Bradyrhizobium_embrapense WP_050406381
Bradyrhizobium_sp._C9 WP_097674130
Bradyrhizobium_erythrophlei WP_072822710
Bradyrhizobium_sp._Tv2a-2 WP_024519304
Bradyrhizobium_sp._ARR65 WP_024507145
Bradyrhizobium_liaoningense WP_061882874
Bradyrhizobium_sp._URHA0002 WP_051337516
Bradyrhizobium_lablabi WP_057860850
Nitrobacter_hamburgensis WP_011511038
Rhodopseudomonas_palustris WP_044408650
Rhodopseudomonas_palustris WP_119857153
Rhodopseudomonas_sp._AAP120 WP_054162592
Rhodopseudomonas_palustris WP_110785014
Phyllobacterium_sp._YR531 WP_008125549
Phyllobacterium_myrsinacearum WP_105736311
Rhizobiales_bacterium WP_122087781
Dongia_sp._URHE0060 WP_028096998
Ensifer_sp._AP48 WP_110881651
Rhizobiales_bacterium WP_113479148
Sinorhizobium_sp._NFACC03 WP_093232731
Sinorhizobium_sp._GL28 WP_058322569
Ensifer_adhaerens WP_065374674
Ensifer_adhaerens WP_104663572
Shinella_sp._65-6 OJU87202
Shinella_sp._HZN7 WP_064333866
Devosia_sp._A16 WP_055047430
Rhizobiales_bacterium PCH47751
Phreatobacter_oligotrophus WP_108175331
Chrysoreinhardia_sp._CCMP2950 CAMPEP_0185701788
Alphaproteobacteria_bacterium_MarineAlpha10_Bin2 PPR24072
Aureimonas_sp._D3 WP_062218383
Aureimonas_ureilytica WP_058635452
Aureimonas_ureilytica WP_058599076
Aureimonas_sp._N4 WP_062232294
Microvirga_massiliensis WP_046865026
Microvirga_ossetica WP_099516009
Bryobacter_aggregatus WP_031499674
Marinobacter_nitratireducens WP_036129142
Marinobacter_sp._LV10R520-4 WP_098423976
Delftia_acidovorans ABR28409
NonPhotosyntheticDiatom QUERY_NAA42P00620.m1
Unknown function (secreted)
0.2

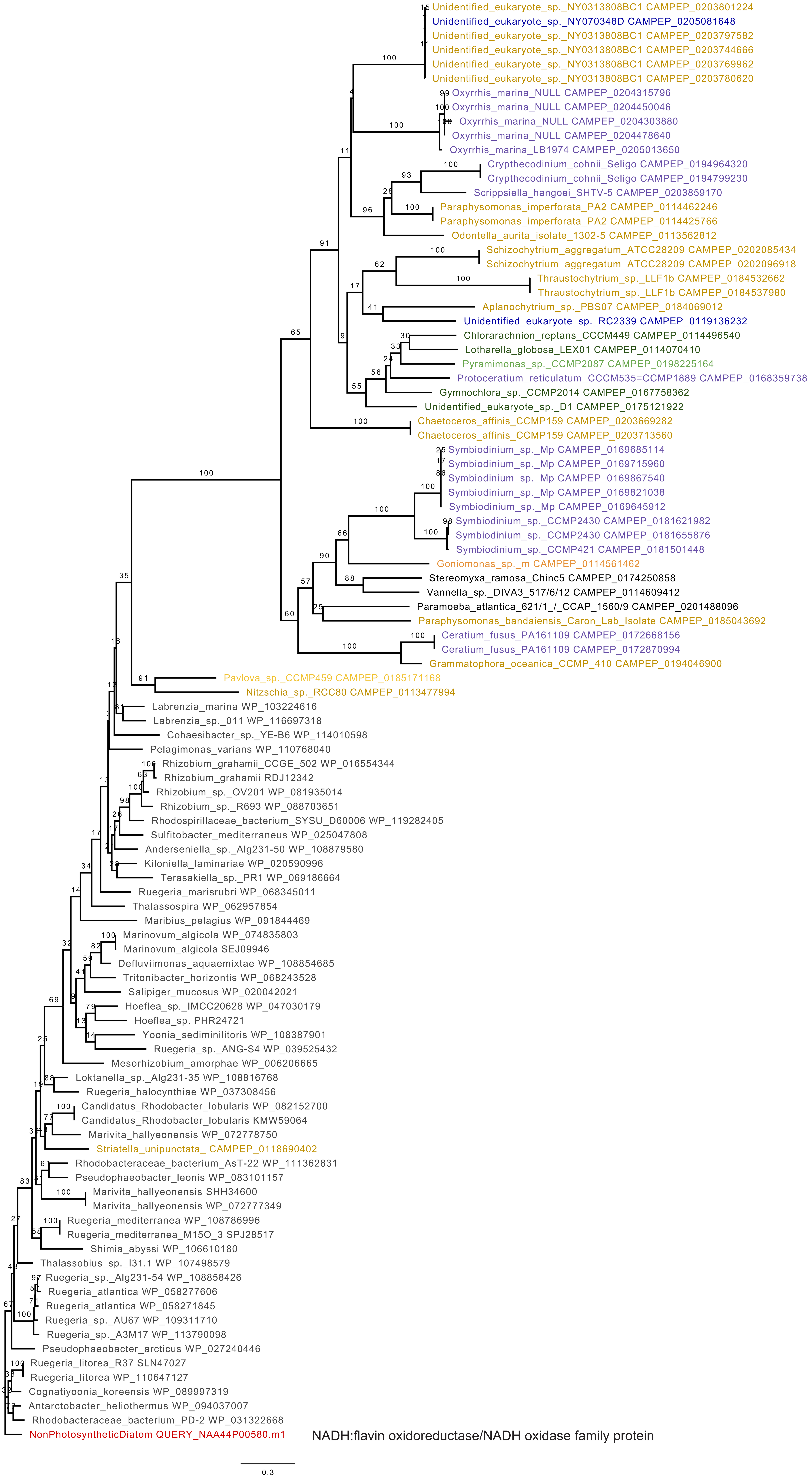
Unidentified_eukaryote_sp._NY0313808BC1 CAMPEP_0203801224
Unidentified_eukaryote_sp._NY070348D CAMPEP_0205081648
Unidentified_eukaryote_sp._NY0313808BC1 CAMPEP_0203797582
Unidentified_eukaryote_sp._NY0313808BC1 CAMPEP_0203744666
Unidentified_eukaryote_sp._NY0313808BC1 CAMPEP_0203769962
Unidentified_eukaryote_sp._NY0313808BC1 CAMPEP_0203780620
Oxyrrhis_marina_NULL CAMPEP_0204315796
Oxyrrhis_marina_NULL CAMPEP_0204450046
Oxyrrhis_marina_NULL CAMPEP_0204303880
Oxyrrhis_marina_NULL CAMPEP_0204478640
Oxyrrhis_marina_LB1974 CAMPEP_0205013650
Crypthecodinium_cohnii_Seligo CAMPEP_0194964320
Crypthecodinium_cohnii_Seligo CAMPEP_0194799230
Scrippsiella_hangoei_SHTV-5 CAMPEP_0203859170
Paraphysomonas_imperforata_PA2 CAMPEP_0114462246
Paraphysomonas_imperforata_PA2 CAMPEP_0114425766
Odontella_aurita_isolate_1302-5 CAMPEP_0113562812
Schizochytrium_aggregatum_ATCC28209 CAMPEP_0202085434
Schizochytrium_aggregatum_ATCC28209 CAMPEP_0202096918
Thraustochytrium_sp._LLF1b CAMPEP_0184532662
Thraustochytrium_sp._LLF1b CAMPEP_0184537980
Aplanochytrium_sp._PBS07 CAMPEP_0184069012
Unidentified_eukaryote_sp._RC2339 CAMPEP_0119136232
Chlorarachnion_reptans_CCCM449 CAMPEP_0114496540
Lotharella_globosa_LEX01 CAMPEP_0114070410
Pyramimonas_sp._CCMP2087 CAMPEP_0198225164
Protoceratium_reticulatum_CCCM535=CCMP1889 CAMPEP_0168359738
Gymnochlora_sp._CCMP2014 CAMPEP_0167758362
Unidentified_eukaryote_sp._D1 CAMPEP_0175121922
Chaetoceros_affinis_CCMP159 CAMPEP_0203669282
Chaetoceros_affinis_CCMP159 CAMPEP_0203713560
Symbiodinium_sp._Mp CAMPEP_0169685114
Symbiodinium_sp._Mp CAMPEP_0169715960
Symbiodinium_sp._Mp CAMPEP_0169867540
Symbiodinium_sp._Mp CAMPEP_0169821038
Symbiodinium_sp._Mp CAMPEP_0169645912
Symbiodinium_sp._CCMP2430 CAMPEP_0181621982
Symbiodinium_sp._CCMP2430 CAMPEP_0181655876
Symbiodinium_sp._CCMP421 CAMPEP_0181501448
Goniomonas_sp._m CAMPEP_0114561462
Stereomyxa_ramosa_Chinc5 CAMPEP_0174250858
Vannella_sp._DIVA3_517/6/12 CAMPEP_0114609412
Paramoeba_atlantica_621/1_/_CCAP_1560/9 CAMPEP_0201488096
Paraphysomonas_bandaiensis_Caron_Lab_Isolate CAMPEP_0185043692
Ceratium_fusus_PA161109 CAMPEP_0172668156
Ceratium_fusus_PA161109 CAMPEP_0172870994
Grammatophora_oceanica_CCMP_410 CAMPEP_0194046900
Pavlova_sp._CCMP459 CAMPEP_0185171168
Nitzschia_sp._RCC80 CAMPEP_0113477994
Labrenzia_marina WP_103224616
Labrenzia_sp._011 WP_116697318
Cohaesibacter_sp._YE-B6 WP_114010598
Pelagimonas_varians WP_110768040
Rhizobium_grahamii_CCGE_502 WP_016554344
Rhizobium_grahamii RDJ12342
Rhizobium_sp._OV201 WP_081935014
Rhizobium_sp._R693 WP_088703651
Rhodospirillaceae_bacterium_SYSU_D60006 WP_119282405
Sulfitobacter_mediterraneus WP_025047808
Anderseniella_sp._Alg231-50 WP_108879580
Kiloniella_laminariae WP_020590996
Terasakiella_sp._PR1 WP_069186664
Ruegeria_marisrubri WP_068345011
Thalassospira WP_062957854
Maribius_pelagius WP_091844469
Marinovum_algicola WP_074835803
Marinovum_algicola SEJ09946
Defluviimonas_aquaemixtae WP_108854685
Tritonibacter_horizontis WP_068243528
Salipiger_mucosus WP_020042021
Hoeflea_sp._IMCC20628 WP_047030179
Hoeflea_sp. PHR24721
Yoonia_sediminilitoris WP_108387901
Ruegeria_sp._ANG-S4 WP_039525432
Mesorhizobium_amorphae WP_006206665
Loktanella_sp._Alg231-35 WP_108816768
Ruegeria_halocynthiae WP_037308456
Candidatus_Rhodobacter_lobularis WP_082152700
Candidatus_Rhodobacter_lobularis KMW59064
Marivita_hallyeonensis WP_072778750
Striatella_unipunctata_ CAMPEP_0118690402
Rhodobacteraceae_bacterium_AsT-22 WP_111362831
Pseudophaeobacter_leonis WP_083101157
Marivita_hallyeonensis SHH34600
Marivita_hallyeonensis WP_072777349
Ruegeria_mediterranea WP_108786996
Ruegeria_mediterranea_M15O_3 SPJ28517
Shimia_abyssi WP_106610180
Thalassobius_sp._I31.1 WP_107498579
Ruegeria_sp._Alg231-54 WP_108858426
Ruegeria_atlantica WP_058277606
Ruegeria_atlantica WP_058271845
Ruegeria_sp._AU67 WP_109311710
Ruegeria_sp._A3M17 WP_113790098
Pseudophaeobacter_arcticus WP_027240446
Ruegeria_litorea_R37 SLN47027
Ruegeria_litorea WP_110647127
Cognatiyoonia_koreensis WP_089997319
Antarctobacter_heliothermus WP_094037007
Rhodobacteraceae_bacterium_PD-2 WP_031322668
NonPhotosyntheticDiatom QUERY_NAA44P00580.m1
NADH:flavin oxidoreductase/NADH oxidase family protein
0.3

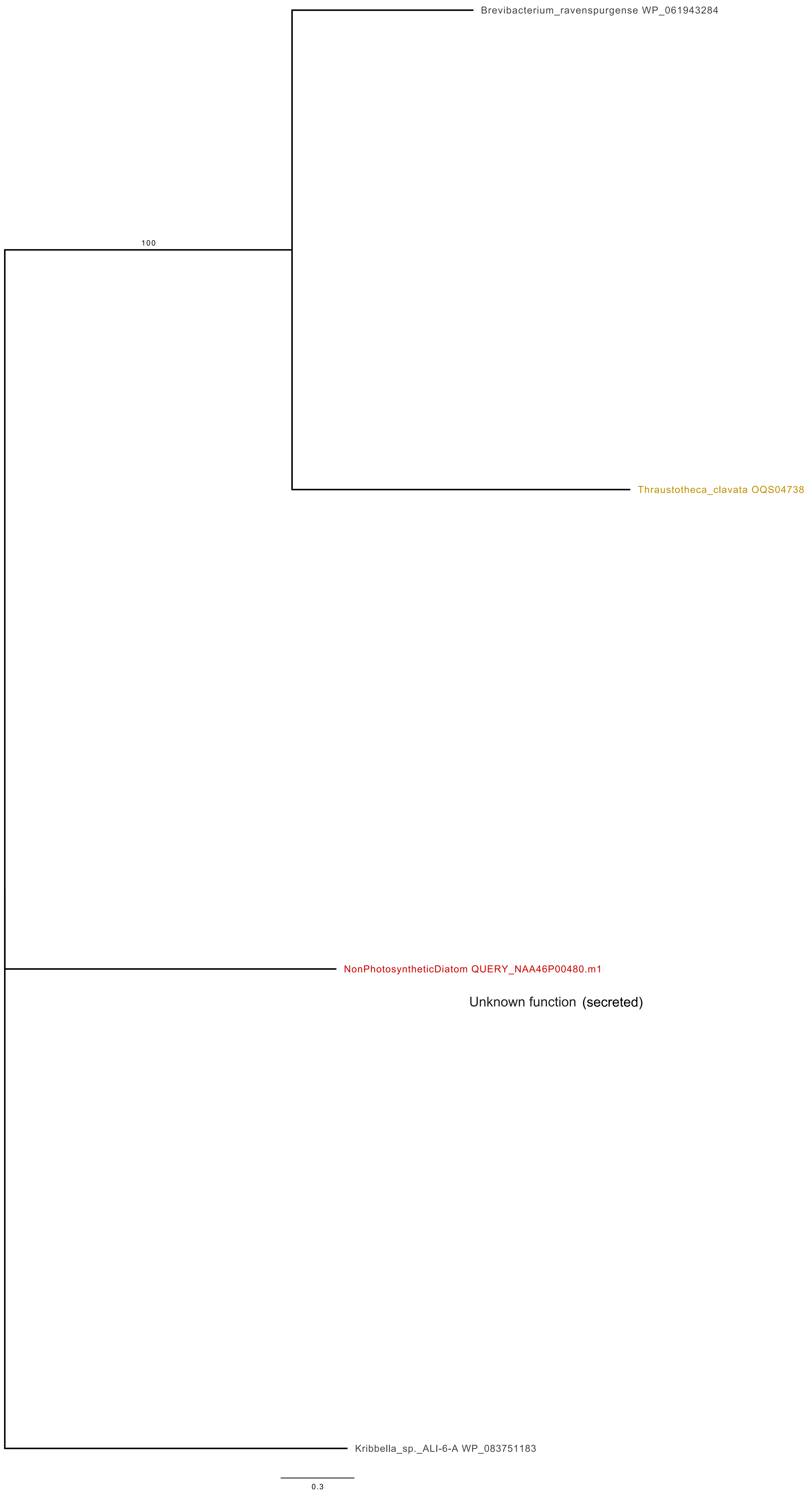
Brevibacterium_ravenspurgense WP_061943284
100
Thraustotheca_clavata OQS04738
NonPhotosyntheticDiatom QUERY_NAA46P00480.m1
Unknown function (secreted)
Kribbella_sp._ALI-6-A WP_083751183
0.3

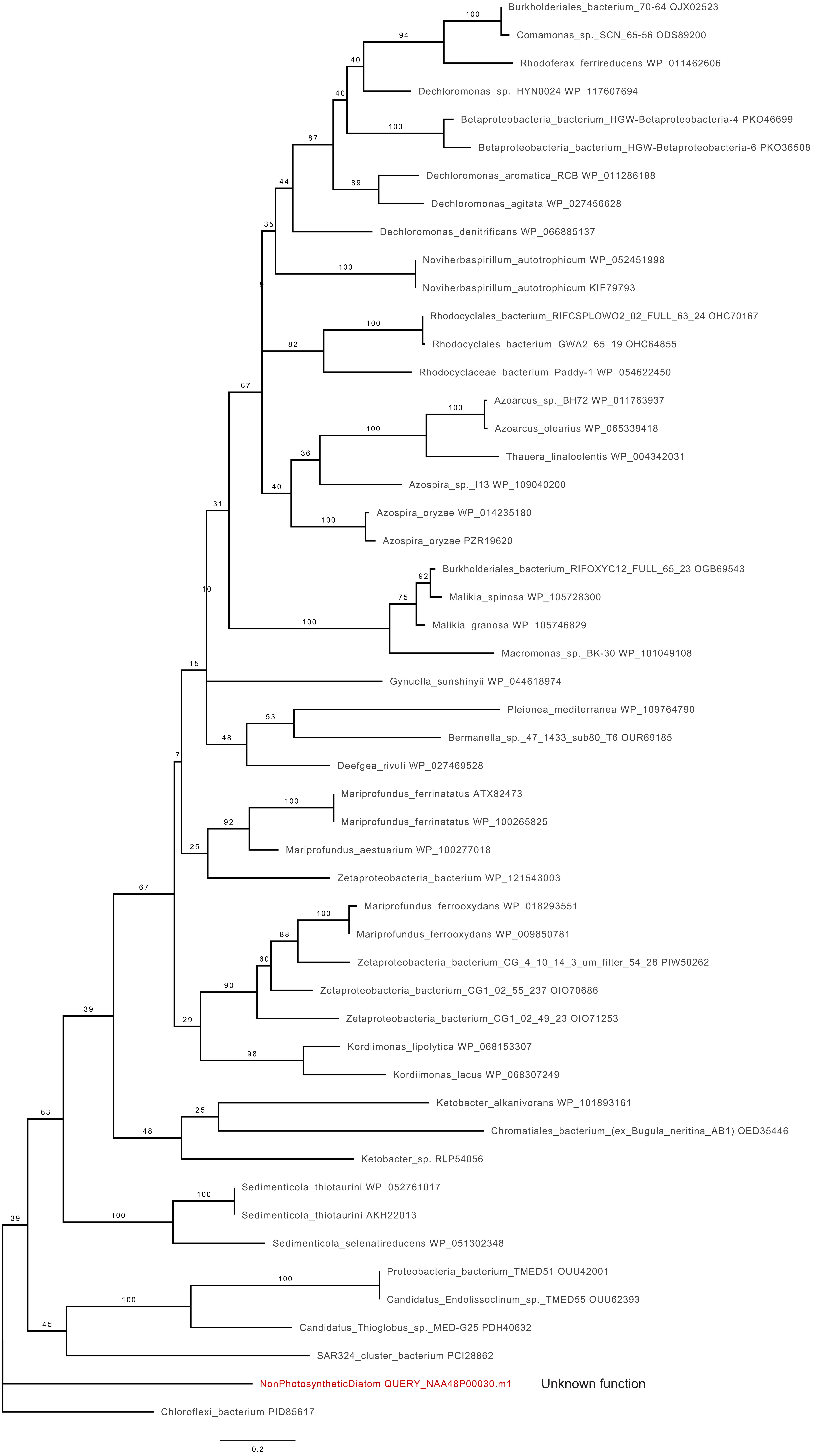
Burkholderiales_bacterium_70-64 OJX02523
Comamonas_sp._SCN_65-56 ODS89200
Rhodoferax_ferrireducens WP_011462606
Dechloromonas_sp._HYN0024 WP_117607694
Betaproteobacteria_bacterium_HGW-Betaproteobacteria-4 PKO46699
Betaproteobacteria_bacterium_HGW-Betaproteobacteria-6 PKO36508
Dechloromonas_aromatica_RCB WP_011286188
Dechloromonas_agitata WP_027456628
Dechloromonas_denitrificans WP_066885137
Noviherbaspirillum_autotrophicum WP_052451998
Noviherbaspirillum_autotrophicum KIF79793
Rhodocyclales_bacterium_RIFCSPLOWO2_02_FULL_63_24 OHC70167
Rhodocyclales_bacterium_GWA2_65_19 OHC64855
Rhodocyclaceae_bacterium_Paddy-1 WP_054622450
Azoarcus_sp._BH72 WP_011763937
Azoarcus_olearius WP_065339418
Thauera_linaloolentis WP_004342031
Azospira_sp._I13 WP_109040200
Azospira_oryzae WP_014235180
Azospira_oryzae PZR19620
Burkholderiales_bacterium_RIFOXYC12_FULL_65_23 OGB69543
Malikia_spinosa WP_105728300
Malikia_granosa WP_105746829
Macromonas_sp._BK-30 WP_101049108
Gynuella_sunshinyii WP_044618974
Pleionea_mediterranea WP_109764790
Bermanella_sp._47_1433_sub80_T6 OUR69185
Deefgea_rivuli WP_027469528
Mariprofundus_ferrinatatus ATX82473
Mariprofundus_ferrinatatus WP_100265825
Mariprofundus_aestuarium WP_100277018
Zetaproteobacteria_bacterium WP_121543003
Mariprofundus_ferrooxydans WP_018293551
Mariprofundus_ferrooxydans WP_009850781
Zetaproteobacteria_bacterium_CG_4_10_14_3_um_filter_54_28 PIW50262
Zetaproteobacteria_bacterium_CG1_02_55_237 OIO70686
Zetaproteobacteria_bacterium_CG1_02_49_23 OIO71253
Kordiimonas_lipolytica WP_068153307
Kordiimonas_lacus WP_068307249
Ketobacter_alkanivorans WP_101893161
Chromatiales_bacterium_(ex_Bugula_neritina_AB1) OED35446
Ketobacter_sp. RLP54056
Sedimenticola_thiotaurini WP_052761017
Sedimenticola_thiotaurini AKH22013
Sedimenticola_selenatireducens WP_051302348
Proteobacteria_bacterium_TMED51 OUU42001
Candidatus_Endolissoclinum_sp._TMED55 OUU62393
Candidatus_Thioglobus_sp._MED-G25 PDH40632
SAR324_cluster_bacterium PCI28862
NonPhotosyntheticDiatom QUERY_NAA48P00030.m1
Unknown function
Chloroflexi_bacterium PID85617
0.2

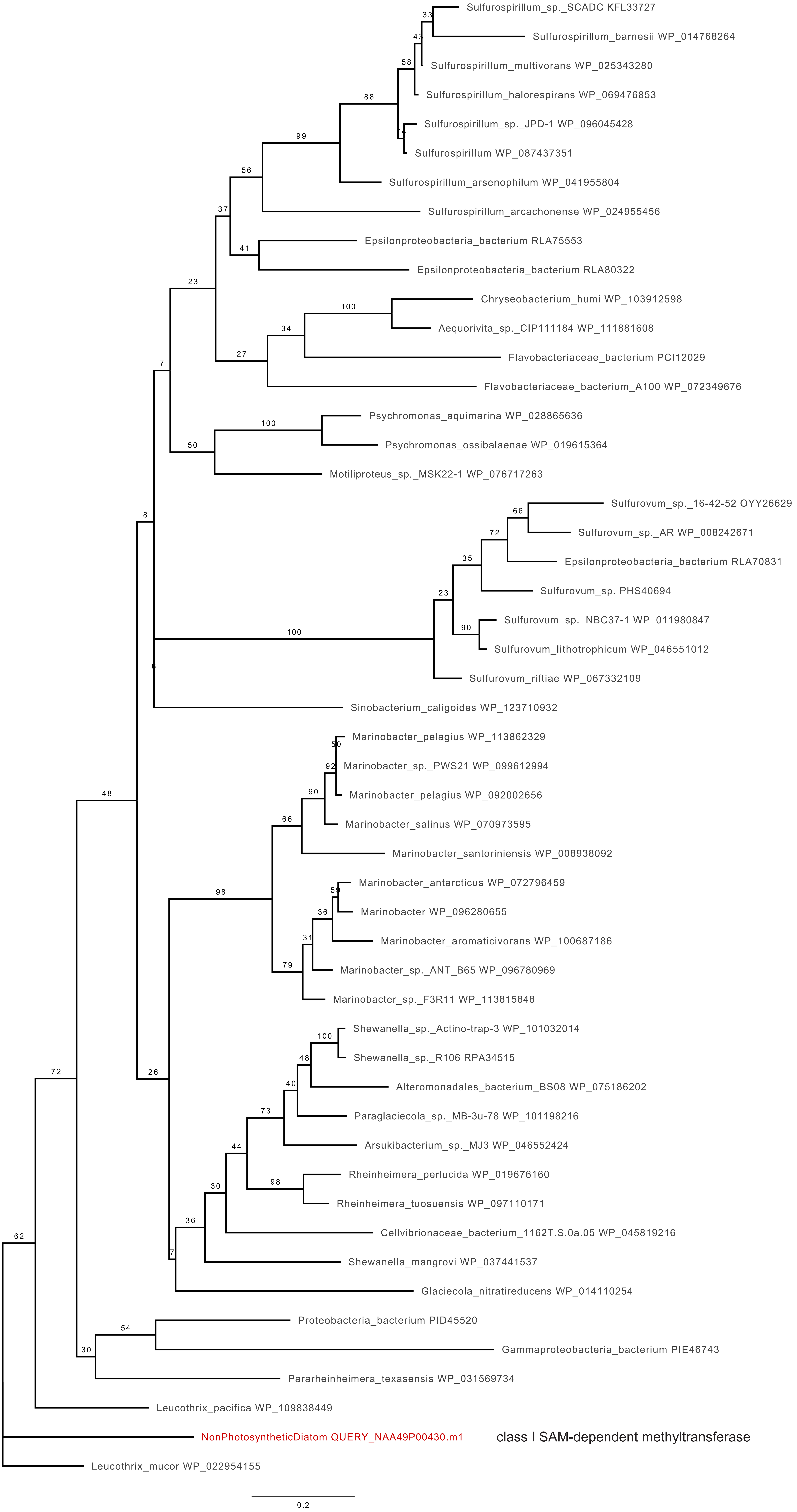
Sulfurospirillum_sp._SCADC KFL33727
Sulfurospirillum_barnesii WP_014768264
Sulfurospirillum_multivorans WP_025343280
Sulfurospirillum_halorespirans WP_069476853
Sulfurospirillum_sp._JPD-1 WP_096045428
Sulfurospirillum WP_087437351
Sulfurospirillum_arsenophilum WP_041955804
Sulfurospirillum_arcachonense WP_024955456
Epsilonproteobacteria_bacterium RLA75553
Epsilonproteobacteria_bacterium RLA80322
Chryseobacterium_humi WP_103912598
Aequorivita_sp._CIP111184 WP_111881608
Flavobacteriaceae_bacterium PCI12029
Flavobacteriaceae_bacterium_A100 WP_072349676
Psychromonas_aquimarina WP_028865636
Psychromonas_ossibalaenae WP_019615364
Motiliproteus_sp._MSK22-1 WP_076717263
Sulfurovum_sp._16-42-52 OYY26629
Sulfurovum_sp._AR WP_008242671
Epsilonproteobacteria_bacterium RLA70831
Sulfurovum_sp. PHS40694
Sulfurovum_sp._NBC37-1 WP_011980847
Sulfurovum_lithotrophicum WP_046551012
Sulfurovum_riftiae WP_067332109
Sinobacterium_caligoides WP_123710932
Marinobacter_pelagius WP_113862329
Marinobacter_sp._PWS21 WP_099612994
Marinobacter_pelagius WP_092002656
Marinobacter_salinus WP_070973595
Marinobacter_santoriniensis WP_008938092
Marinobacter_antarcticus WP_072796459
Marinobacter WP_096280655
Marinobacter_aromaticivorans WP_100687186
Marinobacter_sp._ANT_B65 WP_096780969
Marinobacter_sp._F3R11 WP_113815848
Shewanella_sp._Actino-trap-3 WP_101032014
Shewanella_sp._R106 RPA34515
Alteromonadales_bacterium_BS08 WP_075186202
Paraglaciecola_sp._MB-3u-78 WP_101198216
Arsukibacterium_sp._MJ3 WP_046552424
Rheinheimera_perlucida WP_019676160
Rheinheimera_tuosuensis WP_097110171
Cellvibrionaceae_bacterium_1162T.S.0a.05 WP_045819216
Shewanella_mangrovi WP_037441537
Glaciecola_nitratireducens WP_014110254
Proteobacteria_bacterium PID45520
Gammaproteobacteria_bacterium PIE46743
Pararheinheimera_texasensis WP_031569734
Leucothrix_pacifica WP_109838449
NonPhotosyntheticDiatom QUERY_NAA49P00430.m1
class I SAM-dependent methyltransferase
Leucothrix_mucor WP_022954155
0.2

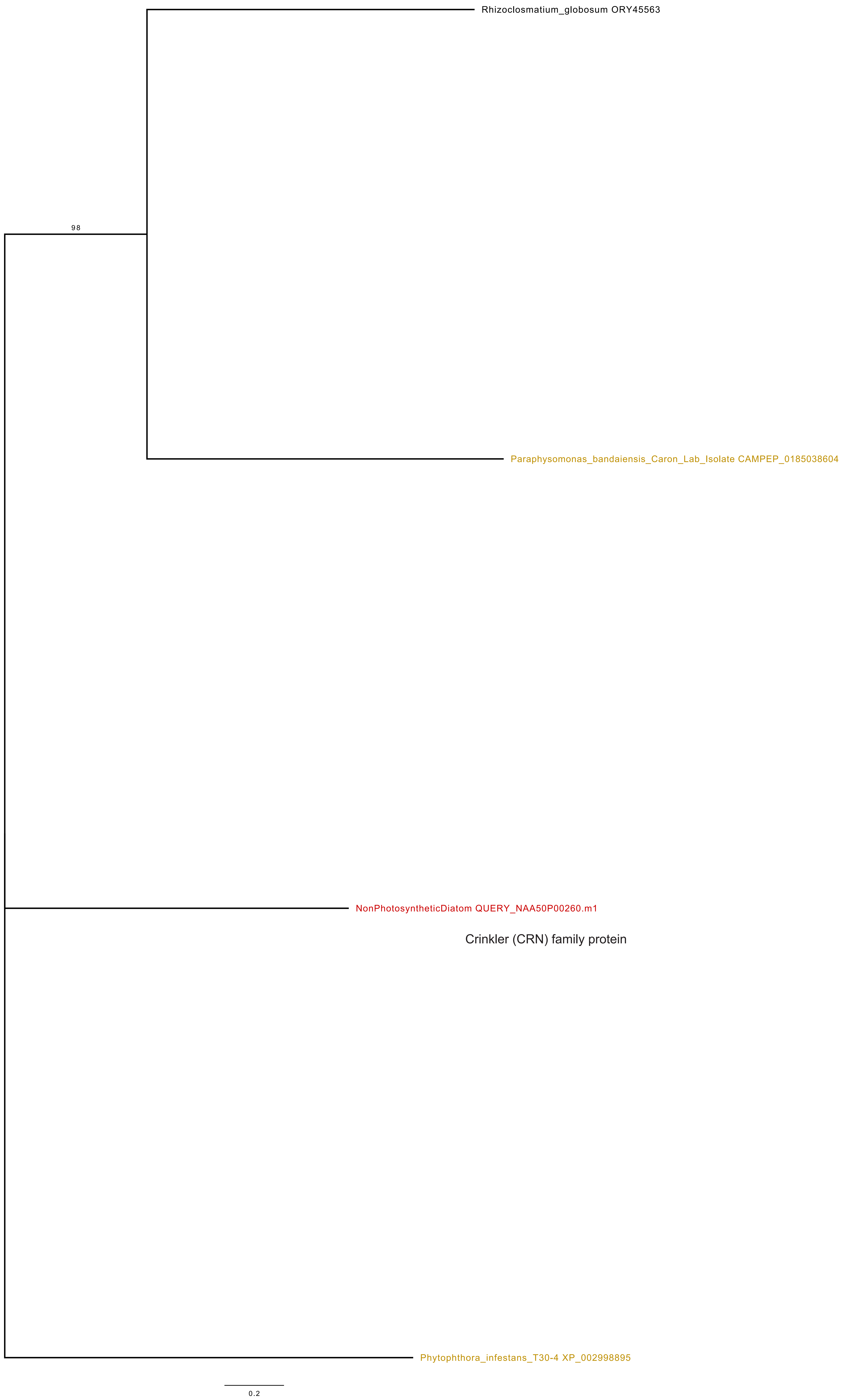
Rhizoclosmatium_globosum ORY45563
98
Paraphysomonas_bandaiensis_Caron_Lab_Isolate CAMPEP_0185038604
NonPhotosyntheticDiatom QUERY_NAA50P00260.m1
Crinkler (CRN) family protein
Phytophthora_infestans_T30-4 XP_002998895
0.2

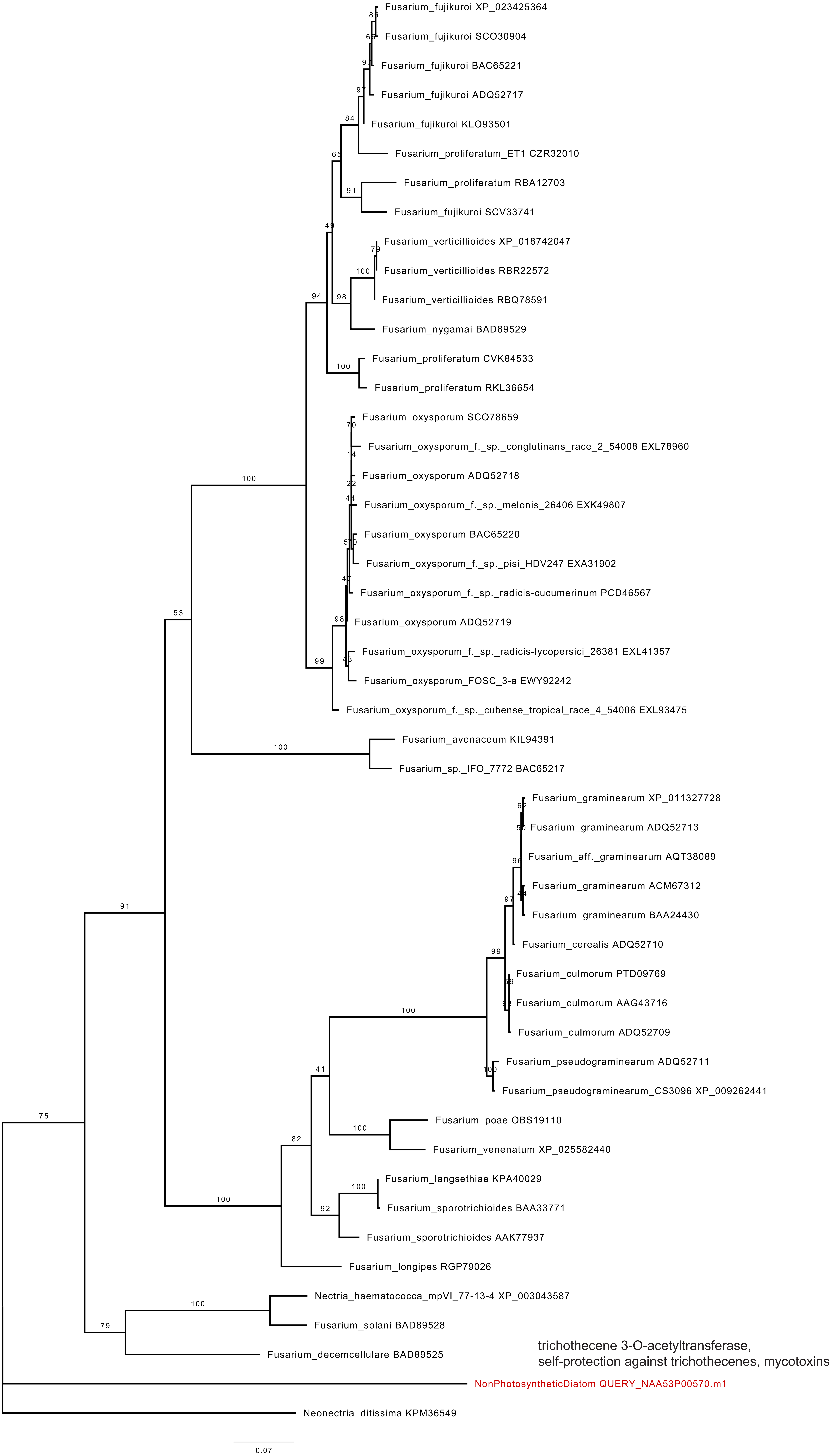
Fusarium_fujikuroi XP_023425364
Fusarium_fujikuroi SCO30904
Fusarium_fujikuroi BAC65221
Fusarium_fujikuroi ADQ52717
Fusarium_fujikuroi KLO93501
Fusarium_proliferatum_ET1 CZR32010
Fusarium_proliferatum RBA12703
Fusarium_fujikuroi SCV33741
Fusarium_verticillioides XP_018742047
Fusarium_verticillioides RBR22572
Fusarium_verticillioides RBQ78591
Fusarium_nygamai BAD89529
Fusarium_proliferatum CVK84533
Fusarium_proliferatum RKL36654
Fusarium_oxysporum SCO78659
Fusarium_oxysporum_f._sp._conglutinans_race_2_54008 EXL78960
Fusarium_oxysporum ADQ52718
Fusarium_oxysporum_f._sp._melonis_26406 EXK49807
Fusarium_oxysporum BAC65220
Fusarium_oxysporum_f._sp._pisi_HDV247 EXA31902
Fusarium_oxysporum_f._sp._radicis-cucumerinum PCD46567
Fusarium_oxysporum ADQ52719
Fusarium_oxysporum_f._sp._radicis-lycopersici_26381 EXL41357
Fusarium_oxysporum_FOSC_3-a EWY92242
Fusarium_oxysporum_f._sp._cubense_tropical_race_4_54006 EXL93475
Fusarium_avenaceum KIL94391
Fusarium_sp._IFO_7772 BAC65217
Fusarium_graminearum XP_011327728
Fusarium_graminearum ADQ52713
Fusarium_aff._graminearum AQT38089
Fusarium_graminearum ACM67312
Fusarium_graminearum BAA24430
Fusarium_cerealis ADQ52710
Fusarium_culmorum PTD09769
Fusarium_culmorum AAG43716
Fusarium_culmorum ADQ52709
Fusarium_pseudograminearum ADQ52711
Fusarium_pseudograminearum_CS3096 XP_009262441
Fusarium_poae OBS19110
Fusarium_venenatum XP_025582440
Fusarium_langsethiae KPA40029
Fusarium_sporotrichioides BAA33771
Fusarium_sporotrichioides AAK77937
Fusarium_longipes RGP79026
Nectria_haematococca_mpVI_77-13-4 XP_003043587
Fusarium_solani BAD89528
Fusarium_decemcellulare BAD89525
trichothecene 3-O-acetyltransferase,
self-protection against trichothecenes, mycotoxins
NonPhotosyntheticDiatom QUERY_NAA53P00570.m1
Neonectria_ditissima KPM36549
0.07

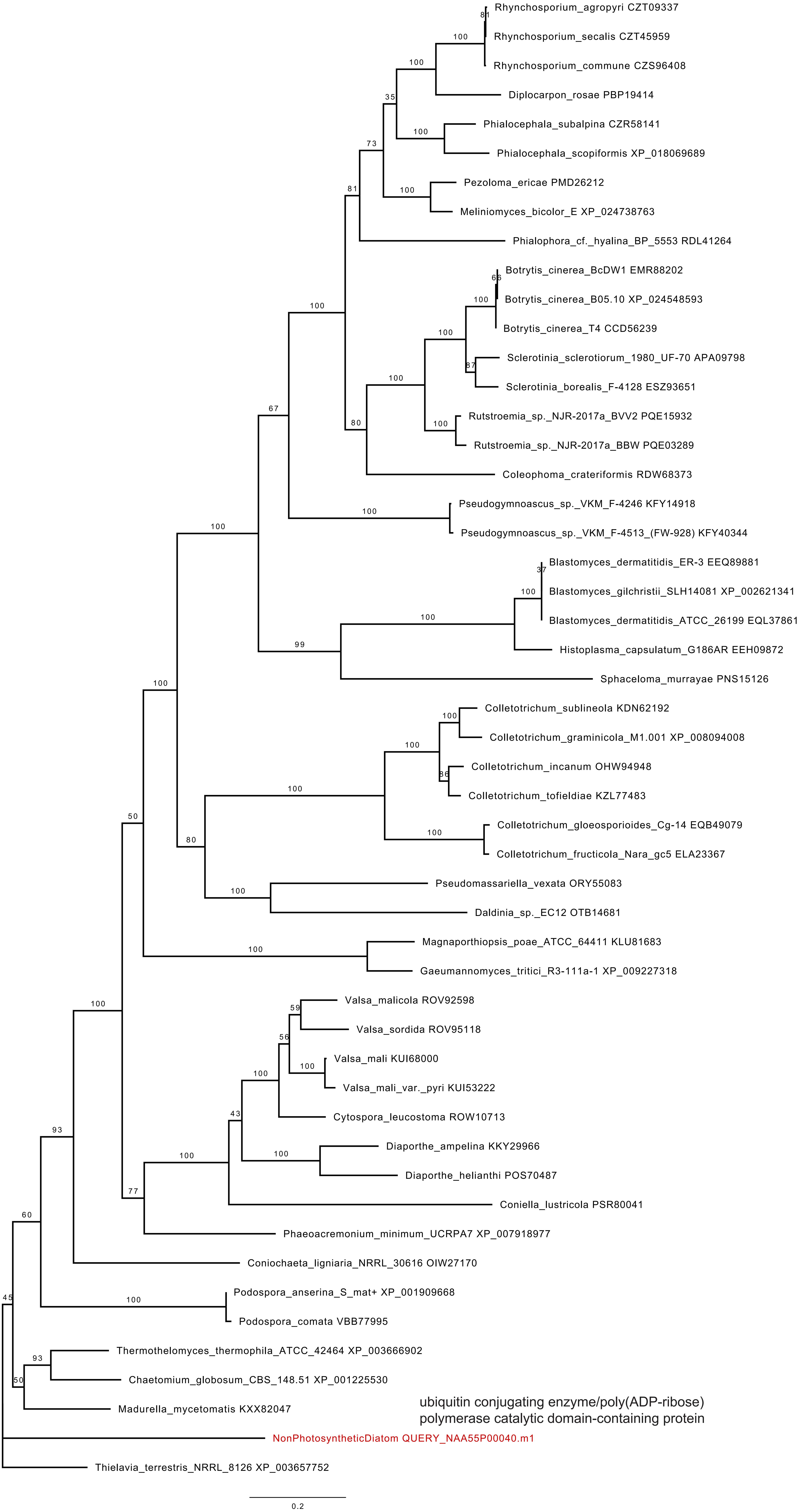
Rhynchosporium_agropyri CZT09337
Rhynchosporium_secalis CZT45959
Rhynchosporium_commune CZS96408
Diplocarpon_rosae PBP19414
Phialocephala_subalpina CZR58141
Phialocephala_scopiformis XP_018069689
Pezoloma_ericae PMD26212
Meliniomyces_bicolor_E XP_024738763
Phialophora_cf._hyalina_BP_5553 RDL41264
Botrytis_cinerea_BcDW1 EMR88202
Botrytis_cinerea_B05.10 XP_024548593
Botrytis_cinerea_T4 CCD56239
Sclerotinia_sclerotiorum_1980_UF-70 APA09798
Sclerotinia_borealis_F-4128 ESZ93651
Rutstroemia_sp._NJR-2017a_BVV2 PQE15932
Rutstroemia_sp._NJR-2017a_BBW PQE03289
Coleophoma_crateriformis RDW68373
Pseudogymnoascus_sp._VKM_F-4246 KFY14918
Pseudogymnoascus_sp._VKM_F-4513_(FW-928) KFY40344
Blastomyces_dermatitidis_ER-3 EEQ89881
Blastomyces_gilchristii_SLH14081 XP_002621341
Blastomyces_dermatitidis_ATCC_26199 EQL37861
Histoplasma_capsulatum_G186AR EEH09872
Sphaceloma_murrayae PNS15126
Colletotrichum_sublineola KDN62192
Colletotrichum_graminicola_M1.001 XP_008094008
Colletotrichum_incanum OHW94948
Colletotrichum_tofieldiae KZL77483
Colletotrichum_gloeosporioides_Cg-14 EQB49079
Colletotrichum_fructicola_Nara_gc5 ELA23367
Pseudomassariella_vexata ORY55083
Daldinia_sp._EC12 OTB14681
Magnaporthiopsis_poae_ATCC_64411 KLU81683
Gaeumannomyces_tritici_R3-111a-1 XP_009227318
Valsa_malicola ROV92598
Valsa_sordida ROV95118
Valsa_mali KUI68000
Valsa_mali_var._pyri KUI53222
Cytospora_leucostoma ROW10713
Diaporthe_ampelina KKY29966
Diaporthe_helianthi POS70487
Coniella_lustricola PSR80041
Phaeoacremonium_minimum_UCRPA7 XP_007918977
Coniochaeta_ligniaria_NRRL_30616 OIW27170
Podospora_anserina_S_mat+ XP_001909668
Podospora_comata VBB77995
Thermothelomyces_thermophila_ATCC_42464 XP_003666902
Chaetomium_globosum_CBS_148.51 XP_001225530
Madurella_mycetomatis KXX82047
ubiquitin conjugating enzyme/poly(ADP-ribose)
polymerase catalytic domain-containing protein
NonPhotosyntheticDiatom QUERY_NAA55P00040.m1
Thielavia_terrestris_NRRL_8126 XP_003657752
0.2

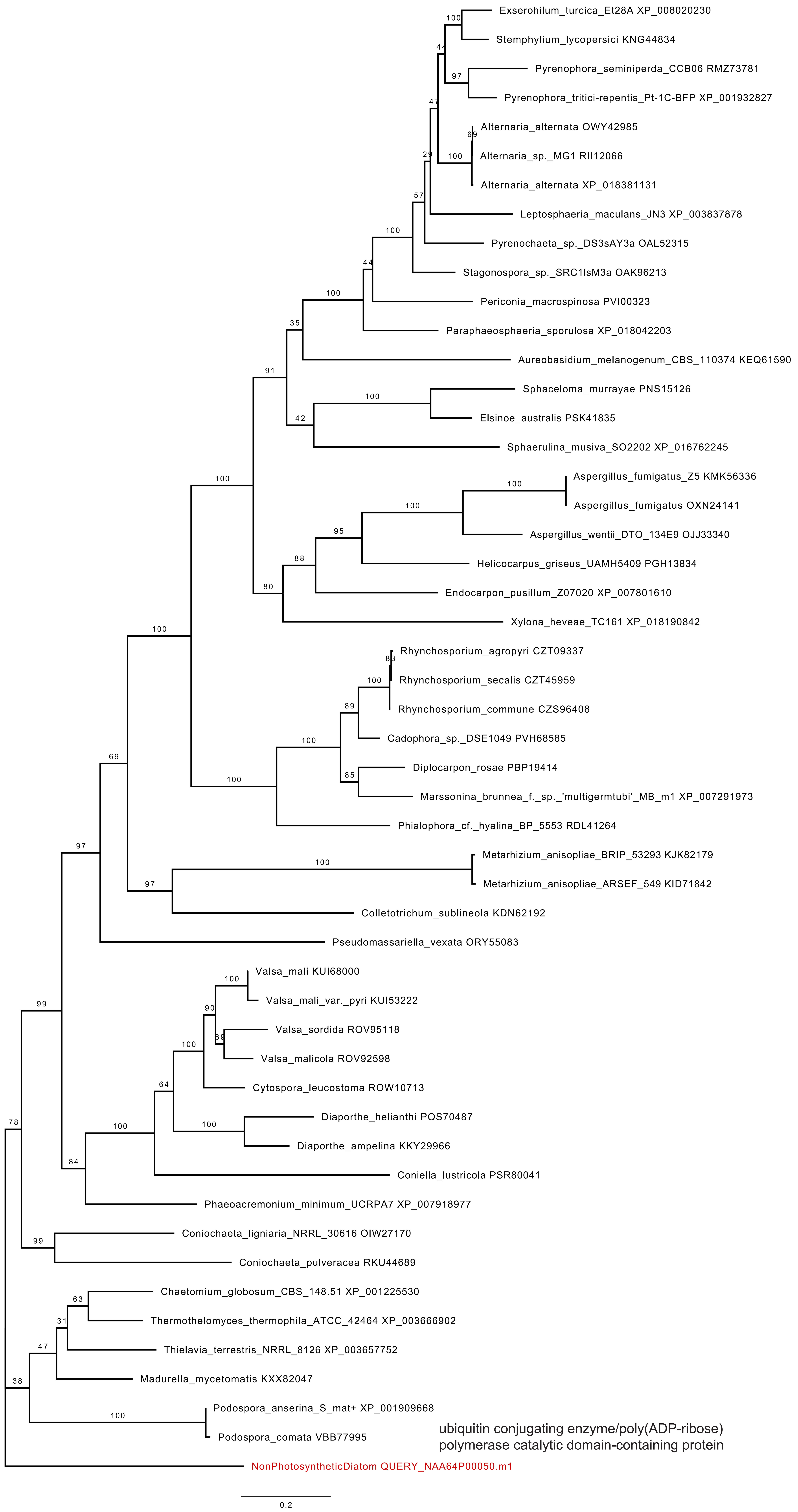

Exserohilum_turcica_Et28A XP_008020230
Stemphylium_lycopersici KNG44834
Pyrenophora_seminiperda_CCB06 RMZ73781
Pyrenophora_tritici-repentis_Pt-1C-BFP XP_001932827
Alternaria_alternata OWY42985
Alternaria_sp._MG1 RII12066
Alternaria_alternata XP_018381131
Leptosphaeria_maculans_JN3 XP_003837878
Pyrenochaeta_sp._DS3sAY3a OAL52315
Stagonospora_sp._SRC1lsM3a OAK96213
Periconia_macrospinosa PVI00323
Paraphaeosphaeria_sporulosa XP_018042203
Aureobasidium_melanogenum_CBS_110374 KEQ61590
Sphaceloma_murrayae PNS15126
Elsinoe_australis PSK41835
Sphaerulina_musiva_SO2202 XP_016762245
Aspergillus_fumigatus_Z5 KMK56336
Aspergillus_fumigatus OXN24141
Aspergillus_wentii_DTO_134E9 OJJ33340
Helicocarpus_griseus_UAMH5409 PGH13834
Endocarpon_pusillum_Z07020 XP_007801610
Xylona_heveae_TC161 XP_018190842
Rhynchosporium_agropyri CZT09337
Rhynchosporium_secalis CZT45959
Rhynchosporium_commune CZS96408
Cadophora_sp._DSE1049 PVH68585
Diplocarpon_rosae PBP19414
Marssonina_brunnea_f._sp._'multigermtubi'_MB_m1 XP_007291973
Phialophora_cf._hyalina_BP_5553 RDL41264
Metarhizium_anisopliae_BRIP_53293 KJK82179
Metarhizium_anisopliae_ARSEF_549 KID71842
Colletotrichum_sublineola KDN62192
Pseudomassariella_vexata ORY55083
Valsa_mali KUI68000
Valsa_mali_var._pyri KUI53222
Valsa_sordida ROV95118
Valsa_malicola ROV92598
Cytospora_leucostoma ROW10713
Diaporthe_helianthi POS70487
Diaporthe_ampelina KKY29966
Coniella_lustricola PSR80041
Phaeoacremonium_minimum_UCRPA7 XP_007918977
Coniochaeta_ligniaria_NRRL_30616 OIW27170
Coniochaeta_pulveracea RKU44689
Chaetomium_globosum_CBS_148.51 XP_001225530
Thermothelomyces_thermophila_ATCC_42464 XP_003666902
Thielavia_terrestris_NRRL_8126 XP_003657752
Madurella_mycetomatis KXX82047
Podospora_anserina_S_mat+ XP_001909668
Podospora_comata VBB77995
ubiquitin conjugating enzyme/poly(ADP-ribose)
polymerase catalytic domain-containing protein
NonPhotosyntheticDiatom QUERY_NAA64P00050.m1
0.2

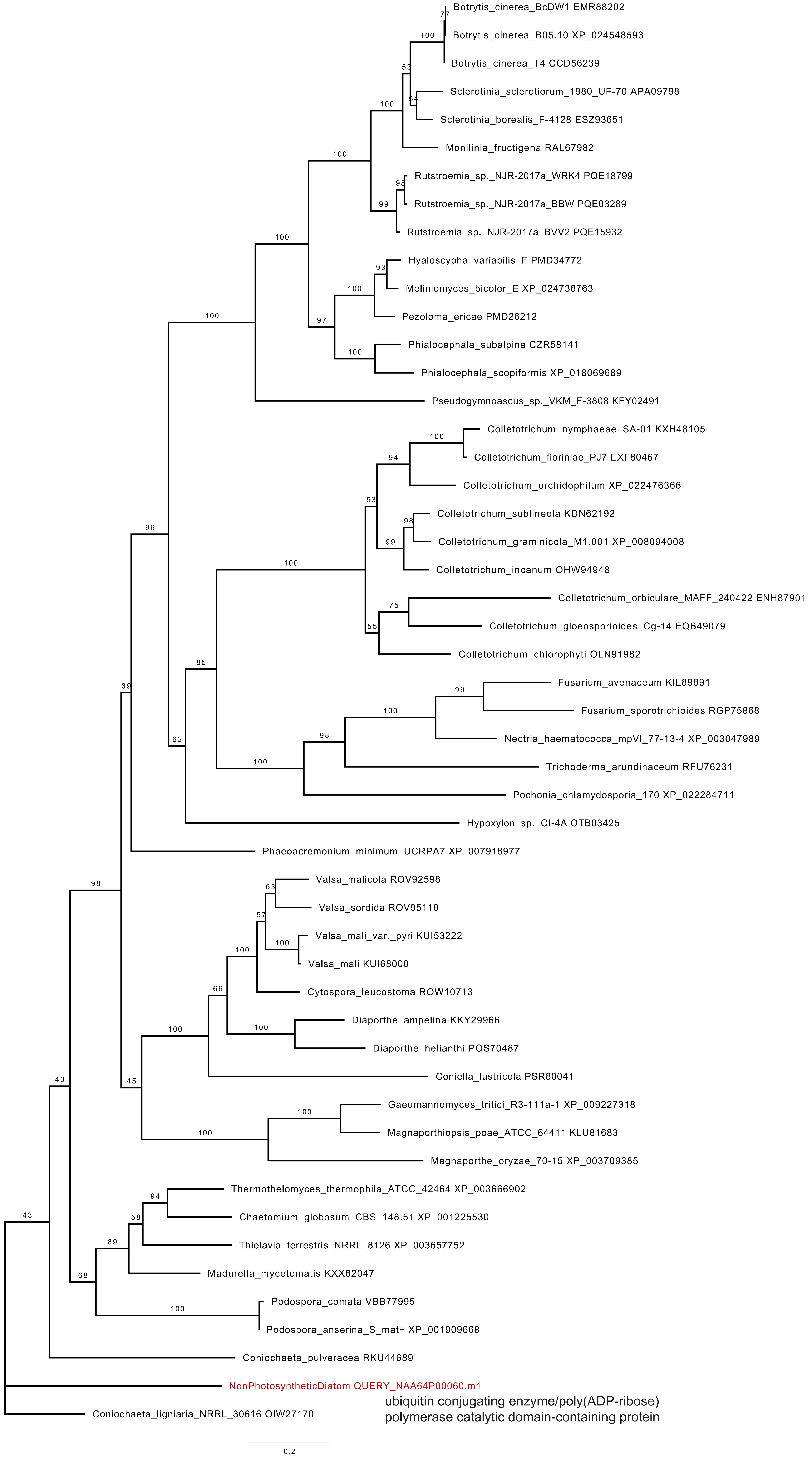

ubiquitin conjugating enzyme/poly(ADP-ribose) polymerase catalytic domain-containing protein

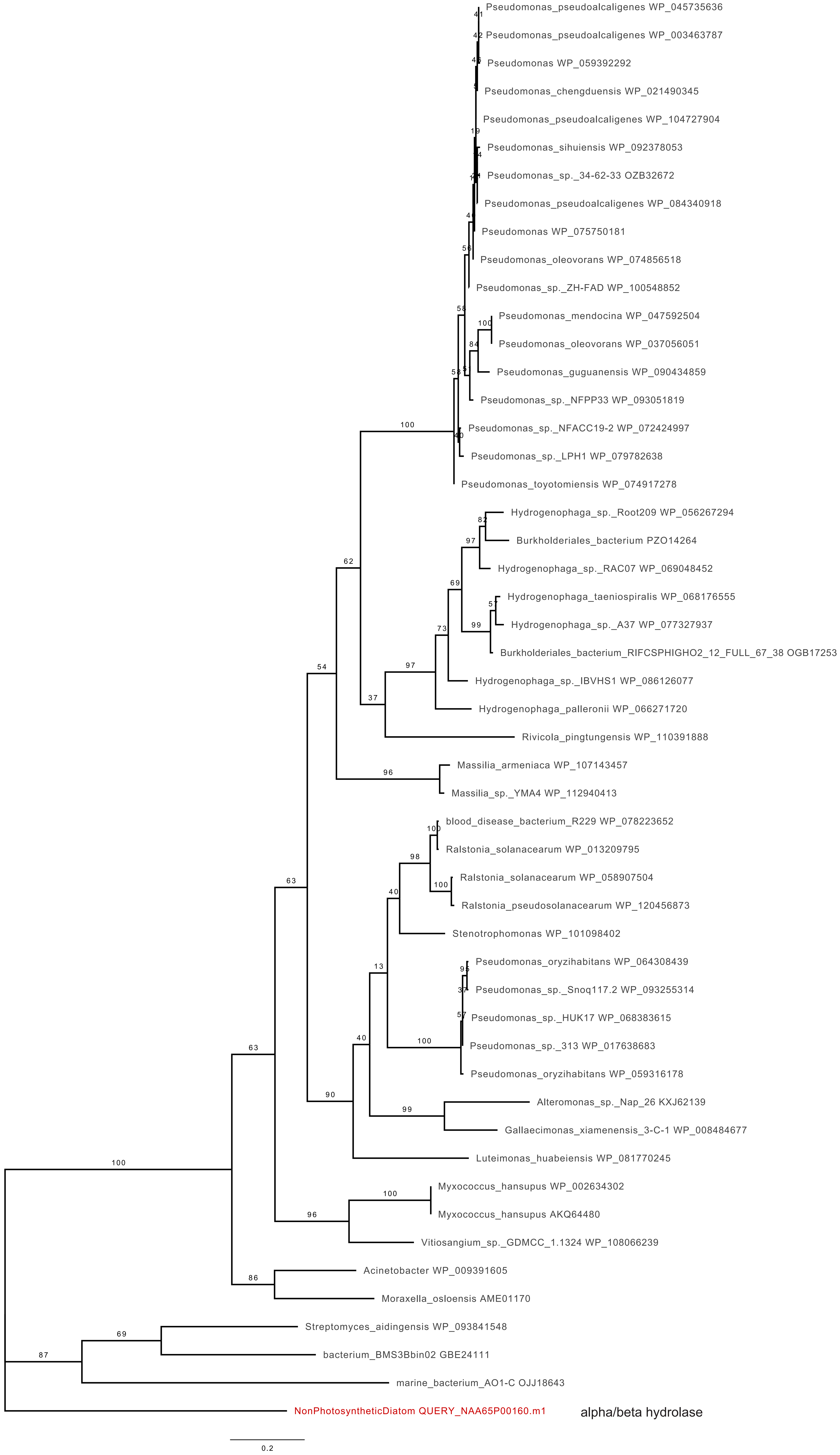
Pseudomonas_pseudoalcaligenes WP_045735636
Pseudomonas_pseudoalcaligenes WP_003463787
Pseudomonas WP_059392292
Pseudomonas_chengduensis WP_021490345
Pseudomonas_pseudoalcaligenes WP_104727904
Pseudomonas_sihuiensis WP_092378053
Pseudomonas_sp._34-62-33 OZB32672
Pseudomonas_pseudoalcaligenes WP_084340918
Pseudomonas WP_075750181
Pseudomonas_oleovorans WP_074856518
Pseudomonas_sp._ZH-FAD WP_100548852
Pseudomonas_mendocina WP_047592504
Pseudomonas_oleovorans WP_037056051
Pseudomonas_guguanensis WP_090434859
Pseudomonas_sp._NFPP33 WP_093051819
Pseudomonas_sp._NFACC19-2 WP_072424997
Pseudomonas_sp._LPH1 WP_079782638
Pseudomonas_toyotomiensis WP_074917278
Hydrogenophaga_sp._Root209 WP_056267294
Burkholderiales_bacterium PZO14264
Hydrogenophaga_sp._RAC07 WP_069048452
Hydrogenophaga_taeniospiralis WP_068176555
Hydrogenophaga_sp._A37 WP_077327937
Burkholderiales_bacterium_RIFCSPHIGHO2_12_FULL_67_38 OGB17253
Hydrogenophaga_sp._IBVHS1 WP_086126077
Hydrogenophaga_palleronii WP_066271720
Rivicola_pingtungensis WP_110391888
Massilia_armeniaca WP_107143457
Massilia_sp._YMA4 WP_112940413
blood_disease_bacterium_R229 WP_078223652
Ralstonia_solanacearum WP_013209795
Ralstonia_solanacearum WP_058907504
Ralstonia_pseudosolanacearum WP_120456873
Stenotrophomonas WP_101098402
Pseudomonas_oryzihabitans WP_064308439
Pseudomonas_sp._Snoq117.2 WP_093255314
Pseudomonas_sp._HUK17 WP_068383615
Pseudomonas_sp._313 WP_017638683
Pseudomonas_oryzihabitans WP_059316178
Alteromonas_sp._Nap_26 KXJ62139
Gallaecimonas_xiamenensis_3-C-1 WP_008484677
Luteimonas_huabeiensis WP_081770245
Myxococcus_hansupus WP_002634302
Myxococcus_hansupus AKQ64480
Vitiosangium_sp._GDMCC_1.1324 WP_108066239
Acinetobacter WP_009391605
Moraxella_osloensis AME01170
Streptomyces_aidingensis WP_093841548
bacterium_BMS3Bbin02 GBE24111
marine_bacterium_AO1-C OJJ18643
NonPhotosyntheticDiatom QUERY_NAA65P00160.m1
alpha/beta hydrolase
0.2

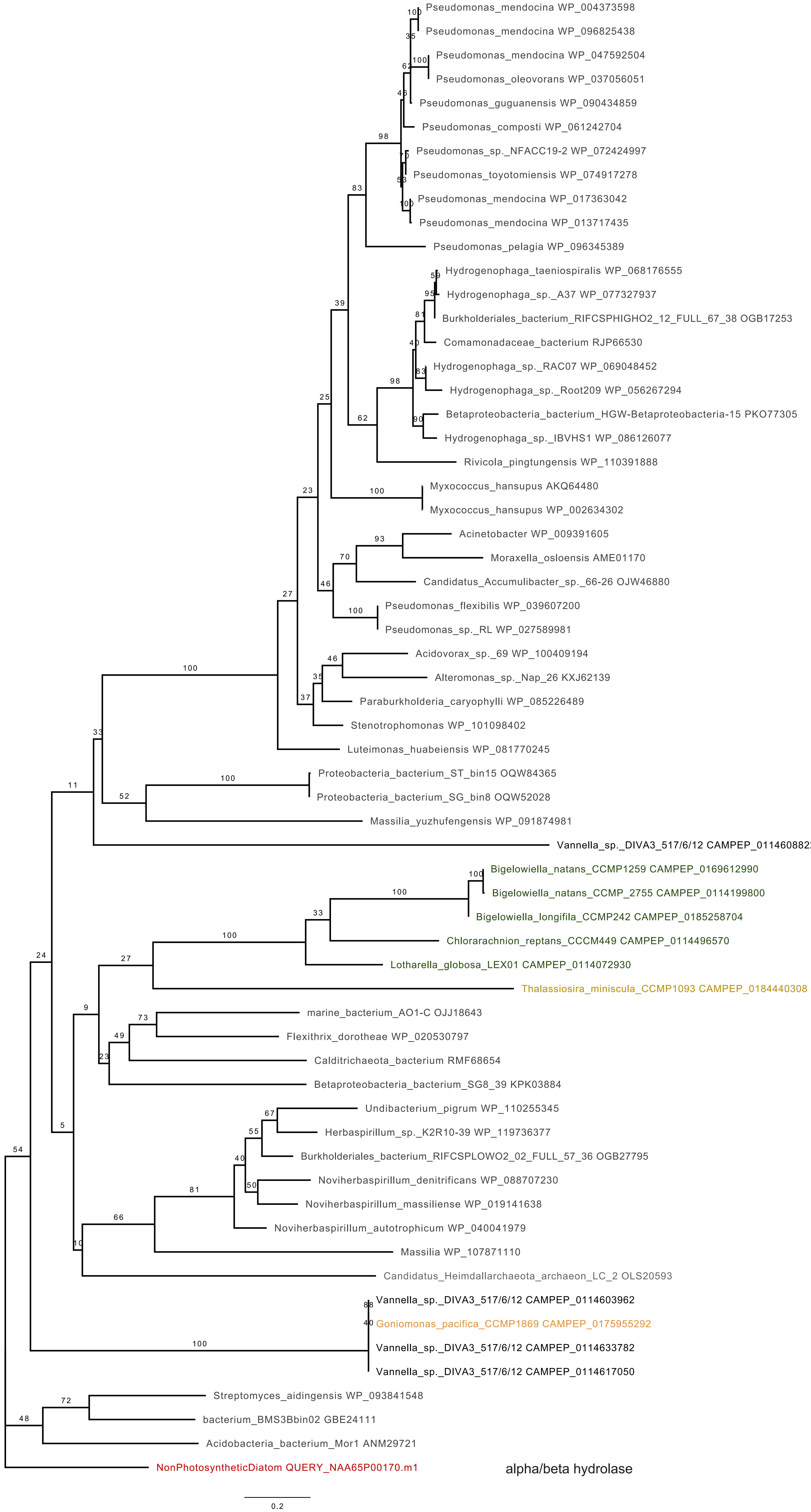
Pseudomonas_mendocina WP_004373598
Pseudomonas_mendocina WP_096825438
Pseudomonas_mendocina WP_047592504
Pseudomonas_oleovorans WP_037056051
Pseudomonas_guguanensis WP_090434859
Pseudomonas_composti WP_061242704
Pseudomonas_sp._NFACC19-2 WP_072424997
Pseudomonas_toyotomiensis WP_074917278
Pseudomonas_mendocina WP_017363042
Pseudomonas_mendocina WP_013717435
Pseudomonas_pelagia WP_096345389
Hydrogenophaga_taeniospiralis WP_068176555
Hydrogenophaga_sp._A37 WP_077327937
Burkholderiales_bacterium_RIFCSPHIGHO2_12_FULL_67_38 OGB17253
Comamonadaceae_bacterium RJP66530
Hydrogenophaga_sp._RAC07 WP_069048452
Hydrogenophaga_sp._Root209 WP_056267294
Betaproteobacteria_bacterium_HGW-Betaproteobacteria-15 PKO77305
Hydrogenophaga_sp._IBVHS1 WP_086126077
Rivicola_pingtungensis WP_110391888
Myxococcus_hansupus AKQ64480
Myxococcus_hansupus WP_002634302
Acinetobacter WP_009391605
Moraxella_osloensis AME01170
Candidatus_Accumulibacter_sp._66-26 OJW46880
Pseudomonas_flexibilis WP_039607200
Pseudomonas_sp._RL WP_027589981
Acidovorax_sp._69 WP_100409194
Alteromonas_sp._Nap_26 KXJ62139
Paraburkholderia_caryophylli WP_085226489
Stenotrophomonas WP_101098402
Luteimonas_huabeiensis WP_081770245
Proteobacteria_bacterium_ST_bin15 OQW84365
Proteobacteria_bacterium_SG_bin8 OQW52028
Massilia_yuzhufengensis WP_091874981
Vannella_sp._DIVA3_517/6/12 CAMPEP_0114608822
Bigelowiella_natans_CCMP1259 CAMPEP_0169612990
Bigelowiella_natans_CCMP_2755 CAMPEP_0114199800
Bigelowiella_longifila_CCMP242 CAMPEP_0185258704
Chlorarachnion_reptans_CCCM449 CAMPEP_0114496570
Lotharella_globosa_LEX01 CAMPEP_0114072930
Thalassiosira_miniscula_CCMP1093 CAMPEP_0184440308
marine_bacterium_AO1-C OJJ18643
Flexithrix_dorotheae WP_020530797
Calditrichaeota_bacterium RMF68654
Betaproteobacteria_bacterium_SG8_39 KPK03884
Undibacterium_pigrum WP_110255345
Herbaspirillum_sp._K2R10-39 WP_119736377
Burkholderiales_bacterium_RIFCSPLOWO2_02_FULL_57_36 OGB27795
Noviherbaspirillum_denitrificans WP_088707230
Noviherbaspirillum_massiliense WP_019141638
Noviherbaspirillum_autotrophicum WP_040041979
Massilia WP_107871110
Candidatus_Heimdallarchaeota_archaeon_LC_2 OLS20593
Vannella_sp._DIVA3_517/6/12 CAMPEP_0114603962
Goniomonas_pacifica_CCMP1869 CAMPEP_0175955292
Vannella_sp._DIVA3_517/6/12 CAMPEP_0114633782
Vannella_sp._DIVA3_517/6/12 CAMPEP_0114617050
Streptomyces_aidingensis WP_093841548
bacterium_BMS3Bbin02 GBE24111
Acidobacteria_bacterium_Mor1 ANM29721
NonPhotosyntheticDiatom QUERY_NAA65P00170.m1
alpha/beta hydrolase
0.2

Fig. S15.

Phylogeny of 70 laterally transferred genes in *N. putrida*. Those sequences were thought to have been gained after speciation as their homologes were not detected even in closely related photosynthetic species in the genus *Nitzschia*. Numbers on branches are maximum likelihood bootstrap values. Highlighted in red are sequences of *N. putrida*. The LGT candidates include five secreted protein sequences (four proteins with unknown functions and polygalacturonase).

Table S1.

Genomes of non-photosynthetic and photosynthetic diatoms. *1: including diverged alleles. Numbers in parentheses are of total numbers in primary contigs and haplotigs.

**Species**	***Nitzschia putrida***	***Fragilariopsis cylindrus***	***Phaeodactylum tricornutum***	***Thalassiosira pseudonana***
**Genome size (Mb)**	35 (47)	61	27	32
**N50 (Mb)**	0.86 (0.55)	1.3	0.945	1.9
**G+C (%)**	47.6 (47.6)	39.8	48.8	47
**Gene count**	15,003 (20,461)	21,066*1	10,402	11,776
**Av gene length (bp)**	1611 (1619)	1575	1511	1553
**Av exon length (bp)**	901 (907)	625	842	612
**Av intron length (bp)**	122 (121)	245	135	124
**Av exon number/gene**	1.69 (1.68)	2.08	1.79	2.49

Table S2.

Tests for positive selection among Silicon Transporter (SIT) codons using Codeml site models. a: Average dN/dS over all sites, b: likelihood ratio test statistic. Calculated with lr.test from extRemes package in R, and c: inferred with Bayes Empirical Bayes at the 50% (95%) posterior probability cutoff.

**Model**	**dN/dS[a]**	**lnL**	**2ΔL[b]**	**Estimates of parameters**	**Positively selected sites (BEB)[c]**
**M0 (one ratio)**	0.1018	-3893.91	-	ω=0.1018	NA
**M3 (discrete)**	0.1422	-3804.88	178.05, df=4, *p*-value=<2.2e-16	p0=0.90449, p1=0.09137, p2=0.00413, ω0=0.02266, ω1=0.93222, ω2=8.85203	0
**M1a (neutral)**	0.1111	-3813.95	-	p0=0.90976, p1=0.09024, ω0=0.02292, ω1=1.00000	NA
**M2a (selection)**	0.1452	-3804.93	18.04, df=2, *p*-value=0.000121	p0=0.90887, p1=0.08718, p2=0.00395, ω0=0.02394, ω1=1.00000, ω2=9.17486	8(2)
**M7 (beta)**	0.1192	-3815.95	-	p=0.03898, q=0.28910	NA
**M8 (beta & ω)**	0.1401	-3805.67	20.57, df=2, *p*-value=3.42e-05	p0=0.99541, p=0.06255, q=0.53977, p1=0.00459, ω=8.13565	12(3)

Table S3. CAZyme genes detected as differentially expressed genes. Differentially expressed genes were detected with the Trinity package (*57,58*). CAZyme genes upregulated in the starch-rich medium are indicated by asterisks.

Loci	Encoded CAZymes	TPM (transcripts per million)					
		Starvation	Starvation	Glucose	Glucose	Starch	Starch
NAA07P00800.m1*	GT96	1.18	0.84	2.48	1.67	5.99	10.54
NAA01P01040.m1*	GT2	1.94	3.45	1.7	2.34	5.39	8.98
NAA15P00430.m1*	GH26	0.39	0.76	1.97	2.04	4.54	4
NAA02P03360.m1*	GH161	4.95	3.93	6.54	3.93	24.45	21.96
NAA05P04290.m1*	GT22	5.48	7.78	39.67	31.79	21	13.63
NAA00P02260.m1	GH29	1.45	2.07	16.48	15.31	5.74	4.78
NAA03P05150.m1	CE4	6.84	6.97	34.95	29.13	31.44	27.5
NAA22P00790.m1	GT2	0.59	0.41	3.05	3.07	0.94	0.72
NAA26P01470.m1	GH17	13.39	12.44	4.47	3.24	7.54	5.13

REFERENCES AND NOTES

1. J. M. Freesec, C. E. Lane, Parasitism finds many solutions to the same problems in red algae (Florideophyceae, Rhodophyta). *Mol. Biochem. Parasitol.* **214**, 105–111 (2017).

2. L. Hadariová, M. Vesteg, V. Hampl, J. Krajčovič, Reductive evolution of chloroplasts in non-photosynthetic plants, algae and protists. *Curr. Genet.* **64**, 365–387 (2018).

3. J. Janouškovec, G. G. Paskerova, T. S. Miroliubova, K. V. Mikhailov, T. Birley, V. V. Aleoshin, T. G. Simdyanov, Apicomplexan-like parasites are polyphyletic and widely but selectively dependent on cryptic plastid organelles. *eLife* **8**, e49662 (2019).

4. J. C. Kissinger, B. P. Brunk, J. Crabtree, M. J. Fraunholz, B. Gajria, A. J. Milgram, D. S. Pearson, J. Schug, A. Bahl, S. J. Diskin, H. Ginsburg, G. R. Grant, D. Gupta, P. Labo, L. Li, M. D. Mailman, S. K. McWeeney, P. Whetzel, C. J. Stoeckert Jr., D. S. Roos, The *Plasmodium* genome database. *Nature* **419**, 490–492 (2002).

5. R. Kamikawa, N. Yubuki, M. Yoshida, M. Taira, N. Nakamura, K. Ishida, B. S. Leander, H. Miyashita, T. Hashimoto, S. Mayama, Y. Inagaki, Multiple losses of photosynthesis in *Nitzschia* (Bacillariophyceae). *Phycol. Res.* **63**, 19–28 (2015).

6. R. G. Dorrell, T. Azuma, M. Nomura, G. Audren de Kerdrel, L. Paoli, S. Yang, C. Bowler, K. Ishii, H. Miyashita, G. H. Gile, R. Kamikawa, Principles of plastid reductive evolution illuminated by nonphotosynthetic chrysophytes. *Proc. Natl. Acad. Sci. U.S.A.* **116**, 6914–6923 (2019).

7. M. Kayama, J. F. Chen, T. Nakada, Y. Nishimura, T. Shikanai, T. Azuma, H. Miyashita, S. Takaichi, Y. Kashiyama, R. Kamikawa, A non-photosynthetic green alga illuminates the reductive evolution of plastid electron transport systems. *BMC Biol.* **18**, 126 (2020).

8. M. Kayama, K. Maciszewski, A. Yabuki, H. Miyashita, A. Karnkowska, R. Kamikawa, Highly reduced plastid genomes of the non-photosynthetic dictyochophyceans *Pteridomonas* spp. (Ochrophyta, SAR) are retained for tRNA-Glu-based organellar heme biosynthesis. *Front. Plant Sci.* **11**, 602455 (2020).

9. L. Van Valen, Molecular evolution as predicted by natural selection. *J. Mol. Evol.* **3**, 89–101 (1974).

10. G. Sun, Y. Xu, H. Liu, T. Sun, J. Zhang, C. Hettenhausen, G. Shen, J. Qi, Y. Qin, J. Li, L. Wang, W. Chang, Z. Guo, I. T. Baldwin, J. Wu, Large-scale gene losses underlie the genome evolution of parasitic plant *Cuscuta australis. Nat. Commun.* **9**, 2683 (2018).

11. C. W. Li, B. E. Volcani, Four new apochlorotic diatoms. *Br. Phycol. J.* **22**, 375–382 (1987).

12. C. B. Field, M. J. Behrenfeld, J. T. Randerson, P. Falkowski, Primary production of the biosphere: Integrating terrestrial and oceanic components. *Science* **281**, 237–240 (1998).

13. D. G. Mann, The species concept in diatoms. *Phycologia* **38**, 437–495 (1999).

14. E. V. Armbrust, J. A. Berges, C. Bowler, B. R. Green, D. Martinez, N. H. Putnam, S. Zhou, A. E. Allen, K. E. Apt, M. Bechner, M. A. Brzezinski, B. K. Chaal, A. Chiovitti, A. K. Davis, M. S. Demarest, J. C. Detter, T. Glavina, D. Goodstein, M. Z. Hadi, U. Hellsten, M. Hildebrand, B. D. Jenkins, J. Jurka, V. V. Kapitonov, N. Kröger, W. W. Y. Lau, T. W. Lane, F. W. Larimer, J. C. Lippmeier, S. Lucas, M. Medina, A. Montsant, M. Obornik, M. S. Parker, B. Palenik, G. J. Pazour, P. M. Richardson, T. A. Rynearson, M. A. Saito, D. C. Schwartz, K. Thamatrakoln, K. Valentin, A. Vardi, F. P. Wilkerson, D. S. Rokhsar, The genome of the diatom *Thalassiosira pseudonana*: Ecology, evolution, and metabolism. *Science* **306**, 79–86 (2004).

15. C. Bowler, A. E. Allen, J. H. Badger, J. Grimwood, K. Jabbari, A. Kuo, U. Maheswari, C. Martens, F. Maumus, R. P. Otillar, E. Rayko, A. Salamov, K. Vandepoele, B. Beszteri, A. Gruber, M. Heijde, M. Katinka, T. Mock, K. Valentin, F. Verret, J. A. Berges, C. Brownlee, J.-P. Cadoret, A. Chiovitti, C. J.

Choi, S. Coesel, A. De Martino, J. C. Detter, C. Durkin, A. Falciatore, J. Fournet, M. Haruta, M. J. J. Huysman, B. D. Jenkins, K. Jiroutova, R. E. Jorgensen, Y. Joubert, A. Kaplan, N. Kröger, P. G. Kroth, J. La Roche, E. Lindquist, M. Lommer, V. Martin-Jézéquel, P. J. Lopez, S. Lucas, M. Mangogna, K. McGinnis, L. K. Medlin, A. Montsant, M.-P. Oudot-Le Secq, C. Napoli, M. Obornik, M. S. Parker, J.-L. Petit, B. M. Porcel, N. Poulsen, M. Robison, L. Rychlewski, T. A. Rynearson, J. Schmutz, H. Shapiro, M. Siaut, M. Stanley, M. R. Sussman, A. R. Taylor, A. Vardi, P. von Dassow, W. Vyverman, A. Willis, L. S. Wyrwicz, D. S. Rokhsar, J. Weissenbach, E. V. Armbrust, B. R. Green, Y. Van de Peer, I. V. Grigoriev, The *Phaeodactylum* genome reveals the evolutionary history of diatom genomes. *Nature* **456**, 239–244 (2008).

16. T. Mock, R. P. Otillar, J. Strauss, M. McMullan, P. Paajanen, J. Schmutz, A. Salamov, R. Sanges, A. Toseland, B. J. Ward, A. E. Allen, C. L. Dupont, S. Frickenhaus, F. Maumus, A. Veluchamy, T. Wu, K. W. Barry, A. Falciatore, M. I. Ferrante, A. E. Fortunato, G. Glöckner, A. Gruber, R. Hipkin, M. G. Janech, P. G. Kroth, F. Leese, E. A. Lindquist, B. R. Lyon, J. Martin, C. Mayer, M. Parker, H. Quesneville, J. A. Raymond, C. Uhlig, R. E. Valas, K. U. Valentin, A. Z. Worden, E. V. Armbrust, M. D. Clark, C. Bowler, B. R. Green, V. Moulton, C. van Oosterhout, I. V. Grigoriev, Evolutionary genomics of the cold-adapted diatom *Fragilariopsis cylindrus*. *Nature* **541**, 536–540 (2017).

17. G. W. Vurture, F. J. Sedlazeck, M. Nattestad, C. J. Underwood, H. Fang, J. Gurtowski, M. C. Schatz, GenomeScope: Fast reference-free genome profiling from short reads. *Bioinformatics* **33**, 2202–2204 (2017).

18. C. S. Chin, P. Peluso, F. J. Sedlazeck, M. Nattestad, G. T. Concepcion, A. Clum, C. Dunn, R. O'Malley, R. Figueroa-Balderas, A. Morales-Cruz, G. R. Cramer, M. Delledonne, C. Luo, J. R. Ecker, D. Cantu, D. R. Rank, M. C. Schatz, Phased diploid genome assembly with single-molecule real-time sequencing. *Nat. Methods* **13**, 1050–1054 (2016).

19. B. J. Walker, T. Abeel, T. Shea, M. Priest, A. Abouelliel, S. Sakthikumar, C. A. Cuomo, Q. Zeng, J. Wortman, S. K. Young, A. M. Earl, Pilon: An integrated tool for comprehensive microbial variant detection and genome assembly improvement. *PLOS ONE* **9**, e112963 (2014).

20. K. J. Hoff, S. Lange, A. Lomsadze, M. Borodovsky, M. Stanke, BRAKER1: Unsupervised RNA-Seq-based genome annotation with GeneMark-ET and AUGUSTUS. *Bioinformatics* **32**, 767–769 (2016).

21. R. M. Waterhouse, M. Seppey, F. A. Simão, M. Manni, P. Ioannidis, G. Klioutchnikov, E. V. Kriventseva, E. M. Zdobnov, BUSCO applications from quality assessments to gene prediction and phylogenomics. *Mol. Biol. Evol.* **35**, 543–548 (2018).

22. A. Gruber, G. Rocap, P. G. Kroth, E. V. Armbrust E.V. T. Mock, Plastid proteome prediction for diatoms and other algae with secondary plastids of the red lineage. *Plant J.* **81**, 519–528 (2015).

23. D. M. Emms, S. Kelly, OrthoFinder: Solving fundamental biases in whole genome comparisons dramatically improves orthogroup inference accuracy. *Genome Biol.* **16**, 157 (2015).

24. R. Kamikawa, G. Tanifuji, S. A. Ishikawa, K. Ishii, Y. Matsuno, N. T. Onodera, K. Ishida, T. Hashimoto, H. Miyashita, S. Mayama, Y. Inagaki, Proposal of a twin arginine translocator system-mediated constraint against loss of ATP synthase genes from nonphotosynthetic plastid genomes. *Mol. Biol. Evol.* **32**, 2598–2604 (2015).

25. D. Moog, A. Nozawa, Y. Tozawa, R. Kamikawa, Substrate specificity of plastid phosphate transporters in a non-photosynthetic diatom and its implication in evolution of red alga-derived complex plastids. *Sci. Rep.* **10**, 1167 (2020).

26. R. Kamikawa, D. Moog, S. Zauner, G. Tanifuji, K. Ishida, H. Miyashita, H. Mayama, T. Hashimoto, U. G. Maier, J. M. Archibald, Y. Inagaki, A non-photosynthetic diatom reveals early steps of reductive evolution in plastids. *Mol. Biol. Evol.* **34**, 2355–2366 (2017).

27. A. E. Allen, C. L. Dupont, M. Oborník, A. Horák, A. Nunes-Nesi, J. P. McCrow, H. Zheng, D. A. Johnson, H. Hu, A. R. Fernie, C. Bowler, Evolution and metabolic significance of the urea cycle in photosynthetic diatoms. *Nature* **473**, 203–207 (2011).

28. S. R. Smith, C. L. Dupont, J. K. McCarthy, J. T. Broddrick, M. Oborník, A. Horák, Z. Füssy, J. Cihlář, S. Kleessen, H. Zheng, J. P. McCrow, K. K. Hixson, W. L. Araújo, A. Nunes-Nesi, A. Fernie, Z. Nikoloski, B. O. Palsson, A. E. Allen, Evolution and regulation of nitrogen flux through compartmentalized metabolic networks in a marine diatom. *Nat. Commun.* **10**, 4552 (2019).

29. J. Ashworth, S. Coesel, A. Lee, E. V. Armbrust, M. V. Orellana, N. S. Baliga, Genome-wide diel growth state transitions in the diatom *Thalassiosira pseudonana*. *Proc. Natl. Acad. Sci. U.S.A.* **110**, 7518–7523 (2013).

30. M. S. Chauton, P. Winge, T. Brembu, O. Vadstein, A. M. Bones, Gene regulation of carbon fixation, storage, and utilization in the diatom *Phaeodactylum tricornutum* acclimated to light/dark cycles. *Plant Physiol.* **161**, 1034–1048 (2013).

31. S. R. Smith, J. T. F. Gillard, A. B. Kustka, J. P. McCrow, J. H. Badger, H. Zheng, A. M. New, C. L. Dupont, T. Obata, A. R. Fernie, A. E. Allen, Correction: Transcriptional orchestration of the global cellular response of a model pennate diatom to diel light cycling under iron limitation. *PLOS Genet.* **13**, e1006688 (2017).

32. M. J. J. Huysman, C. Martens, K. Vandepoele, J. Gillard, E. Rayko, M. Heijde, C. Bowler, D. Inzé, Y. Van de Peer, L. De Veylder, W. Vyverman, Genome-wide analysis of the diatom cell cycle unveils a novel type of cyclins involved in environmental signaling. *Genome Biol.* **11**, R17 (2010).

33. R. Annunziata, A. Ritter, A. Emidio Fortunato, A. Manzotti, S. Cheminant-Navarro, N. Agier, M. J. J. Huysman, P. Winge, A. M. Bones, F. Bouget, M. Cosentino Lagomarsino, J. Bouly, A. Falciatore, bHLH-PAS protein RITMO1 regulates diel biological rhythms in the marine *diatom Phaeodactylum tricornutum*. *Proc. Natl. Acad. Sci. U.S.A.* **116**, 13137–13142 (2019).

34. P. G. Kroth, C. Wilhelm, T. Kottke, An update on aureochromes: Phylogeny–mechanism–function. *J. Plant Physiol.* **217**, 20–26 (2017).

35. S. Coesel, M. Mangogna, T. Ishikawa, M. Heijde, A. Rogato, G. Finazzi, T. Todo, C. Bowler, A. Falciatore, Diatom PtCPF1 is a new cryptochrome/photolyase family member with DNA repair and transcription regulation activity. *EMBO Rep.* **10**, 655–661 (2009).

36. A. E. Fortunato, M. Jaubert, G. Enomoto, J. Bouly, R. Raniello, M. Thaler, M. Malviya, J. S. Bernardes, F. Rappaport, B. Gentili, M. J. J. Huysman, A. Carbone, C. Bowler, M. Ribera d'Alcalà, M. Ikeuchi, A. Falciatore, Diatom phytochromes reveal the existence of far-red-light-based sensing in the ocean. *Plant Cell* **28**, 616–628 (2016).

37. E. Rayko, F. Maumus, U. Maheswari, K. Jabbari, C. Bowler, Transcription factor families inferred from genome sequences of photosynthetic stramenopiles. *New Phytol.* **188**, 52–66 (2010).

38. G. Kong, Y. Chen, Y. Deng, D. Feng, L. Jiang, L. Wan, M. Li, Z. Jiang, P. Xi, The basic leucine zipper transcription factor PlBZP32 associated with the oxidative stress response is critical for pathogenicity of the lychee downy blight oomycete *Peronophythora litchi*. *mSphere* **5**, e00261-20 (2020).

39. T. Fujiwara, S. Hirooka, O. Ohbayashi, R. Onuma, S. Miyagishima, Relationship between cell cycle and diel transcriptomic changes in metabolism in a unicellular red alga. *Plant Physiol.* **183**, 1484–1501 (2020).

40. T. A. Richards, N. J. Talbot, Horizontal gene transfer in osmotrophs: Playing with public goods. *Nat. Rev. Microbiol.* **11**, 720–727 (2013).

41. T. A. Richards, N. J. Talbot, Osmotrophy. *Curr. Biol.* **28**, R1179–R1180 (2018).

42. K. Ishii, R. Kamikawa, Growth characterization of non-photosynthetic diatoms, *Nitzschia* spp., inhabiting estuarine mangrove forests of Ishigaki Island, Japan. *Plankton Benthos Res.* **12**, 164–170 (2017).

43. A. J. Enright, S. Van Dongen, C. A. Ouzounis, An efficient algorithm for large-scale detection of protein families. *Nucleic Acids Res.* **30**, 1575–1584 (2002).

44. Y. Belkhadir, L. Yang, J. Hetzel, J. L. Dangl, J. Chory, The growth–defense pivot: Crisis management in plants mediated by LRR-RK surface receptors. *Trends Biochem. Sci.* **39**, 447–456 (2014).

45. U. Karsten, A. S. Mostaert, R. J. King, M. Kamiya, Y. Hara, Osmoprotectors in some species of Japanese mangrove macroalgae. *Phycol. Res.* **44**, 109–112 (1996).

46. Y. I. Wolf, E. V. Koonin, Genome reduction as the dominant mode of evolution. *Bioessays* **35**, 829–837 (2013).

47. M. Nei, S. Kumar S, *Molecular Evolution and Phylogenetics* (Oxford Univ. Press, New York, 2000), pp. 17–203

48. J. J. Morris, R. E. Lenski, E. R. Zinser, The Black Queen hypothesis: Evolution of dependencies through adaptive gene loss. *MBio* **3**, e00036-12 (2012).

49. E. A. Ottesen, C. R. Young, S. M. Gifford, J. M. Eppley, R. Marin III, S. C. Schuster, C. A. Scholin, E. F. DeLong, Multispecies diel transcriptional oscillations in open ocean heterotrophic bacterial assemblages. *Science* **345**, 207–212 (2014).

50. F. O. Aylward, J. M. Eppley, J. M. Smith, F. P. Chavez, C. A. Scholin, E. F. DeLong, Microbial community transcriptional networks are conserved in three domains at ocean basin scales. *Proc. Natl. Acad. Sci. U.S.A.* **112**, 5443–5448 (2015).

51. K. R. Frischkorn, S. T. Haley, S. T. Dyhrman, Coordinated gene expression between *Trichodesmium* and its microbiome over day–night cycles in the North Pacific Subtropical Gyre. *ISME J.* **12**, 997–1007 (2018).

52. E. A. Ottesen, C. R. Young, J. M. Eppley, J. P. Ryan, F. P. Chavez, C. A. Scholin, E. F. DeLong, Pattern and synchrony of gene expression among sympatric marine microbial populations. *Proc. Natl. Acad. Sci. U.S.A.* **110**, E488–E497 (2013).

53. M. D. Hernández Limón, G. M. M. Hennon, M. J. Harke, K. R. Frischkorn, S. T. Haley, S. T. Dyhrman, Transcriptional patterns of *Emiliania huxleyi* in the North Pacific Subtropical Gyre reveal the daily rhythms of its metabolic potential. *Environ. Microbiol.* **22**, 381–396 (2020).

54. A. M. Bolger, M. A. Lohse, B. Usadel, Trimmomatic: A flexible trimmer for Illumina sequence data. *Bioinformatics* **30**, 2114–2120 (2014).

55. D. Kim, B. Langmead, S. L. Salzberg, HISAT: A fast spliced aligner with low memory requirements. *Nat. Methods* **12**, 357–360 (2015).

56. B. J. Haas, A. L. Delcher, S. M. Mount, J. R. Wortman, R. K. Smith Jr, L. I. Hannick, R. Maiti, C. M. Ronning, D. B. Rusch, C. D. Town, S. L. Salzberg, O. White, Improving the *Arabidopsis* genome annotation using maximal transcript alignment assemblies. *Nucleic Acids Res.* **31**, 5654–5666 (2003).

57. M. G. Grabherr, B. J. Haas, M. Yassour, J. Z. Levin, D. A. Thompson, I. Amit, X. Adiconis, L. Fan, R. Raychowdhury, Q. Zeng, Z. Chen, E. Mauceli, N. Hacohen, A. Gnirke, N. Rhind, F. di Palma, B. W. Birren, C. Nusbaum, K. Lindblad-Toh, N. Friedman, A. Regev, Full-length transcriptome assembly from RNA-Seq data without a reference genome. *Nat. Biotechnol.* **29**, 644–652 (2011).

58. B. J. Haas, A. Papanicolaou, M. Yassour, M. Grabherr, P. D. Blood, J. Bowden, M. Brian Couger, D. Eccles, B. Li, M. Lieber, M. D. MacManes, M. Ott, J. Orvis, N. Pochet, F. Strozzi, N. Weeks, R. Westerman, T. William, C. N. Dewey, R. Henschel, R. D. LeDuc, N. Friedman, A. Regev, De novo transcript sequence reconstruction from RNA-seq using the Trinity platform for reference generation and analysis. *Nat. Protocol* **8**, 1494–1512 (2013).

59. C. Camacho, G. Coulouris, V. Avagyan, N. Ma, J. Papadopoulos, K. Bealer, T. L. Madden, BLAST^{+}: Architecture and applications. *BMC Bioinformatics* **10**, 421 (2009).

60. S. El-Gebali, J. Mistry, A. Bateman, S. R. Eddy, A. Luciani, S. C. Potter, M. Qureshi, L. J. Richardson, G. A. Salazar, A. Smart, E. L. L. Sonnhammer, L. Hirsh, L. Paladin, D. Piovesan, S. C. E. Tosatto, R. D. Finn, The Pfam protein families database in 2019. *Nucleic Acids Res.* **47**, D427–D432 (2019).

61. M. Tarailo-Graovac, N. Chen, Using RepeatMasker to identify repetitive elements in genomic sequences. *Curr. Protoc. Bioinform.* **25**, 4.10.1–4.10.14 (2009).

62. G. Pertea, M. Pertea, GFF utilities: GffRead and GffCompare. *F1000Research* **9**, 304 (2020).

63. P. Jones, D. Binns, H.-Y. Chang, M. Fraser, W. Li, C. McAnulla, H. McWilliam, J. Maslen, A. Mitchell, G. Nuka, S. Pesseat, A. F. Quinn, A. Sangrador-Vegas, M. Scheremetjew, S.-Y. Yong, R. Lopez, S. Hunter, InterProScan 5: Genome-scale protein function classification. *Bioinformatics* **30**, 1236–1240 (2014).

64. A. Marchler-Bauer, S. H. Bryant, CD-Search: Protein domain annotations on the fly. *Nucleic Acids Res.* **32**, W327–W331 (2004).

65. Moriya Y., Itoh M., Okuda S., Yoshizawa A.C., Kanehisa M. KAAS: An automatic genome annotation and pathway reconstruction server. *Nucleic Acids Res.* **35**, W182–W185 (2007).

66. H. Li, V. A. Benedito, M. K. Udvardi, P. X. Zhao, TransportTP: A two-phase classification approach for membrane transporter prediction and characterization. *BMC Bioinformatics* **10**, 418 (2009).

67. V. Lombard, H. Golaconda Ramulu, E. Drula, P.M. Coutinho, B. Henrissat, The carbohydrate-active enzymes database (CAZy) in 2013. *Nucleic Acids Res.* **42**, D490–D495 (2014).

68. S. F. Altschul, T. L. Madden, A. A. Schäffer, J. Zhang, Z. Zhang, W. Miller, D. J. Lipman, Gapped BLAST and PSI-BLAST: A new generation of protein database search programs. *Nucleic Acids Res.* **25**, 3389–3402 (1997).

69. J. Mistry, R. D. Finn, S. R. Eddy, A. Bateman, M. Punta, Challenges in homology search: HMMER3 and convergent evolution of coiled-coil regions. *Nucleic Acids Res.* **41**, e121 (2013).

70. B. A. Curtis, G. Tanifuji, F. Burki, A. Gruber, M. Irimia, S. Maruyama, M. C. Arias, S. G. Ball, G. H. Gile, Y. Hirakawa, J. F. Hopkins, A. Kuo, S. A. Rensing, J. Schmutz, A. Symeonidi, M. Elias, R. J. M. Eveleigh, E. K. Herman, M. J. Klute, T. Nakayama, M. Oborník, A. Reyes-Prieto, E. V. Armbrust, S. J. Aves, R. G. Beiko, P. Coutinho, J. B. Dacks, D. G. Durnford, N. M. Fast, B. R. Green, C. J. Grisdale, F. Hempel, B. Henrissat, M. P. Höppner, K.-I. Ishida, E. Kim, L. Kořený, P. G. Kroth, Y. Liu, S.-B. Malik, U. G. Maier, D. McRose, T. Mock, J. A. D. Neilson, N. T. Onodera, A. M. Poole, E. J. Pritham, T. A. Richards, G. Rocap, S. W. Roy, C. Sarai, S. Schaack, S. Shirato, C. H. Slamovits, D. F. Spencer, S. Suzuki, A. Z. Worden, S. Zauner, K. Barry, C. Bell, A. K. Bharti, J. A. Crow, J. Grimwood, R. Kramer, E. Lindquist, S. Lucas, A. Salamov, G. I. McFadden, C. E. Lane, P. J. Keeling, M. W. Gray, I. V. Grigoriev,

J. M. Archibald, Algal genomes reveal evolutionary mosaicism and the fate of nucleomorphs. *Nature* **492**, 59–65 (2012).

71. U. Cenci, S. J. Sibbald, B. A. Curtis, R. Kamikawa, L. Eme, D. Moog, B. Henrissat, E. Maréchal, M. Chabi, C. Djemiel, A. J. Roger, E. Kim, J. M. Archibald, Nuclear genome sequence of the plastid-lacking cryptomonad *Goniomonas avonlea* provides insights into the evolution of secondary plastids. *BMC Biol.* **16**, 137 (2018).

72. J. R. Bray, J. T. Curtis, An ordination of the upland forest communities of Southern Wisconsin. *Ecol. Monogr.* **27**, 325–349 (1957).

73. J. H. Ward Jr, Hierarchical grouping to optimize an objective function. *J. Am. Stat. Assoc.* **58**, 236–244 (1963).

74. J. Oksanen, Multivariate Analysis of Ecological Communities in R: Vegan tutorial (2015); https://www.mooreecology.com/uploads/2/4/2/1/24213970/vegantutor.pdf.

75. K. Katoh, D. M. Standley, MAFFT multiple sequence alignment software version 7: Improvements in performance and usability. *Mol. Biol. Evol.* **30**, 772–780 (2013).

76. T. A. Hall, BioEdit: A user-friendly biological sequence alignment editor and analysis program for windows 95/98/NT. *Nucleic Acids Symp. Ser.* **41**, 95–98 (1999).

77. L. T. Nguyen, H. A. Schmidt, A. Von Haeseler, B. Q. Minh, IQ-TREE: A fast and effective stochastic algorithm for estimating maximum-likelihood phylogenies. *Mol. Biol. Evol.* **32**, 268–274 (2015).

78. A. Davis, R. Abbriano, S. R. Smith, M. Hildebrand, Clarification of photorespiratory processes and the role of malic enzyme in diatoms. *Protist* **168**, 134–153 (2017).

79. N. H. Gonzalez, G. Felsner, F. D. Schramm, A. Klingl, U. G. Maier, K. Bolte, A single peroxisomal targeting signal mediates matrix protein import in diatoms. *PLOS ONE* **6**, e25316 (2011).

80. A. K. Mix, U. Cenci, T. Heimerl, P. Marter, M. L. Wirkner, D. Moog, Identification and localization of peroxisomal biogenesis proteins indicates the presence of peroxisomes in the cryptophyte *Guillardia theta* and other "Chromalveolates". *Genome Biol. Evol.* **10**, 2834–2852 (2018).

81. K. Sidiropoulos, G. Viteri, C. Sevilla, S. Jupe, M. Webber, M. Orlic-Milacic, B. Jassal, B. May, V. Shamovsky, C. Duenas, K. Rothfels, L. Matthews, H. Song, L. Stein, R. Haw, P. D'Eustachio, P. Ping, H. Hermjakob, A. Fabregat, Reactome enhanced pathway visualization. *Bioinformatics* **33**, 3461–3467 (2017).

82. A. Fabregat, F. Korninger, G. Viteri, K. Sidiropoulos, P. Marin-Garcia, P. Ping, G. Wu, L. Stein, P. D'Eustachio, H. Hermjakob, Reactome graph database: Efficient access to complex pathway data. *PLOS Comput. Biol.* **14**, e1005968 (2018).

83. A. Fabregat, K. Sidiropoulos, G. Viteri, P. Marin-Garcia, P. Ping, L. Stein, P. D'Eustachio, H. Hermjakob, Reactome diagram viewer: Data structures and strategies to boost performance. *Bioinformatics* **34**, 1208–1214 (2018).

84. B. Jassal, L. Matthews, G. Viteri, C. Gong, P. Lorente, A. Fabregat, K. Sidiropoulos, J. Cook, M. Gillespie, R. Haw, F. Loney, B. May, M. Milacic, K. Rothfels, C. Sevilla, V. Shamovsky, S. Shorser, T. Varusai, J. Weiser, G. Wu, L. Stein, H. Hermjakob, P. D'Eustachio, The reactome pathway knowledgebase. *Nucleic Acids Res.* **48**, D498–D503 (2020).

85. UniProt Consortium, UniProt: A worldwide hub of protein knowledge. *Nucleic Acids Res.* **47**, D506–D515 (2019).

86. S. F. Altschul, W. Gish, W. Miller, E. W. Myers, D. J. Lipman, Basic local alignment search tool. *J. Mol. Biol.* **215**, 403–410 (1990).

87. T. Aramaki, R. Blanc-Mathieu, H. Endo, K. Ohkubo, M. Kanehisa, S. Goto, H. Ogata, KofamKOALA: KEGG Ortholog assignment based on profile HMM and adaptive score threshold. *Bioinformatics* **36**, 2251–2252 (2020).

88. Y. Fukasawa, J. Tsuji, S. C. Fu, K. Tomii, P. Horton, K. Imai, MitoFates: Improved prediction of mitochondrial targeting sequences and their cleavage sites. *Mol. Cell. Proteomics* **14**, 1113–1126 (2015).

89. K. Kume, T. Amagasa, T. Hashimoto, H. Kitagawa, NommPred: Prediction of mitochondrial and mitochondrion-related organelle proteins of nonmodel organisms. *Evol. Bioinform. Online* **14**, 1176934318819835 (2018).

90. T. N. Petersen, S. Brunak, G. von Heijne, H. Nielsen, SignalP 4.0: Discriminating signal peptides from transmembrane regions. *Nat. Methods* **8**, 785–786 (2011).

91. M. Kanehisa, S. Goto, Y. Sato, M. Furumichi, M. Tanabe, KEGG for integration and interpretation of large-scale molecular data sets. *Nucleic Acids Res.* **40**, D109–D114 (2012).

92. E. L. Sonnhammer, G. von Heijne, A. Krogh, A hidden Markov model for predicting transmembrane helices in protein sequences. *Proc. Int. Conf. Intell. Syst. Mol. Biol.* **6**, 175–182 (1998).

93. A. Krogh, B. Larsson, G. von Heijne, E. L. Sonnhammer, Predicting transmembrane protein topology with a hidden markov model: Application to complete genomes. *J. Mol. Biol.* **305**, 567–580 (2001).

94. C. G. Bruckner, C. Rehm, H. P. Grossart, P. G. Kroth, Growth and release of extracellular organic compounds by benthic diatoms depend on interactions with bacteria. *Environ. Microbiol.* **13**, 1052–1063 (2011).

95. M. T. Buhmann, B. Schulze, A. Förderer, D. Schleheck, P. G. Kroth, Bacteria may induce the scretion of mucin-like proteins by the diatom *Phaeodactylum tricornutum. J. Phycol.* **52**, 463–474 (2016).

96. M. Lachnit, M. T. Buhmann, J. Klemm, N. Kröger, N. Poulsen, Identification of proteins in the adhesive trails of the diatom *Amphora coffeaeformis*. *Philos. Trans. R. Soc. B* **374**, 20190196 (2019).

97. G. Dell'Aquila, S. Zauner, T. Heimerl, J. Kahnt, V. Samel-Gondesen, S. Runge, F. Hempel, U. G. Maier, Mobilization and cellular distribution of phosphate in the diatom *Phaeodactylum tricornutum. Front. Plant Sci.* **11**, 579 (2020).

98. E. G. Bligh, W. J. Dyer, A rapid method of total lipid extraction and purification. *Can. J. Biochem. Physiol.* **37**, 911–917 (1959).

99. E. Mitani, F. Nakayama, I. Matsuwaki, I. Ichi, A. Kawabata, M. Kawachi, M. Kato, Fatty acid composition profiles of 235 strains of three microalgal divisions within the NIES microbial culture collection. *Microb. Resour. Syst.* **33**, 19–29 (2017).

100. R. S. Wright, A reagent for the non-destructive location of steroids and some other lipophilic materials on silica gel thin-layer chromatograms. *J. Chromatogr.* **59**, 220–221 (1971).

101. N. Sato, Lipids in *Cryptomonas* CR-1. I. Occurrence of betaine lipids. *Plant Cell. Physiol.* **32**, 819–825 (1991).

102. C. F. Allen, P. Good, [48] Acyl lipids in photosynthetic systems. *Methods Enzymol.* **23**, 523–547 (1971).

103. L. K. Johnson, H. Alexander, C. T. Brown, Re-assembly, quality evaluation, and annotation of 678 microbial eukaryotic reference transcriptomes. *GigaScience* **8**, giy158 (2019).

104. A. Criscuolo, S. Gribaldo, BMGE (Block Mapping and Gathering with Entropy): A new software for selection of phylogenetic informative regions from multiple sequence alignments. *BMC Evol. Biol.* **10**, 210 (2010).

105. B. Langmead, S. L. Salzberg, Fast gapped-read alignment with Bowtie 2. *Nat. Methods* **9**, 357–359 (2012).

106. H. Li, B. Handsaker, A. Wysoker, T. Fennell, J. Ruan, N. Homer, G. Marth, G. Abecasis, R. Durbin; 1000 Genome Project Data Processing Subgroup, The sequence alignment/map format and SAMtools. *Bioinformatics* **25**, 2078–2079 (2009).

107. A. R. Quinlan, I. M. Hall, BEDTools: A flexible suite of utilities for comparing genomic features. *Bioinformatics* **26**, 841–842 (2010).

108. R. Ihaka, R. Gentleman, R: A language for data analysis and graphics. *J. Comp. Graph. Stat.* **5**, 299–314 (1996).

109. M. J. L. de Hoon, S. Imoto, J. Nolan, S. Miyano, Open source clustering software. *Bioinformatics* **20**, 1453–1454 (2004).

110. A. J. Saldanha, Java Treeview - Extensible visualization of microarray data. *Bioinformatics* **20**, 3246–3248 (2004).

111. A. Löytynoja, Phylogeny-aware alignment with PRANK. *Methods Mol. Biol.* **1079**, 155–170 (2014).

112. S. Capella-Gutiérrez, J. M. Silla-Martínez, T. Gabaldón, trimAl: A tool for automated alignment trimming in large-scale phylogenetic analyses. *Bioinformatics* **25**, 1972–1973 (2009).

113. G. Talavera, J. Castresana, Improvement of phylogenies after removing divergent and ambiguously aligned blocks from protein sequence alignments. *Syst. Biol.* **56**, 564–577 (2007).

114. R. Bouckaert, T. G. Vaughan, J. Barido-Sottani, S. Duchêne, M. Fourment, A. Gavryushkina, J. Heled, G. Jones, D. Kühnert, N. De Maio, M. Matschiner, BEAST 2.5: An advanced software platform for Bayesian evolutionary analysis. *PLOS Comput. Biol.* **15**, e1006650 (2019).

115. S. Höhna, M. R. May, B. R. Moore, TESS: An R package for efficiently simulating phylogenetic trees and performing Bayesian inference of lineage diversification rates. *Bioinformatics* **32**, 789–791 (2016).

116. Z. Yang, W. S. W. Wong, R. Nielsen, Bayes empirical bayes inference of amino acid sites under positive selection. *Mol. Biol. Evol.* **22**, 1107–1118 (2005)

117. Z. Yang, PAML 4: Phylogenetic analysis by maximum likelihood. *Mol. Biol. Evol.* **24**, 1586–1591 (2007).

118. J.-F. Pombert, N. A. Blouin, C. Lane, D. Boucias, P. J. Keeling, A lack of parasitic reduction in the obligate parasitic green alga *Helicosporidium*. *PLOS Genet.* **10**, e1004355 (2014).

119. S. Suzuki, R. Endoh, R. Manabe, M. Ohkuma, Y. Hirakawa, Multiple losses of photosynthesis and convergent reductive genome evolution in the colourless green algae *Prototheca*. *Sci. Rep.* **8**, 940 (2018).

120. C. A. Durkin, J. A. Koester, S. J. Bender, E. V. Armbrust, The evolution of silicon transporters in diatoms. *J. Phycol.* **52**, 716–731 (2016).
